# MetAP1 and MetAP2 drive cell selectivity for a potent anti-cancer agent in synergy, by controlling glutathione redox state

**DOI:** 10.18632/oncotarget.11216

**Published:** 2016-08-11

**Authors:** Frédéric Frottin, Willy V. Bienvenut, Jérôme Bignon, Eric Jacquet, Alvaro Sebastian Vaca Jacome, Alain Van Dorsselaer, Sarah Cianferani, Christine Carapito, Thierry Meinnel, Carmela Giglione

**Affiliations:** ^1^ Institute for Integrative Biology of the Cell (I2BC), CEA, CNRS, Univ. Paris-Sud, Univ. Paris-Saclay, 91198 Gif-sur-Yvette Cedex, France; ^2^ Institut de Chimie des Substances Naturelles, UPR2301, CNRS avenue de la terrasse, Gif sur Yvette Cedex, France; ^3^ Laboratoire de Spectrométrie de Masse BioOrganique (LSMBO), IPHC, Université de Strasbourg, CNRS, UMR7178, 67087 Strasbourg, France; ^4^ Present address: Department of Cellular Biochemistry, Max Planck Institute of Biochemistry, 82159 Martinsried, Germany

**Keywords:** cotranslational modifications, quantitative targeted proteomics, methionine aminopeptidase, N-terminal processing, glutathione redox homeostasis

## Abstract

Fumagillin and its derivatives are therapeutically useful because they can decrease cancer progression. The specific molecular target of fumagillin is methionine aminopeptidase 2 (MetAP2), one of the two MetAPs present in the cytosol. MetAPs catalyze N-terminal methionine excision (NME), an essential pathway of cotranslational protein maturation. To date, it remains unclear the respective contribution of MetAP1 and MetAP2 to the NME process *in vivo* and why MetAP2 inhibition causes cell cycle arrest only in a subset of cells. Here, we performed a global characterization of the N-terminal methionine excision pathway and the inhibition of MetAP2 by fumagillin in a number of lines, including cancer cell lines. Large-scale N-terminus profiling in cells responsive and unresponsive to fumagillin treatment revealed that both MetAPs were required *in vivo* for M[VT]X-targets and, possibly, for lower-level M[G]X-targets. Interestingly, we found that the responsiveness of the cell lines to fumagillin was correlated with the ability of the cells to modulate their glutathione homeostasis. Indeed, alterations to glutathione status were observed in fumagillin-sensitive cells but not in cells unresponsive to this agent. Proteo-transcriptomic analyses revealed that both MetAP1 and MetAP2 accumulated in a cell-specific manner and that cell sensitivity to fumagillin was related to the levels of these MetAPs, particularly MetAP1. We suggest that MetAP1 levels could be routinely checked in several types of tumor and used as a prognostic marker for predicting the response to treatments inhibiting MetAP2.

## INTRODUCTION

N-terminal methionine excision (NME) is an essential cotranslational pathway and is the first modification to which proteins are subjected, whilst still bound to the ribosome and before their synthesis has even been completed [for a reviews see 1, 2, 3]. In all living organisms and cell compartments in which polypeptides are synthesized, proteins are always initially generated with a methionine as their first residue (iMet). However, this iMet is specifically removed from most of the proteins accumulating at steady state. It has remained unclear for decades why up to two thirds of the proteins in any proteome undergo NME. The strong conservation of NME throughout evolution suggests that this process must have an important function in all organisms. Over 25 years ago, it was suggested that NME played a critical role in protein half-life [4]. Recent advances have provided strong support for the “protein stability” hypothesis, bridging the gap between NME and protein half-life [for reviews see 5, 6]. Moreover, through its global control of protein half-life, NME has been shown to fine tune global glutathione redox homeostasis in plants, yeast and Archaea [7].

During NME, the iMet is removed by Met aminopeptidase (MetAP). In the eukaryotic cytosol, NME is catalyzed by two classes of MetAPs: MetAP1 and MetAP2. MetAP1s are derived from the eubacterial enzyme, whereas MetAP2s originated from Archaea. The reason for the presence of two types of MetAPs in the cytoplasm remains unclear. The major defects observed in mammals, *Drosophila*, *C. elegans* and yeast when one of the two types of MetAPs is knocked out or its expression knocked down [8–12] suggest that the substrate specificities of MetAP1 and MetAP2 may be slightly different. However, regardless of the organism concerned, the two types of MetAPs have very similar substrate specificities *in vitro* [13], and they are interchangeable in plants [7, 14].

NME was long considered a constitutive pathway, because MetAPs routinely act on most of the proteins present in the proteome. However, many studies have shown that NME enzymes are tightly regulated during development and tumorigenesis and during the response to abiotic and biotic stresses [15–19]. Nevertheless, the impact of the regulation of MetAP expression on the modifications to the entire proteome of different cell types has yet to be determined.

Interest in human MetAP enzymes has increased since the fortuitous identification of MetAP2 as the specific target of previously identified antiangiogenic drugs, such as fumagillin [20, 21]. Indeed, fumagillin binds and specifically inhibits MetAP2, but not MetAP1 [7, 14, 20, 21]. Fumagillin was first discovered in the early 1950s, based on its deleterious effects in ameba (*Entamoeba sp.*) [22]. Consistent with the presence of a MetAP2 gene but no MetAP1 gene in the minimal genome of the microsporidian parasite *E. cuniculi*, fumagillin was found to be effective against the class of obligate human intracellular protists including *Enterocytozoon* and *Encephalitozoon spp*. These parasites cause various clinical syndromes in immunocompromised patients, and fumagillin is one of the few drugs active against these diseases. Fumagillin also seems to inhibit the growth of the parasites responsible for malaria and leishmaniosis, two of the most important parasites affecting humans [23]. However, as these organisms have both MetAP genes, the mechanism by which MetAP2 inhibition causes growth arrest is unclear. Moreover, it has been shown that fumagillin and its derivatives inhibit the proliferation not only of endothelial cell lines, but also of a subset of cancer cell lines [18]. This finding, and the observation that MetAP2 protein is overproduced in several tumor cells [18], suggested that MetAP2 was a promising novel target for cancer therapy. Consistent with this hypothesis, TNP-470, a fumagillin analog, proved to be effective for treating solid tumors and arthritis in several animal studies and preclinical trials [24]. The first generation of these compounds was found to have the broadest anticancer spectrum of any agent tested, but phase III clinical trials revealed that these agents were neurotoxic at the concentrations required for tumor regression [25]. To date, only a few new molecules exclusively targeting MetAP2 have been developed (for a complete review see [24] and [26]). These compounds are more potent and less toxic than the original molecules, but none of them have reached advanced phases of clinical testing. The main difficulty in finding new potent and effective drugs active against MetAP2 enzymes remains our limited knowledge of the physiological function of the NME process in which these enzymes are involved and our even poorer understanding of the molecular basis of the consequences of MetAP2 inhibition. In other words, the problem is that we still do not know why fumagillin and its derivatives specifically inhibit the growth of endothelial cell lines and a subset of tumor cell lines. Several hypotheses have been proposed, including differences in the cellular uptake or metabolism of these drugs between sensitive and resistant cells. However, an elegant study recently showed that differences in the permeability of cells to fumagillin and its analogs cannot explain their inhibitory activity against endothelial and cancer cells [27]. It has also been suggested that the cell-type dependent growth inhibition caused by fumagillin may be mediated by unknown proteins directly regulated by MetAP2. The substrates of MetAPs directly involved in the antiproliferative response have not yet been identified. Finally, it has also been suggested that MetAP2 makes a larger contribution than MetAP1 to total methionine aminopeptidase activity within the cell. This possibility has been investigated for more than two decades, but no systematic analysis has been carried out and we therefore still lack a clear view of the respective contributions of MetAP1 and MetAP2 to the process of inhibition *in vivo*, at the proteome level.

Here, we carried out a comprehensive proteomic analysis with an array of cell lines, to investigate the NME *in vivo* and the molecular consequences of MetAP2 inhibition in mammalian cells. We confirmed the selectivity of the inhibitory effects of fumagillin on endothelial cells and several new sets of tumor lines. Large-scale N-terminal proteomic characterization in cell lines responsive and unresponsive to fumagillin treatment highlighted the *in vivo* requirement of both MetAPs for M[VT]X-targets and, potentially, for lower-level MGX targets. Interestingly, glutathione redox homeostasis was altered by MetAP2 inhibition in fumagillin-sensitive cells, but not in fumagillin-insensitive cells. Moreover, the identification of the MetAP2 protein and its quantification by targeted selected reaction monitoring (SRM) mass spectrometry (MS) revealed that the protein accumulated at extremely low levels, but that these levels were slightly higher in fumagillin-insensitive than in fumagillin-sensitive cells. Consistent with this finding, transcripts analysis showed that MetAP levels were strongly correlated with the inhibitory activity of fumagillin in cells, particularly in terms of MetAP1 accumulation within the cell. We suggest that MetAP1 expression could be routinely checked in several types of tumor, as a prognostic marker for predicting the efficacy of any treatments targeting MetAP2 activity and for the fine-tuning of therapeutic strategies.

## RESULTS

### Cell-specific MetAP2 inhibition phenotype

**Figure 1:**
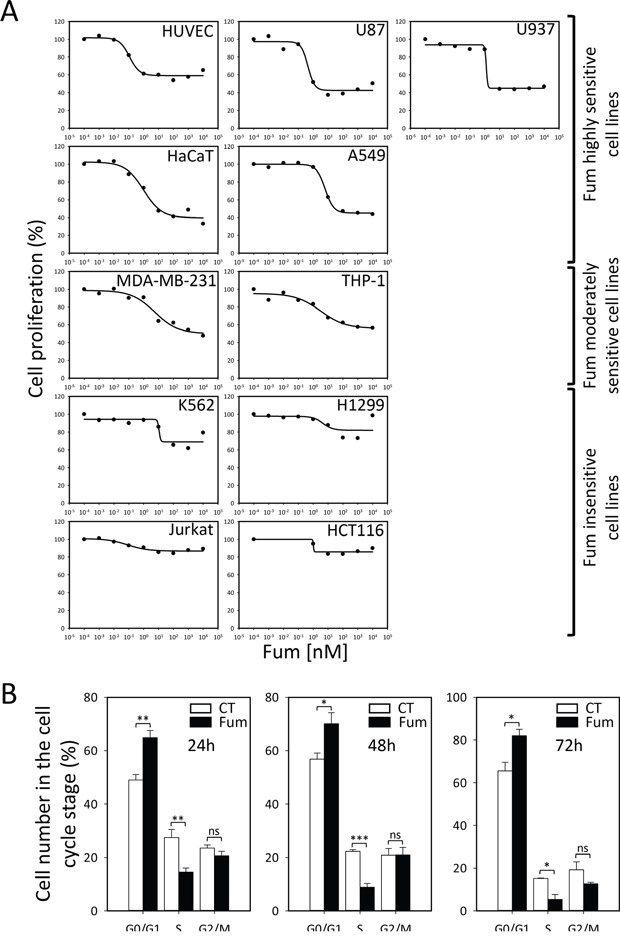
Selective cytostatic effect of MetAP2 inhibition **A.** Analysis of cell proliferation in the presence of various concentrations of fumagillin, for eleven mammalian cell lines. Cells were cultured in presence of the drug for 72 h and their proliferation was then assessed. Cell lines are classified according to their sensitivity to fumagillin. HUVEC, U87, U937, HaCaT and A549 cells were sensitive to fumagillin. MDA-MB-231 and THP-1 cells were considered to be moderately sensitive to fumagillin. K562, H1299, Jurkat and HCT116 cells were insensitive to fumagillin. **B.** Cell-cycle analysis of HUVEC treated with 5 nM fumagillin for 24, 48 or 72 h (Fum, black bars) or mock-treated (CT, white bars). Cell accumulation at the various steps in the cell cycle is shown. Error bars represent the standard error of the mean, n=3-5, *p*-Value from two-sided t-test are represented as described in Methods section. Fum, fumagillin.

We set out to determine the precise selectivity of the MetAP2 inhibition phenotype in large panoply of cell lines. To this end we used a very-well characterized molecule, fumagillin, which has been shown to have potent anti-cellular proliferation activity on endothelial cell lines at very low concentration and specifically inhibits MetAP2 by covalent binding the active pocket of the enzyme [28]. We performed cell growth assays with various mammalian primary, immortalized or tumor cell lines, including endothelial, non-tumor and tumor-derived lines with and without fumagillin treatment, to determine the precise selectivity of the MetAP2 inhibition phenotype. The cell lines analyzed were either insensitive or displayed cytostatic behavior rather than cytotoxicity (Figure 1A and 1B). A strong cytostatic effect was observed in HUVEC, the first cell line shown to be sensitive to fumagillin. In these cells, a 40% decrease in proliferation was observed with nanomolar concentrations of fumagillin, consistent with previous findings (Figure 1A) [15, 29], validating our assay. We classified the lines into three clusters on the basis of fumagillin sensitivity (Figure 1A). The lines highly sensitive to fumagillin included HUVEC, U87, U937, A549 and HaCaT (Figure 1A), with sensitivity values in the range of the reference HUVEC line (Figure 1B). Interestingly, several of these cell lines are cancer cell lines and one is a non-endothelial, non-tumor line (HaCaT). This finding was unexpected because previous studies have suggested that non-endothelial, non-tumor cells are not affected by fumagillin [29]. Two other cell lines (THP-1, from a patient with acute monocytic leukemia, and MDA-MB-231, from a patient with breast cancer) were found to be less sensitive to fumagillin than the cell lines listed above (Figure 1A). By contrast, H1299, a p53-deficient cancer cell line (IARC database), was found to be insensitive to fumagillin. This result is consistent with previous findings suggesting a direct link between the p53 pathway and MetAP2 fumagillin inhibition [8, 30]. However, disagreements remain concerning the underlying mechanism, because the sensitivity of certain tumor cell lines does not appear to be correlated with either p53 status or MetAP2 inhibition [31], as p53 is mutated in over 50% of human tumors [32] displaying different degrees of sensitivity to fumagillin. The Jurkat (mutated p53, IARC database), HCT116 (WT p53, IARC database) and K562 (WT p53, IARC database) cell lines, for example, were all found to be almost completely insensitive to fumagillin (Figure 1A).

As MetAP2 inhibition leads to cell growth arrest, we monitored the different phases of the cell cycle in HUVEC, in the presence and absence of fumagillin. Between 24 h and 72 h, the treated HUVEC were arrested in the G0/G1 phase of the cell cycle (Figure 1B). These results are consistent with the first studies carried out with the fumagillin analog AGM-1470 [33].

Clinical trials with the fumagillin analog TNP-470 revealed that this molecule had a short half-life in the blood (~7 min), possibly due to its rapid metabolism by epoxide hydrolase [34]. In our experiments, we ruled out this possibility by comparing cells from an insensitive line (HCT116) treated with fumagillin once or three times over a 72-hour period. The replacement of the medium with fresh fumagillin-containing medium at 24-hour intervals over the 72-hour period decreased growth rates by only an additional 10%, indicating that metabolism made no significant contribution to the lack of response in these cells (Supplementary Figure 1). We reproduced the effect of fumagillin on the HUVEC reference line, validating our assays, and we then sorted cell lines according to their fumagillin sensitivity. A large range of phenotypes, extending from fumagillin insensitivity to a strong cytostatic effect, was observed.

### Large-scale N-termini characterization highlights the *in vivo* requirement of the full set of active MetAPs for selected targets in all cell lines tested

Here, we addressed a long standing conundrum regarding the *in vivo* substrate specificity of MetAP1 and MetAP2 and the cellular impact of MetAP2 inhibition in sensitive and insensitive cell lines. MetAPs target the N-termini of newly synthesized proteins. We therefore investigated the status of the protein N-termini (the N-terminome), by characterizing the protein N-termini and their modifications by strong cation exchange (SCX) N-terminus enrichment followed by MS/MS analysis (SILProNAQ, [35]), in two sensitive lines and two insensitive lines before and after fumagillin treatment (see Supplementary Figure 2 for the mass spectrometry pipeline). This represents the largest comparative and comprehensive study of the impact of MetAP2 inhibition on the proteome and N-terminome of different lines. We characterized more than 6000 N-termini in total, and up to 1000 different N-terminal peptides per cell line and identified proteins, accordingly. Using this data, we first investigated the specific proteomes of the different cell lines (Supplementary Figure 3A). We reasoned that if one or a few key MetAP2 substrates were the primary cause of the sensitivity to fumagillin, then all sensitive cell lines would have the same specific MetAP2 substrates, which would be strongly affected by fumagillin treatment. In untreated conditions, about half of the proteins identified in one cell line were also retrieved in the other three lines, revealing a high level of proteome similarity between cell lines (Supplementary Figure 3B inset and Supplementary Table 1). Application of the same analysis to the proteins identified after fumagillin treatment resulted in the same overall protein distribution, with about 50% of identified proteins common to all cell lines (Supplementary Figure 3C, inset). Only 24% of the uniquely identified proteins were retrieved from fumagillin-treated insensitive lines (36% in the corresponding control conditions, Supplementary Figure 3B), whereas the number of proteins found only in sensitive lines following fumagillin treatment was unexpectedly found to be greater than that in the corresponding control conditions (30% *vs.* 16%, Supplementary Figure 3B and 3C). This effect could be caused by a difference in an overall protein stability as consequence of fumagillin treatment and reflect a somehow cells sensitivity to fumagillin treatment in general. We thus carried out a direct comparison of the effect of fumagillin treatment on the distribution of identified proteins per cell line. Most of the proteins identified were common to the two sets of conditions (with and without fumagillin) even in highly sensitive lines. A higher degree of proteome similarity was observed between treated and control cells than between different lines (Supplementary Figure 4).

We further investigated the effect of fumagillin on proteomes, by classifying the proteins for each cell line and set of conditions according to their N-terminal status, focusing on the removal or retention of the iMet (e.g. in latin iMet+ indicates totally cleaved, iMet+/− partially cleaved and iMet- retained). MetAP2 inhibition did not change the number of identified proteins common to cell lines for the iMet- and iMet+ classes (Supplementary Table 1). However, for the iMet+/− class, fumagillin treatment resulted in a larger number of shared identified proteins, regardless of cell sensitivity (Supplementary Figure 5). Of note, the enlargement of iMet+/− class in all fumagillin treated cells confirmed the ability of the drug to be addressed into all analyzed cells and to affect MetAP2 activity.

**Figure 2:**
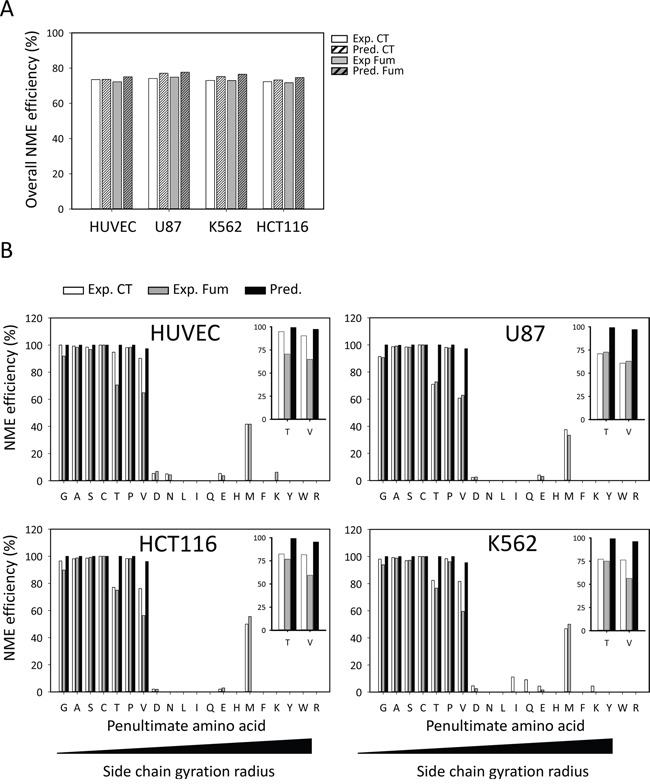
MetAP2 inhibition induces iMet retention of inefficient MetAP substrates Large-scale mass spectrometry analysis of NME efficiency with various cell lines. **A.** Overall NME efficiency is shown for the HUVEC, U87, K562 and HCT116 lines. For each experiment, Uniprot protein accessions were run through the NME predictor (Termi*N*ator, [55]) for comparison. The results are classified by control (CT) and fumagillin-treated samples (Fum). **B.** For each cell line, N-terminal peptides were clustered on the basis of their second amino acids, as represented by the one-letter code, on the *x* axis. For each class, NME efficiency was calculated as the percentage of N-terminal peptides with a cleaved iMet. Data are plotted as a percentage of the total number of peptides identified for the class concerned. Each graph includes results for a control sample (white bars), a fumagillin-treated sample (gray bars) and the results of Termi*N*ator prediction based on the data for control samples. For each line a close up view is given for V and T classes for clarity.

**Figure 3:**
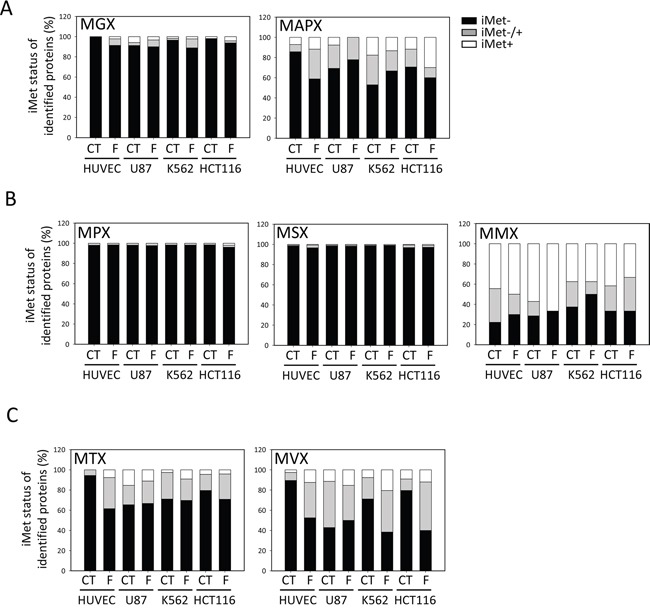
M[T/V]X substrates require the full set of active MetAPs for iMet cleavage For each cell line and set of treatment conditions, the efficiency of NME for proteins with specific N-terminal sequences is analyzed further. The proportion of proteins without the iMet is shown in black, the white bar shows the proportion of proteins with a retained iMet and the gray bar represents proteins with partial cleavage of the iMet residue. **A.** MGX and MAPX proteins, **B.** MPX, MSX and MMX classes and **C.** MTX and MVX classes.

We finally compared our N-termini dataset, enlightening the *in vivo* substrate specificity of MetAP1 and MetAP2, with the predictions obtained with Termi*N*ator3, one of the most reliable predictors of N-terminal protein modifications, including NME (https://bioweb.i2bc.paris-saclay.fr/terminator3/). The experimental data were found overall to be well correlated with the predictions for NME efficiency (Figure 2A). We then assessed the NME efficiency of N-termini according to their second amino acid (*i.e*., the amino acid immediately following the iMet) [13, 36]. Similar results were obtained for all cell lines, showing that cleavage efficiency depended on the gyration radius of the amino-acid side chain at the second position and that cleavage efficiency was high for M[A/G/C/P/S] N-termini (Figure 2B), but low for proteins beginning with M[V/T]. Cleavage efficiency was also low for some proteins harboring a second G residue, despite the prediction of highly efficient cleavage for this amino acid (Figure 2B). There are two non-mutually exclusive interpretations of this small discrepancy between observation and prediction: i) the software may overestimate the cleavage efficiency of these substrates and/or ii) the explored cells may have inadequate levels of MetAP or protease activities. Regardless of fumagillin sensitivity, G cleavage efficiency was found to be low in all four lines (Figure 3A). In our global analysis, a few cleaved M[G] proteins retained their iMet upon fumagillin treatment (Figure 3A). Interestingly, proteins beginning with MAPX were particularly responsive to fumagillin, with an increase in the number of unprocessed proteins retrieved upon MetAP2 inhibition (Figure 3A). This result is entirely consistent with previous reports of a clear decrease *in vitro*, by at least one order of magnitude, for substrates with a proline residue in the P2′ position, even if the second amino acid generally results in highly efficient NME [13]. Other classes of substrates, such as M[M] protein N-termini, displayed abnormal behavior. These exceptional cases may be the result of alternative starts as previously reported [37–39]. Nonetheless, fumagillin treatment had no further effect on cleavage efficiency of all these substrates (Figure 3B). Finally, M[V/T] protein N-termini analysis in the context of fumagillin treatment showed that MetAP2 inhibition led to the production of a larger number of N-terminal peptides displaying partial cleavage rather than complete retention of the iMet (Figure 2B inset, Figure 3C and Supplementary Figure 5).

We then analyzed the second amino acid of the N-terminal peptides identified for an absence of iMet cleavage, to determine whether fumagillin treatment led to a bias in protein identification. This would be the case, for example, if proteins with specific N-termini were degraded and therefore not well retrieved following fumagillin treatment. Such effect would modify the frequencies of the amino acids, in the identified proteins. Fumagillin treatment did not modify the second amino acid frequency of the N-termini in any of the cell lines and these frequencies were very similar in all four lines (Supplementary Figure 6), suggesting an absence of bias in N-termini retrieval. With the experimental design used, we were able to identify, in the different cell N-terminomes, other important N-terminal protein modifications, such as N-α-acetylation and, to a lesser extent, N-myristoylation, which changes would potentially account for differences in fumagillin sensitivity. No changes in overall N-α-acetylation or N-myristoylation were observed (Supplementary Figure 7A and 7C), including when looking specifically at NATA substrates which require N-terminal processing and therefore could be directly affected in case of NME defect (Supplementary Figure 7B). This excludes the possibility that differences in fumagillin sensitivity is due to specific alteration of NME downstream N-terminal modifications. Concerning N-myristoylation, for exemple, we identified three to twelve myristoylated proteins per line, with a considerable overlap between the four cell lines (Supplementary Figure 7C). However, we were able to identify only one protein displaying a clear N-myristoylation defect on MetAP2 inhibition. This protein was the proto-oncogene protein SRC, which was either myristoylated or N-α-acetylated in HCT116 cells (which are insensitive to fumagillin) in control conditions but was retrieved exclusively as the N-α-acetylated form after fumagillin treatment.

To conclude, our proteomic analyses of each cell line with and without MetAP2 inhibition indicated that proteomic differences could not account for the selectivity of fumagillin sensitivity and that fumagillin did not induce major proteome remodeling, regardless of fumagillin sensitivity. On the other hand, our comprehensive proteomic analysis of protein N-termini demonstrated for the first time that MetAP2 inhibition resulted in an increase of unprocessed N-termini which were quantitatively and qualitatively very similar between fumagillin sensitive and insensitive lines. This proteomic analysis tends to exclude that the sensitivity of cell lines to fumagillin is due to i) an already different cleavage extend between sensitive and insensitive lines or ii) one specific MetAP2 substrate or iii) changes of the downstream N-terminal modifications. Therefore, the sensitivity might be driven by others factors than uniquely the specific MetAP2 substrates that are not processed by MetAP1 in sensitive cells. For instance, GAPDH, one of the specific MetAP2 substrates identified in previous studies, was retrieved in all cell lines tested and displayed similar N-terminus modifications upon fumagillin treatment in both sensitive and insensitive cell lines (Supplementary Table 1 and Supplementary Figure 5). We found that M[V/T] substrates and a few other substrates, such as MAPX proteins, required the full set of active MetAPs in all cell lines tested, suggesting a possible effect of low levels of general MetAP activity or availability within the cell. Consistent with this hypothesis, the identification of several types of N-terminus for a given protein, including some displaying partial iMet cleavage, suggested that the requirement for MetAP2 was probably due to a quantitative effect of the decrease in MetAP2 levels and/or overall NME capacity in the sensitive cell lines.

### MetAP2 protein levels are higher in fumagillin unresponsive cell lines than in sensitive cell lines

**Figure 4:**
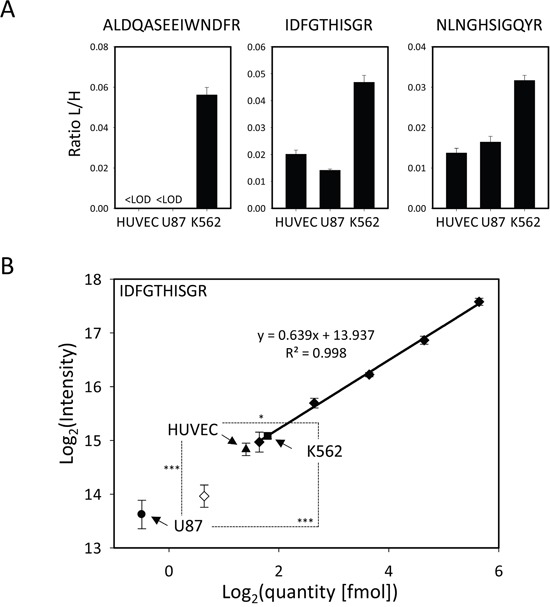
MetAP1 and MetAP2 quantification: both proteins are more abundant in the insensitive cell line K562 **A.** Relative quantification of MetAP1 and MetAP2. The ratios of the summed areas of all transitions of the light over the heavy-labelled peptide (L/H) are shown for sample-preparation quadruplicates for the 6 targeted peptides in three different cell types. **B.** Calibration curve for the absolute quantification of MetAP2 by monitoring the IDFGTHISGR peptide. The calibration curve was done using the points where CVs were lower than 15% and the accuracy was between 80-120% (full diamonds) the lowest point being the limit of quantification (LOQ). The points not meeting these criteria were discarded (empty diamonds). The MetAP2 protein is present in a very low abundance in all three cell lines K562 (box), HUVEC (triangle, below LOQ) and U87 (disk, below LOQ). Both independent studies showed the same trend: MetAP2 is more abundant in fumagillin-insensitive cell lines. The statistical significance of the differences in MetAP2 quantity is given by a two-sample t-test between each line. The *p*-Value is given as described in the Methods section.

We used immunodetection methods to rule out the possibility that cellular levels of MetAP1 and MetAP2 proteins were the primary cause of differences in sensitivity between cell lines. Unfortunately, in our conditions, no MetAP1 or MetAP2 was detected in most of our cell lines, including the most sensitive ones, even if large amounts of protein were used. These proteins therefore seem to be present at only very low levels in the cell lines analyzed. We used a targeted mass spectrometry technique (selected reaction monitoring, SRM) to confirm this finding and to identify and quantify, with high levels of sensitivity and precision, the MetAP1 and MetAP2 proteins in three cell lines: the most sensitive cell line from cancer tissue (U87), the sensitive reference endothelial cell line (HUVEC) and a non-sensitive cell line (K562). Three peptides found to be unique and specific to each MetAP were chosen and subjected to relative quantification. The sensitive lines were found to contain much less MetAP2 and MetAP1 than the insensitive cell line (Figure 4A, Supplementary Figure 8, 9 and Supplementary Table 2). We validated this result by carrying out an absolute quantification experiment with one highly purified and precisely quantified peptide. This experiment confirmed that MetAP2 levels were extremely low in all three cell lines (350 amol MetAP2 protein per μg total protein in K562, whereas U87 and HUVEC had abundances below the limit of quantification, which was 310 amol per μg total protein), and confirmed the finding that sensitive cell lines tended to have lower MetAP2 levels than insensitive lines (Figure 4B, Supplementary Table 2).

### Correlation of MetAP2 inhibition phenotype and MetAP mRNA levels

**Figure 5:**
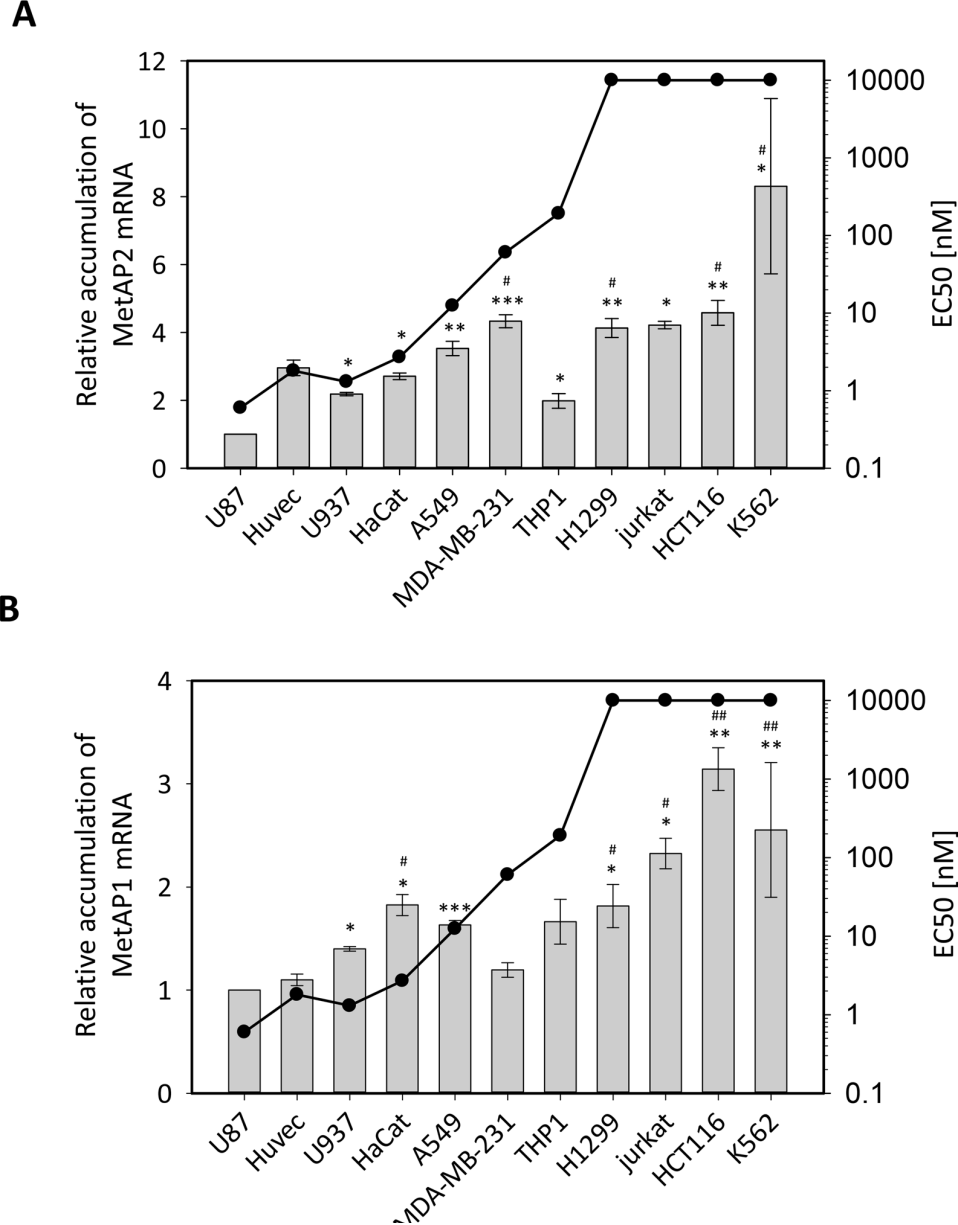
Cell-specific accumulation of MetAP mRNAs correlates with fumagillin sensitivity The eleven cell lines were cultured according the standard procedure. Total RNAs were extracted and the MetAP2 **A.** and MetAP1 **B.** mRNAs were quantified. MetAP mRNAs were quantified with two reference genes from the ten tested. Results are normalized with respect to data for the U87 cell line and are expressed as a fold-change. The data presented are the means from at least three independent experiments. Half maximal effective concentration (EC_50_) values were obtained from the dose-response curves shown in Figure 1A. Error bars represent the standard error of the mean of 3 to 4 experiments. The statistical significance (two-sided t-test) of the differences when comparing U87 (*) and HUVEC (#) lines to the others is displayed. *p*-Value is shown as follow: * or # *p*Val<005, ** or ## *p*Val<0.01, *** *p*Val<0

The low levels of MetAP2 protein observed in sensitive and insensitive cell lines led us to study in detail the mRNA levels for both MetAPs in all the cell lines with different degrees of sensitivity to fumagillin studied. We carried out RT-qPCR, including multiple reference genes, to ensure that measurements were both robust and highly precise. We first focused on MetAP2 mRNA levels, which were found to be higher in the K562 cell line than in any of the other cell lines tested (Figure 5A). By contrast, the U87 cell lines had the lowest levels of MetAP2 mRNA (Figure 5A), consistent with the SRM analysis (Figure 4). These data are also consistent with the fumagillin sensitivities of these cell lines, with U87 being among the most sensitive lines tested, and K562 the least sensitive (Figure 5 and Figure 1A). For the other cell lines tested, MetAP2 levels and cell responsiveness to fumagillin were found to be correlated overall, with a high degree of confidence, but this link was not straightforward when selected cell lines were compared (Figure 5A). For instance, although HUVEC was more sensitive to fumagillin than THP1, its MetAP2 levels were significantly higher than those of THP1 (Figure 5A), suggesting that, for cells with levels of MetAP2 intermediate between those of U87 and K562, other factors contribute to the specific fumagillin sensitivity of the cells. In the cytoplasm of eukaryotic cells, the iMet may be excised by MetAP2 or MetAP1, these two enzymes having the same substrate specificity *in vivo*, but including only a few proteins beginning with M[V/T] (see the above proteomic analysis and [13, 36]). Given that, unlike MetAP2, MetAP1 is not a target of fumagillin, we assumed that differences in the sensitivity of different cell lines might depend not only on MetAP2 levels, but also on the total MetAP activity mediated by both MetAP2 and MetAP1 within the cell. We tested this hypothesis, by determining MetAP1 mRNA levels in all the cell lines tested (Figure 5B). These levels differed by a factor of up to three, indicating that MetAP1 was also regulated (Figure 5B). This result was unexpected, given that MetAP1 is generally considered to be a housekeeping protein. Moreover, as for MetAP2, we found that MetAP1 mRNA levels were very low in U87 and significantly higher in insensitive cell lines, such as H1299, Jurkat, HCT116 and K562 (Figure 5B), confirming the observed trend in the MetAP1 protein relative quantification experiments (Figure 4A, Supplementary Figure 8 and Supplementary Table 2). Interestingly, total MetAP mRNA levels were low in HUVECs, perfectly consistent with the high fumagillin sensitivity of this cell line (Figure 5B). Our results strongly suggest that once all the MetAP2 protein molecules within the cell have been inhibited by covalent binding to fumagillin, MetAP1 is the only remaining MetAP active within the cell. It therefore becomes solely responsible for ensuring the excision of the iMet residue from all the proteins of the cellular proteome for which this cleavage is essential. MetAP1 level is therefore a key factor influencing cell sensitivity to fumagillin.

### Redox homeostasis is altered in cells sensitive to MetAP2 inhibition

**Figure 6:**
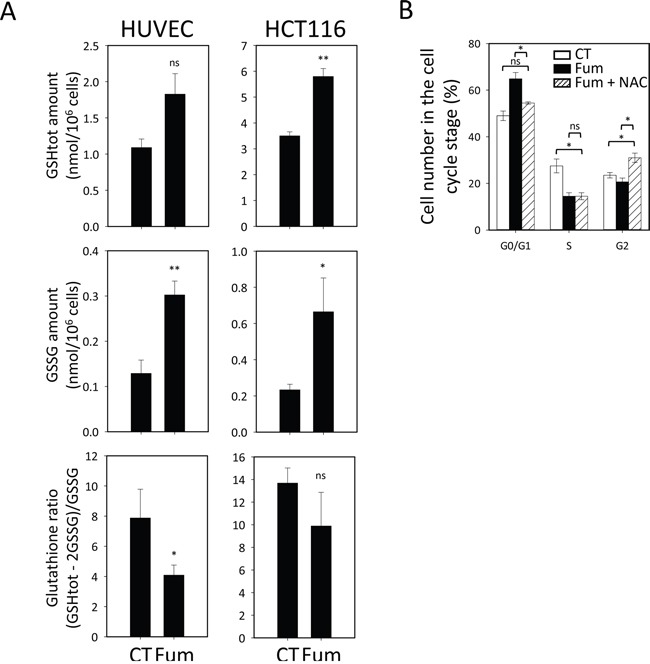
MetAP2 inhibition selectively alters glutathione redox state in fumagillin sensitive cells **A.** HUVEC and HCT116 cells were cultured without (CT) or with 5 nM and 1 μM fumagillin (Fum) for respectively 72 h before glutathione quantification. The amount shown for glutathione corresponds to the total pool of glutathione (top panels); GSSG is the oxidized form of glutathione (middle panels) and the glutathione ratio is the ratio of reduced to oxidized glutathione (bottom panels). Error bars represent the standard error of the mean. **B.** Cell cycle analysis for HUVECs left untreated (CT) or treated (Fum) with 5 nM fumagillin for 24, 48 or 72 h or with fumagillin and N-acetylcysteine (NAC) for 72 h. Cell accumulation at the various steps of the cell cycle is shown. Error bars represent the standard error of the mean. *p*-Value from two-sided t-test are represented as described in Methods section.

The inhibition of NME has been shown to affect glutathione homeostasis in Archaea, *Saccharomyces cerevisiae* and *Arabidopsis thaliana* [7]. Glutathione is the main non-protein thiol in the cell and it controls overall cell redox homeostasis [40]. Cellular glutathione status is governed by the ratio of reduced to oxidized glutathione, the hallmark of cellular redox homeostasis status, and ensures the protection of cell components from harmful reactive oxygen species. In our proteomic study (see above), we identified a number of proteins that were controlled or affected by glutathione and generally involved in redox regulation in conditions of MetAP2 inhibition. One of these proteins was TXNL1 (Supplementary Table 1) already described to be affected at its N-terminus by MetAP2 inhibition. Such changes were observed only in HUVEC cells treated with fumagillin in our experiments. To better investigate the cross-talk between the cytosolic NME process and glutathione homeostasis we determined total glutathione content in both sensitive (HUVEC) and insensitive (HCT116) lines, in the presence and absence of fumagillin treatment. Total glutathione content was significantly higher in lines insensitive to fumagillin than in sensitive lines (Figure 6A), due to the large amounts of both oxidized and reduced glutathione (Figure 6A). Following the treatment of HUVECs with fumagillin, we observed an increase in the total glutathione pool (Figure 6A). The amount of the oxidized form of glutathione (GSSG) was more than doubled in treated HUVECs (Figure 6A), resulting in decreases in the cellular glutathione redox ratio and reducing power (Figure 6A). Unlike the sensitive cell line, the insensitive HCT116 line displayed no change to the glutathione redox ratio following treatment with fumagillin, despite slight increases in the total glutathione pool and GSSG levels (Figure 6A). Indeed, the high total glutathione level of HCT116 cells was sufficient to buffer the increase in GSSG levels induced by fumagillin treatment and to prevent any damage to the redox potential of the glutathione system.

It has already been reported that cell-death induced by TNP-470 in B16F10 murine melanoma cell lines can be prevented by the presence of N-acetylcysteine or cysteine in the medium and that this effect is not induced by direct interaction of the drug with these antioxidants [41]. We investigated the importance of NME for the control of cellular homeostasis, by performing experiments to determine whether the low levels of proliferation resulting from cytoplasmic NME inhibition could be corrected by adding N-acetylcysteine to the culture medium. The presence of N-acetylcysteine in the medium partially prevented the inhibition on proliferation of sensitive cell lines and particularly the fumagillin-induced arrest of HUVECs in G0/G1 (Figure 6B), highlighting the importance of the cytoplasmic NME pathway for the control of the GSH redox pathway to ensure correct cell progression. Altogether, we found that MetAP2 inhibition induces a redox unbalance in cell which can be handled by fumagillin insensitive line. On the contrary, sensitive cells are affected by such effect and are hampered in their cell cycle progression.

## DISCUSSION

In this study, we employed fumagillin as a tool to study cellular and proteome consequences induced by such compound to understand the basis of the selectivity of the anti-cellular activity of MetAP2 inhibition and to lastly shed light on the substrate specificities of the two cytosolic MetAP enzymes *in vivo*. Fumagillin has been shown to target MetAP2 in a highly specific manner, inducing an anti-tumor effect [20, 21, 28, 42]. Moreover ectopic MetAP2 expression increases fumagillin resistance, indicating that the cytostatic phenotype is related to MetAP2 inhibition [43]. This result was surprising given that MetAP2 inhibition in other eukaryotic systems does not lead to a growth defect and all eukaryotes possess both MetAP1 and MetAP2 [7, 10, 14]. It has also recently been shown that cell permeability cannot account for phenotype selectivity [27]. Consistent with these findings, our data suggest that fumagillin is not rapidly metabolized. Therefore the cellular basis of how MetAP2 inhibition could result in different phenotype was still unclear despite it has been basically assumed, with no substantive evidence, that there must be specific substrates that are not processed by MetAP1 and that the failure to remove the iMet from one or more of these MetAP2 specific protein substrates renders them inactive in a critical phase of cell cycle progression.

**Figure 7:**
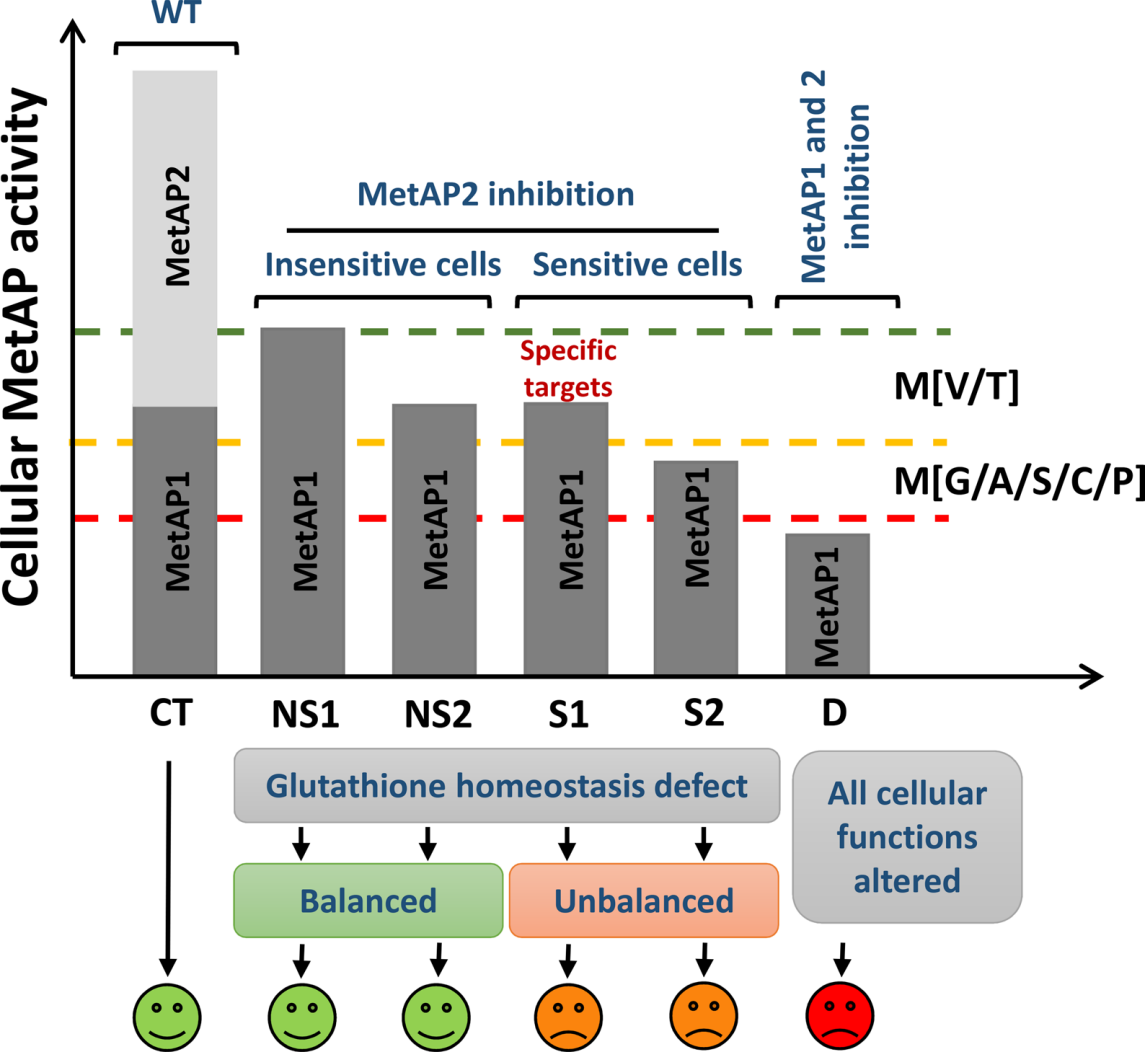
Understanding the cell selectivity of MetAP2 inhibition phenotype The *y*-axis shows total cellular MetAP activity. In control conditions (CT), MetAP1 and MetAP2 are active and catalyze the complete cleavage of MetAP substrates. Upon MetAP2 inhibition, only MetAP1 can process MetAP substrates. Due to their very similar substrate specificities, MetAP1 can cleave most of the MetAP2 substrates. Thus, provided that MetAP1 is not limiting, substrates are processed (NS1). However, if MetAP1 becomes limiting within the cell, then either the unprocessed substrates are not important for cell growth (NS2) or some specific substrates may become less stable, with effects on growth (S1). In such conditions, the first proteins affected are the poorest MetAP substrates (M[V/T]; NS2 + S1). These proteins may be involved in glutathione redox homeostasis and control important aspects of cell life. If smaller amounts of MetAP1 are present in the cell, then a larger number of substrates are likely to be affected, including abundant proteins beginning with MG (S2). Regardless of cell sensitivity, MetAP2 inhibition impairs glutathione redox homeostasis. This defect is overcome or compensated in insensitive lines but not in sensitive lines, resulting in a slow-growth phenotype. If MetAP activity decreases further, too many substrates are likely to be affected, with deleterious effects on cell function, eventually leading to cell death (D).

The extensive characterization carried out in this study provides clues to the basis of cell sensitivity to MetAP2 inhibition. Here, we performed the largest N-terminomic analysis of different cell lines with and without MetAP2 inhibition treatment to date, in which we compared the effect of the drug on protein N-termini in sensitive and insensitive cell lines. MetAP2 inhibition was found to have no effect on the proteome or the nature of the N-termini of the proteins identified, regardless of cell sensitivity to fumagillin. MetAP2 inhibition mostly resulted in a decrease in cleavage efficiency for M[V/T] substrates, and also, to a less extent, for M[G] substrates. This result is consistent with previous reports showing that cyclophilin A, glyceraldehyde-3-phosphate dehydrogenase, a thioredoxin [44], an SH3-binding protein, the elongation factor 2, a thioredoxin-like protein and the hemoglobin α-chain [36, 44, 45] retain their iMet residues when MetAP2 is inhibited. All these proteins have a V as the second amino acid of the N-terminal region. This led to suggestions that MetAP2 might cleave some substrates more efficiently than MetAP1. However, these differences may be modified by other factors *in vivo*, such as the relative enzyme-substrate concentration, as suggested but not experimentally demonstrated in a previous study [36]. Indeed, biochemical studies have demonstrated that M[V/T] proteins are inefficient substrates of both MetAP1 and MetAP2 [13]. We also report here that MetAP2 inhibition decreases NME efficiency mostly for M[V/T] substrates and for several M[G] proteins (Figure 3). This group of substrates therefore requires the full set of active MetAPs, and this is particularly true for abundant proteins, such as glyceraldehyde-3-phosphate dehydrogenase which belongs to the 100 most abundant proteins [46]. Indeed, this pattern perfectly mimics the effects of an overall decrease in total cellular MetAP capacity rather than selective changes in MetAP substrate specificity, which would lead to the least efficient substrates being the most affected. When MetAP2 is not functional, MetAP1 activity is limiting and poor substrates, such as M[V/T], are less efficiently cleaved (Figure 3). In other words, once MetAP2 has been inhibited, the amount of MetAP1 in the cell determines the residual cellular NME activity (Figure 7). Interestingly, specific inhibition of MetAP1 also leads to a strong effect on HUVEC suggesting that the same set of substrates are affected regardless which MetAP is inhibited [9, 47]. Moreover, it has been shown using specific antibodies that the protein 14-3-3γ which has a valine as a second amino acid is a poor substrate of both MetAPs and inhibition of one or the other MetAP leads to an increase of unprocessed 14-3-3γ supporting our findings [47]. Unfortunately, we could identify the 14-3-3γ N-terminal peptide only for the U87 cell line in both control and treated cells. In both situations we characterized only unprocessed iMet indicating that this is a really poor MetAPs substrate and that only a high quantity of both MetAPs can process at least partially such substrates. Despite the highly similar substrate specificities of the two enzymes, specific MetAP2 inhibition by fumagillin blocks the cell cycle of a subset of cells, including endothelial cells. However, fumagillin had similar effects on the proteomes and protein N-termini of fumagillin-sensitive and -insensitive cells. Consequently, the sensitivity phenotype cannot be accounted for by a qualitative component of iMet excision, such as specificity of MetAP substrates, as suggested in the past. Instead, it seems to depend on quantitative aspects of the decrease in cleavage rates. MetAP2 protein levels were extremely low in all cells tested, particularly the most sensitive lines, in which strong regulation of the corresponding mRNA was also observed. Moreover, by precise mRNA quantification, we were able, for the first time, to demonstrate the clear regulation of MetAP1 mRNA levels, with three-fold differences between the cell lines tested, highlighting a new role for MetAP1 in cell physiology in the context of MetAP2 inhibition. It has been suggested that MetAP1 plays an important role in the G_2_/M phase transition and interfering with its activity led to cell cycle arrest [9]. Interestingly, levels of MetAP1 and MetAP2 were clearly correlated with fumagillin-induced cytostasis. Indeed, the MetAP1 mRNA data clearly indicated that the presence of large amounts of MetAP1 protects cells against the effects of fumagillin and allows cell cycle progression.

We discovered that one of the first alterations induced by MetAP2 inhibition was a redox homeostasis defect. Indeed, our redox analyses and proteomic data indicated that GSH redox homeostasis played a role in the phenotype of MetAP2-inhibited cells. In both sensitive and non-sensitive cells the total glutathione pool increased, indicating a clear challenge to the cellular redox system in both situations (Figure 7). However, the glutathione ratio, which represents the redox status of the glutathione in the cell, was decreased by treatment in fumagillin-sensitive cells, but not in insensitive cells (Figure 7). Interestingly, it has also been shown in the higher plant *Arabidopsis thaliana* that decreases in NME levels affect glutathione redox homeostasis. A link between NME inhibition and glutathione has also been established in yeast and Archeae [7]. MetAP2 inhibition has been reported to lead to cell cycle arrest in the G1 phase through the inhibition of Rb protein phosphorylation and CdKi p21 accumulation in endothelial cell lines and in several tumor cells, and both p53 and p21 seem to be required for the antiproliferative effect of TNP-470 [24, 30, 48]. MetAP2 inhibition blocked the cell cycle in G1 phase, with a progressive accumulation of cells at this step. The addition of an antioxidant to sensitive cells in which MetAP2 was inhibited partially overcame this cell cycle arrest. This finding is consistent with the observation that the growth defect induced by NME inhibition in other organisms can be rescued by antioxidant treatment [7, 41]. Thus, MetAP2 inhibition leads to an imbalance in redox homeostasis in fumagillin-sensitive cells, providing a possible explanation for the fumagillin sensitivity phenotype. Another antioxidant pathway, the thioredoxin pathway, was recently shown to play an important role in cancer cells, rendering GSH dispensable during cancer progression [49]. In addition, our study and others identified a thioredoxin protein, TXNL1, as being unprocessed upon MetAP2 inhibition [36, 44, 45]. TXNL1 displays thioredoxin activity. It was suggested that TXNL1 plays a role in the transfer of misfolded nascent chain from the ribosome to the 26S proteasome [50]. Given the cross-talk between the cellular redox state, protein degradation and N-terminal methionine excision discovered in many systems it appears that TXNL1 might be one of the crucial elements at the center of this crossroads. Future studies should clearly include a specific examination of antioxidant systems on MetAP2 inhibition in different cell lines, to assess the potential involvement of the thioredoxin pathway in the insensitivity phenotype. Together, these findings for different model systems suggest that redox homeostasis may play a more general role in NME-compromised cells. Redox impairment has been recognized in the past few years as a specific vulnerability of diverse tumor cells [51]. Targeting the oxygen stress response pathway is considered as a promising strategy for a broad spectrum antitumor effect [52]. Hence, we reasoned that combining MetAP2 targeting treatment with redox-directed therapeutics would lead to more efficient antitumor activity on a wider spectrum of tumor.

In conclusion, MetAP1 level is a key determinant of the MetAP2 inhibition phenotype. Since we showed here that cell sensitivity to fumagillin is correlated with mRNA levels, and with MetAP1 mRNA levels in particular, we therefore suggest that mRNA MetAP1 levels could be routinely checked in several type of tumors and used to predict whether treatment targeting MetAP2 is likely to be effective, thereby guiding the choice of the appropriate treatment. We discovered a clear link between MetAP2 inhibition sensitivity and redox homeostasis which could be utilize for therapeutic strategy improvement. We think that determinations of MetAP1 levels and of imbalances in redox homeostasis are potentially useful new tools that may foster the development of new personalized anticancer strategies.

## MATERIALS AND METHODS

### Chemicals

Modified porcine trypsin was obtained from Promega (Madison, WI); synthetic stable isotope-labeled peptides with C-terminal ^15^N- and ^13^C-labeled arginine residues were purchased in crude (PEPotec SRM Peptides) and purified form (AQUA peptides) for LC-SRM assays from Thermo Fisher Scientific (Ulm, Germany). All other reagents and chemicals were purchased from Sigma Aldrich (St. Louis, MO).

### Cell culture and treatments

Cancer cell lines were obtained from the American Type Culture Collection (ATCC; Rockville, MD, USA). Human umbilical vein endothelial cells (HUVEC) were obtained from Clonetics (Lonza; Walkersville, MD, USA) and were cultured as described in the Supplementary Information and according to the supplier's instructions. For the proteomics and transcriptomics analysis 6.2×10^6^ sensitive and insensitive cells were plated in petri dishes and cultured as described in the Supplementary Information. After 24 h medium is removed and changed with fresh medium containing or not 5μM fumagillin. Cells were cultured for additional 24h before collecting them for further analysis. Cell viability was assessed with the Promega CellTiter-Blue™ reagent, according to the manufacturer's instructions. After 24 h of culture, the cell medium was supplemented with 50 μL of the test compound dissolved in DMSO (<0.1% in each preparation). After incubation for 72 h, we added 20 μL resazurin and incubated the mixture for a further 2 h before recording fluorescence (λex=560 nm, λem=590 nm) in a Victor microtiter plate fluorimeter (Perkin Elmer, USA). Experiments were performed in triplicate.

### Experimental design and statistical rationale

In this studies we compared fumagillin-sensitive and –insensitive lines. Especially for proteomic analysis, to have a broader significance, two cell lines of each class were analyzed. The selection of these lines is described in the first result section. The quality of the methodology used allowed us to characterize more than 1000 unique N-terminal peptides per cell lines (see proteomic analysis in Supplementary Information) allowing to decipher the *in vivo* substrate specificity of MetAPs in different conditions.

Except otherwise stated, all results were produced from at least 3 independent biological repeats (n) and when the mean is computed the associated standard error of the mean is given. The statistical significance is assessed by running a two-sided t-test (GraphPad Software) and the associated *p*-Value is shown as follow: * *p*Val<005, ** *p*Val<0.01, *** *p*Val<0.001, ns non-significant. For mass spectrometry analysis, the overall pipeline is given in Supplementary Figure 2 for clarity and the data processing is described in the mass spectrometry section in Supplementary Information. For MetAP2 and MetAP1 quantification, three peptides were used and multiple injections for each independent repeats. The associated data processing is described in the corresponding section in Supplementary Information. In addition, to assess the quality of the experiment, coefficients of variation are given (Supplementary Figure 9). The transcriptomic analysis was performed with first 10 references genes, the 4 most stables were kept for data normalization as described in corresponding sections.

### MetAP mRNA quantification

Cell pellets were washed with DPBS. RNA was extracted with the NucleoSpin RNA kit (Macherey-Nagel), according to the manufacturer's instructions. The RNA was quantified and its quality was assessed using the Agilent 2100 Bioanalyzer with the eukaryote total RNA 6000 Nano assay. 1 μg of total RNA was reverse-transcribed in a 20 μL final reaction volume using the High Capacity cDNA Reverse Transcription Kit (Life Technologies) with RNase inhibitor according to the manufacturer's instructions. Quantitative PCR for MetAP1, MetAP2 and reference genes was performed using 10 ng cDNA with TaqMan Real-Time PCR assays (Life Technologies). 10 reference genes have been tested and the two most stable genes (18S and PPIA) selected by GeneX software (GeneX Gene Expression Database) were used to normalize the data. The TaqMan Real-Time PCR assays used are: MetAP1-Hs00299385_m1, MetAP2-Hs00199152_m1, 18S-Hs99999901_s1 and PPIA-Hs99999904_m1.

### N-terminal proteomic analysis

Proteomic analyses were performed essentially as previously described [53]. Further details are provided in the Supplementary Information.

### Determination of MetAP2 and MetAP1 proteins accumulation by LC-SRM

Three human cell lines (HUVEC, U87 and K562) were studied. Cells were lysed and proteins were extracted, reduced, alkylated, and precipitated. Proteins were digested with trypsin, in solution, and the resulting tryptic peptides were desalted by SPE and analyzed by microLC-SRM. A first experiment was performed with six crude synthetic peptides specific to MetAP2 and MetAP1, for the relative quantification of MetAP2 in three cell lines. We then carried out a second experiment with one highly purified and precisely quantified AQUA peptide, for the absolute quantification of MetAP2. Further details are provided in the Supplementary Information.

### Glutathione quantification and cell cycle analysis

Glutathione determinations were carried out essentially as described by Rahman *et al*. [54]. Cells were harvested by centrifugation, and the resulting cell pellet was washed with DPBS. The different forms of glutathione were extracted in a buffer containing sulfosalicylic acid to inhibit γ-glutamyl-transferase, by sonication. The enzymatic reaction of the glutathione reductase was used for quantification. Oxidized glutathione was specifically quantified by treating the lysate with 2-vinylpyridine, which inactivates reduced glutathione. Rates of enzymatic activity were recorded and compared with a glutathione standard curve. Cell cycle analysis was performed according to a standard procedure based on propidium iodide staining. Cells were harvested, fixed and stored in ethanol at -20°C until analysis. They were then incubated for 30 minutes with RNase and stained by incubation with 50 μg/mL^−1^ propidium iodide for 15 minutes. Cell cycle profiles were acquired with an FC500 cytometer (Beckman-Coulter).

## SUPPLEMENTARY DATA, FIGURES AND TABLES







## Abbreviations

MetAP: methionine aminopeptidase
M: Methionine
V: Valine
G: Glycine
P: Proline
T: Threonine
C: Cysteine
S: Serine
A: Alanine
X: any amino acid
MYR: myristoylation
NTA: N-terminal acetylation
N/A: not identified
CT: Control
Fum: Fumagillin
iMet: initial Methionine
NME: N-terminal methionine excision
HUVEC: Human umbilical vein endothelial cell
FCS: fetal calf serum
GO: Gene ontology
amol: attomole
SCX: strong cation exchange
RT-PCR: Reverse transcription polymerase chain reaction
GAPDH: glyceraldehyde-3-phosphate dehydrogenase
MS: mass spectrometry
SRM: selected reaction monitoring
EC50: Half maximal effective concentration
LOQ: Limit of Quantification
LOD: Limit of Detection
NAC: N-acetylcysteine
IAA: iodoacetamide
SPE: solid phase extraction.
