# Supplementary material for: MetAP1 and MetAP2 drive cell selectivity for a potent anti-cancer agent in synergy, by controlling glutathione redox state
Source: Oncotarget. 2016 Aug 11;7(39):63306–23. doi: 10.18632/oncotarget.11216 (PMC5325365; doi:10.18632/oncotarget.11216)
Supplement: Supplementary file 1 [file oncotarget-07-63306-s001.pdf]

MetAP1 and MetAP2 drive cell selectivity for a potent anti-cancer agent in synergy, by controlling glutathione redox state

SUPPLEMENTARY DATA

SUPPLEMENTARY MATERIALS AND METHODS

Cell cultures

MDA-MB-231, U87-MG, A549, HaCaT and H1299 cells were grown in Dulbecco's minimal essential medium (DMEM) containing 4.5 g/L glucose supplemented with 10% fetal calf serum (FCS) and 1% glutamine. Human K562, THP-1, Jurkat, U937 and HCT-116 cells were grown in RPMI 1640 containing 10% FCS and 1% glutamine. HUVECs were subcultured after three to six passages and grown to confluence in tissue culture flasks, in endothelial cell growth medium (EGM2) containing growth factors and 2% FCS. All cell lines were maintained at 37°C, under a humidified atmosphere containing 5% CO_2.

N-terminal proteomic analysis

Cell lysates were treated as previously described [1], with protein N-terminal d3-acetylation, cysteine reduction/alkylation, trypsin digestion and SCX fractionation. Fractions eluted from SCX columns with retention times of 3 to 22 min were analyzed as previously described [1] with an Easy Nano-LC II (Thermo Scientific) coupled to a LTQ-Orbitrap™ Velos (Thermo Scientific). SCX fractions were loaded onto an NS-MP-10 pre-column (Nanoseparation, Nieuwkoop, The Netherlands) at a maximum pressure of 220 bars and separated on a Nikkyo Technos capillary column (NTCC-360/100-5-153, Nikkyo Technos Co., Tokyo, Japan). The MS survey scan was acquired by Fourier-Transform scanning 400-2000 Da at 60,000 resolution, with internal calibration. The 20 ions giving the most intense signals were subjected to high CID-MS with an exclusion time of 20 seconds for the selected precursor. MS/MS spectra with parent ion signals of more than 1 count and with a signal-to-noise ratio greater than 1.5 were extracted with Proteome Discoverer (Thermo Scientific, Ver. 1.4) and transferred to Mascot software (Matrix Science, London, UK, Ver. 2.4) for protein identification and the characterization of the co/post-translational modifications by comparison with the *Homo sapiens* reference containing splicing isoforms proteome available from the UniProtKB web site (Release 2014_11, 41988 sequences). The trypsin/P rule, with up to eight missed cleavages was used, with parent and fragment mass tolerances of 10 ppm and 0.6 ppm, respectively. Carbamidomethylcysteine and d3-acetyl on Lys were considered as fixed modifications, whereas Met-oxidation, protein N-terminal myristoylation, acetylation and d3-acetylation, phosphorylation (S/T) and K^+ peptide cationization (D/E) [2] were considered as variable modifications. All data were filtered at a protein false discovery rate of 1% using internal decoy approach.

Accession codes

The mass spectrometry proteomics data have been deposited to the ProteomeXchange Consortium (http://proteomecentral.proteomexchange.org) via the PRIDE partner repository [3], with the dataset identifier PXD002690.

Assessment of MetAP1 and MetAP2 protein accumulation by LC-SRM

Sample preparation

Cells were cultured as previously described in the main text, counted using Vi-cell XR (Beckman Coulter), centrifuged and stored at -80°C. Cell pellets were resuspended in 800 µL of extraction buffer (8 M urea, 2 M thiourea, 1% DTT, protease inhibitors), sonicated and the resulting suspension was centrifuged (5 min, 8000 x *g*, 4°C). The supernatant was collected and split into four 200 µl aliquots for the preparation of quadruplicate samples. The aliquots were stripped of non-protein contaminants by protein precipitation in acetone (1 to 9 volumes of acetone, -20°C and overnight). After centrifugation (15 minutes, 15000 x *g*, 4°C), the protein pellet was dried and resuspended in 200 µL of solubilization buffer (8 M urea, 0.1 M ammonium bicarbonate, pH 8). Protein concentration was determined in the RC-DC protein assay (Bio-Rad, Hercules, CA, USA). Equal amounts of protein were used for all three cell lines. Proteins were reduced (12 mM DTT, 37°C, 30 minutes) and alkylated (40 mM iodoacetamide, IAA, 25°C, 1 hour, in the dark). Samples were diluted in freshly prepared 0.1 M ammonium bicarbonate, to obtain a urea concentration of 1 M, and proteins were digested with trypsin (1:120 enzyme:substrate ratio, 37°C, overnight). Stable isotope-labeled synthetic peptides were used to spike samples. The samples were then acidified with formic acid (pH 3), desalted and concentrated by solid-phase extraction (Sep-Pak C18, 1 cc, 50 mg, Waters). The eluate volume was decreased in a vacuum centrifuge and adjusted with 0.1%

formic acid in water to obtain a final concentration of 4 µg/µL.

Relative quantification

Samples were prepared as described above. Equal volumes of a concentration-balanced mixture of crude stable isotope-labeled peptides were used to spike all samples before the solid phase extraction (SPE) step. This approach was used to correct signal fluctuations and biases introduced during the sample preparation steps. For each peptide, the ratios of the light to heavy peak areas were compared to obtain a relative quantification for the three cell lines. Four sample-preparation replicates were generated and each was analyzed in three injection replicates.

Absolute quantification

Samples were prepared as described above. A pool of the three cell lines was used to mimic the sample matrix. A calibration curve covering a range extending from 1.6 to 50 fmol internal standard (IS) peptide (IDFGTHISGR) in 10 µg of sample matrix injected into the column was generated with this sample pool. The calibration curve was established by plotting the mean chromatographic peak area (using the sum of peak areas for the three selected transitions) from triplicate injections against the amounts of IS peptide injected.

Target peptide selection

Using the UniProtKB/Swiss-Prot protein database containing splicing isoforms, we selected peptides that were unique and specific to MetAP1 and MetAP2. Priority was given to peptides that had already been identified in previous shotgun experiments on equivalent samples, preferentially without fractionation, with high-quality MS/MS spectra. The chosen peptides were 10 to 15 amino acids long, and their sequences contained no missed-cleavage sites or methionine residues. Six peptides were chosen for the first relative quantification experiment (LQCPTCIK, LGIQGSYFCSQECFK and HAQANGFSVVR for MetAP1 and ALDQASEEIWNDFR, IDFGTHISGR and NLNGHSIGQYR for MetAP2) and one peptide (IDFGTHISGR) was used for the absolute quantification of MetAP2.

Transition selection

The best transitions for each peptide were selected by analyzing heavy isotope-labeled peptides by Nano-LC ion-trap coupling (Agilent 1100 Series Nanoflow LC system (Agilent Technologies, Palo Alto, USA) coupled to an amaZon ETD ion trap (Bruker Daltonics, Bremen, Germany) to generate a spectral library. From the MS/MS spectra stored in the spectral library, we were able to extract and monitor at least six y-ion-type transitions for each peptide, with an unscheduled method, on a micro-LC triple quadrupole system (Dionex Ultimate 3000 system coupled to a TSQ Vantage Triple Quadrupole instrument (Thermo Fischer Scientific, San Jose, CA, USA)). This made it possible to determine the retention times of all the targeted peptides, checking the co-elution of endogenous and isotopically labeled peptides, eliminating transitions with interference and adjusting the isotopically labeled peptide concentrations. A concentration-balanced mixture of the crude peptides was prepared, to obtain comparable signal intensities for light and heavy transitions. For each peptide, at least three transitions were monitored for quantification. The complete list of transitions measured is presented in Supplementary Table 2.

Micro-LC-SRM peptide characterization

Peptides were analyzed on a Dionex Ultimate 3000 system coupled to a TSQ Vantage Triple Quadrupole instrument (Thermo Fisher Scientific, San Jose, CA, USA). For each analysis, a volume of 2.5 µL of sample, corresponding to 10 µg of protein, was injected and trapped on a precolumn (Zorbax C18 stable bond, 5 µm, 1.0 × 17 mm, Agilent Technologies) and then separated on a C18 column (Zorbax 300 SB C18, 3.5 µm, 150 × 0.3 mm, Agilent Technologies). The peptides were eluted with a linear gradient of 2% acetonitrile/98% water/0.1% formic acid (solvent A) and 98% acetonitrile/2% water/0.1% formic acid (solvent B). Trapping was performed for 3 minutes, at a flow rate of 50 $\mu L.min^{-1}$ with solvent A. Elution was performed at a flow rate of 5 $\mu L.min^{-1}$ with the following gradient: 3 minutes of 5% B; from 5% to 35% B in 40 minutes; 5 minutes at 80% B; 16 minutes at 5% B. For optimal micro-LC-SRM, the TSQ vantage mass spectrometer was operated with the following parameters: the system was operated in positive mode, the ion spray voltage was set at 3000 V, the capillary temperature at 300°C, the nitrogen collision gas pressure was set to 1.5 mTorr, Q1 and Q3 resolution was set to 0.7 Da and the collision energy was optimized individually for each transition. Unscheduled SRM was used, with a cycle time of 3 s and a dwell time of 34 ms for each transition. The system was controlled with Chromeleon Xpress software (v. 6.8) for the liquid chromatography system and Xcalibur (v. 2.1.0) software for the mass spectrometry system.

Micro-LC-SRM data analysis

The Skyline open-source software package [4] was used to visualize the SRM data, to perform peak selection and transition peak area integration, and to verify SRM peak group identification manually by

checking the co-elution and the correct relative fragment-ion intensities between light and heavy isotope-labeled peptides. The coefficient of variation for peptide retention time was calculated from all measurements of the light and heavy peptide over all replicates (Supplementary Table 2). For the relative quantification experiment, the overall reproducibility of the experiment was verified by calculating light/heavy isotope area ratios for the sum of all transitions, and checking that coefficients of variation were below 20% for all sample preparation replicates. Relative protein quantification and testing for differential protein expression were performed with the R package MSstats (http://www.R-project.org). The acceptance criteria for statistically different protein abundance changes between two conditions were set at a *p*-value below 0.05 and a fold-change of more than 1,2. For the absolute quantification experiment, the linearity criteria required experimental dots in standard curves to display a mean CV of less than 15% between triplicate injections. Experimental dots also had to fall within the mean 80–120% accuracy range when calculating the expected injected amounts from regression equations after base 2 logarithmic transformations (Supplementary Table 2). The limit of quantitation (LOQ) is the lowest point satisfying all the above mentioned criteria. Only the points satisfying all these criteria were used to calculate the linear regression equation and correlation coefficient. Endogenous peptide levels in the three cell lines were measured in sample preparation triplicates (Supplementary Table 2).

SUPPLEMENTARY REFERENCES

1. Linster E, Stephan I, Bienvenut WV, Maple-Grodem J, Myklebust LM, Huber M, Reichelt M, Sticht C, Geir Moller S, Meinnel T, Arnesen T, Giglione C, Hell R, Wirtz M. Downregulation of N-terminal acetylation triggers ABA-mediated drought responses in Arabidopsis. Nature communications. 2015; 6:7640.
2. Bienvenut WV, Sumpton D, Lilla S, Martinez A, Meinnel T, Giglione C. Influence of various endogenous and artefact modifications on large-scale proteomics analysis. Rapid communications in mass spectrometry : RCM. 2013; 27:443-450.
3. Vizcaino JA, Cote RG, Csordas A, Dianes JA, Fabregat A, Foster JM, Griss J, Alpi E, Birim M, Contell J, O'Kelly G, Schoenegger A, Ovelleiro D, Perez-Riverol Y, Reisinger F, Rios D, et al. The PRoteomics IDEntifications (PRIDE) database and associated tools: status in 2013. Nucleic Acids Res. 2013; 41:D1063-1069.
4. MacLean B, Tomazela DM, Shulman N, Chambers M, Finney GL, Frewen B, Kern R, Tabb DL, Liebler DC, MacCoss MJ. Skyline: an open source document editor for creating and analyzing targeted proteomics experiments. Bioinformatics. 2010; 26:966-968.

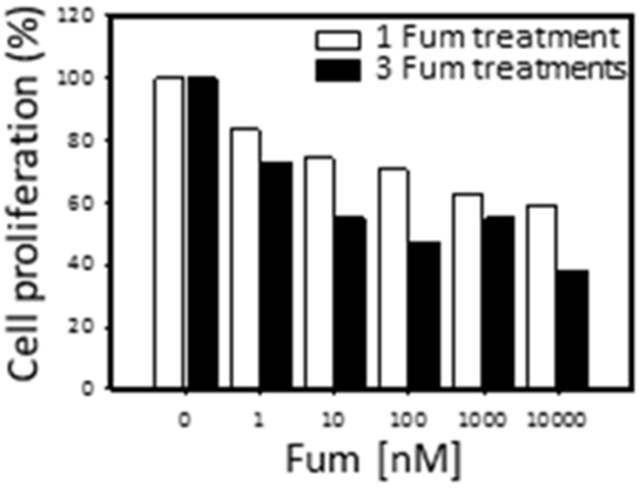

Supplementary Figure S1: Multiple fumagillin treatments do not significantly decrease the cell proliferation rate. HCT116, an insensitive line, was cultured for 72 h with a single fumagillin treatment (white bars) or with the medium replaced with fresh fumagillin-containing medium every 24 h (black bars) before the assessment of proliferation. Fum, fumagilllin.

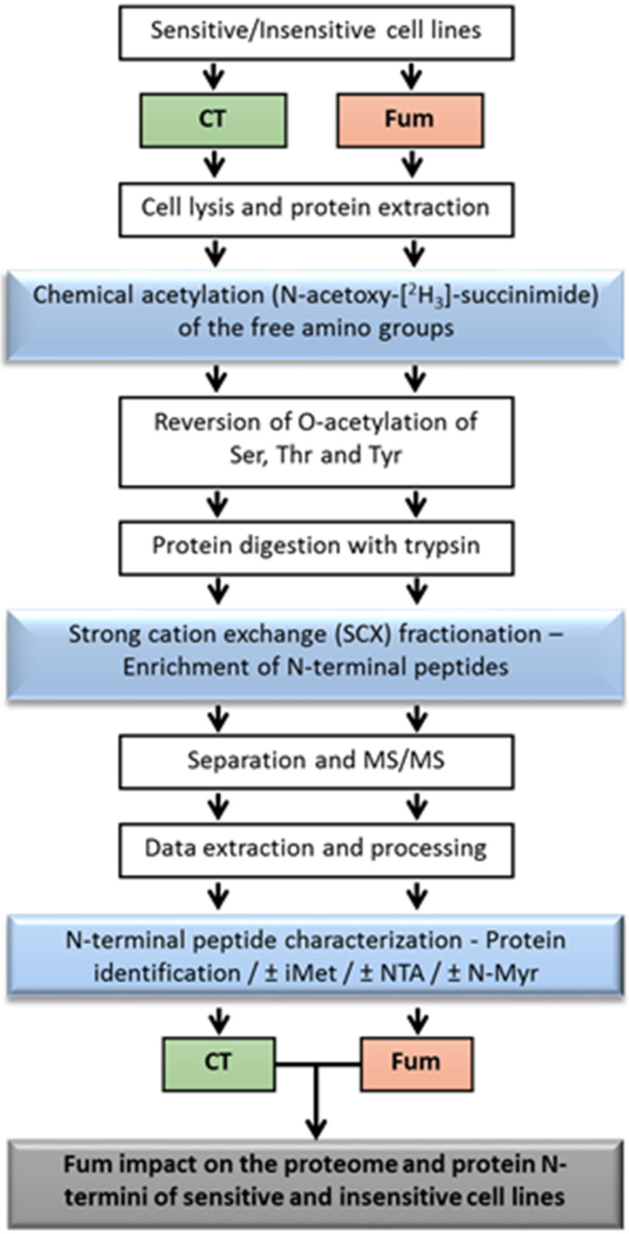

Supplementary Figure S2: Mass spectrometry pipeline for the large-scale identification of N-terminal peptides and modifications. Experimental design and procedure used to identify protein N-termini and to determine their status. The different cell lines were left untreated (CT) or were treated (Fum) before sample processing. The key steps are shown in blue and include the chemical heavy isotope acetylation of N-termini, making it possible to differentiate endogenous acetylation from free N-termini. The strong cation exchange step enriches the preparation in N-terminal peptides. MS and data processing are then used to identify the protein and its N-terminal modifications. Further details are provided in the Materials and Methods section.

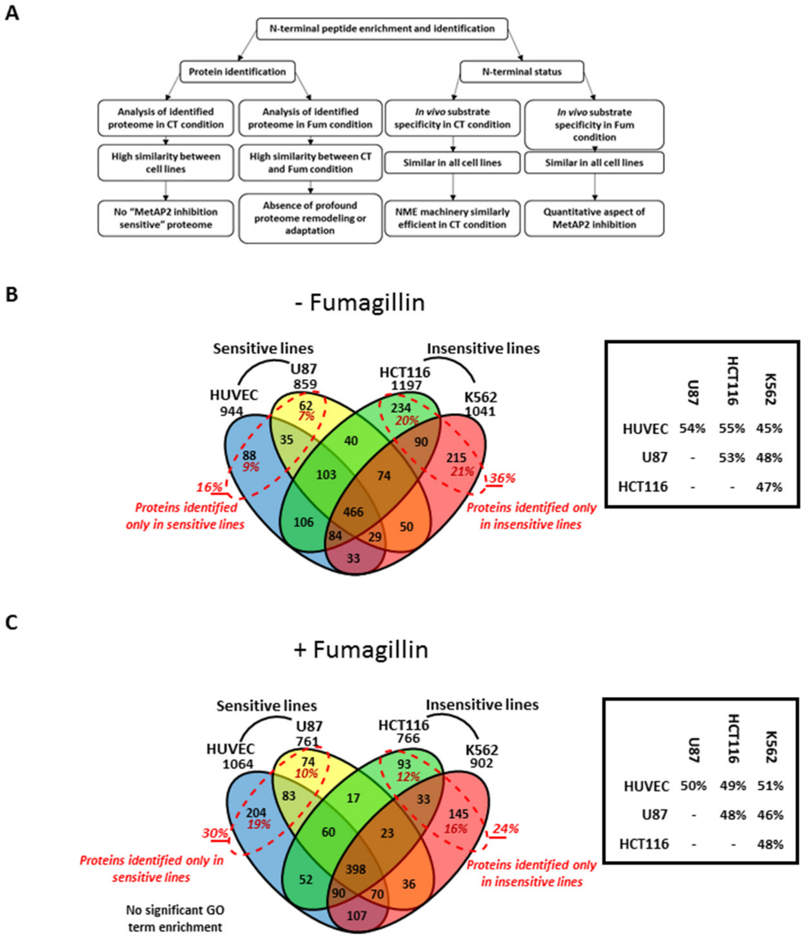

Supplementary Figure S3: Distribution of the identified proteins between cell lines. The overall scheme of mass spectrometry data generated in the course of the study is given **A.** This includes the status of N-terminal peptide and the respective identification of the proteins. In addition, for each branch the main conclusion is given for clarity. All proteins identified by mass spectrometry for each cell line were compared in control samples **B.** and in fumagillin-treated samples **C.** The insets in B and C depict pairwise comparisons of cell lines to analyze the overlap in the proteins identified.

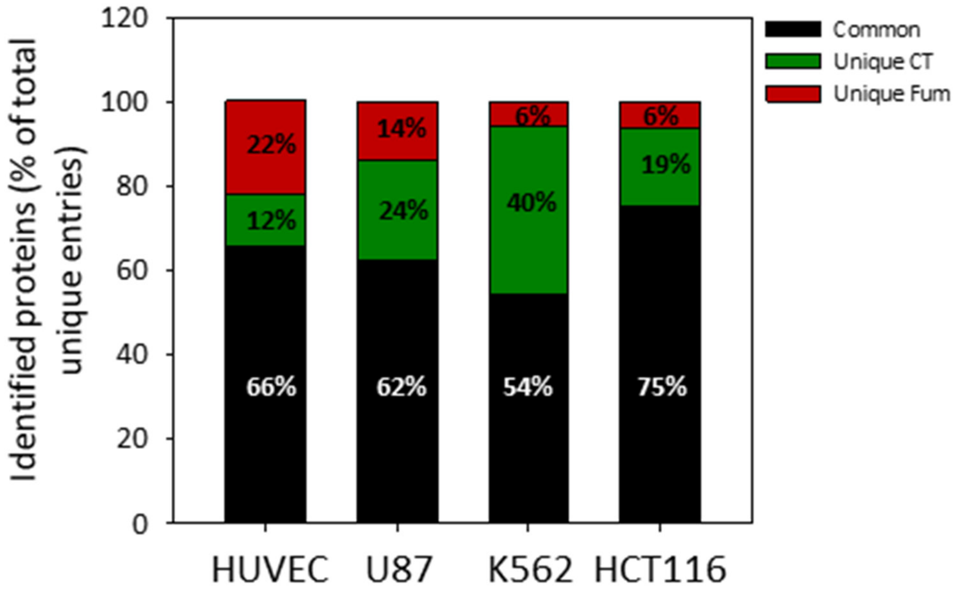

Supplementary Figure S4: Distribution of proteins identified in control cells and cells in which MetAP2 was inhibited. Distribution of the identified proteins between control and fumagillin-treated samples for each cell line (*x*-axis). Proteins identified in both control and treated samples are shown in black. Proteins found only in control samples are shown in green and proteins found only in fumagillin-treated samples are shown in red. 100% corresponds to the total number of unique entries for a given cell line.

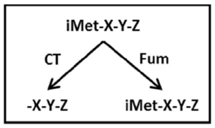

HUVEC

Protein AC	Description	Gene	N-term	CT	Fum	Pred.
**P11586**	**C-1-tetrahydrofolate synthase, cytoplasmic**	**MTHFD1**	MAPAEILNGK	-	+/-	A(2)
P62899	60S ribosomal protein L31	RPL31	MAPAKKGGEK	-	+/-	A(2)
O60427	Fatty acid desaturase 1	FADS1	MAPDPVAAET	-	+/-	A(2)
Q9UNL2	Translocon-associated protein subunit gamma	SSR3	MAPKGSSKQQ	-	+/-	A(2)
**P26583**	**High mobility group protein B2**	**HMGB2**	MGKGDPNKPR	-	+/-	G(2)
O95864	Fatty acid desaturase 2	FADS2	MGKGGNQGEG	-	+/-	G(2)
P04406	Glyceraldehyde-3-phosphate dehydrogenase	GAPDH	MGKVKVGVNG	-	+/-	G(2)
P09651	Heterogeneous nuclear ribonucleoprotein A1	HNRNPA1	MSKSESPKEP	-	+/-	Ac-S(2)
P05141	ADP/ATP translocase 2	SLC25A5	MTDAAVSFAK	-	+/-	Ac-T(2)
P07305	Histone H1.0	H1F0	MTENSTSAPA	-	+/-	Ac-T(2)
P63244	Guanine nucleotide-binding protein subunit beta-2-like 1	GNB2L1	MTEQMTLRGT	-	+/-	Ac-T(2)
Q96AG4	Leucine-rich repeat-containing protein 59	LRRC59	MTKAGSKGGN	-	+/-	Ac-T(2)
P46977	Dolichyl-diphosphooligosaccharide-protein glycosyltransferase subunit STT3A	STT3A	MTKFGFLRLS	-	+/-	Ac-T(2)
P61927	60S ribosomal protein L37	RPL37	MTKGTSSFGK	-	+/-	Ac-T(2)
Q9NPJ3	Acyl-coenzyme A thioesterase 13	ACOT13	MTSMTQSLRE	-	+/-	Ac-T(2)
Q32MZ4	Leucine-rich repeat flightless-interacting protein 1	LRRFIP1	MTSPAAAQSR	-	+/-	Ac-T(2)
P62888	60S ribosomal protein L30	RPL30	MVAAKKTKKS	-	+/-	V(2)
Q9UBE0	SUMO-activating enzyme subunit 1	SAE1	MVEKEEAGGG	-	+/-	Ac-V(2)
**O43396**	**Thioredoxin-like protein 1**	**TXNL1**	MVGVKPVGSD	-	+/-	V(2)
P36871	Phosphoglucomutase-1	PGM1	MVKIVTVKTQ	-	+/-	V(2)
Q9P0U1	Mitochondrial import receptor subunit TOM7 homolog	TOMM7	MVKLSKEAKQ	-	+/-	V(2)
P48739	Phosphatidylinositol transfer protein beta isoform	PITPNB	MVLIKEFRVV	-	+/-	V(2)
Q00169	Phosphatidylinositol transfer protein alpha isoform	PITPNA	MVLLKEYRVI	-	+/-	V(2)
O75396	Vesicle-trafficking protein SEC22b	SEC22B	MVLLTMIARV	-	+/-	V(2)
Q9UN86	Ras GTPase-activating protein-binding protein 2	G3BP2	MVMEKPSPLL	-	+/-	V(2)
P13639	Elongation factor 2	EEF2	MVNFTVDQIR	-	+/-	V(2)
Q969Q0	60S ribosomal protein L36a-like	RPL36AL	MVNVPKTRRT	-	+/-	V(2)
Q96MC6	Hippocampus abundant transcript 1 protein	HIAT1	MTQGKKKKRA	-	+	Ac-T(2)
Q13823	Nucleolar GTP-binding protein 2	GNL2	MVKPKYKGRS	-	+	V(2)
Q7Z4W1	L-xylulose reductase	DCXR	MELFLAGRRV	+/-	+	Ac-M(1)
P61326	Protein mago nashi homolog	MAGOH	MESDFYLRYY	+/-	+	Ac-M(1)
Q9BWD1	Acetyl-CoA acetyltransferase, cytosolic	ACAT2	MNAGSDPVVI	+/-	+	Ac-M(1)
P56385	ATP synthase subunit e, mitochondrial	ATP5I	MVPPVQVSPL	+/-	+	M(1)
Q9BU89	Deoxyhypusine hydroxylase	DOHH	MVTEQEVDAI	+/-	+	V(2)
P25705-2	Isoform 2 of ATP synthase subunit alpha, mitochondrial	ATP5A1	MSSILEERIL		+/-	Ac-S(2)
P49419-2	Isoform 2 of Alpha-aminoadipic semialdehyde dehydrogenase	ALDH7A1	MSTLLINQPQ		+/-	Ac-S(2)
P48449	Lanosterol synthase	LSS	MTEGTCLRRR		+/-	Ac-T(2)
Q92615	La-related protein 4B	LARP4B	MTSDQDAKVV		+/-	Ac-T(2)
P62937	Peptidyl-prolyl cis-trans isomerase A	PPIA	MVNPTVFFDI		+/-	V(2)
Q9UIJ5	Palmitoyltransferase ZDHHC2	ZDHHC2	MAPSGPGSSA		+	A(2)
Q6NUT3	Major facilitator superfamily domain-containing protein 12	MFSD12	MGPGPPAAGA		+	G(2)
Q9H446	RWD domain-containing protein 1	RWDD1	MTDYGEEQRN		+	Ac-T(2)
Q9P0B6	Coiled-coil domain-containing protein 167	CCDC167	MTKKKRENLG		+	Ac-T(2)
Q15758	Neutral amino acid transporter B(0)	SLC1A5	MVADPPRDSK		+	V(2)
Q8N6R1	Stress-associated endoplasmic reticulum protein 2	SERP2	MVAKQRIRMA		+	V(2)

U87

Protein AC	Description	Gene	N-term	CT	Fum	Pred.
P09651	Heterogeneous nuclear ribonucleoprotein A1	HNRNPA1	MSKSESPKEP	-	+/-	Ac-S(2)
**P43003**	**Excitatory amino acid transporter 1**	**SLC1A3**	MTKSNGEEPK	-	+/-	Ac-T(2)
P06396-2	Isoform 2 of Gelsolin	GSN	MVVEHPEFLK	-	+/-	V(2)
P62937	Peptidyl-prolyl cis-trans isomerase A	PPIA	MVNPTVFFDI	+/-	+	V(2)
B2RPK0	Putative high mobility group protein B1-like 1	HMGB1P1	MGKGDPKKPR		+/-	G(2)
P07305	Histone H1.0	H1F0	MTENSTSAPA		+	Ac-T(2)
Q9H981	Actin-related protein 8	ACTR8	MTQAEKGDTE		+	Ac-T(2)
Q32MZ4	Leucine-rich repeat flightless-interacting protein 1	LRRFIP1	MTSPAAAQSR		+	Ac-T(2)
Q8N6R1	Stress-associated endoplasmic reticulum protein 2	SERP2	MVAKQRIRMA		+	V(2)

HCT-116

Protein AC	Description	Gene	N-term	CT	Fum	Pred.
**P11586**	**C-1-tetrahydrofolate synthase, cytoplasmic**	**MTHFD1**	MAPAEILNGK	-	+/-	A(2)
P04406	Glyceraldehyde-3-phosphate dehydrogenase	GAPDH	MGKVKVGVNG	-	+/-	G(2)
P46977	Dolichyl-diphosphooligosaccharide-protein glycosyltransferase subunit STT3A	STT3A	MTKFGFLRLS	-	+/-	Ac-T(2)
P61927	60S ribosomal protein L37	RPL37	MTKGTSSFGK	-	+/-	Ac-T(2)
P62888	60S ribosomal protein L30	RPL30	MVAAKKTKKS	-	+/-	V(2)
P36871	Phosphoglucomutase-1	PGM1	MVKIVTVKTQ	-	+/-	V(2)
P61011	Signal recognition particle 54 kDa protein	SRP54	MVLADLGRKI	-	+/-	V(2)
P49591	Serine--tRNA ligase, cytoplasmic	SARS	MVLDLDLFRV	-	+/-	V(2)
Q00169	Phosphatidylinositol transfer protein alpha isoform	PITPNA	MVLLKEYRVI	-	+/-	V(2)
O75396	Vesicle-trafficking protein SEC22b	SEC22B	MVLLTMIARV	-	+/-	V(2)
Q9UN86	Ras GTPase-activating protein-binding protein 2	G3BP2	MVMEKPSPLL	-	+/-	V(2)
Q969Q0	60S ribosomal protein L36a-like	RPL36AL	MVNVPKTRRT	-	+/-	V(2)
Q9NZJ9	Diphosphoinositol polyphosphate phosphohydrolase 2	NUDT4	MMKFKPNQTR	-	+	*M(1)
O60427	Fatty acid desaturase 1	FADS1	MAPDPVAAET	+/-	+	A(2)
Q9BTX3	Transmembrane protein 208	TMEM208	MAPKGKVGTR	+/-	+	A(2)
P63104	14-3-3 protein zeta/delta	YWHAZ	MDKNELVQKA	+/-	+	Ac-M(1)
O95373	Importin-7	IPO7	MDPNTIIEAL	+/-	+	Ac-M(1)
P19623	Spermidine synthase	SRM	MEPGPDGPAA	+/-	+	Ac-M(1)
P78344	Eukaryotic translation initiation factor 4 gamma 2	EIF4G2	MESAIAEGGA	+/-	+	Ac-M(1)
Q9BZ72	Membrane-associated phosphatidylinositol transfer protein 2	PITPNM2	MIIKEYRIPL	+/-	+	M(1)
Q9Y265	RuvB-like 1	RUVBL1	MKIEEVKSTT	+/-	+	M(1)
Q96QD8	Sodium-coupled neutral amino acid transporter 2	SLC38A2	MKKAEMGRFS	+/-	+	M(1)
P55884	Eukaryotic translation initiation factor 3 subunit B	EIF3B	MQDAENVAVP	+/-	+	M(1)
Q9NPJ3	Acyl-coenzyme A thioesterase 13	ACOT13	MTSMTQSLRE	+/-	+	Ac-T(2)
B2RPK0	Putative high mobility group protein B1-like 1	HMGB1P1	MGKGDPKKPR		+	G(2)
Q9NWS8	Required for meiotic nuclear division protein 1 homolog	RMND1	MPATLLRAVA		+	P(2)

K562

Protein AC	Description	Gene	N-term	CT	Fum	Pred.
P60709	Actin, cytoplasmic 1	ACTB	MDDDIAALVV	-	+/-	Ac-M(1)
B2RPK0	Putative high mobility group protein B1-like 1	HMGB1P1	MGKGDPKKPR	-	+/-	G(2)
**P26583**	**High mobility group protein B2**	**HMGB2**	MGKGDPNKPR	-	+/-	G(2)
P04406	Glyceraldehyde-3-phosphate dehydrogenase	GAPDH	MGKVKVGVNG	-	+/-	G(2)
P62888	60S ribosomal protein L30	RPL30	MVAAKKTKKS	-	+/-	V(2)
P61011	Signal recognition particle 54 kDa protein	SRP54	MVLADLGRKI	-	+/-	V(2)
**Q8N5K1**	**CDGSH iron-sulfur domain-containing protein 2**	**CISD2**	MVLESVARIV	-	+/-	V(2)
Q00169	Phosphatidylinositol transfer protein alpha isoform	PITPNA	MVLLKEYRVI	-	+/-	V(2)
O75396	Vesicle-trafficking protein SEC22b	SEC22B	MVLLTMIARV	-	+/-	V(2)
Q969Q0	60S ribosomal protein L36a-like	RPL36AL	MVNVPKTRRT	-	+/-	V(2)
Q9P0U1	Mitochondrial import receptor subunit TOM7 homolog	TOMM7	MVKLSKEAKQ	-	+	V(2)
Q13823	Nucleolar GTP-binding protein 2	GNL2	MVKPKYKGRS	-	+	V(2)
Q9P0B6	Coiled-coil domain-containing protein 167	CCDC167	MTKKKRENLG		+	Ac-T(2)
P46778	60S ribosomal protein L21	RPL21	MTNTKGKRRG		+	Ac-T(2)
Q15758	Neutral amino acid transporter B(0)	SLC1A5	MVADPPRDSK		+	V(2)
O14802	DNA-directed RNA polymerase III subunit RPC1	POLR3A	MVKEQFRETD		+	V(2)

Supplementary Figure S5: Identification of proteins affected by MetAP2 inhibition. Proteins for which N-terminal peptides displayed lower levels of iMet cleavage after Fum treatment are shown in the tables, for each of the cell lines tested (inset). N-termini are extracted and isolated from Supplementary Table 1, for the sake of clarity. iMet cleaved (-, green); iMet partially cleaved (+/-, orange) and iMet retained (+, red). In bold, proteins annotated as being related to glutathione redox homeostasis.

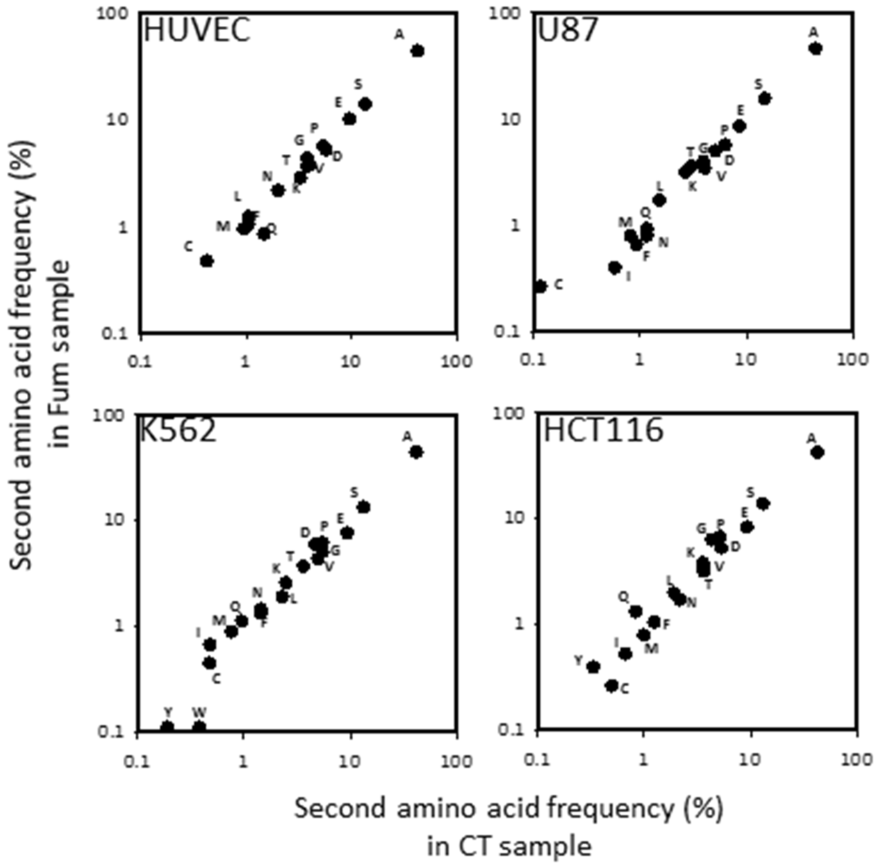

Supplementary Figure S6: The second amino acid frequency of the identified proteins is not affected by MetAP2 inhibition. Comparison of the second amino acid frequency (regardless of NME) between control (CT) and fumagillin-treated (Fum) samples, for each cell line. The *x* and *y*-axes are in logarithmic scales to facilitate visualization of the distribution. The second amino acid is the amino acid in the coding sequence immediately following the iMet. The one-code letter for amino acids is used.

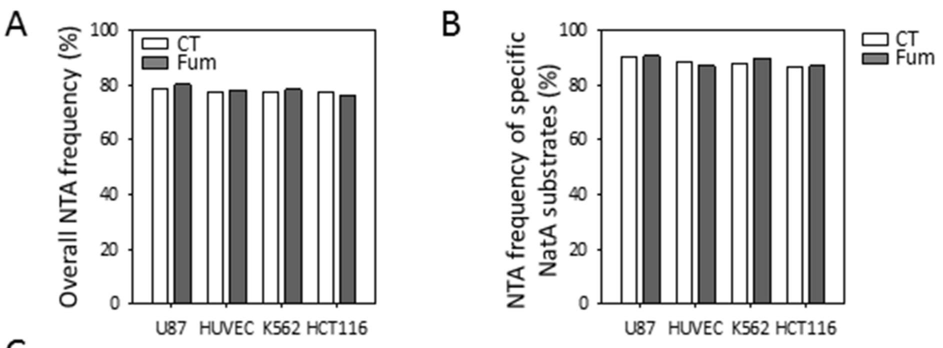

C

Accession	Description	Gene name	N-ter	CTRL	FUM
HCT116					
P12931	Proto-oncogene tyrosine-protein kinase Src	SRC	GSNKSKPKD	MYR/NTA	NTA
Q02952	A-kinase anchor protein 12	AKAP12	GAGSSTEQR	MYR	N/A
P49006	MARCKS-related protein	MARCKSL1	GSQSSKAPR	MYR	N/A
HUVEC					
Q02952	A-kinase anchor protein 12	AKAP12	GAGSSTEQR	MYR	MYR
P04899	Guanine nucleotide-binding protein G(i) subunit alpha-2	GNAI2	GCTVSAEDK	MYR	MYR
P49006	MARCKS-related protein	MARCKSL1	GSQSSKAPR	MYR	MYR
P29966	Myristoylated alanine-rich C-kinase substrate	MARCKS	GAQFSKTAA	MYR	N/A
K562					
P62191	26S protease regulatory subunit 4	PSMC1	GQSQSGGHG	MYR	MYR
P49006	MARCKS-related protein	MARCKSL1	GSQSSKAPR	MYR	MYR
Q02952	A-kinase anchor protein 12	AKAP12	GAGSSTEQR	N/A	MYR
Q96TA1	Niban-like protein 1	FAM129B	GDVLSTHLD	N/A	MYR
P12931	Proto-oncogene tyrosine-protein kinase Src	SRC	GSNKSKPKD	N/A	MYR
P04899	Guanine nucleotide-binding protein G(i) subunit alpha-2	GNAI2	GCTVSAEDK	MYR	N/A
Q9H8Y8	Golgi reassembly-stacking protein 2	GORASP2	GSSQSVEIP	MYR	N/A
U87					
Q02952	A-kinase anchor protein 12	AKAP12	GAGSSTEQR	MYR	MYR
O75896	Tumor suppressor candidate 2	TUSC2	GASGSKARG	MYR	N/A
Q8WXS3	Brain and acute leukemia cytoplasmic protein	BAALC	GCGGSRADA	MYR	N/A
P08754	Guanine nucleotide-binding protein G(k) subunit alpha-3	GNAI3	GCTLSAEDK	MYR	N/A
P04899	Guanine nucleotide-binding protein G(i) subunit alpha-2	GNAI2	GCTVSAEDK	MYR	MYR
Q96TA1	Niban-like protein 1	FAM129B	GDVLSTHLD	MYR	MYR
P37235	Hippocalcin-like protein 1	HPCAL1	GKQNSKLRP	MYR	N/A
O60936	Nucleolar protein 3	NOL3	GNAQERPSE	MYR	N/A
Q9Y478	5'-AMP-activated protein kinase subunit beta-1	PRKAB1	GNTSSERAA	MYR	N/A
P62191	26S protease regulatory subunit 4	PSMC1	GQSQSGGHG	MYR	N/A
P12931	Proto-oncogene tyrosine-protein kinase Src	SRC	GSNKSKPKD	MYR	MYR
P49006	MARCKS-related protein	MARCKSL1	GSQSSKAPR	MYR	N/A

Supplementary Figure S7: N-terminal acetylation and myristoylation in MetAP2 inhibited cells. A. Overall comparison of protein N-terminal acetylation frequency between control (CT) and fumagillin-treated samples (Fum), for each cell line. **B.** Comparison of protein N-terminal acetylation frequency for specific NatA substrates (M[ASTG]) between control (CT) and fumagillin-treated samples (Fum), for each cell line. **C.** Table of proteins identified as being myristoylated in at least one of the conditions, for each cell line. The status of the N-terminal peptide of the protein is shown for all conditions (CTRL/FUM). The Uniprot accession number for the protein, a description of the protein, the corresponding gene name and the N-terminal peptide identified are shown. MYR, myristoylation; NTA, N-terminal acetylation; N/A, not identified.

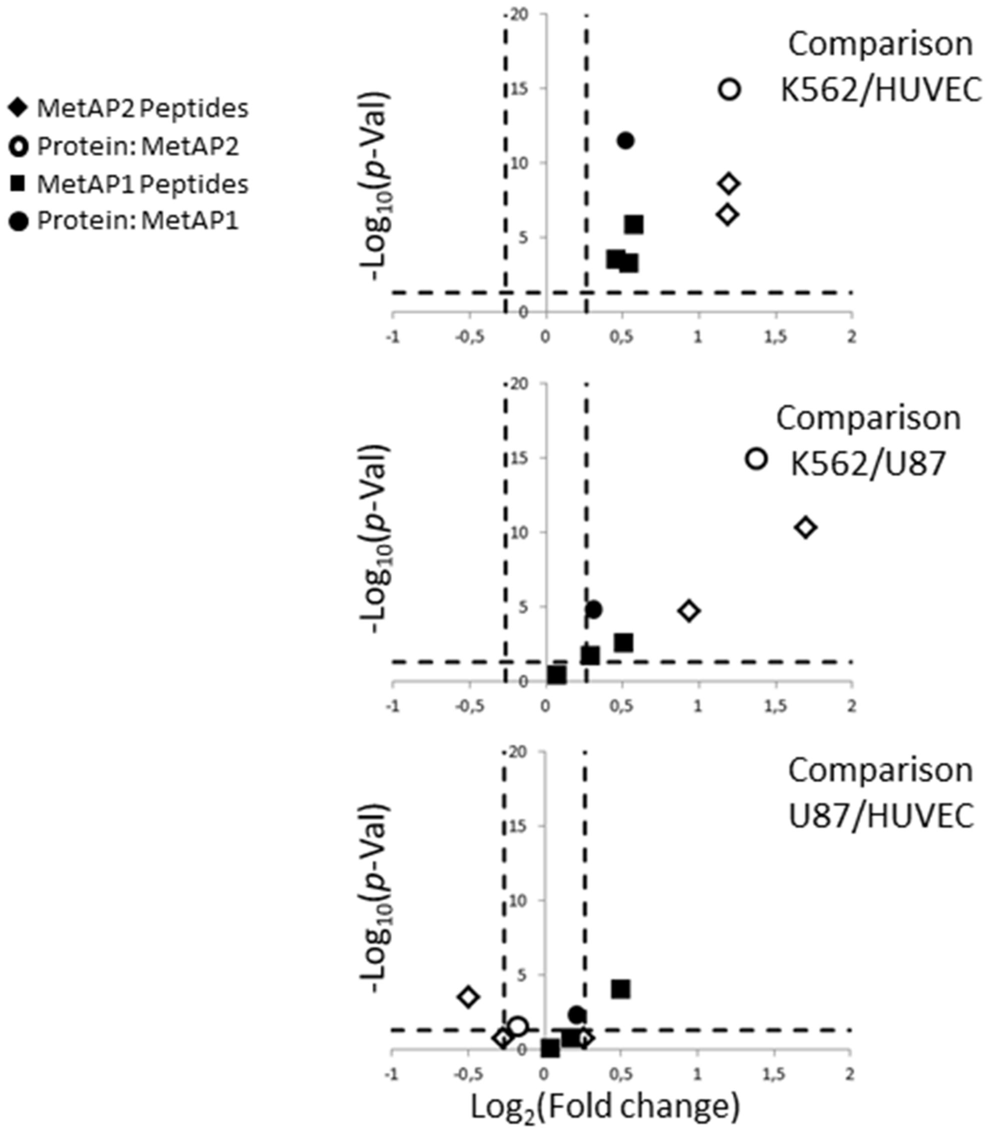

Supplementary Figure S8: Volcano plots representing the relative quantification results of MetAP1 and MetAP2 by LC-SRM. Pairwise comparisons of K562 versus HUVEC K562 versus U87 and U87 versus HUVEC cell lines are shown. The logarithm with base 2 of the fold changes are plotted against negative logarithm adjusted *p*-Values calculated with the R package MSstats for replicate injections, applied to individual peptides and to the summed peptides of MetAP1 and MetAP2 proteins. Horizontal and vertical dashed lines represent a *p*-Value of 0.05 and a fold change of 1.2, respectively. Peptide ALDQASEEIWNDFR is not shown here because it is below the LOD in two of the cell lines.

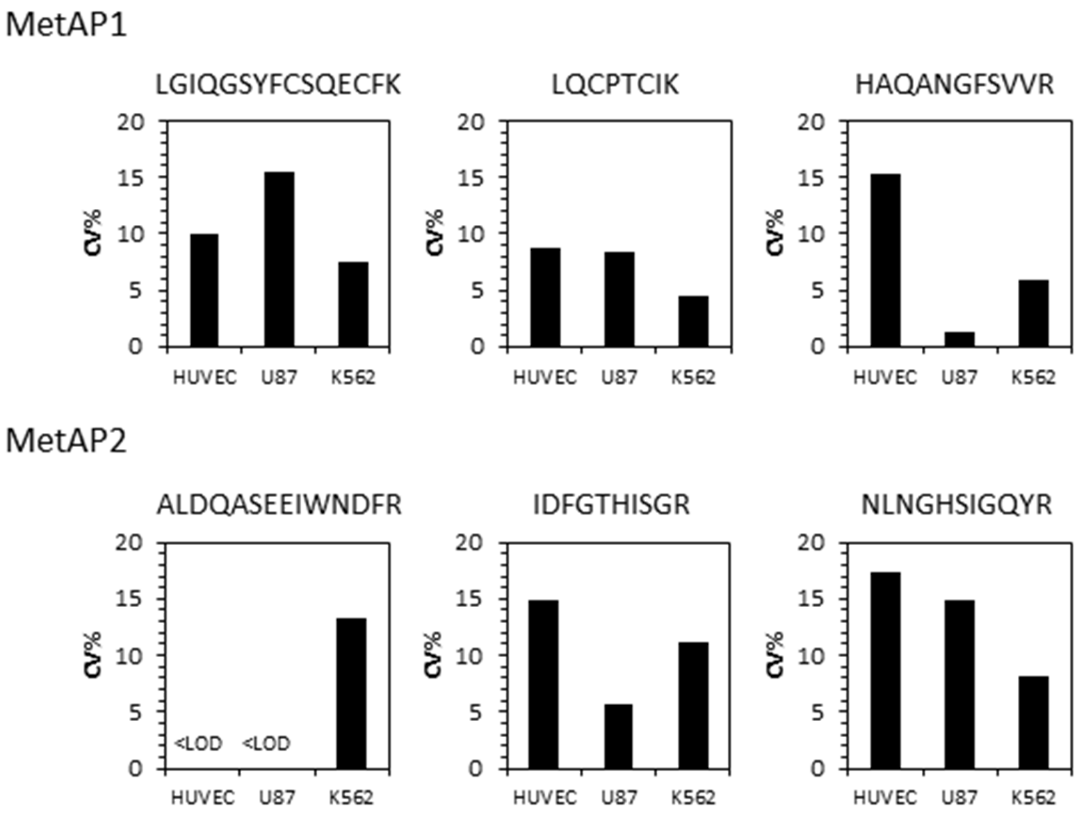

Supplementary Figure S9: Coefficients of variation associated to LC-SRM results. Coefficients of variation (CV%) for sample preparation quadruplicates* for the 6 targeted peptides for MetAP1 and MetAP2 in three different cell types. *except for one U87 sample that got lost during solid phase extraction.

Supplementary Table S1: Overview of protein N-terminus status. Overview of protein N-terminus status by cell line, for control and treated (fumagillin) conditions. For each column, symbols (0, -, +, +/-) indicate the status of the N-terminal peptide. "0": N-terminal peptide not characterized; "-": N-terminus peptide with no iMet; "+": N-terminus peptide with iMet retained; "+/-": N-terminus peptide characterized both with and without the iMet (partial cleavage).

See Supplementary File 1

Supplementary Table S2: The complete list of transitions measured for MetAP1 and MetAP2

See Supplementary File 2
