# Supplementary material for: MetAP1 and MetAP2 drive cell selectivity for a potent anti-cancer agent in synergy, by controlling glutathione redox state
Source: Oncotarget. 2016 Aug 11;7(39):63306–23. doi: 10.18632/oncotarget.11216 (PMC5325365; doi:10.18632/oncotarget.11216)
Supplement: Supplementary file 2 [file oncotarget-07-63306-s002.docx]

**Supplemental Table 1:** Overview of protein N-terminus status. Overview of protein N-terminus status by cell line, for control and treated (fumagillin) conditions. For each column, symbols (0, -, +, +/-) indicate the status of the N-terminal peptide. "0": N-terminal peptide not characterized; "-": N-terminus peptide with no iMet; "+": N-terminus peptide with iMet retained; "+/-": N-terminus peptide characterized both with and without the iMet (partial cleavage).

|  |  |  | **Cell lines** | **HCT116** | | **K562** | | **HUVEC** | | **U87** | |
| --- | --- | --- | --- | --- | --- | --- | --- | --- | --- | --- | --- |
|  |  |  |  |  |  |  |  |  |  |  |  |
| **Protein AC** | **Description** | **Gene Name** | **Theoritical Protein  N-terminus** | CT | Fum | CT | Fum | CT | Fum | CT | Fum |
| Q86U42 | Polyadenylate-binding protein 2 | PABPN1 | MAAAAAAAAA | - | - | - | - | - | - | - | - |
| Q16585 | Beta-sarcoglycan | SGCB | MAAAAAAAAE | - | - | 0 | 0 | - | - | 0 | 0 |
| Q96S94 | Cyclin-L2 | CCNL2 | MAAAAAAAGA | - | - | - | - | - | - | 0 | - |
| O75822 | Eukaryotic translation initiation factor 3 subunit J | EIF3J | MAAAAAAAGD | - | - | - | - | - | - | - | - |
| Q9UH36 | SRR1-like protein | SRRD | MAAAAAAALE | - | 0 | 0 | 0 | 0 | 0 | 0 | 0 |
| Q9H3H3-2 | Isoform 2 of UPF0696 protein C11orf68 | C11orf68 | MAAAAAAAVA | 0 | 0 | - | - | 0 | 0 | 0 | 0 |
| P28482 | Mitogen-activated protein kinase 1 | MAPK1 | MAAAAAAGAG | - | - | - | - | - | - | - | - |
| P09417 | Dihydropteridine reductase | QDPR | MAAAAAAGEA | - | - | 0 | - | - | - | - | - |
| Q9UID3 | Vacuolar protein sorting-associated protein 51 homolog | VPS51 | MAAAAAAGPS | - | 0 | - | - | 0 | - | - | - |
| Q9NR33 | DNA polymerase epsilon subunit 4 | POLE4 | MAAAAAAGSG | - | 0 | 0 | 0 | - | - | - | - |
| Q8WVM8 | Sec1 family domain-containing protein 1 | SCFD1 | MAAAAAATAA | - | - | - | - | - | - | - | - |
| Q86X55 | Histone-arginine methyltransferase CARM1 | CARM1 | MAAAAAAVGP | 0 | - | - | - | - | 0 | - | - |
| P51788 | Chloride channel protein 2 | CLCN2 | MAAAAAEEGM | - | 0 | 0 | 0 | 0 | 0 | 0 | 0 |
| O00410 | Importin-5 | IPO5 | MAAAAAEQQQ | - | - | - | - | - | - | 0 | 0 |
| Q9NXW9 | Alpha-ketoglutarate-dependent dioxygenase alkB homolog 4 | ALKBH4 | MAAAAAETPE | - | - | - | - | - | - | - | 0 |
| Q9BTV7 | CDK5 and ABL1 enzyme substrate 2 | CABLES2 | MAAAAAGGAP | 0 | - | 0 | - | 0 | 0 | 0 | - |
| P23610 | Factor VIII intron 22 protein | F8A1 | MAAAAAGLGG | 0 | 0 | 0 | 0 | - | 0 | - | 0 |
| Q9Y2Z0 | Suppressor of G2 allele of SKP1 homolog | SUGT1 | MAAAAAGTAT | - | - | - | - | - | - | - | - |
| Q7L5D6 | Golgi to ER traffic protein 4 homolog | GET4 | MAAAAAMAEQ | - | - | - | - | - | - | - | - |
| P27361 | Mitogen-activated protein kinase 3 | MAPK3 | MAAAAAQGGG | - | - | 0 | 0 | - | - | - | - |
| Q9Y2U8 | Inner nuclear membrane protein Man1 | LEMD3 | MAAAAASAPQ | - | - | - | - | - | - | - | - |
| Q5VYK3 | Proteasome-associated protein ECM29 homolog | ECM29 | MAAAAASASQ | - | - | - | - | - | - | - | - |
| Q9HD20 | Manganese-transporting ATPase 13A1 | ATP13A1 | MAAAAAVGNA | - | 0 | 0 | 0 | 0 | 0 | 0 | 0 |
| Q96E14 | RecQ-mediated genome instability protein 2 | RMI2 | MAAAADSFSG | 0 | - | 0 | 0 | - | 0 | 0 | 0 |
| Q99615 | DnaJ homolog subfamily C member 7 | DNAJC7 | MAAAAECDVV | 0 | 0 | - | - | 0 | 0 | - | - |
| Q6QNY1 | Biogenesis of lysosome-related organelles complex 1 subunit 2 | BLOC1S2 | MAAAAEGVLA | - | 0 | - | - | - | 0 | - | 0 |
| O43324 | Eukaryotic translation elongation factor 1 epsilon-1 | EEF1E1 | MAAAAELSLL | 0 | 0 | - | - | 0 | - | - | 0 |
| Q96KQ7 | Histone-lysine N-methyltransferase EHMT2 | EHMT2 | MAAAAGAAAA | 0 | 0 | - | - | 0 | 0 | 0 | 0 |
| Q6NXT1 | Ankyrin repeat domain-containing protein 54 | ANKRD54 | MAAAAGDADD | 0 | 0 | 0 | 0 | 0 | 0 | 0 | - |
| Q9HCJ3 | Ribonucleoprotein PTB-binding 2 | RAVER2 | MAAAAGDGGG | - | - | 0 | 0 | - | - | 0 | 0 |
| Q92922 | SWI/SNF complex subunit SMARCC1 | SMARCC1 | MAAAAGGGGP | - | 0 | - | - | 0 | - | 0 | 0 |
| Q5SRE5 | Nucleoporin NUP188 homolog | NUP188 | MAAAAGGPCV | - | 0 | 0 | 0 | - | - | 0 | - |
| Q00796 | Sorbitol dehydrogenase | SORD | MAAAAKPNNL | - | - | 0 | 0 | 0 | 0 | - | - |
| Q3KQV3 | Zinc finger protein 792 | ZNF792 | MAAAALRDPA | 0 | 0 | 0 | 0 | 0 | - | 0 | 0 |
| O43615 | Mitochondrial import inner membrane translocase subunit TIM44 | TIMM44 | MAAAALRSGW | 0 | 0 | 0 | 0 | - | 0 | 0 | 0 |
| Q9NX46 | Poly(ADP-ribose) glycohydrolase ARH3 | ADPRHL2 | MAAAAMAAAA | - | - | - | - | - | - | - | - |
| Q15283 | Ras GTPase-activating protein 2 | RASA2 | MAAAAPAAAA | 0 | 0 | - | - | 0 | 0 | - | - |
| Q9Y584 | Mitochondrial import inner membrane translocase subunit Tim22 | TIMM22 | MAAAAPNAGG | 0 | 0 | - | 0 | 0 | 0 | 0 | 0 |
| Q9H5N1 | Rab GTPase-binding effector protein 2 | RABEP2 | MAAAAPVAAD | 0 | 0 | 0 | 0 | 0 | - | 0 | - |
| P42566 | Epidermal growth factor receptor substrate 15 | EPS15 | MAAAAQLSLT | - | 0 | - | - | 0 | - | - | 0 |
| Q8TBC3 | SH3KBP1-binding protein 1 | SHKBP1 | MAAAATAAEG | - | - | - | - | - | - | - | - |
| Q8N1B4 | Vacuolar protein sorting-associated protein 52 homolog | VPS52 | MAAAATMAAA | - | 0 | - | - | - | - | - | - |
| Q9NVR0 | Kelch-like protein 11 | KLHL11 | MAAAAVAAAA | 0 | 0 | 0 | 0 | 0 | 0 | 0 | 0 |
| Q96K37 | Solute carrier family 35 member E1 | SLC35E1 | MAAAAVGAGH | 0 | - | 0 | 0 | 0 | 0 | 0 | - |
| Q15005 | Signal peptidase complex subunit 2 | SPCS2 | MAAAAVQGGR | - | - | - | - | - | - | - | - |
| Q8N1G4 | Leucine-rich repeat-containing protein 47 | LRRC47 | MAAAAVSESW | 0 | 0 | - | - | 0 | - | - | - |
| P49914 | 5-formyltetrahydrofolate cyclo-ligase | MTHFS | MAAAAVSSAK | - | - | - | - | - | - | - | 0 |
| O00231 | 26S proteasome non-ATPase regulatory subunit 11 | PSMD11 | MAAAAVVEFQ | - | - | - | - | - | - | - | - |
| Q8NI27 | THO complex subunit 2 | THOC2 | MAAAAVVVPA | - | 0 | - | - | 0 | - | - | 0 |
| Q9H9B1 | Histone-lysine N-methyltransferase EHMT1 | EHMT1 | MAAADAEAVP | - | 0 | 0 | - | 0 | - | 0 | 0 |
| P23025 | DNA repair protein complementing XP-A cells | XPA | MAAADGALPE | - | - | 0 | 0 | - | - | 0 | 0 |
| P30153 | Serine/threonine-protein phosphatase 2A 65 kDa regulatory subunit A alpha isoform | PPP2R1A | MAAADGDDSL | - | 0 | - | - | - | - | - | 0 |
| A6NDG6 | Phosphoglycolate phosphatase | PGP | MAAAEAGGDD | - | 0 | 0 | 0 | - | 0 | 0 | - |
| Q99942 | E3 ubiquitin-protein ligase RNF5 | RNF5 | MAAAEEEDGG | - | 0 | 0 | 0 | 0 | - | 0 | - |
| P55263 | Adenosine kinase | ADK | MAAAEEEPKP | - | - | - | - | - | - | - | - |
| Q53H96 | Pyrroline-5-carboxylate reductase 3 | PYCRL | MAAAEPSPRR | - | 0 | 0 | 0 | 0 | - | 0 | - |
| Q13523 | Serine/threonine-protein kinase PRP4 homolog | PRPF4B | MAAAETQSLR | - | 0 | 0 | 0 | - | - | 0 | - |
| Q9UHI6 | Probable ATP-dependent RNA helicase DDX20 | DDX20 | MAAAFEASGA | 0 | 0 | - | 0 | 0 | 0 | 0 | 0 |
| Q9UL25 | Ras-related protein Rab-21 | RAB21 | MAAAGGGGGG | - | 0 | 0 | - | - | - | 0 | - |
| Q6ZN18 | Zinc finger protein AEBP2 | AEBP2 | MAAAITDMAD | - | 0 | 0 | 0 | 0 | 0 | 0 | 0 |
| Q9UJS0 | Calcium-binding mitochondrial carrier protein Aralar2 | SLC25A13 | MAAAKVALTK | - | - | - | - | - | - | - | - |
| Q96EL3 | 39S ribosomal protein L53, mitochondrial | MRPL53 | MAAALARLGL | - | 0 | 0 | 0 | - | 0 | 0 | 0 |
| Q96EV2 | RNA-binding protein 33 | RBM33 | MAAALGASGG | - | - | - | - | - | - | - | - |
| P62877 | E3 ubiquitin-protein ligase RBX1 | RBX1 | MAAAMDVDTP | - | - | - | - | - | - | - | - |
| O43598 | -phosphate N-hydrolase 1 | DNPH1 | MAAAMVPGRS | - | - | - | - | - | - | - | 0 |
| Q9GZN8 | UPF0687 protein C20orf27 | C20orf27 | MAAANKGNKP | - | - | - | - | - | - | - | - |
| Q9BRD0 | BUD13 homolog | BUD13 | MAAAPPLSKA | - | 0 | 0 | 0 | 0 | 0 | 0 | 0 |
| Q12765 | Secernin-1 | SCRN1 | MAAAPPSYCF | - | 0 | 0 | 0 | - | 0 | 0 | 0 |
| P78345 | Ribonuclease P protein subunit p38 | RPP38 | MAAAPQAPGR | - | 0 | 0 | 0 | - | - | - | 0 |
| Q8TEL6 | Short transient receptor potential channel 4-associated protein | TRPC4AP | MAAAPVAAGS | - | 0 | 0 | 0 | - | - | - | - |
| O96005 | Cleft lip and palate transmembrane protein 1 | CLPTM1 | MAAAQEADGA | - | 0 | 0 | 0 | - | - | 0 | - |
| Q96AB3 | Isochorismatase domain-containing protein 2, mitochondrial | ISOC2 | MAAARPSLGR | 0 | 0 | 0 | 0 | 0 | 0 | 0 | 0 |
| Q5VWZ2 | Lysophospholipase-like protein 1 | LYPLAL1 | MAAASGSVLQ | - | - | - | - | - | - | - | - |
| Q6P6C2 | RNA demethylase ALKBH5 | ALKBH5 | MAAASGYTDL | - | - | - | - | - | - | - | 0 |
| Q6VN20 | Ran-binding protein 10 | RANBP10 | MAAATADPGA | - | 0 | - | - | 0 | 0 | - | - |
| Q9NWH9 | SAFB-like transcription modulator | SLTM | MAAATGAVAA | - | - | - | - | - | - | - | - |
| P12955 | Xaa-Pro dipeptidase | PEPD | MAAATGPSFW | 0 | 0 | - | 0 | 0 | 0 | 0 | 0 |
| Q9UDW1 | Cytochrome b-c1 complex subunit 9 | UQCR10 | MAAATLTSKL | 0 | 0 | - | - | 0 | - | 0 | 0 |
| Q9NS69 | Mitochondrial import receptor subunit TOM22 homolog | TOMM22 | MAAAVAAAGA | 0 | 0 | 0 | 0 | 0 | 0 | 0 | - |
| Q7Z5L9 | Interferon regulatory factor 2-binding protein 2 | IRF2BP2 | MAAAVAVAAA | - | - | - | - | - | - | - | - |
| Q8IZL8 | Proline-, glutamic acid- and leucine-rich protein 1 | PELP1 | MAAAVLSGPS | 0 | - | - | - | 0 | - | 0 | - |
| Q00688 | Peptidyl-prolyl cis-trans isomerase FKBP3 | FKBP3 | MAAAVPQRAW | - | 0 | 0 | 0 | 0 | 0 | 0 | - |
| A6NDU8 | UPF0600 protein C5orf51 | C5orf51 | MAAAVSSVVR | - | 0 | - | - | - | - | - | 0 |
| O43583 | Density-regulated protein | DENR | MAADISESSG | - | - | - | - | - | - | - | - |
| Q8TDD1 | ATP-dependent RNA helicase DDX54 | DDX54 | MAADKGPAAG | - | - | 0 | 0 | - | - | 0 | 0 |
| Q9HAU0 | Pleckstrin homology domain-containing family A member 5 | PLEKHA5 | MAADLNLEWI | - | 0 | 0 | 0 | 0 | 0 | 0 | 0 |
| Q92616 | Translational activator GCN1 | GCN1L1 | MAADTQVSET | - | - | - | - | - | - | - | - |
| Q8IY67 | Ribonucleoprotein PTB-binding 1 | RAVER1 | MAADVSVTHR | - | - | 0 | 0 | - | 0 | 0 | 0 |
| Q9Y5Q8 | General transcription factor 3C polypeptide 5 | GTF3C5 | MAAEAADLGL | - | 0 | - | - | - | - | - | 0 |
| Q96A65 | Exocyst complex component 4 | EXOC4 | MAAEAAGGKY | - | - | - | - | - | - | - | - |
| O75352 | Mannose-P-dolichol utilization defect 1 protein | MPDU1 | MAAEADGPLK | - | - | - | - | - | - | - | - |
| Q16576-2 | Isoform 2 of Histone-binding protein RBBP7 | RBBP7 | MAAEAGVVGA | - | 0 | 0 | 0 | 0 | 0 | 0 | 0 |
| A2RTX5 | Probable threonine--tRNA ligase 2, cytoplasmic | TARSL2 | MAAEALAAEA | - | - | 0 | - | 0 | - | 0 | 0 |
| P78318 | Immunoglobulin-binding protein 1 | IGBP1 | MAAEDELQLP | - | - | - | - | - | - | - | 0 |
| Q96FW1 | Ubiquitin thioesterase OTUB1 | OTUB1 | MAAEEPQQQK | - | 0 | - | - | - | 0 | - | 0 |
| Q96P53 | WD repeat and FYVE domain-containing protein 2 | WDFY2 | MAAEIQPKPL | 0 | 0 | 0 | 0 | 0 | 0 | - | - |
| Q9UHB9-2 | Isoform 2 of Signal recognition particle subunit SRP68 | SRP68 | MAAEKQVPGG | - | - | - | - | - | 0 | 0 | - |
| Q8N2W9 | E3 SUMO-protein ligase PIAS4 | PIAS4 | MAAELVEAKN | - | 0 | - | 0 | 0 | 0 | 0 | 0 |
| Q8N6H7 | ADP-ribosylation factor GTPase-activating protein 2 | ARFGAP2 | MAAEPNKTEI | - | 0 | - | - | - | - | - | - |
| Q6XQN6 | Nicotinate phosphoribosyltransferase | NAPRT | MAAEQDPEAR | - | 0 | 0 | 0 | 0 | 0 | 0 | 0 |
| Q96BP3 | Peptidylprolyl isomerase domain and WD repeat-containing protein 1 | PPWD1 | MAAESGSDFQ | - | - | 0 | 0 | - | - | - | - |
| O75420 | PERQ amino acid-rich with GYF domain-containing protein 1 | GIGYF1 | MAAETLNFGP | 0 | 0 | - | 0 | 0 | - | 0 | 0 |
| Q6Y7W6 | PERQ amino acid-rich with GYF domain-containing protein 2 | GIGYF2 | MAAETQTLNF | - | - | - | - | 0 | - | - | - |
| Q9GZS1-2 | Isoform 2 of DNA-directed RNA polymerase I subunit RPA49 | POLR1E | MAAEVLPSAR | - | 0 | - | - | 0 | - | 0 | 0 |
| P28288 | ATP-binding cassette sub-family D member 3 | ABCD3 | MAAFSKYLTA | - | 0 | 0 | 0 | 0 | 0 | 0 | 0 |
| O76003 | Glutaredoxin-3 | GLRX3 | MAAGAAEAAV | 0 | 0 | - | - | - | - | - | 0 |
| Q8WUD6 | Cholinephosphotransferase 1 | CHPT1 | MAAGAGAGSA | - | 0 | 0 | 0 | 0 | - | 0 | - |
| Q15554 | Telomeric repeat-binding factor 2 | TERF2 | MAAGAGTAGP | - | 0 | - | 0 | 0 | 0 | 0 | 0 |
| Q8N668 | COMM domain-containing protein 1 | COMMD1 | MAAGELEGGK | 0 | - | - | - | 0 | 0 | 0 | 0 |
| Q9Y3B8-3 | Isoform 3 of Oligoribonuclease, mitochondrial | REXO2 | MAAGESMAQR | - | - | 0 | 0 | - | - | 0 | 0 |
| Q96B26 | Exosome complex component RRP43 | EXOSC8 | MAAGFKTVEP | - | - | - | - | - | - | - | - |
| Q9UL63 | Muskelin | MKLN1 | MAAGGAVAAA | - | - | - | - | - | - | - | - |
| P30566 | Adenylosuccinate lyase | ADSL | MAAGGDHGSP | 0 | 0 | 0 | 0 | 0 | 0 | 0 | 0 |
| Q5VT52 | Regulation of nuclear pre-mRNA domain-containing protein 2 | RPRD2 | MAAGGGGGSS | - | - | 0 | 0 | - | - | - | 0 |
| Q9NZM5 | Glioma tumor suppressor candidate region gene 2 protein | GLTSCR2 | MAAGGSGVGG | - | 0 | 0 | 0 | - | - | 0 | 0 |
| O75815 | Breast cancer anti-estrogen resistance protein 3 | BCAR3 | MAAGKFASLP | 0 | 0 | 0 | 0 | - | 0 | 0 | 0 |
| Q92685 | Dol-P-Man:Man(5)GlcNAc(2)-PP-Dol alpha-1,3-mannosyltransferase | ALG3 | MAAGLRKRGR | 0 | 0 | 0 | 0 | 0 | 0 | 0 | 0 |
| Q15427 | Splicing factor 3B subunit 4 | SF3B4 | MAAGPISERN | - | - | 0 | 0 | - | - | - | - |
| O75607 | Nucleoplasmin-3 | NPM3 | MAAGTAAALA | - | - | - | - | - | - | - | - |
| P26641 | Elongation factor 1-gamma | EEF1G | MAAGTLYTYP | - | - | - | - | - | - | - | - |
| O95249 | Golgi SNAP receptor complex member 1 | GOSR1 | MAAGTSSYWE | - | - | - | - | 0 | - | - | 0 |
| O43681 | ATPase ASNA1 | ASNA1 | MAAGVAGWGV | 0 | 0 | - | - | 0 | 0 | 0 | 0 |
| Q5TBB1 | Ribonuclease H2 subunit B | RNASEH2B | MAAGVDCGDG | - | 0 | - | 0 | - | - | - | 0 |
| P52272 | Heterogeneous nuclear ribonucleoprotein M | HNRNPM | MAAGVEAAAE | 0 | 0 | - | - | 0 | 0 | 0 | 0 |
| O95926 | Pre-mRNA-splicing factor SYF2 | SYF2 | MAAIAASEVL | 0 | 0 | - | - | 0 | 0 | - | 0 |
| Q9UJ70 | N-acetyl-D-glucosamine kinase | NAGK | MAAIYGGVEG | - | - | - | - | - | - | - | - |
| O14683 | Tumor protein p53-inducible protein 11 | TP53I11 | MAAKQPPPLM | - | 0 | 0 | 0 | 0 | 0 | 0 | 0 |
| Q9UBM7 | 7-dehydrocholesterol reductase | DHCR7 | MAAKSQPNIP | - | - | - | - | - | - | - | - |
| P35232 | Prohibitin | PHB | MAAKVFESIG | 0 | - | - | - | 0 | 0 | 0 | 0 |
| P47897 | Glutamine--tRNA ligase | QARS | MAALDSLSLF | 0 | 0 | - | - | 0 | - | - | 0 |
| Q9NUP9 | Protein lin-7 homolog C | LIN7C | MAALGEPVRL | - | 0 | 0 | 0 | - | - | - | - |
| Q9Y256 | CAAX prenyl protease 2 | RCE1 | MAALGGDGLR | - | 0 | - | - | - | - | 0 | 0 |
| P35269 | General transcription factor IIF subunit 1 | GTF2F1 | MAALGPSSQN | - | - | - | - | - | - | - | - |
| Q86Y56 | HEAT repeat-containing protein 2 | HEATR2 | MAALGVAEAV | - | - | - | - | 0 | 0 | 0 | 0 |
| A6NFI3 | Zinc finger protein 316 | ZNF316 | MAALHTTPDS | - | - | - | - | 0 | - | 0 | 0 |
| Q9HA72 | Calcium homeostasis modulator protein 2 | CALHM2 | MAALIAENFR | - | - | 0 | 0 | 0 | - | - | 0 |
| P55199 | RNA polymerase II elongation factor ELL | ELL | MAALKEDRSY | - | 0 | 0 | 0 | 0 | - | 0 | 0 |
| P62910 | 60S ribosomal protein L32 | RPL32 | MAALRPLVKP | - | - | - | - | - | - | - | - |
| Q9P000 | COMM domain-containing protein 9 | COMMD9 | MAALTAEHFA | 0 | 0 | 0 | 0 | 0 | 0 | - | 0 |
| Q96KP4 | Cytosolic non-specific dipeptidase | CNDP2 | MAALTTLFKY | - | 0 | - | - | 0 | - | - | - |
| P27708 | CAD protein | CAD | MAALVLEDGS | - | - | - | - | - | - | - | - |
| O14744 | Protein arginine N-methyltransferase 5 | PRMT5 | MAAMAVGGAG | - | - | - | - | - | - | - | - |
| Q9Y4Y9 | U6 snRNA-associated Sm-like protein LSm5 | LSM5 | MAANATTNPS | - | - | - | - | - | - | - | - |
| Q9NRY2 | SOSS complex subunit C | INIP | MAANSSGQGF | 0 | 0 | 0 | 0 | - | - | 0 | 0 |
| Q9H9F9 | Actin-related protein 5 | ACTR5 | MAANVFPFRD | 0 | 0 | - | 0 | 0 | - | 0 | 0 |
| Q9BTV4 | Transmembrane protein 43 | TMEM43 | MAANYSSTST | - | 0 | 0 | 0 | - | - | - | - |
| Q9BZE9 | Tether containing UBX domain for GLUT4 | ASPSCR1 | MAAPAGGGGS | - | 0 | 0 | - | - | - | 0 | - |
| Q12809-2 | Isoform B of Potassium voltage-gated channel subfamily H member 2 | KCNH2 | MAAPAGKASR | 0 | 0 | 0 | 0 | 0 | 0 | 0 | 0 |
| P0C2W1 | F-box/SPRY domain-containing protein 1 | FBXO45 | MAAPAPGAGA | - | 0 | - | - | - | - | 0 | - |
| O95336 | 6-phosphogluconolactonase | PGLS | MAAPAPGLIS | 0 | 0 | - | - | 0 | 0 | 0 | 0 |
| Q7RTV5 | Thioredoxin-like protein AAED1 | AAED1 | MAAPAPVTRQ | 0 | 0 | 0 | 0 | - | - | 0 | 0 |
| Q9NXG2 | THUMP domain-containing protein 1 | THUMPD1 | MAAPAQQTTQ | - | - | 0 | 0 | - | - | - | - |
| Q8NBT0 | POC1 centriolar protein homolog A | POC1A | MAAPCAEDPS | - | 0 | 0 | 0 | 0 | 0 | 0 | 0 |
| Q96G03 | Phosphoglucomutase-2 | PGM2 | MAAPEGSGLG | - | - | - | - | - | - | - | - |
| Q16799 | Reticulon-1 | RTN1 | MAAPGDPQDE | 0 | 0 | 0 | 0 | 0 | 0 | 0 | - |
| Q9UBC2 | Epidermal growth factor receptor substrate 15-like 1 | EPS15L1 | MAAPLIPLSQ | 0 | 0 | 0 | - | 0 | 0 | 0 | - |
| Q96LJ7 | Dehydrogenase/reductase SDR family member 1 | DHRS1 | MAAPMNGQVC | - | - | 0 | 0 | 0 | 0 | 0 | 0 |
| Q04446 | 1,4-alpha-glucan-branching enzyme | GBE1 | MAAPMTPAAR | 0 | 0 | - | - | 0 | 0 | - | - |
| Q5RI15 | Cytochrome c oxidase protein 20 homolog | COX20 | MAAPPEPGEP | 0 | 0 | - | - | 0 | 0 | 0 | - |
| Q3MHD2 | Protein LSM12 homolog | LSM12 | MAAPPGEYFS | - | - | 0 | 0 | 0 | - | 0 | 0 |
| Q92979 | Ribosomal RNA small subunit methyltransferase NEP1 | EMG1 | MAAPSDGFKP | - | - | - | - | - | - | - | - |
| Q96MW1 | Coiled-coil domain-containing protein 43 | CCDC43 | MAAPSEVAAI | 0 | 0 | - | - | 0 | 0 | - | 0 |
| Q5JTJ3-3 | Isoform 3 of Cytochrome c oxidase assembly factor 6 homolog | COA6 | MAAPSMKERQ | 0 | 0 | 0 | 0 | - | - | 0 | 0 |
| P45985 | Dual specificity mitogen-activated protein kinase kinase 4 | MAP2K4 | MAAPSPSGGG | - | 0 | 0 | 0 | 0 | 0 | 0 | 0 |
| Q8N0U8 | Vitamin K epoxide reductase complex subunit 1-like protein 1 | VKORC1L1 | MAAPVLLRVS | - | 0 | 0 | 0 | 0 | 0 | 0 | 0 |
| Q13144 | Translation initiation factor eIF-2B subunit epsilon | EIF2B5 | MAAPVVAPPG | - | - | - | - | - | - | - | - |
| Q16401 | 26S proteasome non-ATPase regulatory subunit 5 | PSMD5 | MAAQALALLR | - | - | - | - | - | - | - | - |
| Q9H7Z6 | Histone acetyltransferase KAT8 | KAT8 | MAAQGAAAAV | 0 | 0 | 0 | 0 | 0 | 0 | 0 | - |
| P62826 | GTP-binding nuclear protein Ran | RAN | MAAQGEPQVQ | - | 0 | - | - | 0 | 0 | 0 | 0 |
| Q6NZ67 | Mitotic-spindle organizing protein 2B | MZT2B | MAAQGVGPGP | 0 | 0 | 0 | 0 | 0 | 0 | - | 0 |
| Q9UI14 | Prenylated Rab acceptor protein 1 | RABAC1 | MAAQKDQQKD | - | - | 0 | 0 | - | - | - | - |
| O14497 | AT-rich interactive domain-containing protein 1A | ARID1A | MAAQVAPAAA | 0 | 0 | - | - | 0 | 0 | - | - |
| Q9NUV9 | GTPase IMAP family member 4 | GIMAP4 | MAAQYGSMSF | 0 | 0 | 0 | 0 | - | - | 0 | 0 |
| Q9HCN4 | GPN-loop GTPase 1 | GPN1 | MAASAAAAEL | - | - | - | - | - | - | - | - |
| Q13263 | Transcription intermediary factor 1-beta | TRIM28 | MAASAAAASA | - | - | - | - | - | - | - | - |
| P23588 | Eukaryotic translation initiation factor 4B | EIF4B | MAASAKKKNK | - | 0 | 0 | 0 | 0 | 0 | 0 | 0 |
| Q5HYJ3 | Protein FAM76B | FAM76B | MAASALYACT | - | 0 | 0 | 0 | 0 | 0 | 0 | 0 |
| Q9Y4I1 | Unconventional myosin-Va | MYO5A | MAASELYTKF | - | - | 0 | 0 | - | - | - | 0 |
| Q8WVM0 | Dimethyladenosine transferase 1, mitochondrial | TFB1M | MAASGKLSTC | - | 0 | - | 0 | 0 | - | 0 | 0 |
| Q8IXQ5 | Kelch-like protein 7 | KLHL7 | MAASGVEKSS | - | 0 | - | 0 | - | 0 | - | 0 |
| Q14409 | Putative glycerol kinase 3 | GK3P | MAASKKAVLG | 0 | 0 | - | - | - | - | 0 | 0 |
| O94826 | Mitochondrial import receptor subunit TOM70 | TOMM70A | MAASKPVEAA | - | - | - | - | - | - | - | - |
| Q9NRM2 | Zinc finger protein 277 | ZNF277 | MAASKTQGAV | - | 0 | 0 | 0 | 0 | - | - | 0 |
| Q9P0J0 | NADH dehydrogenase [ubiquinone] 1 alpha subcomplex subunit 13 | NDUFA13 | MAASKVKQDM | - | - | - | - | - | - | - | - |
| O15160 | DNA-directed RNA polymerases I and III subunit RPAC1 | POLR1C | MAASQAVEEM | - | - | - | - | - | - | - | - |
| O14733 | Dual specificity mitogen-activated protein kinase kinase 7 | MAP2K7 | MAASSLEQKL | - | - | - | - | 0 | 0 | - | 0 |
| Q9NPJ6 | Mediator of RNA polymerase II transcription subunit 4 | MED4 | MAASSSGEKE | - | 0 | 0 | 0 | 0 | - | 0 | - |
| Q9BZJ0-2 | Isoform 2 of Crooked neck-like protein 1 | CRNKL1 | MAASTAAGKQ | - | - | 0 | 0 | - | - | - | - |
| Q9UJX2 | Cell division cycle protein 23 homolog | CDC23 | MAASTSMVPV | 0 | 0 | - | - | 0 | 0 | - | - |
| Q96IU4 | Alpha/beta hydrolase domain-containing protein 14B | ABHD14B | MAASVEQREG | - | - | 0 | 0 | 0 | - | 0 | 0 |
| Q9NWT6 | Hypoxia-inducible factor 1-alpha inhibitor | HIF1AN | MAATAAEAVA | - | - | - | - | - | - | - | - |
| Q8WUK0 | Phosphatidylglycerophosphatase and protein-tyrosine phosphatase 1 | PTPMT1 | MAATALLEAG | - | - | 0 | - | - | - | - | 0 |
| Q02978 | Mitochondrial 2-oxoglutarate/malate carrier protein | SLC25A11 | MAATASAGAG | - | - | - | - | - | - | - | - |
| O60518 | Ran-binding protein 6 | RANBP6 | MAATASAGVP | 0 | 0 | 0 | 0 | 0 | 0 | 0 | 0 |
| Q14141 | Septin-6 | sept-06 | MAATDIARQV | - | 0 | 0 | 0 | - | - | 0 | 0 |
| Q92599 | Septin-8 | sept-08 | MAATDLERFS | - | 0 | 0 | 0 | - | - | - | - |
| O95456 | Proteasome assembly chaperone 1 | PSMG1 | MAATFFGEVV | - | - | - | - | - | - | - | - |
| Q16831 | Uridine phosphorylase 1 | UPP1 | MAATGANAEK | 0 | - | 0 | 0 | 0 | 0 | - | - |
| Q9Y3X0 | Coiled-coil domain-containing protein 9 | CCDC9 | MAATLDLKSK | 0 | - | - | - | - | - | - | - |
| Q86TX2 | Acyl-coenzyme A thioesterase 1 | ACOT1 | MAATLILEPA | 0 | 0 | 0 | 0 | - | - | - | - |
| P28072 | Proteasome subunit beta type-6 | PSMB6 | MAATLLAARG | - | 0 | - | - | 0 | 0 | 0 | - |
| Q9NQP4 | Prefoldin subunit 4 | PFDN4 | MAATMKKAAA | - | 0 | 0 | 0 | 0 | 0 | - | 0 |
| Q13404 | Ubiquitin-conjugating enzyme E2 variant 1 | UBE2V1 | MAATTGSGVK | - | - | - | - | - | - | - | - |
| Q8IZD4 | mRNA-decapping enzyme 1B | DCP1B | MAAVAAGGLV | - | - | - | - | - | - | - | - |
| Q6Y1H2 | Very-long-chain (3R)-3-hydroxyacyl-CoA dehydratase 2 | PTPLB | MAAVAATAAA | - | - | 0 | 0 | - | - | - | - |
| Q9UI10 | Translation initiation factor eIF-2B subunit delta | EIF2B4 | MAAVAVAVRE | - | - | - | - | - | - | - | - |
| P17858 | ATP-dependent 6-phosphofructokinase, liver type | PFKL | MAAVDLEKLR | - | - | - | - | - | - | - | - |
| Q9H6E5 | Speckle targeted PIP5K1A-regulated poly(A) polymerase | TUT1 | MAAVDSDVES | - | 0 | - | - | - | - | - | 0 |
| Q3SXM5 | Inactive hydroxysteroid dehydrogenase-like protein 1 | HSDL1 | MAAVDSFYLL | - | - | - | - | 0 | - | - | - |
| Q8WU17 | E3 ubiquitin-protein ligase RNF139 | RNF139 | MAAVGPPQQQ | - | 0 | - | 0 | 0 | - | 0 | 0 |
| O43379 | WD repeat-containing protein 62 | WDR62 | MAAVGSGGYA | - | 0 | 0 | 0 | 0 | - | - | 0 |
| Q06187 | Tyrosine-protein kinase BTK | BTK | MAAVILESIF | 0 | 0 | - | - | 0 | 0 | 0 | 0 |
| P61758 | Prefoldin subunit 3 | VBP1 | MAAVKDSCGK | - | - | - | - | - | - | - | - |
| P40227 | T-complex protein 1 subunit zeta | CCT6A | MAAVKTLNPK | - | - | - | - | - | - | - | - |
| Q8IWY9 | Codanin-1 | CDAN1 | MAAVLESLLR | - | - | - | - | 0 | 0 | - | - |
| Q9BRP1 | Programmed cell death protein 2-like | PDCD2L | MAAVLKPVLL | - | - | - | - | - | - | - | 0 |
| Q9H3P7 | Golgi resident protein GCP60 | ACBD3 | MAAVLNAERL | - | 0 | - | - | - | - | - | - |
| P12270 | Nucleoprotein TPR | TPR | MAAVLQQVLE | - | - | - | - | - | - | - | - |
| Q9Y5X3 | Sorting nexin-5 | SNX5 | MAAVPELLQQ | - | - | - | - | - | - | - | - |
| P54132 | Bloom syndrome protein | BLM | MAAVPQNNLQ | 0 | 0 | - | 0 | 0 | 0 | 0 | 0 |
| Q15046 | Lysine--tRNA ligase | KARS | MAAVQAAEVK | - | - | - | - | - | - | - | - |
| Q9Y312 | Protein AAR2 homolog | AAR2 | MAAVQMDPEL | - | - | - | - | - | - | - | - |
| Q6AI08 | HEAT repeat-containing protein 6 | HEATR6 | MAAVQVVGSW | - | - | 0 | 0 | 0 | 0 | 0 | 0 |
| Q7Z406 | Myosin-14 | MYH14 | MAAVTMSVPG | - | - | 0 | 0 | 0 | 0 | 0 | 0 |
| Q9BV81 | ER membrane protein complex subunit 6 | EMC6 | MAAVVAKREG | - | - | - | - | - | - | - | - |
| Q92785 | Zinc finger protein ubi-d4 | DPF2 | MAAVVENVVK | 0 | 0 | - | - | 0 | 0 | 0 | 0 |
| Q9H920 | RING finger protein 121 | RNF121 | MAAVVEVEVG | 0 | 0 | 0 | 0 | 0 | - | - | - |
| Q9H1Z4 | WD repeat-containing protein 13 | WDR13 | MAAVWQQVLA | 0 | 0 | 0 | 0 | 0 | 0 | - | 0 |
| P18669 | Phosphoglycerate mutase 1 | PGAM1 | MAAYKLVLIR | - | - | - | - | - | 0 | - | - |
| Q9H270 | Vacuolar protein sorting-associated protein 11 homolog | VPS11 | MAAYLQWRRF | 0 | 0 | - | - | 0 | - | 0 | 0 |
| P09382 | Galectin-1 | LGALS1 | MACGLVASNL | - | - | - | - | - | - | - | - |
| Q8IUR7 | Armadillo repeat-containing protein 8 | ARMC8 | MACLLETPIR | - | 0 | - | 0 | - | - | - | 0 |
| P06703 | Protein S100-A6 | S100A6 | MACPLDQAIG | 0 | - | 0 | 0 | 0 | 0 | 0 | 0 |
| Q93100-2 | Isoform 2 of Phosphorylase b kinase regulatory subunit beta | PHKB | MACSPDAVVS | 0 | 0 | 0 | 0 | 0 | 0 | 0 | 0 |
| Q7LBC6 | Lysine-specific demethylase 3B | KDM3B | MADAAASPVG | - | - | - | - | - | - | - | - |
| Q7Z7C8-2 | Isoform 2 of Transcription initiation factor TFIID subunit 8 | TAF8 | MADAAATAGA | - | - | - | - | - | - | - | 0 |
| Q96C86 | m7GpppX diphosphatase | DCPS | MADAAPQLGK | - | - | - | - | - | 0 | - | 0 |
| O94874 | E3 UFM1-protein ligase 1 | UFL1 | MADAWEEIRR | - | - | - | 0 | 0 | - | 0 | 0 |
| P15880 | 40S ribosomal protein S2 | RPS2 | MADDAGAAGG | - | 0 | - | - | - | - | 0 | 0 |
| Q96GQ5 | UPF0420 protein C16orf58 | C16orf58 | MADDAGLETP | 0 | 0 | 0 | 0 | 0 | 0 | - | 0 |
| Q14498 | RNA-binding protein 39 | RBM39 | MADDIDIEAM | 0 | 0 | - | 0 | 0 | 0 | 0 | 0 |
| Q6IS14 | Eukaryotic translation initiation factor 5A-1-like | EIF5AL1 | MADDLDFETG | - | 0 | - | - | - | - | - | 0 |
| Q9UHA4 | Ragulator complex protein LAMTOR3 | LAMTOR3 | MADDLKRFLY | - | 0 | 0 | 0 | - | - | - | - |
| P62495 | Eukaryotic peptide chain release factor subunit 1 | ETF1 | MADDPSAADR | - | 0 | 0 | 0 | 0 | - | 0 | - |
| P62310 | U6 snRNA-associated Sm-like protein LSm3 | LSM3 | MADDVDQQQT | - | - | - | - | - | - | - | - |
| Q96EB6 | NAD-dependent protein deacetylase sirtuin-1 | SIRT1 | MADEAALALQ | - | - | - | - | 0 | 0 | - | - |
| P53814 | Smoothelin | SMTN | MADEALAGLD | - | 0 | - | 0 | - | - | 0 | 0 |
| O00418 | Eukaryotic elongation factor 2 kinase | EEF2K | MADEDLIFRL | - | 0 | - | 0 | - | - | 0 | 0 |
| Q13526 | Peptidyl-prolyl cis-trans isomerase NIMA-interacting 1 | PIN1 | MADEEKLPPG | - | 0 | 0 | 0 | 0 | 0 | 0 | 0 |
| O14737 | Programmed cell death protein 5 | PDCD5 | MADEELEALR | - | 0 | 0 | 0 | - | - | - | 0 |
| P49773 | Histidine triad nucleotide-binding protein 1 | HINT1 | MADEIAKAQV | - | - | - | - | - | - | - | - |
| Q03135-2 | Isoform 2 of Caveolin-1 | CAV1 | MADELSEKQV | - | - | 0 | 0 | - | - | 0 | - |
| Q8IXW5 | Putative RNA polymerase II subunit B1 CTD phosphatase RPAP2 | RPAP2 | MADFAGPSSA | 0 | 0 | - | 0 | 0 | 0 | 0 | 0 |
| Q15056 | Eukaryotic translation initiation factor 4H | EIF4H | MADFDTYDDR | - | - | - | - | - | - | - | - |
| Q9BW61 | DET1- and DDB1-associated protein 1 | DDA1 | MADFLKGLPV | - | 0 | 0 | 0 | 0 | 0 | - | 0 |
| Q14344 | Guanine nucleotide-binding protein subunit alpha-13 | GNA13 | MADFLPSRSV | - | 0 | 0 | 0 | 0 | 0 | 0 | 0 |
| Q8TBC4 | NEDD8-activating enzyme E1 catalytic subunit | UBA3 | MADGEEPEKK | - | 0 | 0 | 0 | 0 | 0 | 0 | - |
| P62140 | Serine/threonine-protein phosphatase PP1-beta catalytic subunit | PPP1CB | MADGELNVDS | - | - | - | - | - | - | - | - |
| P14859-5 | Isoform 5 of POU domain, class 2, transcription factor 1 | POU2F1 | MADGGAASQD | - | - | - | - | 0 | - | - | 0 |
| Q9H330 | Transmembrane protein 245 | TMEM245 | MADGGGPKDA | - | - | 0 | 0 | - | - | 0 | 0 |
| O60504-2 | Isoform Beta of Vinexin | SORBS3 | MADGGSPFLG | - | - | 0 | 0 | - | - | - | - |
| Q8WW12 | PEST proteolytic signal-containing nuclear protein | PCNP | MADGKAGDEK | - | - | - | - | - | - | - | - |
| Q9Y285 | Phenylalanine--tRNA ligase alpha subunit | FARSA | MADGQVAELL | - | - | - | - | - | - | - | - |
| Q8NEM2 | SHC SH2 domain-binding protein 1 | SHCBP1 | MADGSLTGGG | - | - | - | 0 | - | - | - | - |
| Q99733 | Nucleosome assembly protein 1-like 4 | NAP1L4 | MADHSFSDGV | - | - | - | - | 0 | 0 | 0 | - |
| P55209 | Nucleosome assembly protein 1-like 1 | NAP1L1 | MADIDNKEQS | 0 | 0 | - | - | 0 | 0 | - | 0 |
| O00170 | AH receptor-interacting protein | AIP | MADIIARLRE | - | 0 | - | - | - | - | - | - |
| P49418 | Amphiphysin | AMPH | MADIKTGIFA | 0 | 0 | 0 | 0 | 0 | 0 | - | - |
| P62280 | 40S ribosomal protein S11 | RPS11 | MADIQTERAY | - | - | 0 | - | - | - | - | - |
| Q9BY77 | Polymerase delta-interacting protein 3 | POLDIP3 | MADISLDELI | 0 | 0 | 0 | 0 | - | 0 | 0 | 0 |
| O94811 | Tubulin polymerization-promoting protein | TPPP | MADKAKPAKA | - | 0 | 0 | 0 | 0 | 0 | 0 | 0 |
| Q09028 | Histone-binding protein RBBP4 | RBBP4 | MADKEAAFDD | - | - | - | - | - | - | - | - |
| Q09028-2 | Isoform 2 of Histone-binding protein RBBP4 | RBBP4 | MADKEAFDDA | - | - | - | - | 0 | - | 0 | 0 |
| O43301 | Heat shock 70 kDa protein 12A | HSPA12A | MADKEAGGSD | - | 0 | 0 | 0 | 0 | 0 | 0 | - |
| Q9UK45 | U6 snRNA-associated Sm-like protein LSm7 | LSM7 | MADKEKKKKE | - | 0 | - | - | 0 | - | - | 0 |
| Q86V81 | THO complex subunit 4 | ALYREF | MADKMDMSLD | 0 | - | - | - | - | 0 | - | 0 |
| P63313 | Thymosin beta-10 | TMSB10 | MADKPDMGEI | 0 | 0 | 0 | 0 | 0 | 0 | - | - |
| Q9H936 | Mitochondrial glutamate carrier 1 | SLC25A22 | MADKQISLPA | 0 | 0 | 0 | 0 | 0 | 0 | 0 | 0 |
| O15042 | U2 snRNP-associated SURP motif-containing protein | U2SURP | MADKTPGGSQ | - | - | - | - | - | - | - | - |
| Q5EG05 | Caspase recruitment domain-containing protein 16 | CARD16 | MADKVLKEKR | 0 | 0 | 0 | 0 | - | - | - | - |
| P33176 | Kinesin-1 heavy chain | KIF5B | MADLAECNIK | - | - | - | - | - | - | - | - |
| Q9H6Z4-3 | Isoform 3 of Ran-binding protein 3 | RANBP3 | MADLANEEKP | - | 0 | - | - | - | 0 | - | - |
| P36873 | Serine/threonine-protein phosphatase PP1-gamma catalytic subunit | PPP1CC | MADLDKLNID | - | - | - | - | - | - | - | - |
| Q9H9E3 | Conserved oligomeric Golgi complex subunit 4 | COG4 | MADLDSPPKL | - | 0 | 0 | 0 | - | 0 | 0 | 0 |
| P25098 | Beta-adrenergic receptor kinase 1 | ADRBK1 | MADLEAVLAD | 0 | 0 | - | 0 | 0 | 0 | 0 | 0 |
| P47755 | F-actin-capping protein subunit alpha-2 | CAPZA2 | MADLEEQLSD | - | - | - | - | - | - | - | - |
| O75391 | Sperm-associated antigen 7 | SPAG7 | MADLLGSILS | 0 | 0 | - | - | - | - | - | 0 |
| P27816 | Microtubule-associated protein 4 | MAP4 | MADLSLADAL | - | - | - | - | - | - | - | - |
| Q01518 | Adenylyl cyclase-associated protein 1 | CAP1 | MADMQNLVER | - | - | - | - | - | - | - | - |
| O00629 | Importin subunit alpha-3 | KPNA4 | MADNEKLDNQ | - | - | 0 | 0 | - | - | - | - |
| P05771 | Protein kinase C beta type | PRKCB | MADPAAGPPP | 0 | 0 | - | - | 0 | 0 | 0 | 0 |
| P29083 | General transcription factor IIE subunit 1 | GTF2E1 | MADPDVLTEV | - | - | - | - | - | - | - | 0 |
| Q13561 | Dynactin subunit 2 | DCTN2 | MADPKYADLP | - | - | - | - | - | - | - | - |
| P29218 | Inositol monophosphatase 1 | IMPA1 | MADPWQECMD | 0 | 0 | - | 0 | 0 | 0 | 0 | 0 |
| P62913-2 | Isoform 2 of 60S ribosomal protein L11 | RPL11 | MADQGEKENP | - | - | - | - | +/- | +/- | +/- | +/- |
| P62158 | Calmodulin | CALM1 | MADQLTEEQI | 0 | 0 | 0 | 0 | 0 | 0 | 0 | 0 |
| P07741 | Adenine phosphoribosyltransferase | APRT | MADSELQLVE | - | - | - | - | - | - | - | - |
| Q5SY16 | -hydroxyl-kinase NOL9 | NOL9 | MADSGLLLKR | 0 | 0 | - | - | 0 | 0 | 0 | 0 |
| Q96C90 | Protein phosphatase 1 regulatory subunit 14B | PPP1R14B | MADSGTAGGA | - | - | - | - | - | - | - | - |
| Q92917 | G patch domain and KOW motifs-containing protein | GPKOW | MADSKEGVLP | 0 | 0 | - | - | 0 | - | 0 | 0 |
| P49662-2 | Isoform 2 of Caspase-4 | CASP4 | MADSMQEKQR | - | 0 | 0 | 0 | 0 | - | 0 | 0 |
| P49589-3 | Isoform 3 of Cysteine--tRNA ligase, cytoplasmic | CARS | MADSSGQQAP | - | 0 | 0 | 0 | 0 | - | - | - |
| P49589 | Cysteine--tRNA ligase, cytoplasmic | CARS | MADSSGQQGK | - | - | 0 | 0 | - | - | 0 | 0 |
| Q8IV50 | LysM and putative peptidoglycan-binding domain-containing protein 2 | LYSMD2 | MADSSPALSL | - | 0 | - | - | - | - | 0 | 0 |
| O14681 | Etoposide-induced protein 2.4 homolog | EI24 | MADSVKTFLQ | - | - | - | - | - | - | - | - |
| Q9H867 | Protein-lysine methyltransferase METTL21D | VCPKMT | MADTLESSLE | - | - | 0 | 0 | 0 | 0 | 0 | 0 |
| Q9NY33 | Dipeptidyl peptidase 3 | DPP3 | MADTQYILPN | - | - | - | - | - | - | - | - |
| P17252 | Protein kinase C alpha type | PRKCA | MADVFPGNDS | 0 | 0 | - | - | - | - | - | - |
| Q6P1J9 | Parafibromin | CDC73 | MADVLSVLRQ | - | - | - | - | - | - | - | - |
| Q9NRG0 | Chromatin accessibility complex protein 1 | CHRAC1 | MADVVVGKDK | - | - | - | - | - | - | - | - |
| Q96AE4 | Far upstream element-binding protein 1 | FUBP1 | MADYSTVPPP | 0 | 0 | - | - | 0 | 0 | - | - |
| O00116 | Alkyldihydroxyacetonephosphate synthase, peroxisomal | AGPS | MAEAAAAAGG | 0 | 0 | - | - | 0 | 0 | 0 | 0 |
| Q96JB2 | Conserved oligomeric Golgi complex subunit 3 | COG3 | MAEAALLLLP | - | - | - | - | - | - | - | - |
| Q9HCE9 | Anoctamin-8 | ANO8 | MAEAASGAGG | - | 0 | 0 | 0 | 0 | 0 | 0 | - |
| Q96EC8 | Protein YIPF6 | YIPF6 | MAEAEESPGD | - | 0 | 0 | 0 | 0 | - | 0 | - |
| Q86VR2 | Protein FAM134C | FAM134C | MAEAEGVPTT | 0 | 0 | - | - | 0 | - | 0 | - |
| O60927 | Protein phosphatase 1 regulatory subunit 11 | PPP1R11 | MAEAGAGLSE | 0 | 0 | 0 | 0 | - | - | - | - |
| Q96P48 | Arf-GAP with Rho-GAP domain, ANK repeat and PH domain-containing protein 1 | ARAP1 | MAEAGDAALS | - | 0 | - | - | 0 | - | 0 | 0 |
| Q16254 | Transcription factor E2F4 | E2F4 | MAEAGPQAPP | - | 0 | - | - | 0 | - | - | - |
| P50416 | Carnitine O-palmitoyltransferase 1, liver isoform | CPT1A | MAEAHQAVAF | 0 | - | 0 | 0 | 0 | 0 | 0 | 0 |
| Q9NYB0 | Telomeric repeat-binding factor 2-interacting protein 1 | TERF2IP | MAEAMDLGKD | - | 0 | 0 | 0 | 0 | 0 | 0 | 0 |
| Q16512-2 | Isoform 2 of Serine/threonine-protein kinase N1 | PKN1 | MAEANNPSEQ | 0 | 0 | - | - | 0 | - | 0 | 0 |
| Q6P1K2 | Polyamine-modulated factor 1 | PMF1 | MAEASSANLG | - | 0 | - | 0 | - | 0 | 0 | 0 |
| Q9HCE0 | Ectopic P granules protein 5 homolog | EPG5 | MAEAVKPQRR | - | 0 | 0 | 0 | 0 | - | 0 | - |
| O95571 | Persulfide dioxygenase ETHE1, mitochondrial | ETHE1 | MAEAVLRVAR | - | 0 | 0 | 0 | 0 | 0 | 0 | 0 |
| P48382 | DNA-binding protein RFX5 | RFX5 | MAEDEPDAKS | - | 0 | 0 | 0 | 0 | 0 | 0 | - |
| P14854 | Cytochrome c oxidase subunit 6B1 | COX6B1 | MAEDMETKIK | - | 0 | - | - | - | - | - | - |
| P54274 | Telomeric repeat-binding factor 1 | TERF1 | MAEDVSSAAP | - | 0 | - | 0 | 0 | 0 | 0 | 0 |
| Q7Z388 | Probable C-mannosyltransferase DPY19L4 | DPY19L4 | MAEEEGPPVE | - | 0 | 0 | 0 | - | - | 0 | 0 |
| Q9P2R3 | Rabankyrin-5 | ANKFY1 | MAEEEVAKLE | - | 0 | 0 | 0 | 0 | 0 | 0 | 0 |
| P25398 | 40S ribosomal protein S12 | RPS12 | MAEEGIAAGG | - | 0 | - | - | 0 | 0 | - | - |
| P21980 | Protein-glutamine gamma-glutamyltransferase 2 | TGM2 | MAEELVLERC | - | - | - | - | - | - | - | - |
| O00299 | Chloride intracellular channel protein 1 | CLIC1 | MAEEQPQVEL | 0 | 0 | - | - | 0 | 0 | - | 0 |
| O14545 | TRAF-type zinc finger domain-containing protein 1 | TRAFD1 | MAEFLDDQET | - | 0 | - | - | - | 0 | - | 0 |
| Q9Y342 | Plasmolipin | PLLP | MAEFPSKVST | - | 0 | 0 | 0 | - | 0 | 0 | 0 |
| Q86V48 | Leucine zipper protein 1 | LUZP1 | MAEFTSYKET | 0 | 0 | 0 | 0 | - | - | 0 | 0 |
| Q9Y4E8 | Ubiquitin carboxyl-terminal hydrolase 15 | USP15 | MAEGGAADLD | - | - | - | - | - | - | - | - |
| Q6ZN17 | Protein lin-28 homolog B | LIN28B | MAEGGASKGG | 0 | 0 | - | - | 0 | 0 | 0 | 0 |
| Q9Y2V7 | Conserved oligomeric Golgi complex subunit 6 | COG6 | MAEGSGEVVA | 0 | 0 | 0 | 0 | 0 | 0 | - | 0 |
| Q96G46-2 | Isoform 2 of tRNA-dihydrouridine(47) synthase [NAD(P)(+)]-like | DUS3L | MAEGTAEAPL | 0 | 0 | 0 | 0 | - | - | - | 0 |
| Q9NYH9 | U3 small nucleolar RNA-associated protein 6 homolog | UTP6 | MAEIIQERIE | 0 | 0 | - | - | - | - | 0 | 0 |
| P60510 | Serine/threonine-protein phosphatase 4 catalytic subunit | PPP4C | MAEISDLDRQ | - | - | - | - | - | - | - | - |
| Q9GZY8-2 | Isoform 2 of Mitochondrial fission factor | MFF | MAEISRIQYE | 0 | 0 | 0 | 0 | 0 | 0 | 0 | 0 |
| O00399 | Dynactin subunit 6 | DCTN6 | MAEKTQKSVK | 0 | 0 | 0 | 0 | 0 | 0 | 0 | 0 |
| O95361 | Tripartite motif-containing protein 16 | TRIM16 | MAELDLMAPG | - | - | 0 | 0 | - | - | - | - |
| Q9Y5J9 | Mitochondrial import inner membrane translocase subunit Tim8 B | TIMM8B | MAELGEADEA | - | - | - | - | - | - | - | 0 |
| O15212 | Prefoldin subunit 6 | PFDN6 | MAELIQKKLQ | 0 | 0 | - | 0 | 0 | 0 | 0 | 0 |
| Q99941 | Cyclic AMP-dependent transcription factor ATF-6 beta | ATF6B | MAELMLLSEI | 0 | 0 | 0 | - | 0 | - | 0 | 0 |
| Q9UN36 | Protein NDRG2 | NDRG2 | MAELQEVQIT | 0 | 0 | - | - | 0 | 0 | 0 | 0 |
| Q8IZP0 | Abl interactor 1 | ABI1 | MAELQMLLEE | - | 0 | - | 0 | 0 | - | - | - |
| Q96C01 | Protein FAM136A | FAM136A | MAELQQLRVQ | - | 0 | - | - | - | - | - | 0 |
| P45974 | Ubiquitin carboxyl-terminal hydrolase 5 | USP5 | MAELSEEALL | - | - | - | - | - | - | - | - |
| Q04323 | UBX domain-containing protein 1 | UBXN1 | MAELTALESL | - | 0 | - | - | - | - | - | 0 |
| P51114 | Fragile X mental retardation syndrome-related protein 1 | FXR1 | MAELTVEVRG | 0 | - | 0 | 0 | 0 | 0 | 0 | 0 |
| Q96I24 | Far upstream element-binding protein 3 | FUBP3 | MAELVQGQSA | - | - | 0 | 0 | - | 0 | 0 | 0 |
| Q9HAB8 | Phosphopantothenate--cysteine ligase | PPCS | MAEMDPVAEF | - | 0 | 0 | 0 | - | 0 | 0 | 0 |
| O00499 | Myc box-dependent-interacting protein 1 | BIN1 | MAEMGSKGVT | - | - | 0 | 0 | - | - | - | - |
| P05455 | Lupus La protein | SSB | MAENGDNEKM | 0 | 0 | - | - | 0 | 0 | 0 | 0 |
| Q9UHD9 | Ubiquilin-2 | UBQLN2 | MAENGESSGP | 0 | 0 | 0 | 0 | 0 | 0 | 0 | 0 |
| Q96GC9 | Vacuole membrane protein 1 | VMP1 | MAENGKNCDQ | - | 0 | 0 | 0 | 0 | - | 0 | - |
| Q8NHP6 | Motile sperm domain-containing protein 2 | MOSPD2 | MAENHAQNKA | 0 | 0 | 0 | 0 | 0 | 0 | - | 0 |
| Q92793 | CREB-binding protein | CREBBP | MAENLLDGPP | - | - | 0 | 0 | - | 0 | 0 | 0 |
| O00505 | Importin subunit alpha-4 | KPNA3 | MAENPSLENH | 0 | 0 | 0 | 0 | 0 | 0 | 0 | 0 |
| Q9H8V3 | Protein ECT2 | ECT2 | MAENSVLTST | - | 0 | - | - | - | - | 0 | 0 |
| Q9NQT5 | Exosome complex component RRP40 | EXOSC3 | MAEPASVAAE | - | - | - | - | - | - | - | - |
| Q9Y5Z4 | Heme-binding protein 2 | HEBP2 | MAEPLQPDPG | 0 | 0 | - | 0 | 0 | 0 | 0 | 0 |
| Q04760 | Lactoylglutathione lyase | GLO1 | MAEPQPPSGG | - | 0 | 0 | 0 | 0 | 0 | 0 | 0 |
| Q92830 | Histone acetyltransferase KAT2A | KAT2A | MAEPSQAPTP | - | 0 | - | - | 0 | - | 0 | 0 |
| Q96H79 | Zinc finger CCCH-type antiviral protein 1-like | ZC3HAV1L | MAEPTVCSFL | - | 0 | 0 | 0 | 0 | 0 | 0 | 0 |
| P52565 | Rho GDP-dissociation inhibitor 1 | ARHGDIA | MAEQEPTAEQ | 0 | 0 | 0 | 0 | 0 | 0 | 0 | - |
| O15541 | RING finger protein 113A | RNF113A | MAEQLSPGKA | 0 | 0 | - | - | 0 | 0 | 0 | 0 |
| P11413 | Glucose-6-phosphate 1-dehydrogenase | G6PD | MAEQVALSRT | - | - | - | - | - | - | - | - |
| Q9NRF9 | DNA polymerase epsilon subunit 3 | POLE3 | MAERPEDLNL | - | - | 0 | 0 | 0 | 0 | - | - |
| O60869 | Endothelial differentiation-related factor 1 | EDF1 | MAESDWDTVT | - | - | - | - | - | - | - | 0 |
| Q8TAT6 | Nuclear protein localization protein 4 homolog | NPLOC4 | MAESIIIRVQ | - | - | - | - | - | - | - | - |
| P09874 | Poly [ADP-ribose] polymerase 1 | PARP1 | MAESSDKLYR | - | - | - | - | - | - | - | - |
| P49736 | DNA replication licensing factor MCM2 | MCM2 | MAESSESFTM | - | - | - | - | - | - | - | - |
| Q12788 | Transducin beta-like protein 3 | TBL3 | MAETAAGVGR | - | 0 | 0 | 0 | - | - | 0 | 0 |
| Q9BUP3 | Oxidoreductase HTATIP2 | HTATIP2 | MAETEALSKL | - | - | - | - | - | - | 0 | 0 |
| Q08945 | FACT complex subunit SSRP1 | SSRP1 | MAETLEFNDV | - | 0 | - | - | - | - | 0 | - |
| Q14151 | Scaffold attachment factor B2 | SAFB2 | MAETLPGSGD | 0 | 0 | - | - | - | - | 0 | 0 |
| Q15424 | Scaffold attachment factor B1 | SAFB | MAETLSGLGD | - | 0 | - | - | - | - | - | - |
| O60493 | Sorting nexin-3 | SNX3 | MAETVADTRR | 0 | 0 | 0 | 0 | - | - | - | - |
| Q8TF09 | Dynein light chain roadblock-type 2 | DYNLRB2 | MAEVEETLKR | - | - | - | - | - | - | - | - |
| P62841 | 40S ribosomal protein S15 | RPS15 | MAEVEQKKKR | - | - | - | - | - | - | - | - |
| P85037 | Forkhead box protein K1 | FOXK1 | MAEVGEDSGA | - | 0 | 0 | 0 | 0 | 0 | 0 | - |
| Q92993 | Histone acetyltransferase KAT5 | KAT5 | MAEVGEIIEG | - | 0 | 0 | 0 | 0 | 0 | 0 | 0 |
| Q9H1D9 | DNA-directed RNA polymerase III subunit RPC6 | POLR3F | MAEVKVKVQP | - | 0 | - | 0 | - | 0 | 0 | 0 |
| P40429 | 60S ribosomal protein L13a | RPL13A | MAEVQVLVLD | - | - | - | - | - | - | - | - |
| Q8IVM0 | Coiled-coil domain-containing protein 50 | CCDC50 | MAEVSIDQSK | 0 | - | 0 | - | - | - | - | - |
| P60228 | Eukaryotic translation initiation factor 3 subunit E | EIF3E | MAEYDLTTRI | - | - | - | - | - | - | - | - |
| O43175 | D-3-phosphoglycerate dehydrogenase | PHGDH | MAFANLRKVL | - | - | - | - | - | - | - | - |
| P60866 | 40S ribosomal protein S20 | RPS20 | MAFKDTGKTP | - | - | 0 | - | - | 0 | - | - |
| Q9Y6M9 | NADH dehydrogenase [ubiquinone] 1 beta subcomplex subunit 9 | NDUFB9 | MAFLASGPYL | 0 | 0 | 0 | 0 | - | 0 | 0 | 0 |
| O15084 | Serine/threonine-protein phosphatase 6 regulatory ankyrin repeat subunit A | ANKRD28 | MAFLKLRDQP | - | - | - | - | 0 | - | - | 0 |
| P43304 | Glycerol-3-phosphate dehydrogenase, mitochondrial | GPD2 | MAFQKAVKGT | 0 | 0 | - | 0 | 0 | 0 | 0 | 0 |
| A1L3X0 | Elongation of very long chain fatty acids protein 7 | ELOVL7 | MAFSDLTSRT | - | - | - | - | 0 | 0 | 0 | 0 |
| O43426 | Synaptojanin-1 | SYNJ1 | MAFSKGFRIY | 0 | 0 | 0 | 0 | - | - | 0 | 0 |
| Q86XN6 | Zinc finger protein 761 | ZNF761 | MAFSQGLLTF | 0 | - | 0 | 0 | 0 | 0 | 0 | 0 |
| A6ND36 | Protein FAM83G | FAM83G | MAFSQVQCLD | 0 | 0 | 0 | 0 | 0 | 0 | 0 | 0 |
| Q9Y6X9 | MORC family CW-type zinc finger protein 2 | MORC2 | MAFTNYSSLN | - | - | - | - | 0 | - | 0 | 0 |
| Q96CS7 | Pleckstrin homology domain-containing family B member 2 | PLEKHB2 | MAFVKSGWLL | 0 | - | - | - | 0 | 0 | - | 0 |
| Q9BXR0 | Queuine tRNA-ribosyltransferase | QTRT1 | MAGAATQASL | - | - | - | - | - | - | - | - |
| Q9BRA0 | N-alpha-acetyltransferase 38, NatC auxiliary subunit | NAA38 | MAGAGPTMLL | 0 | 0 | 0 | 0 | - | 0 | 0 | 0 |
| O95295 | SNARE-associated protein Snapin | SNAPIN | MAGAGSAAVS | - | - | - | - | - | - | - | - |
| P35240 | Merlin | NF2 | MAGAIASRMS | - | 0 | 0 | 0 | 0 | - | 0 | 0 |
| Q9NP71 | Carbohydrate-responsive element-binding protein | MLXIPL | MAGALAGLAA | - | - | 0 | - | 0 | 0 | 0 | 0 |
| Q9Y5U8 | Mitochondrial pyruvate carrier 1 | MPC1 | MAGALVRKAA | 0 | 0 | 0 | 0 | - | - | 0 | - |
| Q8NFU3 | Thiosulfate sulfurtransferase/rhodanese-like domain-containing protein 1 | TSTD1 | MAGAPTVSLP | - | - | 0 | 0 | 0 | 0 | 0 | 0 |
| Q01970 | 1-phosphatidylinositol 4,5-bisphosphate phosphodiesterase beta-3 | PLCB3 | MAGAQPGVHA | - | 0 | 0 | 0 | 0 | 0 | 0 | 0 |
| P30154 | Serine/threonine-protein phosphatase 2A 65 kDa regulatory subunit A beta isoform | PPP2R1B | MAGASELGTG | 0 | 0 | - | - | 0 | 0 | - | 0 |
| Q02878 | 60S ribosomal protein L6 | RPL6 | MAGEKVEKPD | - | 0 | 0 | 0 | 0 | 0 | 0 | 0 |
| Q9BUQ8 | Probable ATP-dependent RNA helicase DDX23 | DDX23 | MAGELADKKD | - | - | - | - | - | - | - | - |
| P56557 | Transmembrane protein 50B | TMEM50B | MAGFLDNFRW | - | - | - | - | - | - | 0 | 0 |
| Q13637 | Ras-related protein Rab-32 | RAB32 | MAGGGAGDPG | 0 | 0 | 0 | 0 | - | - | - | - |
| P23497 | Nuclear autoantigen Sp-100 | SP100 | MAGGGGDLST | - | 0 | 0 | 0 | - | - | 0 | 0 |
| P0C0S5/Q71UI9 | Histone H2A.Z or H2V | H2AFZ/H2AFV | MAGGKAGKDS | - | - | - | - | - | - | - | - |
| P17655 | Calpain-2 catalytic subunit | CAPN2 | MAGIAAKLAK | - | - | - | - | - | - | - | - |
| O95183 | Vesicle-associated membrane protein 5 | VAMP5 | MAGIELERCQ | 0 | 0 | 0 | 0 | - | - | 0 | 0 |
| A0JNW5 | UHRF1-binding protein 1-like | UHRF1BP1L | MAGIIKKQIL | - | 0 | 0 | 0 | 0 | 0 | 0 | 0 |
| P52434 | DNA-directed RNA polymerases I, II, and III subunit RPABC3 | POLR2H | MAGILFEDIF | 0 | 0 | - | - | 0 | 0 | 0 | 0 |
| P06753-2 | Isoform 2 of Tropomyosin alpha-3 chain | TPM3 | MAGITTIEAV | - | - | - | - | - | - | - | - |
| P16083 | Ribosyldihydronicotinamide dehydrogenase [quinone] | NQO2 | MAGKKVLIVY | 0 | 0 | - | - | 0 | 0 | 0 | 0 |
| P14927 | Cytochrome b-c1 complex subunit 7 | UQCRB | MAGKQAVSAS | - | 0 | - | - | 0 | - | - | - |
| P51151 | Ras-related protein Rab-9A | RAB9A | MAGKSSLFKV | 0 | 0 | 0 | 0 | 0 | - | 0 | 0 |
| Q9NPD3 | Exosome complex component RRP41 | EXOSC4 | MAGLELLSDQ | - | - | - | - | - | - | - | - |
| Q92530 | Proteasome inhibitor PI31 subunit | PSMF1 | MAGLEVLFAS | - | 0 | - | - | 0 | - | - | 0 |
| Q96KR6 | Protein FAM210B | FAM210B | MAGLLALLGP | 0 | 0 | - | - | 0 | 0 | 0 | 0 |
| P67936 | Tropomyosin alpha-4 chain | TPM4 | MAGLNSLEAV | - | - | - | - | - | - | - | - |
| P61088 | Ubiquitin-conjugating enzyme E2 N | UBE2N | MAGLPRRIIK | 0 | 0 | 0 | 0 | - | 0 | - | 0 |
| Q8NC56 | LEM domain-containing protein 2 | LEMD2 | MAGLSDLELR | - | 0 | - | 0 | - | - | 0 | 0 |
| O75935 | Dynactin subunit 3 | DCTN3 | MAGLTDLQRL | 0 | 0 | - | 0 | - | - | - | 0 |
| Q9NW13 | RNA-binding protein 28 | RBM28 | MAGLTLFVGR | - | - | - | - | - | 0 | - | - |
| Q86UX7 | Fermitin family homolog 3 | FERMT3 | MAGMKTASGD | 0 | 0 | - | - | 0 | 0 | 0 | 0 |
| Q9Y606-2 | Isoform 2 of tRNA pseudouridine synthase A, mitochondrial | PUS1 | MAGNAEPPPA | - | 0 | - | - | 0 | - | - | 0 |
| P46108 | Adapter molecule crk | CRK | MAGNFDSEER | - | - | 0 | 0 | - | - | - | - |
| Q9Y4E6 | WD repeat-containing protein 7 | WDR7 | MAGNSLVLPI | - | - | - | 0 | 0 | - | 0 | 0 |
| Q8N2U0 | Transmembrane protein 256 | TMEM256 | MAGPAAAFRR | - | - | - | - | - | - | - | - |
| Q9BUP3-3 | Isoform 3 of Oxidoreductase HTATIP2 | HTATIP2 | MAGPAALSAA | 0 | - | - | - | 0 | 0 | 0 | 0 |
| P50213 | Isocitrate dehydrogenase [NAD] subunit alpha, mitochondrial | IDH3A | MAGPAWISKV | - | - | - | 0 | 0 | 0 | 0 | 0 |
| O00459 | Phosphatidylinositol 3-kinase regulatory subunit beta | PIK3R2 | MAGPEGFQYR | - | 0 | - | 0 | 0 | 0 | 0 | 0 |
| Q9P0I2 | ER membrane protein complex subunit 3 | EMC3 | MAGPELLLDS | - | - | - | - | - | - | - | - |
| Q96IX5 | Up-regulated during skeletal muscle growth protein 5 | USMG5 | MAGPESDAQY | 0 | 0 | - | 0 | 0 | 0 | 0 | 0 |
| Q5T447 | E3 ubiquitin-protein ligase HECTD3 | HECTD3 | MAGPGPGAVL | - | 0 | - | 0 | - | - | 0 | 0 |
| Q8N653 | Leucine-zipper-like transcriptional regulator 1 | LZTR1 | MAGPGSTGGQ | - | - | - | 0 | - | - | - | - |
| Q9P015 | 39S ribosomal protein L15, mitochondrial | MRPL15 | MAGPLQGGGA | - | 0 | 0 | 0 | - | - | - | 0 |
| Q9H633 | Ribonuclease P protein subunit p21 | RPP21 | MAGPVKDREA | - | 0 | 0 | 0 | 0 | - | 0 | 0 |
| P85298 | Rho GTPase-activating protein 8 | ARHGAP8 | MAGQDPALST | - | 0 | 0 | 0 | 0 | 0 | 0 | 0 |
| Q9H2V7 | Protein spinster homolog 1 | SPNS1 | MAGSDTAPFL | 0 | 0 | - | 0 | 0 | 0 | 0 | 0 |
| P78527 | DNA-dependent protein kinase catalytic subunit | PRKDC | MAGSGAGVRC | 0 | 0 | 0 | - | 0 | 0 | 0 | 0 |
| Q8WV07 | Oral cancer-overexpressed protein 1 | ORAOV1 | MAGSQDIFDA | 0 | 0 | 0 | 0 | - | 0 | 0 | 0 |
| Q9NRG1 | Phosphoribosyltransferase domain-containing protein 1 | PRTFDC1 | MAGSSEEAPD | 0 | 0 | 0 | 0 | 0 | - | - | - |
| P09493-5 | Isoform 5 of Tropomyosin alpha-1 chain | TPM1 | MAGSSSLEAV | - | 0 | - | - | - | - | - | - |
| Q96IZ6/Q6P1Q9 | Methyltransferase-like protein 2A or 2B | METTL2A/METTL2B | MAGSYPEGAP | - | - | - | - | - | - | - | - |
| P60604 | Ubiquitin-conjugating enzyme E2 G2 | UBE2G2 | MAGTALKRLM | 0 | 0 | 0 | 0 | - | 0 | - | 0 |
| Q9BT30 | Alpha-ketoglutarate-dependent dioxygenase alkB homolog 7, mitochondrial | ALKBH7 | MAGTGLLALR | 0 | 0 | - | - | - | - | 0 | 0 |
| O75934 | Pre-mRNA-splicing factor SPF27 | BCAS2 | MAGTGLVAGE | 0 | 0 | - | - | 0 | 0 | 0 | 0 |
| Q8WXE1 | ATR-interacting protein | ATRIP | MAGTSAPGSK | - | 0 | 0 | 0 | 0 | - | 0 | 0 |
| P25205 | DNA replication licensing factor MCM3 | MCM3 | MAGTVVLDDV | - | - | - | - | - | - | - | - |
| Q6P2Q9 | Pre-mRNA-processing-splicing factor 8 | PRPF8 | MAGVFPYRGP | 0 | 0 | - | 0 | 0 | 0 | 0 | 0 |
| Q6NXT6 | Transmembrane anterior posterior transformation protein 1 homolog | TAPT1 | MAGVGDAAAP | - | 0 | - | - | 0 | - | - | - |
| Q16718 | NADH dehydrogenase [ubiquinone] 1 alpha subcomplex subunit 5 | NDUFA5 | MAGVLKKTTG | 0 | 0 | 0 | 0 | - | 0 | - | 0 |
| P42229 | Signal transducer and activator of transcription 5A | STAT5A | MAGWIQAQQL | 0 | 0 | - | - | 0 | 0 | 0 | 0 |
| Q5JVF3 | PCI domain-containing protein 2 | PCID2 | MAHITINQYL | 0 | - | 0 | 0 | 0 | 0 | - | 0 |
| Q9BZE4 | Nucleolar GTP-binding protein 1 | GTPBP4 | MAHYNFKKIT | 0 | 0 | - | 0 | 0 | 0 | 0 | 0 |
| O15504 | Nucleoporin-like protein 2 | NUPL2 | MAICQFFLQG | - | - | - | 0 | 0 | 0 | 0 | 0 |
| P61619 | Protein transport protein Sec61 subunit alpha isoform 1 | SEC61A1 | MAIKFLEVIK | 0 | 0 | - | 0 | 0 | 0 | 0 | 0 |
| Q9Y2C4 | Nuclease EXOG, mitochondrial | EXOG | MAIKSIASRL | - | 0 | 0 | 0 | 0 | - | 0 | 0 |
| P51809 | Vesicle-associated membrane protein 7 | VAMP7 | MAILFAVVAR | - | 0 | - | - | 0 | 0 | - | 0 |
| P08107 | Heat shock 70 kDa protein 1A/1B | HSPA1A | MAKAAAIGID | 0 | 0 | - | - | - | 0 | 0 | 0 |
| P78316 | Nucleolar protein 14 | NOP14 | MAKAKKVGAR | - | - | - | - | - | - | 0 | - |
| Q4LDG9 | Dynein light chain 1, axonemal | DNAL1 | MAKATTIKEA | - | 0 | 0 | 0 | - | - | - | 0 |
| Q96B96 | Promethin | TMEM159 | MAKEEPQSIS | - | - | - | - | 0 | - | - | - |
| Q9HCJ6 | Synaptic vesicle membrane protein VAT-1 homolog-like | VAT1L | MAKEGVEKAE | 0 | 0 | 0 | 0 | 0 | 0 | 0 | - |
| O15371 | Eukaryotic translation initiation factor 3 subunit D | EIF3D | MAKFMTPVIQ | - | 0 | - | - | 0 | 0 | - | - |
| O15347 | High mobility group protein B3 | HMGB3 | MAKGDPKKPK | 0 | 0 | - | 0 | 0 | 0 | 0 | 0 |
| O75533 | Splicing factor 3B subunit 1 | SF3B1 | MAKIAKTHED | - | - | 0 | 0 | 0 | 0 | 0 | 0 |
| P42766 | 60S ribosomal protein L35 | RPL35 | MAKIKARDLR | - | - | 0 | - | - | - | - | - |
| P31949 | Protein S100-A11 | S100A11 | MAKISSPTET | - | - | - | - | - | - | - | - |
| O95870 | Abhydrolase domain-containing protein 16A | ABHD16A | MAKLLSCVLG | - | - | - | - | - | - | - | - |
| P22314-2 | Isoform 2 of Ubiquitin-like modifier-activating enzyme 1 | UBA1 | MAKNGSEADI | - | - | 0 | 0 | - | - | 0 | 0 |
| Q8N0T1 | Uncharacterized protein C8orf59 | C8orf59 | MAKNKLRGPK | 0 | 0 | 0 | 0 | 0 | - | - | 0 |
| P08133 | Annexin A6 | ANXA6 | MAKPAQGAKY | - | - | - | - | - | - | - | - |
| Q9UL46 | Proteasome activator complex subunit 2 | PSME2 | MAKPCGVRLS | - | 0 | - | - | 0 | - | - | - |
| P06737 | Glycogen phosphorylase, liver form | PYGL | MAKPLTDQEK | - | - | - | - | - | - | - | - |
| P11216 | Glycogen phosphorylase, brain form | PYGB | MAKPLTDSEK | - | - | - | - | - | - | - | - |
| Q16773 | Kynurenine--oxoglutarate transaminase 1 | CCBL1 | MAKQLQARRL | - | 0 | 0 | 0 | - | - | - | - |
| Q9NTX5-2 | Isoform 2 of Ethylmalonyl-CoA decarboxylase | ECHDC1 | MAKSLLKTAS | - | - | - | - | - | - | - | - |
| Q9NZU5 | LIM and cysteine-rich domains protein 1 | LMCD1 | MAKVAKDLNP | 0 | 0 | 0 | 0 | - | - | - | 0 |
| O95292 | Vesicle-associated membrane protein-associated protein B/C | VAPB | MAKVEQVLSL | - | - | 0 | 0 | - | 0 | - | - |
| Q8N5D0 | WD and tetratricopeptide repeats protein 1 | WDTC1 | MAKVNITRDL | 0 | 0 | - | 0 | 0 | - | 0 | 0 |
| Q9NVP2 | Histone chaperone ASF1B | ASF1B | MAKVSVLNVA | 0 | - | 0 | 0 | 0 | 0 | 0 | 0 |
| O95433 | Activator of 90 kDa heat shock protein ATPase homolog 1 | AHSA1 | MAKWGEGDPR | - | - | - | - | 0 | - | - | - |
| Q9Y3D7 | Mitochondrial import inner membrane translocase subunit TIM16 | PAM16 | MAKYLAQIIV | 0 | 0 | 0 | - | 0 | 0 | 0 | 0 |
| Q96CX2 | BTB/POZ domain-containing protein KCTD12 | KCTD12 | MALADSTRGL | 0 | 0 | 0 | 0 | - | - | 0 | - |
| Q9BT67 | NEDD4 family-interacting protein 1 | NDFIP1 | MALALAALAA | - | - | - | - | - | - | - | - |
| Q9UL42 | Paraneoplastic antigen Ma2 | PNMA2 | MALALLEDWC | 0 | 0 | 0 | 0 | 0 | - | - | - |
| P28074 | Proteasome subunit beta type-5 | PSMB5 | MALASVLERP | - | - | 0 | 0 | 0 | 0 | - | 0 |
| Q13608 | Peroxisome assembly factor 2 | PEX6 | MALAVLRVLE | 0 | 0 | - | - | 0 | 0 | 0 | 0 |
| P62195 | 26S protease regulatory subunit 8 | PSMC5 | MALDGPEQME | - | - | - | - | - | - | - | - |
| P50613 | Cyclin-dependent kinase 7 | CDK7 | MALDVKSRAK | - | 0 | - | 0 | - | - | - | 0 |
| P42696 | RNA-binding protein 34 | RBM34 | MALEGMSKRK | - | 0 | 0 | 0 | - | - | 0 | 0 |
| B1AK53 | Espin | ESPN | MALEQALQAA | - | 0 | 0 | 0 | 0 | 0 | 0 | 0 |
| P63272 | Transcription elongation factor SPT4 | SUPT4H1 | MALETVPKDL | - | - | - | - | - | - | - | - |
| Q9H7Z3 | Protein NRDE2 homolog | NRDE2 | MALFPAFAGL | 0 | 0 | - | - | 0 | 0 | 0 | - |
| O14617 | AP-3 complex subunit delta-1 | AP3D1 | MALKMVKGSI | - | - | - | - | - | - | - | - |
| Q9UHD1 | Cysteine and histidine-rich domain-containing protein 1 | CHORDC1 | MALLCYNRGC | - | - | - | - | - | - | - | 0 |
| Q8WW59 | SPRY domain-containing protein 4 | SPRYD4 | MALLFARSLR | 0 | 0 | 0 | 0 | - | - | 0 | 0 |
| Q9H4H8 | Protein FAM83D | FAM83D | MALLSEGLDE | 0 | 0 | 0 | 0 | 0 | 0 | 0 | 0 |
| O60232 | Sjoegren syndrome/scleroderma autoantigen 1 | SSSCA1 | MALNGAEVDD | 0 | 0 | - | 0 | 0 | 0 | 0 | 0 |
| P0CJ79 | Zinc finger protein 888 | ZNF888 | MALPQGLLTF | 0 | - | - | - | 0 | 0 | 0 | 0 |
| Q8N6N7 | Acyl-CoA-binding domain-containing protein 7 | ACBD7 | MALQADFDRA | 0 | 0 | 0 | 0 | 0 | 0 | 0 | 0 |
| Q13257 | Mitotic spindle assembly checkpoint protein MAD2A | MAD2L1 | MALQLSREQG | - | 0 | 0 | 0 | 0 | 0 | 0 | 0 |
| Q96BW9 | Phosphatidate cytidylyltransferase, mitochondrial | TAMM41 | MALQTLQSSW | - | - | 0 | - | 0 | - | 0 | 0 |
| O00159 | Unconventional myosin-Ic | MYO1C | MALQVELVPT | 0 | 0 | 0 | 0 | - | - | 0 | 0 |
| Q7Z4G4 | tRNA (guanine(10)-N2)-methyltransferase homolog | TRMT11 | MALSCTLNRY | - | 0 | 0 | 0 | 0 | - | 0 | 0 |
| Q9BTW9 | Tubulin-specific chaperone D | TBCD | MALSDEPAAG | 0 | 0 | - | - | 0 | 0 | 0 | 0 |
| O15056 | Synaptojanin-2 | SYNJ2 | MALSKGLRLL | 0 | 0 | - | - | - | - | 0 | - |
| Q9Y696 | Chloride intracellular channel protein 4 | CLIC4 | MALSMPLNGL | 0 | 0 | 0 | 0 | 0 | 0 | 0 | 0 |
| A2RRD8 | Zinc finger protein 320 | ZNF320 | MALSQGLLTF | - | 0 | - | 0 | 0 | 0 | 0 | 0 |
| Q9NYP3 | Protein downstream neighbor of Son | DONSON | MALSVPGYSP | 0 | 0 | - | 0 | 0 | 0 | 0 | 0 |
| Q13573 | SNW domain-containing protein 1 | SNW1 | MALTSFLPAP | - | 0 | - | 0 | - | - | 0 | 0 |
| P56545 | C-terminal-binding protein 2 | CTBP2 | MALVDKHKVK | 0 | - | 0 | 0 | 0 | 0 | - | 0 |
| Q9BVM4 | Gamma-glutamylaminecyclotransferase | GGACT | MALVFVYGTL | - | 0 | 0 | 0 | 0 | 0 | 0 | 0 |
| Q8TE77 | Protein phosphatase Slingshot homolog 3 | SSH3 | MALVTVSRSP | - | 0 | 0 | 0 | 0 | 0 | 0 | 0 |
| Q8IYI6 | Exocyst complex component 8 | EXOC8 | MAMAMSDSGA | - | 0 | 0 | - | 0 | - | - | - |
| Q9HAV4 | Exportin-5 | XPO5 | MAMDQVNALC | 0 | 0 | - | 0 | 0 | 0 | 0 | 0 |
| Q99661 | Kinesin-like protein KIF2C | KIF2C | MAMDSSLQAR | - | 0 | 0 | - | - | - | 0 | 0 |
| Q9UBQ5 | Eukaryotic translation initiation factor 3 subunit K | EIF3K | MAMFEQMRAN | - | - | - | - | - | - | - | - |
| Q9NV96 | Cell cycle control protein 50A | TMEM30A | MAMNYNAKDE | - | - | - | - | - | - | 0 | - |
| Q9Y3B4 | Splicing factor 3B subunit 6 | SF3B6 | MAMQAAKRAN | - | 0 | 0 | 0 | - | - | - | 0 |
| Q9BRG1 | Vacuolar protein-sorting-associated protein 25 | VPS25 | MAMSFEWPWQ | 0 | - | 0 | 0 | 0 | 0 | 0 | 0 |
| Q16637 | Survival motor neuron protein | SMN1 | MAMSSGGSGG | - | - | - | - | 0 | - | 0 | - |
| P48059 | LIM and senescent cell antigen-like-containing domain protein 1 | LIMS1 | MANALASATC | 0 | 0 | - | 0 | 0 | 0 | 0 | 0 |
| Q9GZT9 | Egl nine homolog 1 | EGLN1 | MANDSGGPGG | - | 0 | 0 | 0 | 0 | - | - | 0 |
| P11766 | Alcohol dehydrogenase class-3 | ADH5 | MANEVIKCKA | 0 | - | - | - | 0 | 0 | 0 | 0 |
| P61086 | Ubiquitin-conjugating enzyme E2 K | UBE2K | MANIAVQRIK | - | - | 0 | 0 | - | - | - | - |
| Q14690 | Protein RRP5 homolog | PDCD11 | MANLEESFPR | - | 0 | 0 | 0 | - | - | 0 | 0 |
| P40123 | Adenylyl cyclase-associated protein 2 | CAP2 | MANMQGLVER | 0 | 0 | 0 | 0 | - | - | 0 | - |
| Q12904 | Aminoacyl tRNA synthase complex-interacting multifunctional protein 1 | AIMP1 | MANNDAVLKR | - | - | - | - | - | - | - | - |
| P52655 | Transcription initiation factor IIA subunit 1 | GTF2A1 | MANSANTNTV | 0 | 0 | 0 | 0 | - | - | - | 0 |
| Q68CP9 | AT-rich interactive domain-containing protein 2 | ARID2 | MANSTGKAPP | - | 0 | 0 | 0 | 0 | 0 | 0 | 0 |
| O60884 | DnaJ homolog subfamily A member 2 | DNAJA2 | MANVADTKLY | - | 0 | - | - | 0 | - | - | - |
| P51798 | H(+)/Cl(-) exchange transporter 7 | CLCN7 | MANVSKKVSW | 0 | - | - | - | 0 | 0 | 0 | 0 |
| O60683 | Peroxisome biogenesis factor 10 | PEX10 | MAPAAASPPE | 0 | 0 | 0 | 0 | - | - | 0 | 0 |
| P11586 | C-1-tetrahydrofolate synthase, cytoplasmic | MTHFD1 | MAPAEILNGK | - | +/- | +/- | - | - | +/- | +/- | +/- |
| P62899 | 60S ribosomal protein L31 | RPL31 | MAPAKKGGEK | +/- | - | - | - | - | +/- | - | - |
| Q14684 | Ribosomal RNA processing protein 1 homolog B | RRP1B | MAPAMQPAEI | 0 | 0 | 0 | 0 | - | - | 0 | 0 |
| O60427 | Fatty acid desaturase 1 | FADS1 | MAPDPVAAET | +/- | + | +/- | +/- | - | +/- | 0 | 0 |
| Q9BVQ7 | Spermatogenesis-associated protein 5-like protein 1 | SPATA5L1 | MAPDSDPFPE | 0 | 0 | + | + | 0 | 0 | + | 0 |
| P09669 | Cytochrome c oxidase subunit 6C | COX6C | MAPEVLPKPR | - | 0 | +/- | +/- | - | - | 0 | 0 |
| P31939 | Bifunctional purine biosynthesis protein PURH | ATIC | MAPGQLALFS | 0 | - | +/- | - | - | - | - | - |
| Q15785 | Mitochondrial import receptor subunit TOM34 | TOMM34 | MAPKFPDSVE | 0 | 0 | 0 | 0 | 0 | 0 | - | 0 |
| Q9BTX3 | Transmembrane protein 208 | TMEM208 | MAPKGKVGTR | +/- | + | + | + | +/- | +/- | +/- | +/- |
| Q9UNL2 | Translocon-associated protein subunit gamma | SSR3 | MAPKGSSKQQ | - | 0 | - | - | - | +/- | - | - |
| P0CW27 | Coiled-coil domain-containing protein 166 | CCDC166 | MAPKKKRGPS | - | 0 | 0 | 0 | 0 | 0 | 0 | 0 |
| Q86Y39 | NADH dehydrogenase [ubiquinone] 1 alpha subcomplex subunit 11 | NDUFA11 | MAPKVFRQYW | 0 | 0 | - | - | 0 | 0 | 0 | 0 |
| O00743 | Serine/threonine-protein phosphatase 6 catalytic subunit | PPP6C | MAPLDLDKYV | - | - | - | - | - | - | - | - |
| Q9NX62 | Inositol monophosphatase 3 | IMPAD1 | MAPMGIRLSP | 0 | 0 | 0 | 0 | + | + | 0 | 0 |
| P30536 | Translocator protein | TSPO | MAPPWVPAMG | 0 | 0 | - | 0 | 0 | 0 | 0 | 0 |
| Q6P5R6 | 60S ribosomal protein L22-like 1 | RPL22L1 | MAPQKDRKPK | - | 0 | 0 | 0 | 0 | 0 | 0 | 0 |
| Q15833 | Syntaxin-binding protein 2 | STXBP2 | MAPSGLKAVV | - | 0 | - | - | 0 | - | 0 | 0 |
| Q9UIJ5 | Palmitoyltransferase ZDHHC2 | ZDHHC2 | MAPSGPGSSA | + | 0 | 0 | 0 | 0 | + | 0 | 0 |
| Q6P1K1 | Heme transporter HRG1 | SLC48A1 | MAPSRLQLGL | + | + | + | 0 | 0 | 0 | 0 | 0 |
| Q66K64 | DDB1- and CUL4-associated factor 15 | DCAF15 | MAPSSKSERN | - | 0 | 0 | 0 | 0 | - | 0 | 0 |
| Q9BY44 | Eukaryotic translation initiation factor 2A | EIF2A | MAPSTPLLTV | - | - | - | - | - | - | - | - |
| P54819 | Adenylate kinase 2, mitochondrial | AK2 | MAPSVPAAEP | - | - | +/- | +/- | - | - | +/- | - |
| Q8N7H5 | RNA polymerase II-associated factor 1 homolog | PAF1 | MAPTIQTQAQ | - | - | - | - | - | - | - | - |
| Q8TF40 | Folliculin-interacting protein 1 | FNIP1 | MAPTLFQKLF | 0 | 0 | - | - | 0 | 0 | - | 0 |
| Q9ULX3 | RNA-binding protein NOB1 | NOB1 | MAPVEHVVAD | - | 0 | 0 | 0 | 0 | 0 | - | 0 |
| Q08AE8 | Protein spire homolog 1 | SPIRE1 | MAQAAGPAGG | - | 0 | 0 | 0 | 0 | - | 0 | - |
| P52209 | 6-phosphogluconate dehydrogenase, decarboxylating | PGD | MAQADIALIG | 0 | 0 | 0 | - | 0 | 0 | 0 | 0 |
| O95816 | BAG family molecular chaperone regulator 2 | BAG2 | MAQAKINAKA | - | - | - | - | - | - | - | - |
| Q4V328 | GRIP1-associated protein 1 | GRIPAP1 | MAQALSEEEF | - | - | - | - | - | - | - | - |
| P62913 | 60S ribosomal protein L11 | RPL11 | MAQDQGEKEN | - | - | - | - | - | - | - | - |
| Q8IWT0 | Protein archease | ZBTB8OS | MAQEEEDVRD | 0 | 0 | - | 0 | 0 | 0 | 0 | 0 |
| Q7Z309 | Protein FAM122B | FAM122B | MAQEKMELDL | 0 | - | 0 | 0 | 0 | 0 | 0 | 0 |
| P53367 | Arfaptin-1 | ARFIP1 | MAQESPKNSA | - | - | - | - | - | - | - | - |
| Q9NXV6 | CDKN2A-interacting protein | CDKN2AIP | MAQEVSEYLS | - | - | - | - | - | - | - | - |
| Q7Z3C6-3 | Isoform 3 of Autophagy-related protein 9A | ATG9A | MAQFDTEYQR | 0 | - | - | - | 0 | - | 0 | - |
| Q9BU02 | Thiamine-triphosphatase | THTPA | MAQGLIEVER | 0 | 0 | 0 | 0 | 0 | 0 | 0 | 0 |
| Q00610 | Clathrin heavy chain 1 | CLTC | MAQILPIRFQ | - | - | - | - | - | - | - | - |
| Q9Y5A7 | NEDD8 ultimate buster 1 | NUB1 | MAQKKYLQAK | - | 0 | - | - | 0 | - | - | - |
| Q13564 | NEDD8-activating enzyme E1 regulatory subunit | NAE1 | MAQLGKLLKE | - | - | - | - | - | - | - | - |
| Q99623 | Prohibitin-2 | PHB2 | MAQNLKDLAG | - | - | - | - | - | - | - | - |
| Q15276 | Rab GTPase-binding effector protein 1 | RABEP1 | MAQPGPASQP | - | 0 | 0 | 0 | - | - | - | - |
| Q9NUI1 | Peroxisomal 2,4-dienoyl-CoA reductase | DECR2 | MAQPPPDVEG | 0 | 0 | 0 | 0 | - | 0 | 0 | 0 |
| Q712K3 | Ubiquitin-conjugating enzyme E2 R2 | UBE2R2 | MAQQQMTSSQ | 0 | 0 | - | - | 0 | 0 | 0 | 0 |
| P78347 | General transcription factor II-I | GTF2I | MAQVAMSTLP | - | - | - | - | - | - | - | - |
| Q5T2E6 | UPF0668 protein C10orf76 | C10orf76 | MAQVEKRGGL | - | 0 | 0 | 0 | 0 | - | 0 | - |
| P40763 | Signal transducer and activator of transcription 3 | STAT3 | MAQWNQLQQL | - | - | - | - | - | - | - | - |
| Q14320 | Protein FAM50A | FAM50A | MAQYKGAASE | - | - | - | - | - | - | - | - |
| P49427 | Ubiquitin-conjugating enzyme E2 R1 | CDC34 | MARPLVPSSQ | 0 | 0 | - | - | 0 | 0 | 0 | 0 |
| Q6PJF5 | Inactive rhomboid protein 2 | RHBDF2 | MASADKNGGS | - | 0 | 0 | 0 | - | 0 | 0 | 0 |
| Q6ZMI0 | Protein phosphatase 1 regulatory subunit 21 | PPP1R21 | MASAELQGKY | - | 0 | - | 0 | 0 | - | 0 | 0 |
| O95716 | Ras-related protein Rab-3D | RAB3D | MASAGDTQAG | - | 0 | 0 | 0 | - | - | 0 | - |
| Q9BQJ4 | Transmembrane protein 47 | TMEM47 | MASAGSGMEE | 0 | 0 | 0 | 0 | - | - | 0 | 0 |
| Q9NWA0 | Mediator of RNA polymerase II transcription subunit 9 | MED9 | MASAGVAAGR | 0 | 0 | 0 | 0 | - | - | 0 | 0 |
| Q9UNS2 | COP9 signalosome complex subunit 3 | COPS3 | MASALEQFVN | - | - | - | - | - | - | - | - |
| Q9P0L0 | Vesicle-associated membrane protein-associated protein A | VAPA | MASASGAMAK | 0 | 0 | - | - | 0 | 0 | 0 | 0 |
| Q99622 | Protein C10 | C12orf57 | MASASTQPAA | 0 | 0 | - | 0 | 0 | 0 | 0 | 0 |
| Q13496 | Myotubularin | MTM1 | MASASTSKYN | - | 0 | 0 | 0 | 0 | 0 | 0 | 0 |
| Q86VP6 | Cullin-associated NEDD8-dissociated protein 1 | CAND1 | MASASYHISN | - | - | 0 | 0 | 0 | 0 | 0 | 0 |
| O95273 | Cyclin-D1-binding protein 1 | CCNDBP1 | MASATAPAAA | 0 | 0 | - | 0 | 0 | 0 | - | 0 |
| P51610 | Host cell factor 1 | HCFC1 | MASAVSPANL | - | - | - | - | - | - | - | - |
| Q16512 | Serine/threonine-protein kinase N1 | PKN1 | MASDAVQSEP | 0 | 0 | 0 | 0 | 0 | - | 0 | - |
| Q9UBB6-2 | Isoform 2 of Neurochondrin | NCDN | MASDCEPALN | - | - | - | - | 0 | 0 | 0 | 0 |
| Q9P2G9 | Kelch-like protein 8 | KLHL8 | MASDSMSSKQ | - | 0 | 0 | 0 | 0 | - | 0 | 0 |
| P50851 | Lipopolysaccharide-responsive and beige-like anchor protein | LRBA | MASEDNRVPS | - | 0 | 0 | 0 | 0 | 0 | 0 | 0 |
| Q96QU8 | Exportin-6 | XPO6 | MASEEASLRA | 0 | 0 | 0 | 0 | 0 | - | 0 | 0 |
| Q9HB71 | Calcyclin-binding protein | CACYBP | MASEELQKDL | - | - | - | - | - | - | - | 0 |
| Q9Y232-2 | Isoform 2 of Chromodomain Y-like protein | CDYL | MASEELYEVE | - | 0 | - | - | - | - | 0 | 0 |
| P29972 | Aquaporin-1 | AQP1 | MASEFKKKLF | 0 | 0 | - | 0 | 0 | 0 | 0 | 0 |
| Q8IWV8 | E3 ubiquitin-protein ligase UBR2 | UBR2 | MASELEPEVQ | - | - | - | - | - | - | 0 | 0 |
| O75164 | Lysine-specific demethylase 4A | KDM4A | MASESETLNP | - | 0 | 0 | 0 | 0 | - | 0 | 0 |
| P04424 | Argininosuccinate lyase | ASL | MASESGKLWG | - | - | 0 | 0 | 0 | - | 0 | 0 |
| Q9BXP2 | Solute carrier family 12 member 9 | SLC12A9 | MASESSPLLA | - | 0 | 0 | 0 | - | - | 0 | 0 |
| P55072 | Transitional endoplasmic reticulum ATPase | VCP | MASGADSKGD | - | - | 0 | - | - | 0 | - | - |
| P49790 | Nuclear pore complex protein Nup153 | NUP153 | MASGAGGVGG | - | - | - | - | - | - | 0 | 0 |
| Q96AT9 | Ribulose-phosphate 3-epimerase | RPE | MASGCKIGPS | - | 0 | 0 | 0 | 0 | - | - | 0 |
| Q9H4A4 | Aminopeptidase B | RNPEP | MASGEHSPGS | 0 | 0 | 0 | 0 | 0 | 0 | 0 | - |
| O76094 | Signal recognition particle subunit SRP72 | SRP72 | MASGGSGGVS | 0 | 0 | - | - | 0 | - | - | 0 |
| Q16611 | Bcl-2 homologous antagonist/killer | BAK1 | MASGQGPGPP | - | 0 | 0 | 0 | - | - | 0 | - |
| Q96NC0 | Zinc finger matrin-type protein 2 | ZMAT2 | MASGSGTKNL | - | - | - | - | - | - | - | 0 |
| Q13126 | -thioadenosine phosphorylase | MTAP | MASGTTTTAV | - | 0 | 0 | 0 | 0 | 0 | 0 | 0 |
| P23528 | Cofilin-1 | CFL1 | MASGVAVSDG | - | - | - | - | - | - | - | - |
| P60981 | Destrin | DSTN | MASGVQVADE | - | - | - | - | - | - | - | - |
| Q9Y281 | Cofilin-2 | CFL2 | MASGVTVNDE | 0 | 0 | 0 | 0 | - | - | - | - |
| Q9P253 | Vacuolar protein sorting-associated protein 18 homolog | VPS18 | MASILDEYEN | - | - | - | - | - | - | - | - |
| Q16576 | Histone-binding protein RBBP7 | RBBP7 | MASKEMFEDT | - | - | - | - | - | - | - | - |
| Q5XPI4 | E3 ubiquitin-protein ligase RNF123 | RNF123 | MASKGAGMSF | - | 0 | - | - | 0 | 0 | 0 | 0 |
| Q96IK0 | Transmembrane protein 101 | TMEM101 | MASKIGSRRW | - | 0 | 0 | 0 | 0 | - | 0 | 0 |
| Q8WUP2 | Filamin-binding LIM protein 1 | FBLIM1 | MASKPEKRVA | 0 | 0 | 0 | 0 | - | - | 0 | 0 |
| O60762 | Dolichol-phosphate mannosyltransferase subunit 1 | DPM1 | MASLEVSRSP | - | 0 | - | - | - | - | - | - |
| Q9UQN3 | Charged multivesicular body protein 2b | CHMP2B | MASLFKKKTV | - | - | - | - | - | - | - | - |
| O75683 | Surfeit locus protein 6 | SURF6 | MASLLAKDAY | - | 0 | 0 | 0 | 0 | 0 | 0 | 0 |
| P61289 | Proteasome activator complex subunit 3 | PSME3 | MASLLKVDQE | 0 | 0 | - | - | - | - | - | 0 |
| Q9UJW0 | Dynactin subunit 4 | DCTN4 | MASLLQSDRV | - | 0 | - | - | - | - | - | - |
| Q96HA9 | Peroxisomal membrane protein 11C | PEX11G | MASLSGLASA | - | - | 0 | 0 | 0 | 0 | 0 | 0 |
| P78371 | T-complex protein 1 subunit beta | CCT2 | MASLSLAPVN | - | - | - | - | - | - | - | - |
| Q13501 | Sequestosome-1 | SQSTM1 | MASLTVKAYL | 0 | 0 | - | - | 0 | 0 | - | - |
| Q9H425 | Uncharacterized protein C1orf198 | C1orf198 | MASMAAAIAA | 0 | - | - | 0 | 0 | - | 0 | 0 |
| P48643 | T-complex protein 1 subunit epsilon | CCT5 | MASMGTLAFD | - | - | - | - | - | - | - | 0 |
| P80404 | 4-aminobutyrate aminotransferase, mitochondrial | ABAT | MASMLLAQRL | 0 | 0 | 0 | 0 | 0 | 0 | 0 | 0 |
| Q96B02 | Ubiquitin-conjugating enzyme E2 W | UBE2W | MASMQKRLQK | - | 0 | 0 | 0 | 0 | 0 | 0 | 0 |
| P41208 | Centrin-2 | CETN2 | MASNFKKANM | - | 0 | 0 | 0 | - | 0 | - | 0 |
| Q13185 | Chromobox protein homolog 3 | CBX3 | MASNKTTLQK | 0 | 0 | 0 | 0 | 0 | 0 | 0 | 0 |
| Q9NVH2 | Integrator complex subunit 7 | INTS7 | MASNSTKSFL | - | 0 | - | - | 0 | 0 | 0 | 0 |
| P07910 | Heterogeneous nuclear ribonucleoproteins C1/C2 | HNRNPC | MASNVTNKTD | - | - | - | - | - | - | - | - |
| Q9H6L5 | Protein FAM134B | FAM134B | MASPAPPEHA | 0 | 0 | 0 | 0 | 0 | 0 | 0 | - |
| O15131 | Importin subunit alpha-6 | KPNA5 | MASPGKDNYR | - | 0 | 0 | 0 | 0 | - | 0 | - |
| P35568 | Insulin receptor substrate 1 | IRS1 | MASPPESDGF | - | 0 | 0 | 0 | 0 | 0 | 0 | 0 |
| P25325 | 3-mercaptopyruvate sulfurtransferase | MPST | MASPQLCRAL | - | 0 | 0 | 0 | 0 | - | 0 | 0 |
| Q13601 | KRR1 small subunit processome component homolog | KRR1 | MASPSLERPE | - | - | 0 | 0 | 0 | 0 | 0 | 0 |
| Q92506 | Estradiol 17-beta-dehydrogenase 8 | HSD17B8 | MASQLQNRLR | - | 0 | 0 | 0 | 0 | - | 0 | 0 |
| P07954-2 | Isoform Cytoplasmic of Fumarate hydratase, mitochondrial | FH | MASQNSFRIE | - | 0 | 0 | 0 | - | - | 0 | 0 |
| P52732 | Kinesin-like protein KIF11 | KIF11 | MASQPNSSAK | 0 | 0 | - | - | 0 | 0 | 0 | 0 |
| O75348 | V-type proton ATPase subunit G 1 | ATP6V1G1 | MASQSQGIQQ | - | - | - | - | - | - | - | - |
| Q7KZF4 | Staphylococcal nuclease domain-containing protein 1 | SND1 | MASSAQSGGS | - | - | - | - | - | - | - | - |
| P16949 | Stathmin | STMN1 | MASSDIQVKE | - | - | - | - | - | - | - | - |
| O75381 | Peroxisomal membrane protein PEX14 | PEX14 | MASSEQAEQP | - | 0 | - | - | - | - | - | - |
| Q9Y2R0 | Cytochrome c oxidase assembly factor 3 homolog, mitochondrial | COA3 | MASSGAGDPL | - | - | - | - | - | - | - | - |
| Q8N511 | Transmembrane protein 199 | TMEM199 | MASSLLAGER | 0 | 0 | - | - | - | - | - | - |
| Q9Y530 | O-acetyl-ADP-ribose deacetylase 1 | OARD1 | MASSLNEDPE | - | 0 | - | - | 0 | - | 0 | 0 |
| O95861 | -bisphosphate nucleotidase 1 | BPNT1 | MASSNTVLMR | - | 0 | - | - | - | - | - | - |
| Q9BU19 | Zinc finger protein 692 | ZNF692 | MASSPAVDVS | - | 0 | 0 | 0 | 0 | 0 | 0 | 0 |
| Q01658 | Protein Dr1 | DR1 | MASSSGNDDD | 0 | 0 | 0 | 0 | - | 0 | - | 0 |
| P78383 | Solute carrier family 35 member B1 | SLC35B1 | MASSSSLVPD | - | 0 | - | - | 0 | - | 0 | 0 |
| O15294 | UDP-N-acetylglucosamine--peptide N-acetylglucosaminyltransferase 110 kDa subunit | OGT | MASSVGNVAD | - | - | - | - | - | - | - | - |
| Q9BQS8 | FYVE and coiled-coil domain-containing protein 1 | FYCO1 | MASTNAESQL | - | 0 | 0 | 0 | - | - | - | 0 |
| P02538 | Keratin, type II cytoskeletal 6A | KRT6A | MASTSTTIRS | 0 | 0 | 0 | 0 | - | 0 | 0 | 0 |
| O15254 | Peroxisomal acyl-coenzyme A oxidase 3 | ACOX3 | MASTVEGGDT | 0 | 0 | 0 | 0 | - | - | 0 | 0 |
| Q96DA6 | Mitochondrial import inner membrane translocase subunit TIM14 | DNAJC19 | MASTVVAVGL | 0 | 0 | - | - | 0 | - | 0 | 0 |
| Q9H3H1 | tRNA dimethylallyltransferase, mitochondrial | TRIT1 | MASVAAARAV | 0 | 0 | 0 | 0 | 0 | 0 | 0 | 0 |
| Q99541 | Perilipin-2 | PLIN2 | MASVAVDPQP | - | - | - | - | - | - | 0 | 0 |
| Q9UKK6 | NTF2-related export protein 1 | NXT1 | MASVDFKTYV | - | 0 | 0 | 0 | - | - | 0 | 0 |
| P56134 | ATP synthase subunit f, mitochondrial | ATP5J2 | MASVGECPAP | 0 | 0 | 0 | 0 | 0 | 0 | 0 | 0 |
| P46063 | ATP-dependent DNA helicase Q1 | RECQL | MASVSALTEE | 0 | 0 | - | 0 | 0 | 0 | 0 | 0 |
| P56134-2 | Isoform 2 of ATP synthase subunit f, mitochondrial | ATP5J2 | MASVVPVKDK | 0 | 0 | - | 0 | 0 | 0 | 0 | 0 |
| P25686 | DnaJ homolog subfamily B member 2 | DNAJB2 | MASYYEILDV | 0 | 0 | 0 | 0 | 0 | 0 | 0 | - |
| Q15020 | Squamous cell carcinoma antigen recognized by T-cells 3 | SART3 | MATAAETSAS | - | - | - | - | 0 | 0 | - | - |
| P29144 | Tripeptidyl-peptidase 2 | TPP2 | MATAATEEPF | - | 0 | - | - | 0 | 0 | - | - |
| Q8WTW3 | Conserved oligomeric Golgi complex subunit 1 | COG1 | MATAATSPAL | - | - | - | - | - | - | - | - |
| Q92598-4 | Isoform 4 of Heat shock protein 105 kDa | HSPH1 | MATAAVLRGP | + | 0 | 0 | 0 | 0 | 0 | 0 | 0 |
| O43169 | Cytochrome b5 type B | CYB5B | MATAEASGSD | - | - | 0 | 0 | - | - | - | 0 |
| Q9NVH1 | DnaJ homolog subfamily C member 11 | DNAJC11 | MATALSEEEL | 0 | 0 | - | - | - | - | 0 | 0 |
| P47895 | Aldehyde dehydrogenase family 1 member A3 | ALDH1A3 | MATANGAVEN | - | - | 0 | 0 | 0 | 0 | - | - |
| P20700 | Lamin-B1 | LMNB1 | MATATPVPPR | - | 0 | 0 | 0 | - | - | 0 | 0 |
| Q9NUQ8 | ATP-binding cassette sub-family F member 3 | ABCF3 | MATCAEILRS | - | 0 | - | - | - | - | - | - |
| P46934-4 | Isoform 4 of E3 ubiquitin-protein ligase NEDD4 | NEDD4 | MATCAVEVFG | - | 0 | - | - | - | - | - | - |
| Q96C19 | EF-hand domain-containing protein D2 | EFHD2 | MATDELATKL | - | - | - | - | - | - | - | - |
| Q14764 | Major vault protein | MVP | MATEEFIIRI | - | - | 0 | 0 | - | - | - | - |
| P61964 | WD repeat-containing protein 5 | WDR5 | MATEEKKPET | - | - | - | - | - | - | - | - |
| Q9BX69 | Caspase recruitment domain-containing protein 6 | CARD6 | MATESTPSEI | 0 | 0 | 0 | 0 | - | 0 | 0 | 0 |
| P82979 | SAP domain-containing ribonucleoprotein | SARNP | MATETVELHK | - | - | - | - | - | - | - | 0 |
| Q9BYG3 | MKI67 FHA domain-interacting nucleolar phosphoprotein | NIFK | MATFSGPAGP | 0 | 0 | - | - | 0 | 0 | 0 | 0 |
| Q15637 | Splicing factor 1 | SF1 | MATGANATPL | - | - | - | - | - | - | - | - |
| Q9C0F1 | Centrosomal protein of 44 kDa | CEP44 | MATGDLKRSL | 0 | 0 | 0 | 0 | 0 | - | 0 | 0 |
| Q96PU5 | E3 ubiquitin-protein ligase NEDD4-like | NEDD4L | MATGLGEPVY | 0 | 0 | 0 | 0 | 0 | 0 | 0 | - |
| Q08257 | Quinone oxidoreductase | CRYZ | MATGQKLMRA | - | 0 | - | - | - | - | - | - |
| Q6NYC8 | Phostensin | PPP1R18 | MATIPDWKLQ | 0 | 0 | - | - | 0 | - | 0 | 0 |
| P00441 | Superoxide dismutase [Cu-Zn] | SOD1 | MATKAVCVLK | - | 0 | 0 | 0 | 0 | 0 | 0 | 0 |
| Q8WXA3-2 | Isoform 2 of RUN and FYVE domain-containing protein 2 | RUFY2 | MATKDPTAVE | - | 0 | 0 | 0 | 0 | - | - | 0 |
| P09525 | Annexin A4 | ANXA4 | MATKGGTVKA | - | - | 0 | 0 | - | - | - | - |
| Q14019 | Coactosin-like protein | COTL1 | MATKIDKEAC | - | - | - | - | - | - | - | - |
| Q86TV6 | Tetratricopeptide repeat protein 7B | TTC7B | MATKKAGSRL | - | 0 | 0 | 0 | - | - | 0 | - |
| Q6NUQ4 | Transmembrane protein 214 | TMEM214 | MATKTAGVGR | - | - | 0 | 0 | - | - | - | - |
| O95997 | Securin | PTTG1 | MATLIYVDKE | - | 0 | 0 | 0 | - | 0 | - | 0 |
| P00338 | L-lactate dehydrogenase A chain | LDHA | MATLKDQLIY | 0 | 0 | 0 | 0 | 0 | 0 | - | 0 |
| P29692 | Elongation factor 1-delta | EEF1D | MATNFLAHEK | 0 | 0 | - | 0 | 0 | 0 | - | 0 |
| P18583 | Protein SON | SON | MATNIEQIFR | - | - | - | - | - | - | - | 0 |
| P48637 | Glutathione synthetase | GSS | MATNWGSLLQ | - | 0 | - | - | 0 | - | - | - |
| Q92843 | Bcl-2-like protein 2 | BCL2L2 | MATPASAPDT | - | 0 | 0 | 0 | 0 | - | 0 | - |
| P48426 | Phosphatidylinositol 5-phosphate 4-kinase type-2 alpha | PIP4K2A | MATPGNLGSS | - | 0 | 0 | 0 | 0 | 0 | 0 | 0 |
| Q9BW72 | HIG1 domain family member 2A, mitochondrial | HIGD2A | MATPGPVIPE | 0 | 0 | - | 0 | 0 | 0 | 0 | - |
| Q8IY18 | Structural maintenance of chromosomes protein 5 | SMC5 | MATPSKKTST | - | 0 | - | 0 | 0 | 0 | 0 | 0 |
| P35222 | Catenin beta-1 | CTNNB1 | MATQADLMEL | 0 | - | 0 | 0 | - | - | - | 0 |
| Q6PKG0 | La-related protein 1 | LARP1 | MATQVEPLLP | - | 0 | 0 | - | 0 | 0 | - | 0 |
| Q53ET0 | CREB-regulated transcription coactivator 2 | CRTC2 | MATSGANGPG | - | 0 | 0 | 0 | - | - | 0 | 0 |
| Q9NW64 | Pre-mRNA-splicing factor RBM22 | RBM22 | MATSLGSNTY | - | - | - | - | - | - | 0 | 0 |
| Q9NVD7 | Alpha-parvin | PARVA | MATSPQKSPS | - | - | 0 | 0 | - | 0 | - | - |
| Q9UBD5 | Origin recognition complex subunit 3 | ORC3 | MATSSMSKGC | 0 | 0 | - | 0 | 0 | 0 | 0 | 0 |
| Q9GZM5 | Protein YIPF3 | YIPF3 | MATTAAPAGG | - | 0 | 0 | 0 | - | - | 0 | - |
| O00471 | Exocyst complex component 5 | EXOC5 | MATTAELFEE | 0 | 0 | 0 | - | - | - | - | - |
| Q13555 | Calcium/calmodulin-dependent protein kinase type II subunit gamma | CAMK2G | MATTATCTRF | 0 | 0 | 0 | 0 | 0 | 0 | 0 | 0 |
| P38919 | Eukaryotic initiation factor 4A-III | EIF4A3 | MATTATMATS | - | - | - | - | - | - | - | - |
| Q86TI2 | Dipeptidyl peptidase 9 | DPP9 | MATTGTPTAD | - | 0 | 0 | 0 | 0 | 0 | - | - |
| Q9H0E2 | Toll-interacting protein | TOLLIP | MATTVSTQRG | - | 0 | 0 | 0 | - | - | 0 | - |
| Q6N063 | 2-oxoglutarate and iron-dependent oxygenase domain-containing protein 2 | OGFOD2 | MATVGAPRHF | - | 0 | 0 | 0 | 0 | 0 | 0 | 0 |
| A2RU67 | Uncharacterized protein KIAA1467 | KIAA1467 | MATVLSRALK | 0 | 0 | 0 | 0 | 0 | 0 | 0 | - |
| Q01469 | Fatty acid-binding protein, epidermal | FABP5 | MATVQQLEGR | - | - | - | - | - | - | - | - |
| Q9Y230 | RuvB-like 2 | RUVBL2 | MATVTATTKV | - | - | - | - | - | - | - | - |
| P46821 | Microtubule-associated protein 1B | MAP1B | MATVVVEATE | 0 | 0 | 0 | 0 | 0 | 0 | - | - |
| Q15437 | Protein transport protein Sec23B | SEC23B | MATYLEFIQQ | - | - | - | - | - | - | - | - |
| Q9P086 | Mediator of RNA polymerase II transcription subunit 11 | MED11 | MATYSLANER | 0 | 0 | 0 | 0 | - | 0 | 0 | 0 |
| Q13884 | Beta-1-syntrophin | SNTB1 | MAVAAAAAAA | 0 | 0 | 0 | 0 | 0 | 0 | - | - |
| Q32NB8 | CDP-diacylglycerol--glycerol-3-phosphate 3-phosphatidyltransferase, mitochondrial | PGS1 | MAVAAAAAAG | - | 0 | 0 | 0 | 0 | 0 | 0 | 0 |
| Q9NQX4 | Unconventional myosin-Vc | MYO5C | MAVAELYTQY | 0 | - | 0 | 0 | 0 | 0 | 0 | 0 |
| O43447 | Peptidyl-prolyl cis-trans isomerase H | PPIH | MAVANSSPVN | 0 | 0 | - | - | 0 | 0 | - | 0 |
| Q96A72 | Protein mago nashi homolog 2 | MAGOHB | MAVASDFYLR | - | - | - | - | - | - | - | 0 |
| Q9NVA2 | Septin-11 | sept-11 | MAVAVGRPSN | 0 | 0 | 0 | 0 | - | - | 0 | 0 |
| Q9H307 | Pinin | PNN | MAVAVRTLQE | 0 | 0 | 0 | 0 | 0 | 0 | 0 | 0 |
| Q9P2W9 | Syntaxin-18 | STX18 | MAVDITLLFR | - | 0 | 0 | 0 | 0 | 0 | 0 | 0 |
| Q14644 | Ras GTPase-activating protein 3 | RASA3 | MAVEDEGLRV | 0 | 0 | 0 | 0 | 0 | - | 0 | - |
| Q92990 | Glomulin | GLMN | MAVEELQSII | - | - | - | - | - | - | - | - |
| P78346 | Ribonuclease P protein subunit p30 | RPP30 | MAVFADLDLR | - | - | - | - | - | - | - | 0 |
| P61247 | 40S ribosomal protein S3a | RPS3A | MAVGKNKRLT | - | - | - | - | - | - | - | - |
| O75746 | Calcium-binding mitochondrial carrier protein Aralar1 | SLC25A12 | MAVKVQTTKR | - | - | - | - | - | - | - | - |
| Q9Y320 | Thioredoxin-related transmembrane protein 2 | TMX2 | MAVLAPLIAL | - | - | - | - | - | - | - | - |
| Q8N8A2 | Serine/threonine-protein phosphatase 6 regulatory ankyrin repeat subunit B | ANKRD44 | MAVLKLTDQP | 0 | 0 | 0 | 0 | 0 | 0 | 0 | 0 |
| Q96J01 | THO complex subunit 3 | THOC3 | MAVPAAAMGP | 0 | 0 | - | - | 0 | 0 | 0 | 0 |
| Q9Y6G5 | COMM domain-containing protein 10 | COMMD10 | MAVPAALILR | - | 0 | - | - | - | 0 | - | - |
| P21796 | Voltage-dependent anion-selective channel protein 1 | VDAC1 | MAVPPTYADL | - | - | - | - | - | - | - | - |
| P23396 | 40S ribosomal protein S3 | RPS3 | MAVQISKKRK | - | - | - | - | - | - | - | - |
| P43246 | DNA mismatch repair protein Msh2 | MSH2 | MAVQPKETLQ | 0 | 0 | - | - | 0 | - | 0 | 0 |
| P38435 | Vitamin K-dependent gamma-carboxylase | GGCX | MAVSAGSART | 0 | 0 | 0 | 0 | 0 | 0 | 0 | 0 |
| Q99816 | Tumor susceptibility gene 101 protein | TSG101 | MAVSESQLKK | - | 0 | 0 | 0 | - | 0 | - | 0 |
| Q15819 | Ubiquitin-conjugating enzyme E2 variant 2 | UBE2V2 | MAVSTGVKVP | - | - | - | - | - | - | - | - |
| Q9NR56 | Muscleblind-like protein 1 | MBNL1 | MAVSVTPIRD | 0 | 0 | - | - | - | - | - | - |
| Q92871 | Phosphomannomutase 1 | PMM1 | MAVTAQAARR | 0 | 0 | 0 | 0 | 0 | - | 0 | - |
| Q9NW38 | E3 ubiquitin-protein ligase FANCL | FANCL | MAVTEASLLR | 0 | 0 | 0 | 0 | 0 | 0 | 0 | 0 |
| Q9BVM2 | Protein DPCD | DPCD | MAVTGWLESL | - | - | - | - | 0 | 0 | - | - |
| P54725 | UV excision repair protein RAD23 homolog A | RAD23A | MAVTITLKTL | 0 | 0 | 0 | 0 | 0 | 0 | 0 | 0 |
| Q9Y5B9 | FACT complex subunit SPT16 | SUPT16H | MAVTLDKDAY | - | - | - | - | - | - | 0 | 0 |
| Q8IXT5 | RNA-binding protein 12B | RBM12B | MAVVIRLLGL | 0 | 0 | 0 | 0 | - | 0 | 0 | 0 |
| Q9NTZ6 | RNA-binding protein 12 | RBM12 | MAVVIRLQGL | 0 | 0 | 0 | 0 | 0 | 0 | - | 0 |
| Q9H993 | UPF0364 protein C6orf211 | C6orf211 | MAVVPASLSG | 0 | 0 | 0 | 0 | 0 | 0 | 0 | 0 |
| P52657 | Transcription initiation factor IIA subunit 2 | GTF2A2 | MAYQLYRNTT | 0 | 0 | - | - | 0 | - | 0 | 0 |
| P60660 | Myosin light polypeptide 6 | MYL6 | MCDFTEDQTA | - | 0 | - | - | - | - | 0 | 0 |
| P58546 | Myotrophin | MTPN | MCDKEFMWAL | - | 0 | - | - | 0 | 0 | 0 | 0 |
| O94808 | Glutamine--fructose-6-phosphate aminotransferase [isomerizing] 2 | GFPT2 | MCGIFAYMNY | 0 | 0 | 0 | 0 | 0 | 0 | - | 0 |
| Q2VPK5 | Cytoplasmic tRNA 2-thiolation protein 2 | CTU2 | MCQVGEDYGE | - | - | - | - | - | - | 0 | - |
| Q8N8J7 | Uncharacterized protein C4orf32 | C4orf32 | MCSAGELLRG | - | 0 | - | - | 0 | - | 0 | 0 |
| O60678 | Protein arginine N-methyltransferase 3 | PRMT3 | MCSLASGATG | - | 0 | 0 | 0 | 0 | - | 0 | - |
| Q9NRG9 | Aladin | AAAS | MCSLGLFPPP | - | - | - | 0 | - | - | 0 | 0 |
| Q96HD9 | N-acyl-aromatic-L-amino acid amidohydrolase (carboxylate-forming) | ACY3 | MCSLPVPREP | 0 | 0 | 0 | 0 | - | 0 | 0 | 0 |
| Q9Y4P1 | Cysteine protease ATG4B | ATG4B | MDAATLTYDT | + | + | + | + | + | + | + | + |
| Q6IPU0 | Centromere protein P | CENPP | MDAELAEVRA | 0 | 0 | 0 | 0 | 0 | 0 | 0 | 0 |
| Q4J6C6-4 | Isoform 4 of Prolyl endopeptidase-like | PREPL | MDAFEKVRTK | + | + | + | + | + | + | + | + |
| Q8IYB3 | Serine/arginine repetitive matrix protein 1 | SRRM1 | MDAGFFRGTS | 0 | 0 | + | + | + | + | 0 | 0 |
| P09493/P07951 | Tropomyosin alpha-1 or beta chain | TPM1/TPM2 | MDAIKKKMQM | + | 0 | 0 | 0 | 0 | 0 | 0 | 0 |
| Q9UKB3 | DnaJ homolog subfamily C member 12 | DNAJC12 | MDAILNYRSE | 0 | 0 | 0 | 0 | 0 | 0 | 0 | 0 |
| P41226 | Ubiquitin-like modifier-activating enzyme 7 | UBA7 | MDALDASKLL | 0 | 0 | 0 | 0 | 0 | 0 | + | + |
| Q9NNW5 | WD repeat-containing protein 6 | WDR6 | MDALEDYVWP | + | 0 | + | 0 | 0 | + | 0 | 0 |
| Q5SW96 | Low density lipoprotein receptor adapter protein 1 | LDLRAP1 | MDALKSAGRA | + | + | + | 0 | + | + | + | 0 |
| Q9HBH0 | Rho-related GTP-binding protein RhoF | RHOF | MDAPGALAQT | + | + | 0 | 0 | 0 | 0 | 0 | 0 |
| P49585 | Choline-phosphate cytidylyltransferase A | PCYT1A | MDAQCSAKVN | + | + | + | + | + | + | + | 0 |
| Q6P474 | Putative pyridoxal-dependent decarboxylase domain-containing protein 2 | PDXDC2P | MDASLEKIAD | 0 | 0 | 0 | 0 | 0 | 0 | 0 | 0 |
| Q9Y6W3 | Calpain-7 | CAPN7 | MDATALERDA | + | 0 | + | 0 | 0 | 0 | 0 | 0 |
| P60709 | Actin, cytoplasmic 1 | ACTB | MDDDIAALVV | - | - | - | +/- | - | - | - | - |
| Q13418 | Integrin-linked protein kinase | ILK | MDDIFTQCRE | + | + | 0 | 0 | + | + | + | 0 |
| P62714 | Serine/threonine-protein phosphatase 2A catalytic subunit beta isoform | PPP2CB | MDDKAFTKEL | 0 | 0 | 0 | + | 0 | 0 | 0 | 0 |
| Q14232 | Translation initiation factor eIF-2B subunit alpha | EIF2B1 | MDDKELIEYF | 0 | 0 | + | + | 0 | 0 | 0 | 0 |
| Q9BTC0 | Death-inducer obliterator 1 | DIDO1 | MDDKGDPSNE | 0 | 0 | 0 | 0 | 0 | 0 | + | + |
| Q9BUN5 | Coiled-coil domain-containing protein 28B | CCDC28B | MDDKKKKRSP | 0 | 0 | 0 | 0 | 0 | + | + | 0 |
| P62258 | 14-3-3 protein epsilon | YWHAE | MDDREDLVYQ | + | + | 0 | 0 | + | 0 | + | 0 |
| O60716 | Catenin delta-1 | CTNND1 | MDDSEVESTA | 0 | 0 | + | + | 0 | 0 | 0 | 0 |
| Q9NP72 | Ras-related protein Rab-18 | RAB18 | MDEDVLTTLK | 0 | 0 | 0 | 0 | 0 | 0 | 0 | 0 |
| Q8N5A5 | Zinc finger CCCH-type with G patch domain-containing protein | ZGPAT | MDEESLESAL | + | 0 | 0 | 0 | 0 | + | 0 | 0 |
| Q13033 | Striatin-3 | STRN3 | MDELAGGGGG | + | + | 0 | 0 | 0 | + | 0 | + |
| Q04206 | Transcription factor p65 | RELA | MDELFPLIFP | 0 | 0 | + | + | 0 | 0 | 0 | 0 |
| Q9UGV2 | Protein NDRG3 | NDRG3 | MDELQDVQLT | 0 | 0 | + | + | 0 | 0 | + | + |
| O43592 | Exportin-T | XPOT | MDEQALLGLN | 0 | 0 | + | + | + | + | + | + |
| Q9BTU6 | Phosphatidylinositol 4-kinase type 2-alpha | PI4K2A | MDETSPLVSP | + | 0 | 0 | 0 | + | + | 0 | 0 |
| O60784 | Target of Myb protein 1 | TOM1 | MDFLLGNPFS | + | + | + | + | + | + | + | + |
| Q32P44 | Echinoderm microtubule-associated protein-like 3 | EML3 | MDGAAGPGDG | + | 0 | 0 | + | + | + | + | + |
| Q08378 | Golgin subfamily A member 3 | GOLGA3 | MDGASAEQDG | + | + | 0 | 0 | + | + | 0 | + |
| Q99704 | Docking protein 1 | DOK1 | MDGAVMEGPL | 0 | 0 | 0 | 0 | 0 | 0 | 0 | 0 |
| Q9H4I3 | TraB domain-containing protein | TRABD | MDGEEQQPPH | 0 | 0 | + | + | 0 | 0 | 0 | 0 |
| Q16644 | MAP kinase-activated protein kinase 3 | MAPKAPK3 | MDGETAEEQG | 0 | 0 | 0 | 0 | 0 | 0 | 0 | + |
| Q9GZU8 | Protein FAM192A | FAM192A | MDGGDDGNLI | + | + | + | + | + | + | + | + |
| P26599 | Polypyrimidine tract-binding protein 1 | PTBP1 | MDGIVPDIAV | + | + | + | + | + | + | + | + |
| Q96DX5 | Ankyrin repeat and SOCS box protein 9 | ASB9 | MDGKQGGMDG | + | 0 | 0 | 0 | + | + | 0 | 0 |
| Q5T6V5 | UPF0553 protein C9orf64 | C9orf64 | MDGLLNPRES | 0 | 0 | + | + | 0 | + | 0 | 0 |
| Q9H6W3 | Bifunctional lysine-specific demethylase and histidyl-hydroxylase NO66 | NO66 | MDGLQASAGP | 0 | 0 | 0 | 0 | + | 0 | 0 | 0 |
| Q07812 | Apoptosis regulator BAX | BAX | MDGSGEQPRG | + | 0 | 0 | 0 | 0 | +/- | 0 | + |
| O15530-2 | Isoform 2 of 3-phosphoinositide-dependent protein kinase 1 | PDPK1 | MDGTAAEPRP | + | 0 | 0 | 0 | 0 | 0 | 0 | 0 |
| Q9BZ67 | FERM domain-containing protein 8 | FRMD8 | MDGTEGSAGQ | + | + | 0 | 0 | + | + | + | + |
| P78559 | Microtubule-associated protein 1A | MAP1A | MDGVAEFSEY | 0 | 0 | 0 | 0 | 0 | 0 | + | 0 |
| P18440 | Arylamine N-acetyltransferase 1 | NAT1 | MDIEAYLERI | 0 | 0 | 0 | 0 | 0 | 0 | 0 | 0 |
| Q9HAY6 | -monooxygenase | BCO1 | MDIIFGRNRK | + | 0 | + | + | + | 0 | 0 | 0 |
| Q99633 | Pre-mRNA-splicing factor 18 | PRPF18 | MDILKSEILR | + | 0 | + | + | + | + | + | 0 |
| O95562 | Vesicle transport protein SFT2B | SFT2D2 | MDKLKKVLSG | + | + | + | + | + | + | + | + |
| P63104 | 14-3-3 protein zeta/delta | YWHAZ | MDKNELVQKA | +/- | + | + | + | +/- | +/- | + | + |
| P31946-2 | Isoform Short of 14-3-3 protein beta/alpha | YWHAB | MDKSELVQKA | + | + | + | + | + | + | + | + |
| Q8N4T8 | Carbonyl reductase family member 4 | CBR4 | MDKVCAVFGG | + | 0 | 0 | 0 | 0 | + | 0 | 0 |
| Q14566 | DNA replication licensing factor MCM6 | MCM6 | MDLAAAAEPG | + | + | + | + | 0 | 0 | + | + |
| O43542 | DNA repair protein XRCC3 | XRCC3 | MDLDLLDLNP | + | + | 0 | 0 | 0 | 0 | 0 | 0 |
| Q9H0C8 | Integrin-linked kinase-associated serine/threonine phosphatase 2C | ILKAP | MDLFGDLPEP | + | + | + | + | + | + | + | + |
| Q96JP0 | Protein fem-1 homolog C | FEM1C | MDLKTAVFNA | + | 0 | 0 | 0 | 0 | 0 | 0 | 0 |
| Q9BQD3 | KxDL motif-containing protein 1 | KXD1 | MDLPDSASRV | 0 | 0 | 0 | 0 | + | 0 | 0 | 0 |
| Q9NQ92 | Coordinator of PRMT5 and differentiation stimulator | COPRS | MDLQAAGAQA | + | 0 | 0 | + | + | + | + | + |
| O75792 | Ribonuclease H2 subunit A | RNASEH2A | MDLSELERDN | + | + | 0 | + | + | 0 | 0 | 0 |
| Q15287 | RNA-binding protein with serine-rich domain 1 | RNPS1 | MDLSGVKKKS | 0 | 0 | + | 0 | 0 | 0 | 0 | 0 |
| Q9Y6K5 | -oligoadenylate synthase 3 | OAS3 | MDLYSTPAAA | + | + | 0 | 0 | + | 0 | 0 | 0 |
| Q92688 | Acidic leucine-rich nuclear phosphoprotein 32 family member B | ANP32B | MDMKRRIHLE | 0 | 0 | 0 | + | 0 | 0 | 0 | 0 |
| Q9BUJ2-4 | Isoform 4 of Heterogeneous nuclear ribonucleoprotein U-like protein 1 | HNRNPUL1 | MDNITRQNQF | 0 | 0 | 0 | + | 0 | 0 | 0 | 0 |
| O43765 | Small glutamine-rich tetratricopeptide repeat-containing protein alpha | SGTA | MDNKKRLAYA | + | + | 0 | 0 | + | + | 0 | + |
| O00161 | Synaptosomal-associated protein 23 | SNAP23 | MDNLSSEEIQ | + | + | + | + | + | + | + | + |
| Q03111 | Protein ENL | MLLT1 | MDNQCTVQVR | + | 0 | + | + | + | + | 0 | 0 |
| P54920 | Alpha-soluble NSF attachment protein | NAPA | MDNSGKEAEA | 0 | 0 | + | + | + | 0 | + | 0 |
| Q9UBR5 | Chemokine-like factor | CKLF | MDNVQPKIKH | 0 | 0 | 0 | 0 | 0 | 0 | 0 | + |
| P50402 | Emerin | EMD | MDNYADLSDT | 0 | 0 | + | + | + | + | + | + |
| Q9NQW6 | Actin-binding protein anillin | ANLN | MDPFTEKLLE | + | 0 | + | + | + | + | + | + |
| Q6IQ22 | Ras-related protein Rab-12 | RAB12 | MDPGAALQRR | + | + | 0 | 0 | + | + | 0 | 0 |
| Q6P4I2 | WD repeat-containing protein 73 | WDR73 | MDPGDDWLVE | + | + | 0 | 0 | 0 | 0 | 0 | 0 |
| P07992 | DNA excision repair protein ERCC-1 | ERCC1 | MDPGKDKEGV | 0 | 0 | 0 | 0 | 0 | + | + | 0 |
| O95373 | Importin-7 | IPO7 | MDPNTIIEAL | +/- | + | + | + | + | +/- | + | + |
| Q13813 | Spectrin alpha chain, non-erythrocytic 1 | SPTAN1 | MDPSGVKVLE | 0 | + | 0 | 0 | + | + | + | + |
| P29084 | Transcription initiation factor IIE subunit beta | GTF2E2 | MDPSLLRERE | 0 | 0 | + | 0 | 0 | 0 | 0 | 0 |
| Q96GX5 | Serine/threonine-protein kinase greatwall | MASTL | MDPTAGSKKE | + | + | + | + | + | + | + | + |
| Q96LD8 | Sentrin-specific protease 8 | SENP8 | MDPVVLSYMD | + | 0 | + | + | 0 | 0 | 0 | 0 |
| Q9H871 | Protein RMD5 homolog A | RMND5A | MDQCVTVERE | + | 0 | 0 | 0 | + | 0 | 0 | 0 |
| Q8N128 | Protein FAM177A1 | FAM177A1 | MDQEPVGGVE | + | + | 0 | 0 | + | + | 0 | 0 |
| Q9NVI1 | Fanconi anemia group I protein | FANCI | MDQKILSLAA | 0 | 0 | 0 | 0 | 0 | 0 | 0 | 0 |
| P60059 | Protein transport protein Sec61 subunit gamma | SEC61G | MDQVMQFVEP | + | + | + | + | + | + | + | + |
| Q96ER3 | Protein SAAL1 | SAAL1 | MDRNPSPPPP | 0 | 0 | 0 | 0 | 0 | 0 | 0 | 0 |
| O43399 | Tumor protein D54 | TPD52L2 | MDSAGQDINL | 0 | + | + | + | 0 | 0 | + | + |
| Q8WUF5 | RelA-associated inhibitor | PPP1R13L | MDSEAFQSAR | + | + | 0 | 0 | + | + | 0 | 0 |
| Q96ST2 | Protein IWS1 homolog | IWS1 | MDSEYYSGDQ | 0 | 0 | + | 0 | 0 | + | + | + |
| Q99719-2 | Isoform 2 of Septin-5 | sept-05 | MDSLAAPQDR | 0 | 0 | + | 0 | 0 | 0 | 0 | 0 |
| Q9NS91 | E3 ubiquitin-protein ligase RAD18 | RAD18 | MDSLAESRWP | + | 0 | 0 | 0 | 0 | + | 0 | 0 |
| O76024 | Wolframin | WFS1 | MDSNTAPLGP | 0 | 0 | 0 | 0 | + | + | 0 | 0 |
| Q9NVV0 | Trimeric intracellular cation channel type B | TMEM38B | MDSPWDELAL | + | 0 | + | + | 0 | 0 | 0 | + |
| Q9GZU7 | Carboxy-terminal domain RNA polymerase II polypeptide A small phosphatase 1 | CTDSP1 | MDSSAVITQI | + | 0 | 0 | 0 | + | + | + | 0 |
| P49588 | Alanine--tRNA ligase, cytoplasmic | AARS | MDSTLTASEI | + | + | + | + | + | + | + | + |
| Q8NFW8 | N-acylneuraminate cytidylyltransferase | CMAS | MDSVEKGAAT | + | + | + | + | + | + | + | + |
| Q9NWS6 | Protein FAM118A | FAM118A | MDSVEKTTNR | + | 0 | 0 | 0 | + | 0 | 0 | 0 |
| Q8N1F7 | Nuclear pore complex protein Nup93 | NUP93 | MDTEGFGELL | + | + | + | + | 0 | 0 | + | 0 |
| Q15366 | Poly(rC)-binding protein 2 | PCBP2 | MDTGVIEGGL | + | 0 | 0 | 0 | 0 | 0 | 0 | 0 |
| Q8NI08 | Nuclear receptor coactivator 7 | NCOA7 | MDTKEEKKER | + | 0 | 0 | + | + | + | 0 | + |
| Q86W56-2 | Isoform 2 of Poly(ADP-ribose) glycohydrolase | PARG | MDTKGIKTAE | 0 | 0 | 0 | 0 | 0 | 0 | + | 0 |
| P51813 | Cytoplasmic tyrosine-protein kinase BMX | BMX | MDTKSILEEL | 0 | 0 | 0 | 0 | + | + | 0 | 0 |
| Q8NFW9-4 | Isoform 4 of Rab effector MyRIP | MYRIP | MDTLAVALRV | 0 | 0 | 0 | + | 0 | 0 | 0 | 0 |
| Q9H0H5 | Rac GTPase-activating protein 1 | RACGAP1 | MDTMMLNVRN | 0 | 0 | 0 | 0 | 0 | 0 | 0 | 0 |
| Q9UBL3-2 | Isoform 2 of Set1/Ash2 histone methyltransferase complex subunit ASH2 | ASH2L | MDTQAGSVDE | 0 | 0 | 0 | + | 0 | + | 0 | 0 |
| Q15370 | Transcription elongation factor B polypeptide 2 | TCEB2 | MDVFLMIRRH | + | + | + | + | + | + | + | 0 |
| Q8WYA6 | Beta-catenin-like protein 1 | CTNNBL1 | MDVGELLSYQ | + | + | + | + | + | + | + | 0 |
| Q9NW08 | DNA-directed RNA polymerase III subunit RPC2 | POLR3B | MDVLAEEFGN | 0 | 0 | + | + | 0 | 0 | 0 | 0 |
| P54136 | Arginine--tRNA ligase, cytoplasmic | RARS | MDVLVSECSA | + | + | + | + | + | + | 0 | 0 |
| O75915 | PRA1 family protein 3 | ARL6IP5 | MDVNIAPLRA | + | + | + | + | +/- | + | + | + |
| Q99527 | G-protein coupled estrogen receptor 1 | GPER1 | MDVTSQARGV | + | 0 | 0 | + | 0 | 0 | 0 | 0 |
| Q8IY22 | C-Maf-inducing protein | CMIP | MDVTSSSGGG | + | 0 | 0 | + | + | 0 | 0 | 0 |
| O14787 | Transportin-2 | TNPO2 | MDWQPDEQGL | 0 | 0 | + | + | 0 | 0 | 0 | 0 |
| Q8N573-2 | Isoform 2 of Oxidation resistance protein 1 | OXR1 | MDYLTTFTEK | 0 | 0 | 0 | 0 | 0 | 0 | 0 | 0 |
| Q9Y5Y2 | Cytosolic Fe-S cluster assembly factor NUBP2 | NUBP2 | MEAAAEPGNL | + | + | + | + | + | + | + | + |
| Q9NPH2 | Inositol-3-phosphate synthase 1 | ISYNA1 | MEAAAQFFVE | 0 | 0 | + | 0 | 0 | 0 | 0 | 0 |
| Q6YBV0 | Proton-coupled amino acid transporter 4 | SLC36A4 | MEAAATPAAA | 0 | 0 | 0 | 0 | + | + | 0 | 0 |
| Q8NFI3 | Cytosolic endo-beta-N-acetylglucosaminidase | ENGASE | MEAAAVTVTR | 0 | 0 | 0 | 0 | 0 | 0 | 0 | 0 |
| Q96I15 | Selenocysteine lyase | SCLY | MEAAVAPGRD | + | 0 | + | + | + | 0 | + | 0 |
| Q01804 | OTU domain-containing protein 4 | OTUD4 | MEAAVGVPDG | + | + | + | 0 | + | + | 0 | 0 |
| Q9Y6M7-10 | Isoform 10 of Sodium bicarbonate cotransporter 3 | SLC4A7 | MEADGAGEQM | + | 0 | 0 | 0 | 0 | 0 | 0 | 0 |
| Q96EA4 | Protein Spindly | SPDL1 | MEADIITNLR | 0 | 0 | 0 | 0 | + | 0 | 0 | 0 |
| Q96NB2 | Sideroflexin-2 | SFXN2 | MEADLSGFNI | + | + | 0 | 0 | + | + | 0 | 0 |
| Q8IXU6 | Solute carrier family 35 member F2 | SLC35F2 | MEADSPAGPG | 0 | 0 | + | 0 | 0 | 0 | + | + |
| P28070 | Proteasome subunit beta type-4 | PSMB4 | MEAFLGSRSG | 0 | 0 | + | 0 | 0 | 0 | 0 | 0 |
| Q96JM3 | Chromosome alignment-maintaining phosphoprotein 1 | CHAMP1 | MEAFQELRKP | + | 0 | + | + | 0 | + | 0 | 0 |
| Q6XZF7 | Dynamin-binding protein | DNMBP | MEAGSVVRAI | + | 0 | 0 | 0 | + | + | 0 | 0 |
| P62993 | Growth factor receptor-bound protein 2 | GRB2 | MEAIAKYDFK | + | 0 | + | + | 0 | + | 0 | 0 |
| Q9BW66 | Cyclin-dependent kinase 2-interacting protein | CINP | MEAKTLGTVT | 0 | 0 | + | 0 | + | 0 | 0 | 0 |
| Q6NT16 | MFS-type transporter SLC18B1 | SLC18B1 | MEALGDLEGP | 0 | + | + | 0 | 0 | 0 | 0 | 0 |
| Q9Y282 | Endoplasmic reticulum-Golgi intermediate compartment protein 3 | ERGIC3 | MEALGKLKQF | + | 0 | + | + | 0 | + | + | + |
| P61923 | Coatomer subunit zeta-1 | COPZ1 | MEALILEPSL | 0 | 0 | 0 | 0 | 0 | 0 | 0 | 0 |
| P06132 | Uroporphyrinogen decarboxylase | UROD | MEANGLGPQG | + | + | + | + | + | + | + | + |
| P51817 | cAMP-dependent protein kinase catalytic subunit PRKX | PRKX | MEAPGLAQAA | + | 0 | 0 | 0 | 0 | 0 | 0 | 0 |
| O94763 | Unconventional prefoldin RPB5 interactor 1 | URI1 | MEAPTVETPP | 0 | + | 0 | 0 | 0 | 0 | 0 | 0 |
| Q13045 | Protein flightless-1 homolog | FLII | MEATGVLPFV | + | + | + | + | + | + | + | + |
| Q8WUD4 | Coiled-coil domain-containing protein 12 | CCDC12 | MEATTAGVGR | + | + | + | + | + | + | + | 0 |
| Q8N6M0 | OTU domain-containing protein 6B | OTUD6B | MEAVLTEELD | 0 | 0 | + | + | 0 | 0 | + | 0 |
| Q9H3S7 | Tyrosine-protein phosphatase non-receptor type 23 | PTPN23 | MEAVPRMPMI | 0 | 0 | 0 | 0 | 0 | 0 | 0 | 0 |
| Q9BW60 | Elongation of very long chain fatty acids protein 1 | ELOVL1 | MEAVVNLYQE | + | + | 0 | 0 | 0 | 0 | + | 0 |
| P31483 | Nucleolysin TIA-1 isoform p40 | TIA1 | MEDEMPKTLY | 0 | 0 | + | + | + | + | 0 | 0 |
| P23193 | Transcription elongation factor A protein 1 | TCEA1 | MEDEVVRFAK | + | 0 | + | + | + | + | 0 | 0 |
| Q9NYT0 | Pleckstrin-2 | PLEK2 | MEDGVLKEGF | + | + | 0 | 0 | 0 | 0 | 0 | 0 |
| Q9NQC3 | Reticulon-4 | RTN4 | MEDLDQSPLV | 0 | 0 | + | + | 0 | 0 | + | + |
| Q9Y6D9 | Mitotic spindle assembly checkpoint protein MAD1 | MAD1L1 | MEDLGENTMV | 0 | + | 0 | 0 | + | + | 0 | 0 |
| Q96FT9 | Intraflagellar transport protein 43 homolog | IFT43 | MEDLLDLDEE | 0 | 0 | 0 | 0 | 0 | 0 | + | 0 |
| O76021 | Ribosomal L1 domain-containing protein 1 | RSL1D1 | MEDSASASLS | 0 | 0 | + | 0 | 0 | + | + | + |
| O95677 | Eyes absent homolog 4 | EYA4 | MEDSQDLNEQ | 0 | 0 | 0 | 0 | 0 | 0 | + | + |
| Q6IBW4 | Condensin-2 complex subunit H2 | NCAPH2 | MEDVEARFAH | 0 | 0 | 0 | + | 0 | + | 0 | 0 |
| Q9P270 | SLAIN motif-containing protein 2 | SLAIN2 | MEDVNSNVNA | 0 | 0 | + | 0 | + | + | + | 0 |
| Q86TP1 | Protein prune homolog | PRUNE | MEDYLQGCRA | 0 | 0 | 0 | 0 | 0 | 0 | 0 | 0 |
| P06493 | Cyclin-dependent kinase 1 | CDK1 | MEDYTKIEKI | + | 0 | 0 | 0 | + | + | 0 | 0 |
| Q9NP74 | Palmdelphin | PALMD | MEEAELVKGR | 0 | 0 | 0 | 0 | + | + | + | + |
| Q8TD55 | Pleckstrin homology domain-containing family O member 2 | PLEKHO2 | MEEEGVKEAG | + | + | 0 | + | + | + | + | + |
| P63261 | Actin, cytoplasmic 2 | ACTG1 | MEEEIAALVI | - | 0 | - | - | - | - | +/- | +/- |
| P43686 | 26S protease regulatory subunit 6B | PSMC4 | MEEIGILVEK | 0 | 0 | + | + | 0 | 0 | 0 | 0 |
| Q5TH69 | Brefeldin A-inhibited guanine nucleotide-exchange protein 3 | ARFGEF3 | MEEILRKLQK | + | 0 | + | 0 | 0 | 0 | 0 | 0 |
| Q8N720 | Zinc finger protein 655 | ZNF655 | MEEIPAQEAA | + | 0 | 0 | 0 | 0 | 0 | 0 | 0 |
| Q8NFH3 | Nucleoporin Nup43 | NUP43 | MEEIYAKFVS | + | 0 | + | + | 0 | 0 | 0 | + |
| Q9H4G0-2 | Isoform 2 of Band 4.1-like protein 1 | EPB41L1 | MEEKDYSEAD | + | 0 | 0 | 0 | 0 | 0 | 0 | + |
| O60711 | Leupaxin | LPXN | MEELDALLEE | 0 | 0 | 0 | 0 | 0 | 0 | + | 0 |
| Q9HA64 | Ketosamine-3-kinase | FN3KRP | MEELLRRELG | 0 | 0 | 0 | 0 | 0 | 0 | 0 | 0 |
| O95155 | Ubiquitin conjugation factor E4 B | UBE4B | MEELSADEIR | + | 0 | 0 | 0 | 0 | 0 | 0 | 0 |
| O15446 | DNA-directed RNA polymerase I subunit RPA34 | CD3EAP | MEEPQAGDAA | + | 0 | 0 | 0 | + | + | 0 | 0 |
| P10155 | 60 kDa SS-A/Ro ribonucleoprotein | TROVE2 | MEESVNQMQP | + | 0 | 0 | 0 | 0 | 0 | 0 | 0 |
| P53384 | Cytosolic Fe-S cluster assembly factor NUBP1 | NUBP1 | MEEVPHDCPG | 0 | 0 | 0 | 0 | 0 | 0 | + | 0 |
| O60830 | Mitochondrial import inner membrane translocase subunit Tim17-B | TIMM17B | MEEYAREPCP | 0 | 0 | 0 | 0 | 0 | + | 0 | 0 |
| Q96QG7 | Myotubularin-related protein 9 | MTMR9 | MEFAELIKTP | + | + | + | + | + | + | + | + |
| Q9GZP8 | Immortalization up-regulated protein | IMUP | MEFDLGAALE | + | + | 0 | 0 | 0 | 0 | 0 | 0 |
| Q9NR50 | Translation initiation factor eIF-2B subunit gamma | EIF2B3 | MEFQAVVMAV | + | 0 | + | 0 | + | + | 0 | + |
| Q9BYX2 | TBC1 domain family member 2A | TBC1D2 | MEGAGENAPE | + | 0 | 0 | 0 | 0 | + | 0 | + |
| Q9Y5L0 | Transportin-3 | TNPO3 | MEGAKPTLQL | 0 | 0 | + | + | 0 | 0 | + | 0 |
| Q9BZF1 | Oxysterol-binding protein-related protein 8 | OSBPL8 | MEGGLADGEP | + | + | 0 | 0 | 0 | 0 | 0 | 0 |
| P51991-2 | Isoform 2 of Heterogeneous nuclear ribonucleoprotein A3 | HNRNPA3 | MEGHDPKEPE | 0 | 0 | 0 | 0 | 0 | 0 | + | 0 |
| Q8IUH4 | Palmitoyltransferase ZDHHC13 | ZDHHC13 | MEGPGLGSQC | 0 | 0 | 0 | 0 | + | + | 0 | 0 |
| P17987 | T-complex protein 1 subunit alpha | TCP1 | MEGPLSVFGD | +/- | +/- | +/- | +/- | + | + | + | + |
| Q8WVP7 | Limb region 1 protein homolog | LMBR1 | MEGQDEVSAR | + | 0 | 0 | + | 0 | + | 0 | 0 |
| Q15050 | Ribosome biogenesis regulatory protein homolog | RRS1 | MEGQSVEELL | + | 0 | 0 | 0 | + | + | + | 0 |
| Q96NT5 | Proton-coupled folate transporter | SLC46A1 | MEGSASPPEK | + | 0 | + | 0 | 0 | + | 0 | 0 |
| P49366 | Deoxyhypusine synthase | DHPS | MEGSLEREAP | + | 0 | 0 | 0 | 0 | + | 0 | 0 |
| P18124 | 60S ribosomal protein L7 | RPL7 | MEGVEEKKKE | + | + | + | + | + | + | + | + |
| Q5SNT2 | Transmembrane protein 201 | TMEM201 | MEGVSALLAR | + | + | 0 | 0 | + | 0 | 0 | 0 |
| Q8N3V7-2 | Isoform 2 of Synaptopodin | SYNPO | MEGYSEEASL | 0 | 0 | 0 | 0 | + | 0 | 0 | 0 |
| Q6PJT7 | Zinc finger CCCH domain-containing protein 14 | ZC3H14 | MEIGTEISRK | 0 | 0 | 0 | 0 | + | 0 | 0 | 0 |
| Q09666 | Neuroblast differentiation-associated protein AHNAK | AHNAK | MEKEETTREL | + | + | 0 | 0 | + | +/- | + | + |
| Q9Y2G2-3 | Isoform 3 of Caspase recruitment domain-containing protein 8 | CARD8 | MEKKECPEKS | 0 | 0 | + | 0 | + | + | 0 | 0 |
| Q9Y365 | PCTP-like protein | STARD10 | MEKLAASTEP | + | 0 | 0 | 0 | 0 | 0 | 0 | 0 |
| Q9Y223 | Bifunctional UDP-N-acetylglucosamine 2-epimerase/N-acetylmannosamine kinase | GNE | MEKNGNNRKL | + | 0 | 0 | 0 | 0 | 0 | 0 | 0 |
| Q9H5K3 | Protein O-mannose kinase | POMK | MEKQPQNSRR | + | 0 | 0 | 0 | + | + | 0 | 0 |
| P43007 | Neutral amino acid transporter A | SLC1A4 | MEKSNETNGY | 0 | 0 | 0 | 0 | 0 | 0 | + | + |
| P27348 | 14-3-3 protein theta | YWHAQ | MEKTELIQKA | + | + | + | + | + | + | + | + |
| P22626 | Heterogeneous nuclear ribonucleoproteins A2/B1 | HNRNPA2B1 | MEKTLETVPL | + | + | + | + | + | + | + | + |
| O95749 | Geranylgeranyl pyrophosphate synthase | GGPS1 | MEKTQETVQR | + | + | + | + | + | + | + | 0 |
| O94927 | HAUS augmin-like complex subunit 5 | HAUS5 | MELAQEAREL | 0 | 0 | + | 0 | 0 | 0 | 0 | 0 |
| P61165 | Transmembrane protein 258 | TMEM258 | MELEAMSRYT | + | + | + | + | + | + | + | + |
| O60271 | C-Jun-amino-terminal kinase-interacting protein 4 | SPAG9 | MELEDGVVYQ | 0 | + | 0 | 0 | + | + | + | + |
| P62195-2 | Isoform 2 of 26S protease regulatory subunit 8 | PSMC5 | MELEEGKAGS | + | + | 0 | 0 | + | - | - | 0 |
| Q06203 | Amidophosphoribosyltransferase | PPAT | MELEELGIRE | 0 | 0 | 0 | 0 | 0 | 0 | 0 | 0 |
| P34947 | G protein-coupled receptor kinase 5 | GRK5 | MELENIVANT | 0 | 0 | + | + | 0 | 0 | 0 | 0 |
| Q8IUI8-2 | Isoform 2 of Cytokine receptor-like factor 3 | CRLF3 | MELEPELLLQ | + | + | + | + | + | + | 0 | 0 |
| P51648 | Fatty aldehyde dehydrogenase | ALDH3A2 | MELEVRRVRQ | 0 | 0 | 0 | 0 | + | + | 0 | 0 |
| Q7Z4W1 | L-xylulose reductase | DCXR | MELFLAGRRV | + | + | + | + | +/- | + | + | + |
| Q9BTE1 | Dynactin subunit 5 | DCTN5 | MELGELLYNK | 0 | 0 | + | + | 0 | 0 | 0 | 0 |
| Q9H6R7 | WD repeat-containing protein C2orf44 | C2orf44 | MELGKGKLLR | + | + | + | 0 | + | + | 0 | 0 |
| O95551 | Tyrosyl-DNA phosphodiesterase 2 | TDP2 | MELGSCLEGG | + | + | 0 | 0 | 0 | 0 | 0 | 0 |
| Q14974 | Importin subunit beta-1 | KPNB1 | MELITILEKT | + | 0 | + | + | 0 | + | 0 | 0 |
| Q9BXV9 | Uncharacterized protein C14orf142 | C14orf142 | MELLGEYVGQ | 0 | 0 | + | 0 | + | + | 0 | 0 |
| Q9Y679 | Ancient ubiquitous protein 1 | AUP1 | MELPSGPGPE | + | 0 | + | + | 0 | + | + | + |
| Q8WUX1 | Sodium-coupled neutral amino acid transporter 5 | SLC38A5 | MELQDPKMNG | 0 | + | + | 0 | + | 0 | 0 | 0 |
| Q68E01 | Integrator complex subunit 3 | INTS3 | MELQKGKGAA | + | + | + | + | + | + | + | + |
| P08195 | 4F2 cell-surface antigen heavy chain | SLC3A2 | MELQPPEASI | + | + | + | + | 0 | 0 | 0 | 0 |
| Q9H3K6 | BolA-like protein 2 | BOLA2 | MELSAEYLRE | + | + | + | + | + | + | + | + |
| P55060 | Exportin-2 | CSE1L | MELSDANLQT | 0 | 0 | + | + | 0 | 0 | 0 | 0 |
| Q8N1A0 | Keratin-like protein KRT222 | KRT222 | MELSQLLNEI | + | 0 | 0 | 0 | 0 | 0 | 0 | 0 |
| Q32P44-3 | Isoform 3 of Echinoderm microtubule-associated protein-like 3 | EML3 | MELVKAALAE | 0 | 0 | + | 0 | 0 | 0 | 0 | 0 |
| Q9UI09 | NADH dehydrogenase [ubiquinone] 1 alpha subcomplex subunit 12 | NDUFA12 | MELVQVLKRG | + | + | + | + | + | + | + | + |
| Q8IYB9 | Zinc finger protein 595 | ZNF595 | MELVTFRDVA | 0 | 0 | 0 | 0 | 0 | 0 | 0 | 0 |
| Q9BTT0 | Acidic leucine-rich nuclear phosphoprotein 32 family member E | ANP32E | MEMKKKINLE | + | + | + | + | + | + | + | + |
| Q8IWX8 | Calcium homeostasis endoplasmic reticulum protein | CHERP | MEMPLPPDDQ | 0 | 0 | 0 | + | 0 | + | 0 | 0 |
| Q96PZ0 | Pseudouridylate synthase 7 homolog | PUS7 | MEMTEMTGVS | + | 0 | + | 0 | 0 | + | 0 | 0 |
| Q9UHB6-2 | Isoform Alpha of LIM domain and actin-binding protein 1 | LIMA1 | MENCLGESRH | + | 0 | 0 | 0 | + | + | 0 | 0 |
| Q9NX76 | CKLF-like MARVEL transmembrane domain-containing protein 6 | CMTM6 | MENGAVYSPT | + | + | + | + | + | + | + | + |
| Q9Y6M4 | Casein kinase I isoform gamma-3 | CSNK1G3 | MENKKKDKDK | 0 | 0 | 0 | 0 | + | + | + | 0 |
| Q7Z2T5 | TRMT1-like protein | TRMT1L | MENMAEEELL | 0 | 0 | + | 0 | 0 | 0 | 0 | 0 |
| Q9NVW2 | E3 ubiquitin-protein ligase RLIM | RLIM | MENSDSNDKG | + | 0 | 0 | 0 | 0 | + | 0 | + |
| O43310 | CBP80/20-dependent translation initiation factor | CTIF | MENSSAASAS | + | 0 | 0 | 0 | 0 | + | + | 0 |
| Q14651 | Plastin-1 | PLS1 | MENSTTTISR | + | 0 | 0 | 0 | 0 | 0 | 0 | 0 |
| P47224 | Guanine nucleotide exchange factor MSS4 | RABIF | MEPAEQPSEL | + | 0 | 0 | 0 | 0 | 0 | + | 0 |
| Q9UJY5 | ADP-ribosylation factor-binding protein GGA1 | GGA1 | MEPAMEPETL | + | 0 | 0 | 0 | + | + | 0 | + |
| Q9Y4R8 | Telomere length regulation protein TEL2 homolog | TELO2 | MEPAPSEVRL | + | 0 | + | 0 | + | + | 0 | 0 |
| Q15738 | Sterol-4-alpha-carboxylate 3-dehydrogenase, decarboxylating | NSDHL | MEPAVSEPMR | + | + | + | + | + | + | + | + |
| P36954 | DNA-directed RNA polymerase II subunit RPB9 | POLR2I | MEPDGTYEPG | + | 0 | 0 | 0 | + | + | + | 0 |
| P48163 | NADP-dependent malic enzyme | ME1 | MEPEAPRRRH | + | 0 | 0 | 0 | 0 | + | 0 | 0 |
| Q7Z6J9 | tRNA-splicing endonuclease subunit Sen54 | TSEN54 | MEPEPEPAAV | + | 0 | 0 | 0 | 0 | 0 | 0 | 0 |
| Q9UIQ6 | Leucyl-cystinyl aminopeptidase | LNPEP | MEPFTNDRLQ | 0 | 0 | + | + | + | 0 | + | 0 |
| P19623 | Spermidine synthase | SRM | MEPGPDGPAA | +/- | + | + | + | + | + | + | + |
| Q01432-3 | Isoform 1C of AMP deaminase 3 | AMPD3 | MEPGSAEMPR | 0 | 0 | 0 | 0 | 0 | 0 | 0 | 0 |
| Q8TED1 | Probable glutathione peroxidase 8 | GPX8 | MEPLAAYPLK | + | 0 | 0 | 0 | + | + | 0 | 0 |
| P46379 | Large proline-rich protein BAG6 | BAG6 | MEPNDSTSTA | 0 | 0 | + | + | 0 | 0 | + | + |
| Q9UHD8-3 | Isoform 3 of Septin-9 | sept-09 | MEPPASKVPE | 0 | 0 | 0 | 0 | 0 | 0 | + | + |
| O60610 | Protein diaphanous homolog 1 | DIAPH1 | MEPPGGSLGP | + | + | + | + | + | + | + | + |
| Q99726 | Zinc transporter 3 | SLC30A3 | MEPSPAAGGL | + | 0 | 0 | 0 | 0 | 0 | 0 | 0 |
| P11166 | Solute carrier family 2, facilitated glucose transporter member 1 | SLC2A1 | MEPSSKKLTG | + | + | + | + | + | + | + | + |
| Q16719 | Kynureninase | KYNU | MEPSSLELPA | 0 | 0 | 0 | 0 | 0 | 0 | + | + |
| O95999 | B-cell lymphoma/leukemia 10 | BCL10 | MEPTAPSLTE | 0 | 0 | 0 | 0 | 0 | 0 | + | 0 |
| Q6P9H4 | Connector enhancer of kinase suppressor of ras 3 | CNKSR3 | MEPVTKWSPK | + | 0 | 0 | 0 | 0 | 0 | 0 | 0 |
| O95219 | Sorting nexin-4 | SNX4 | MEQAPPDPER | + | + | 0 | 0 | + | + | + | + |
| P02549 | Spectrin alpha chain, erythrocytic 1 | SPTA1 | MEQFPKETVV | 0 | 0 | + | 0 | 0 | 0 | 0 | 0 |
| Q9H479 | Fructosamine-3-kinase | FN3K | MEQLLRAELR | 0 | 0 | + | + | 0 | 0 | 0 | 0 |
| P30740 | Leukocyte elastase inhibitor | SERPINB1 | MEQLSSANTR | + | + | + | + | + | + | + | + |
| O60879 | Protein diaphanous homolog 2 | DIAPH2 | MEQPGAAASG | + | 0 | 0 | 0 | + | + | + | + |
| Q96AY4 | Tetratricopeptide repeat protein 28 | TTC28 | MEQSPPPAPE | 0 | 0 | 0 | 0 | + | + | 0 | 0 |
| Q99549 | M-phase phosphoprotein 8 | MPHOSPH8 | MEQVAEGARV | + | 0 | 0 | 0 | 0 | + | 0 | 0 |
| Q05209 | Tyrosine-protein phosphatase non-receptor type 12 | PTPN12 | MEQVEILRKF | + | + | + | 0 | + | + | + | 0 |
| Q08495-3 | Isoform 3 of Dematin | DMTN | MERLQKAKMD | - | 0 | 0 | 0 | - | 0 | - | - |
| Q3ZAQ7 | Vacuolar ATPase assembly integral membrane protein VMA21 | VMA21 | MERPDKAALN | + | 0 | 0 | 0 | 0 | 0 | 0 | + |
| Q8TEX9 | Importin-4 | IPO4 | MESAGLEQLL | + | + | + | + | + | + | + | + |
| P78344 | Eukaryotic translation initiation factor 4 gamma 2 | EIF4G2 | MESAIAEGGA | +/- | + | + | + | + | + | + | + |
| Q96EM0 | Trans-L-3-hydroxyproline dehydratase | L3HYPDH | MESALAVPRL | 0 | 0 | + | 0 | + | + | + | + |
| O00159-2 | Isoform 2 of Unconventional myosin-Ic | MYO1C | MESALTARDR | + | + | + | 0 | + | + | 0 | 0 |
| P61326 | Protein mago nashi homolog | MAGOH | MESDFYLRYY | + | + | + | + | +/- | + | 0 | 0 |
| P33981 | Dual specificity protein kinase TTK | TTK | MESEDLSGRE | + | 0 | + | + | + | + | 0 | 0 |
| Q9NRX1 | RNA-binding protein PNO1 | PNO1 | MESEMETQSA | 0 | 0 | 0 | 0 | + | + | 0 | + |
| O43760 | Synaptogyrin-2 | SYNGR2 | MESGAYGAAK | + | + | + | + | 0 | + | 0 | 0 |
| Q8TDP1 | Ribonuclease H2 subunit C | RNASEH2C | MESGDEAAIE | + | 0 | 0 | 0 | 0 | + | 0 | 0 |
| P10644 | cAMP-dependent protein kinase type I-alpha regulatory subunit | PRKAR1A | MESGSTAASE | + | + | + | + | + | + | + | + |
| Q9BWM7 | Sideroflexin-3 | SFXN3 | MESKMGELPL | 0 | + | 0 | 0 | + | + | + | + |
| Q9BQC3 | Diphthamide biosynthesis protein 2 | DPH2 | MESMFSSPAE | + | 0 | 0 | 0 | 0 | 0 | 0 | 0 |
| Q9NXW2 | DnaJ homolog subfamily B member 12 | DNAJB12 | MESNKDEAER | + | 0 | 0 | 0 | 0 | + | 0 | + |
| P46734 | Dual specificity mitogen-activated protein kinase kinase 3 | MAP2K3 | MESPASSQPA | + | + | + | + | + | + | + | + |
| Q8NBA8 | DTW domain-containing protein 2 | DTWD2 | MESQKEARTL | + | 0 | 0 | 0 | 0 | + | 0 | + |
| Q15814 | Tubulin-specific chaperone C | TBCC | MESVSCSAAA | + | + | + | + | + | + | 0 | 0 |
| P61163 | Alpha-centractin | ACTR1A | MESYDVIANQ | 0 | 0 | 0 | 0 | 0 | 0 | 0 | 0 |
| P29401 | Transketolase | TKT | MESYHKPDQQ | + | + | + | + | + | + | + | + |
| P61978 | Heterogeneous nuclear ribonucleoprotein K | HNRNPK | METEQPEETF | 0 | 0 | + | 0 | + | + | 0 | + |
| Q4G0J3 | La-related protein 7 | LARP7 | METESGNQEK | + | 0 | 0 | 0 | 0 | 0 | 0 | 0 |
| P49642 | DNA primase small subunit | PRIM1 | METFDPTELP | 0 | 0 | + | 0 | 0 | 0 | 0 | 0 |
| Q99685-2 | Isoform 2 of Monoglyceride lipase | MGLL | METGPEDPSS | 0 | 0 | 0 | 0 | + | + | + | 0 |
| Q4KMP7 | TBC1 domain family member 10B | TBC1D10B | METGTAPLVA | + | + | + | + | + | + | 0 | + |
| Q12874 | Splicing factor 3A subunit 3 | SF3A3 | METILEQQRR | + | + | + | + | + | + | + | + |
| Q9BZD4 | Kinetochore protein Nuf2 | NUF2 | METLSFPRYN | 0 | 0 | 0 | + | + | 0 | 0 | 0 |
| P50453 | Serpin B9 | SERPINB9 | METLSNASGT | + | + | + | + | 0 | + | 0 | 0 |
| Q9NRP0 | Oligosaccharyltransferase complex subunit OSTC | OSTC | METLYRVPFL | 0 | 0 | + | + | 0 | 0 | 0 | 0 |
| O60684 | Importin subunit alpha-7 | KPNA6 | METMASPGKD | + | + | + | 0 | + | + | + | + |
| P02545 | Prelamin-A/C | LMNA | METPSQRRAT | + | 0 | 0 | 0 | 0 | + | 0 | + |
| P40937 | Replication factor C subunit 5 | RFC5 | METSALKQQE | + | + | + | + | + | + | + | + |
| Q9BWH2 | FUN14 domain-containing protein 2 | FUNDC2 | METSAPRAGS | 0 | 0 | 0 | 0 | 0 | + | 0 | 0 |
| Q13043 | Serine/threonine-protein kinase 4 | STK4 | METVQLRNPP | 0 | 0 | 0 | 0 | 0 | + | 0 | 0 |
| Q96RS6 | NudC domain-containing protein 1 | NUDCD1 | MEVAANCSLR | + | + | + | + | + | + | + | 0 |
| O15164 | Transcription intermediary factor 1-alpha | TRIM24 | MEVAVEKAVA | 0 | 0 | + | + | 0 | 0 | 0 | 0 |
| Q8WVX3 | Uncharacterized protein C4orf3 | C4orf3 | MEVDAPGVDG | + | + | + | + | +/- | +/- | + | + |
| Q7L1V2 | Vacuolar fusion protein MON1 homolog B | MON1B | MEVGGDTAAP | 0 | 0 | + | 0 | 0 | 0 | 0 | + |
| Q8N5I9 | Uncharacterized protein C12orf45 | C12orf45 | MEVHGKPKAS | 0 | 0 | + | 0 | 0 | 0 | 0 | 0 |
| O75781 | Paralemmin-1 | PALM | MEVLAAETTS | + | + | + | 0 | + | + | 0 | 0 |
| Q9Y5B0 | RNA polymerase II subunit A C-terminal domain phosphatase | CTDP1 | MEVPAAGRVP | 0 | 0 | + | 0 | 0 | + | 0 | 0 |
| P11388 | DNA topoisomerase 2-alpha | TOP2A | MEVSPLQPVN | 0 | 0 | + | 0 | 0 | 0 | 0 | 0 |
| Q8IUR7-2 | Isoform 2 of Armadillo repeat-containing protein 8 | ARMC8 | MEVTASSRHY | 0 | 0 | 0 | 0 | + | 0 | 0 | 0 |
| O00273 | DNA fragmentation factor subunit alpha | DFFA | MEVTGDAGVP | + | 0 | + | + | 0 | 0 | + | + |
| Q7Z2K6 | Endoplasmic reticulum metallopeptidase 1 | ERMP1 | MEWGSESAAV | 0 | + | + | 0 | 0 | + | 0 | 0 |
| Q92973-2 | Isoform 2 of Transportin-1 | TNPO1 | MEYEWKPDEQ | 0 | 0 | + | 0 | 0 | 0 | 0 | 0 |
| P49721 | Proteasome subunit beta type-2 | PSMB2 | MEYLIGIQGP | 0 | 0 | + | 0 | + | 0 | + | 0 |
| Q96D46 | 60S ribosomal export protein NMD3 | NMD3 | MEYMAESTDR | + | + | + | + | + | + | + | + |
| Q05932-2 | Isoform 2 of Folylpolyglutamate synthase, mitochondrial | FPGS | MEYQDAVRML | 0 | 0 | 0 | 0 | 0 | 0 | 0 | 0 |
| P41214 | Eukaryotic translation initiation factor 2D | EIF2D | MFAKAFRVKS | + | + | + | + | + | + | 0 | 0 |
| O60443 | Non-syndromic hearing impairment protein 5 | DFNA5 | MFAKATRNFL | 0 | 0 | 0 | 0 | 0 | + | + | + |
| Q14691 | DNA replication complex GINS protein PSF1 | GINS1 | MFCEKAMELI | + | 0 | 0 | 0 | 0 | 0 | 0 | 0 |
| Q53FT3 | Protein Hikeshi | C11orf73 | MFGCLVAGRL | 0 | 0 | + | + | + | 0 | 0 | 0 |
| Q07820 | Induced myeloid leukemia cell differentiation protein Mcl-1 | MCL1 | MFGLKRNAVI | 0 | 0 | + | 0 | 0 | 0 | 0 | 0 |
| Q13439 | Golgin subfamily A member 4 | GOLGA4 | MFKKLKQKIS | 0 | 0 | 0 | 0 | 0 | 0 | 0 | 0 |
| Q9NPE3 | H/ACA ribonucleoprotein complex subunit 3 | NOP10 | MFLQYYLNEQ | + | 0 | + | 0 | 0 | 0 | 0 | 0 |
| P04632 | Calpain small subunit 1 | CAPNS1 | MFLVNSFLKG | 0 | 0 | 0 | 0 | 0 | 0 | 0 | 0 |
| Q15393 | Splicing factor 3B subunit 3 | SF3B3 | MFLYNLTLQR | + | + | + | + | 0 | + | + | 0 |
| Q8WUM0 | Nuclear pore complex protein Nup133 | NUP133 | MFPAAPSPRT | + | + | + | + | + | 0 | 0 | 0 |
| Q8N1F1 | Putative uncharacterized protein LRRC75A-AS1, mitochondrial | LRRC75A-AS1 | MFPGSLSRGR | + | 0 | 0 | 0 | 0 | 0 | 0 | 0 |
| Q13620-1 | Isoform 2 of Cullin-4B | CUL4B | MFPTGFSSPS | 0 | 0 | 0 | 0 | + | + | + | + |
| Q9H2P0 | Activity-dependent neuroprotector homeobox protein | ADNP | MFQLPVNNLG | + | + | + | + | 0 | + | 0 | 0 |
| Q3YEC7 | Rab-like protein 6 | RABL6 | MFSALKKLVG | + | + | + | + | + | + | + | + |
| P49005 | DNA polymerase delta subunit 2 | POLD2 | MFSEQAAQRA | + | 0 | + | + | + | + | 0 | 0 |
| P62081 | 40S ribosomal protein S7 | RPS7 | MFSSSAKIVK | 0 | 0 | 0 | 0 | 0 | 0 | 0 | 0 |
| Q86VS8 | Protein Hook homolog 3 | HOOK3 | MFSVESLERA | 0 | 0 | 0 | 0 | + | 0 | + | 0 |
| Q9NZN4 | EH domain-containing protein 2 | EHD2 | MFSWLKRGGA | + | + | + | + | 0 | + | + | + |
| Q9H223 | EH domain-containing protein 4 | EHD4 | MFSWMGRQAG | + | 0 | + | 0 | + | + | 0 | 0 |
| Q9H4M9 | EH domain-containing protein 1 | EHD1 | MFSWVSKDAR | + | + | + | + | + | + | + | + |
| Q0PNE2 | Elongator complex protein 6 | ELP6 | MFVELNNLLN | + | + | + | + | + | 0 | + | 0 |
| Q9BSL1 | Ubiquitin-associated domain-containing protein 1 | UBAC1 | MFVQEEKIFA | 0 | 0 | + | + | 0 | 0 | 0 | 0 |
| O75419 | Cell division control protein 45 homolog | CDC45 | MFVSDFRKEF | + | 0 | + | + | 0 | + | 0 | 0 |
| Q9H4I0 | Double-strand-break repair protein rad21-like protein 1 | RAD21L1 | MFYTHVLMSK | + | 0 | 0 | 0 | 0 | 0 | 0 | 0 |
| Q5VT66 | Mitochondrial amidoxime-reducing component 1 | MARC1 | MGAAGSSALA | - | 0 | 0 | 0 | 0 | 0 | 0 | 0 |
| Q02952 | A-kinase anchor protein 12 | AKAP12 | MGAGSSTEQR | - | - | 0 | 0 | - | - | 0 | 0 |
| Q8NFA0 | Ubiquitin carboxyl-terminal hydrolase 32 | USP32 | MGAKESRIGF | - | - | 0 | 0 | 0 | 0 | 0 | 0 |
| O75896 | Tumor suppressor candidate 2 | TUSC2 | MGASGSKARG | - | 0 | 0 | 0 | 0 | 0 | 0 | 0 |
| Q6I9Y2 | THO complex subunit 7 homolog | THOC7 | MGAVTDDEVI | 0 | 0 | - | - | - | 0 | 0 | 0 |
| P61313 | 60S ribosomal protein L15 | RPL15 | MGAYKYIQEL | - | - | - | - | 0 | - | - | - |
| Q8WWI5 | Choline transporter-like protein 1 | SLC44A1 | MGCCSSASSA | - | 0 | 0 | 0 | 0 | - | 0 | 0 |
| Q9BY42 | Protein RTF2 homolog | RTFDC1 | MGCDGGTIPK | 0 | 0 | 0 | 0 | 0 | - | 0 | 0 |
| P30825 | High affinity cationic amino acid transporter 1 | SLC7A1 | MGCKVLLNIG | - | - | - | - | - | - | - | - |
| P63092 | Guanine nucleotide-binding protein G(s) subunit alpha isoforms short | GNAS | MGCLGNSKTE | - | - | 0 | 0 | - | - | - | 0 |
| P08754 | Guanine nucleotide-binding protein G(k) subunit alpha | GNAI3 | MGCTLSAEDK | 0 | - | 0 | 0 | 0 | 0 | 0 | 0 |
| P04899 | Guanine nucleotide-binding protein G(i) subunit alpha-2 | GNAI2 | MGCTVSAEDK | - | 0 | 0 | 0 | - | - | 0 | 0 |
| Q3B7T1 | Erythroid differentiation-related factor 1 | EDRF1 | MGDAKEAGAE | - | - | - | - | 0 | - | - | - |
| P54577 | Tyrosine--tRNA ligase, cytoplasmic | YARS | MGDAPSPEEK | - | - | 0 | 0 | - | 0 | - | - |
| Q9UQ88 | Cyclin-dependent kinase 11A | CDK11A | MGDEKDSWKV | 0 | 0 | - | - | 0 | 0 | 0 | 0 |
| P35658 | Nuclear pore complex protein Nup214 | NUP214 | MGDEMDAMIP | - | 0 | - | - | - | - | - | - |
| P12235 | ADP/ATP translocase 1 | SLC25A4 | MGDHAWSFLK | 0 | 0 | - | 0 | 0 | 0 | 0 | 0 |
| P13637 | Sodium/potassium-transporting ATPase subunit alpha-3 | ATP1A3 | MGDKKDDKDS | - | - | 0 | 0 | 0 | 0 | 0 | 0 |
| Q8IY57-3 | Isoform 3 of YY1-associated factor 2 | YAF2 | MGDKKSPTRK | 0 | 0 | 0 | 0 | 0 | 0 | 0 | 0 |
| Q8IY57 | YY1-associated factor 2 | YAF2 | MGDKKSPTRP | - | 0 | 0 | 0 | 0 | 0 | 0 | 0 |
| Q96F85 | CB1 cannabinoid receptor-interacting protein 1 | CNRIP1 | MGDLPGLVRL | 0 | 0 | - | 0 | - | - | 0 | 0 |
| Q9NP61 | ADP-ribosylation factor GTPase-activating protein 3 | ARFGAP3 | MGDPSKQDIL | - | 0 | - | - | - | - | - | - |
| Q04917 | 14-3-3 protein eta | YWHAH | MGDREQLLQR | 0 | 0 | 0 | - | 0 | 0 | 0 | 0 |
| Q9NQT8 | Kinesin-like protein KIF13B | KIF13B | MGDSKVKVAV | - | - | - | 0 | - | - | - | 0 |
| Q08211 | ATP-dependent RNA helicase A | DHX9 | MGDVKNFLYA | - | - | - | - | 0 | - | 0 | 0 |
| Q96TA1 | Niban-like protein 1 | FAM129B | MGDVLSTHLD | 0 | - | 0 | 0 | 0 | 0 | 0 | 0 |
| P0CB43 | Protein FAM203B | FAM203B | MGEAGAGAGA | 0 | 0 | - | 0 | 0 | 0 | - | - |
| Q92989 | -hydroxyl-kinase Clp1 | CLP1 | MGEEANDDKK | - | - | - | - | - | 0 | 0 | 0 |
| Q96B97-3 | Isoform 3 of SH3 domain-containing kinase-binding protein 1 | SH3KBP1 | MGEETIGKKL | 0 | 0 | 0 | 0 | 0 | 0 | - | - |
| Q9NZD8 | Maspardin | SPG21 | MGEIKVSPDY | - | 0 | - | 0 | 0 | 0 | 0 | 0 |
| O96015 | Dynein light chain 4, axonemal | DNAL4 | MGETEGKKDE | - | 0 | 0 | 0 | 0 | 0 | 0 | 0 |
| Q14683 | Structural maintenance of chromosomes protein 1A | SMC1A | MGFLKLIEIE | 0 | 0 | - | 0 | 0 | 0 | 0 | 0 |
| P22307-2 | Isoform SCP2 of Non-specific lipid-transfer protein | SCP2 | MGFPEAASSF | 0 | 0 | - | - | 0 | 0 | 0 | 0 |
| P46777 | 60S ribosomal protein L5 | RPL5 | MGFVKVVKNK | - | - | - | - | - | - | - | - |
| O15554 | Intermediate conductance calcium-activated potassium channel protein 4 | KCNN4 | MGGDLVLGLG | - | - | - | - | 0 | 0 | - | 0 |
| Q7Z478 | ATP-dependent RNA helicase DHX29 | DHX29 | MGGKNKKHKA | - | - | 0 | 0 | 0 | 0 | - | - |
| O00541 | Pescadillo homolog | PES1 | MGGLEKKKYE | - | - | - | - | - | - | - | - |
| P69891 | Hemoglobin subunit gamma-1 | HBG1 | MGHFTEEDKA | 0 | 0 | - | - | 0 | 0 | 0 | 0 |
| P55039 | Developmentally-regulated GTP-binding protein 2 | DRG2 | MGILEKISEI | 0 | 0 | - | - | 0 | - | - | 0 |
| Q8NB46 | Serine/threonine-protein phosphatase 6 regulatory ankyrin repeat subunit C | ANKRD52 | MGILSITDQP | 0 | 0 | - | - | 0 | 0 | 0 | 0 |
| P39748 | Flap endonuclease 1 | FEN1 | MGIQGLAKLI | - | 0 | +/- | +/- | - | - | - | - |
| Q9GZR2 | RNA exonuclease 4 | REXO4 | MGKAKVPASK | - | - | - | - | - | - | 0 | - |
| P25685 | DnaJ homolog subfamily B member 1 | DNAJB1 | MGKDYYQTLG | - | - | - | - | - | - | - | - |
| Q92625 | Ankyrin repeat and SAM domain-containing protein 1A | ANKS1A | MGKEQELLEA | - | - | - | 0 | 0 | 0 | 0 | 0 |
| P61353 | 60S ribosomal protein L27 | RPL27 | MGKFMKPGKV | - | - | - | - | - | - | - | - |
| B2RPK0 | Putative high mobility group protein B1-like 1 | HMGB1P1 | MGKGDPKKPR | 0 | + | - | +/- | - | 0 | 0 | +/- |
| P09429 | High mobility group protein B1 | HMGB1 | MGKGDPKKPR | 0 | 0 | 0 | 0 | 0 | 0 | + | 0 |
| P26583 | High mobility group protein B2 | HMGB2 | MGKGDPNKPR | - | - | - | +/- | - | +/- | - | - |
| O95864 | Fatty acid desaturase 2 | FADS2 | MGKGGNQGEG | 0 | 0 | 0 | 0 | - | +/- | 0 | 0 |
| Q9NWV4 | UPF0587 protein C1orf123 | C1orf123 | MGKIALQLKA | 0 | - | - | - | 0 | - | 0 | 0 |
| Q8TBB5 | Kelch domain-containing protein 4 | KLHDC4 | MGKKGKKEKK | - | - | - | - | - | - | - | - |
| Q8IY81 | pre-rRNA processing protein FTSJ3 | FTSJ3 | MGKKGKVGKS | - | - | - | - | - | - | - | 0 |
| Q9BV36 | Melanophilin | MLPH | MGKKLDLSKL | 0 | 0 | 0 | 0 | 0 | 0 | - | - |
| Q9Y324 | rRNA-processing protein FCF1 homolog | FCF1 | MGKQKKTRKY | - | 0 | 0 | 0 | - | - | 0 | 0 |
| P37235 | Hippocalcin-like protein 1 | HPCAL1 | MGKQNSKLRP | 0 | - | 0 | 0 | 0 | 0 | 0 | 0 |
| O60870 | DNA/RNA-binding protein KIN17 | KIN | MGKSDFLTPK | - | - | - | - | - | - | - | - |
| P04406 | Glyceraldehyde-3-phosphate dehydrogenase | GAPDH | MGKVKVGVNG | - | +/- | - | +/- | - | +/- | +/- | +/- |
| Q9BVS4 | Serine/threonine-protein kinase RIO2 | RIOK2 | MGKVNVAKLR | - | - | - | - | - | - | - | - |
| O95159 | Zinc finger protein-like 1 | ZFPL1 | MGLCKCPKRK | 0 | 0 | 0 | 0 | 0 | - | 0 | 0 |
| P0CG29 | Glutathione S-transferase theta-2 | GSTT2 | MGLELFLDLV | 0 | 0 | 0 | 0 | - | - | 0 | 0 |
| O00255 | Menin | MEN1 | MGLKAAQKTL | - | - | - | - | 0 | - | 0 | 0 |
| Q8WXX5 | DnaJ homolog subfamily C member 9 | DNAJC9 | MGLLDLCEEV | 0 | 0 | - | - | 0 | 0 | 0 | 0 |
| O43292 | Glycosylphosphatidylinositol anchor attachment 1 protein | GPAA1 | MGLLSDPVRR | 0 | 0 | - | - | 0 | - | 0 | - |
| P36405 | ADP-ribosylation factor-like protein 3 | ARL3 | MGLLSILRKL | - | - | - | - | - | - | - | - |
| P48506 | Glutamate--cysteine ligase catalytic subunit | GCLC | MGLLSQGSPL | - | - | - | - | 0 | 0 | 0 | - |
| P36404 | ADP-ribosylation factor-like protein 2 | ARL2 | MGLLTILKKM | - | - | - | - | - | - | - | 0 |
| Q9UNX4 | WD repeat-containing protein 3 | WDR3 | MGLTKQYLRY | 0 | - | - | 0 | 0 | 0 | 0 | 0 |
| Q96P11 | Probable 28S rRNA (cytosine-C(5))-methyltransferase | NSUN5 | MGLYAAAAGV | - | - | - | - | 0 | - | 0 | 0 |
| O60936 | Nucleolar protein 3 | NOL3 | MGNAQERPSE | 0 | - | 0 | 0 | 0 | 0 | 0 | 0 |
| Q86VD7 | Mitochondrial coenzyme A transporter SLC25A42 | SLC25A42 | MGNGVKEGPV | 0 | 0 | 0 | 0 | 0 | 0 | 0 | 0 |
| P84077 | ADP-ribosylation factor 1 | ARF1 | MGNIFANLFK | 0 | - | - | 0 | 0 | 0 | 0 | 0 |
| P61204 | ADP-ribosylation factor 3 | ARF3 | MGNIFGNLLK | 0 | - | 0 | 0 | 0 | 0 | 0 | 0 |
| Q6NUT3 | Major facilitator superfamily domain-containing protein 12 | MFSD12 | MGPGPPAAGA | + | + | + | + | 0 | + | + | + |
| Q15003 | Condensin complex subunit 2 | NCAPH | MGPPGPALPA | 0 | 0 | - | 0 | 0 | 0 | 0 | 0 |
| O15260 | Surfeit locus protein 4 | SURF4 | MGQNDLMGTA | - | - | - | - | - | - | - | - |
| P62191 | 26S protease regulatory subunit 4 | PSMC1 | MGQSQSGGHG | 0 | - | 0 | 0 | 0 | 0 | 0 | 0 |
| Q9UQR0 | Sex comb on midleg-like protein 2 | SCML2 | MGQTVNEDSM | 0 | 0 | - | 0 | 0 | 0 | 0 | 0 |
| Q8N4P3 | -pyrophosphohydrolase MESH1 | HDDC3 | MGSEAAQLLE | - | 0 | - | - | - | - | - | 0 |
| Q96QC0 | Serine/threonine-protein phosphatase 1 regulatory subunit 10 | PPP1R10 | MGSGPIDPKE | 0 | 0 | - | - | 0 | - | 0 | 0 |
| O75298-3 | Isoform RTN2-C of Reticulon-2 | RTN2 | MGSKVADLLY | - | 0 | 0 | 0 | 0 | 0 | 0 | 0 |
| P12931 | Proto-oncogene tyrosine-protein kinase Src | SRC | MGSNKSKPKD | - | - | 0 | 0 | - | - | 0 | 0 |
| Q96AA3 | Protein RFT1 homolog | RFT1 | MGSQEVLGHA | 0 | 0 | 0 | 0 | 0 | 0 | 0 | 0 |
| P49006 | MARCKS-related protein | MARCKSL1 | MGSQSSKAPR | - | - | 0 | 0 | - | - | 0 | 0 |
| Q9Y4D8-5 | Isoform 5 of Probable E3 ubiquitin-protein ligase HECTD4 | HECTD4 | MGSSAAAAAA | 0 | 0 | 0 | - | 0 | 0 | 0 | 0 |
| O00625 | Pirin | PIR | MGSSKKVTLS | - | - | - | - | - | - | - | 0 |
| Q9H8Y8 | Golgi reassembly-stacking protein 2 | GORASP2 | MGSSQSVEIP | 0 | - | 0 | 0 | 0 | 0 | 0 | 0 |
| Q7RTP0 | Magnesium transporter NIPA1 | NIPA1 | MGTAAAAAAA | - | - | - | - | - | - | 0 | 0 |
| P0DJ07 | Protein PET100 homolog, mitochondrial | PET100 | MGVKLEIFRM | 0 | 0 | 0 | 0 | 0 | 0 | 0 | 0 |
| Q9H0D6 | exoribonuclease 2 | XRN2 | MGVPAFFRWL | 0 | 0 | - | 0 | 0 | 0 | 0 | 0 |
| Q9NZB2 | Constitutive coactivator of PPAR-gamma-like protein 1 | FAM120A | MGVQGFQDYI | - | 0 | 0 | 0 | 0 | 0 | 0 | 0 |
| P28715 | DNA repair protein complementing XP-G cells | ERCC5 | MGVQGLWKLL | - | 0 | - | 0 | 0 | - | 0 | - |
| P62942 | Peptidyl-prolyl cis-trans isomerase FKBP1A | FKBP1A | MGVQVETISP | - | - | - | - | - | - | - | - |
| Q9H944 | Mediator of RNA polymerase II transcription subunit 20 | MED20 | MGVTCVSQMP | 0 | 0 | - | - | 0 | 0 | - | - |
| Q8N183 | Mimitin, mitochondrial | NDUFAF2 | MGWSQDLFRA | 0 | 0 | - | 0 | 0 | 0 | 0 | 0 |
| Q52LW3 | Rho GTPase-activating protein 29 | ARHGAP29 | MIAHKQKKTK | + | 0 | 0 | 0 | 0 | 0 | 0 | 0 |
| Q7Z401 | C-myc promoter-binding protein | DENND4A | MIEDKGPRVA | 0 | 0 | 0 | 0 | 0 | 0 | 0 | 0 |
| Q96DI7 | U5 small nuclear ribonucleoprotein 40 kDa protein | SNRNP40 | MIEQQKRKGP | + | 0 | 0 | + | + | + | 0 | 0 |
| Q9BZL1 | Ubiquitin-like protein 5 | UBL5 | MIEVVCNDRL | + | 0 | + | + | + | 0 | + | 0 |
| Q9BZ72 | Membrane-associated phosphatidylinositol transfer protein 2 | PITPNM2 | MIIKEYRIPL | +/- | + | 0 | 0 | + | 0 | + | 0 |
| Q92572 | AP-3 complex subunit sigma-1 | AP3S1 | MIKAILIFNN | 0 | + | 0 | 0 | 0 | 0 | 0 | 0 |
| Q9UBF2 | Coatomer subunit gamma-2 | COPG2 | MIKKFDKKDE | + | 0 | 0 | 0 | 0 | 0 | 0 | 0 |
| P61081 | NEDD8-conjugating enzyme Ubc12 | UBE2M | MIKLFSLKQQ | + | 0 | + | + | 0 | 0 | + | + |
| O15144 | Actin-related protein 2/3 complex subunit 2 | ARPC2 | MILLEVNNRI | + | + | + | + | + | + | + | + |
| P54136-2 | Isoform Monomeric of Arginine--tRNA ligase, cytoplasmic | RARS | MINIISRLQE | 0 | 0 | + | + | 0 | + | 0 | 0 |
| Q8TDW0 | Volume-regulated anion channel subunit LRRC8C | LRRC8C | MIPVTEFRQF | 0 | 0 | 0 | 0 | + | 0 | 0 | 0 |
| Q8IWT6 | Volume-regulated anion channel subunit LRRC8A | LRRC8A | MIPVTELRYF | 0 | 0 | 0 | 0 | + | 0 | 0 | 0 |
| O95232 | Luc7-like protein 3 | LUC7L3 | MISAAQLLDE | + | + | + | + | + | + | + | + |
| Q9Y5P6 | Mannose-1-phosphate guanyltransferase beta | GMPPB | MKALILVGGY | + | + | + | 0 | + | + | + | + |
| Q02543 | 60S ribosomal protein L18a | RPL18A | MKASGTLREY | + | + | + | + | + | + | + | + |
| Q8TEA8 | D-tyrosyl-tRNA(Tyr) deacylase 1 | DTD1 | MKAVVQRVTR | + | + | + | + | + | + | + | + |
| Q8IXJ9 | Putative Polycomb group protein ASXL1 | ASXL1 | MKDKQKKKKE | + | 0 | 0 | 0 | 0 | 0 | 0 | 0 |
| Q07326 | Phosphatidylinositol-glycan biosynthesis class F protein | PIGF | MKDNDIKRLL | + | 0 | 0 | 0 | + | + | 0 | 0 |
| O76071 | Probable cytosolic iron-sulfur protein assembly protein CIAO1 | CIAO1 | MKDSLVLLGR | + | + | + | + | + | + | + | + |
| Q92974-3 | Isoform 3 of Rho guanine nucleotide exchange factor 2 | ARHGEF2 | MKEAKDARYT | 0 | 0 | + | + | + | + | + | 0 |
| Q2VIQ3 | Chromosome-associated kinesin KIF4B | KIF4B | MKEEVKGIPV | + | 0 | + | + | + | + | 0 | + |
| Q9BWT3 | Poly(A) polymerase gamma | PAPOLG | MKEMSANTVL | + | 0 | 0 | 0 | 0 | 0 | 0 | 0 |
| P20290-2 | Isoform 2 of Transcription factor BTF3 | BTF3 | MKETIMNQEK | + | + | + | + | + | + | + | + |
| Q06265 | Exosome complex component RRP45 | EXOSC9 | MKETPLSNCE | + | + | + | + | + | + | 0 | 0 |
| P61254 | 60S ribosomal protein L26 | RPL26 | MKFNPFVTSD | + | + | 0 | + | 0 | +/- | 0 | + |
| O95166 | Gamma-aminobutyric acid receptor-associated protein | GABARAP | MKFVYKEEHP | + | 0 | 0 | 0 | 0 | 0 | + | + |
| Q9UNE7 | E3 ubiquitin-protein ligase CHIP | STUB1 | MKGKEEKEGG | + | + | + | + | + | + | + | + |
| Q9NZ01 | Very-long-chain enoyl-CoA reductase | TECR | MKHYEVEILD | + | + | 0 | 0 | 0 | 0 | + | + |
| Q9Y265 | RuvB-like 1 | RUVBL1 | MKIEEVKSTT | +/- | + | + | + | + | + | + | + |
| Q9NVM4 | Protein arginine N-methyltransferase 7 | PRMT7 | MKIFCSRANP | 0 | 0 | + | + | 0 | 0 | 0 | 0 |
| Q96QD8 | Sodium-coupled neutral amino acid transporter 2 | SLC38A2 | MKKAEMGRFS | +/- | + | + | + | + | + | + | + |
| Q8IXI2 | Mitochondrial Rho GTPase 1 | RHOT1 | MKKDVRILLV | + | 0 | + | + | + | + | + | + |
| Q2M2I8 | AP2-associated protein kinase 1 | AAK1 | MKKFFDSRRE | + | + | 0 | 0 | + | + | + | + |
| Q9NSY1 | BMP-2-inducible protein kinase | BMP2K | MKKFSRMPKS | 0 | 0 | + | + | 0 | 0 | 0 | 0 |
| O14544 | Suppressor of cytokine signaling 6 | SOCS6 | MKKISLKTLR | + | 0 | 0 | 0 | 0 | 0 | 0 | 0 |
| Q4KMQ2 | Anoctamin-6 | ANO6 | MKKMSRNVLL | + | 0 | 0 | 0 | 0 | 0 | 0 | 0 |
| Q17R89 | Rho GTPase-activating protein 44 | ARHGAP44 | MKKQFNRMRQ | + | 0 | + | + | + | + | 0 | 0 |
| P84101-4 | Isoform 4 of Small EDRK-rich factor 2 | SERF2 | MKKQSDSVKG | + | 0 | 0 | 0 | + | 0 | 0 | 0 |
| Q9UHD8-7 | Isoform 7 of Septin-9 | sept-09 | MKKSYSALKR | + | 0 | 0 | 0 | 0 | 0 | 0 | 0 |
| Q9UHD8 | Septin-9 | sept-09 | MKKSYSGGTR | + | + | + | 0 | 0 | 0 | 0 | 0 |
| Q9GZR7 | ATP-dependent RNA helicase DDX24 | DDX24 | MKLKDTKSRP | + | 0 | 0 | 0 | + | + | 0 | 0 |
| O94979 | Protein transport protein Sec31A | SEC31A | MKLKEVDRTA | + | + | + | + | + | +/- | + | + |
| Q6ICB4 | Sesquipedalian-2 | FAM109B | MKLNERSVAH | 0 | 0 | 0 | 0 | 0 | + | 0 | 0 |
| P62753 | 40S ribosomal protein S6 | RPS6 | MKLNISFPAT | + | + | + | + | + | + | + | + |
| P30039 | Phenazine biosynthesis-like domain-containing protein | PBLD | MKLPIFIADA | + | 0 | 0 | 0 | 0 | 0 | 0 | 0 |
| Q99856 | AT-rich interactive domain-containing protein 3A | ARID3A | MKLQAVMETL | + | 0 | + | + | 0 | 0 | 0 | 0 |
| Q9H974 | Queuine tRNA-ribosyltransferase subunit QTRTD1 | QTRTD1 | MKLSLTKVVN | + | + | 0 | 0 | 0 | 0 | 0 | 0 |
| Q6UXN9 | WD repeat-containing protein 82 | WDR82 | MKLTDSVLRS | + | + | + | + | + | + | + | + |
| O14974 | Protein phosphatase 1 regulatory subunit 12A | PPP1R12A | MKMADAKQKR | + | + | + | 0 | + | + | + | + |
| Q2M1Z3 | Rho GTPase-activating protein 31 | ARHGAP31 | MKNKGAKQKL | 0 | 0 | 0 | 0 | + | + | 0 | 0 |
| Q9NPF2 | Carbohydrate sulfotransferase 11 | CHST11 | MKPALLEVMR | 0 | 0 | 0 | 0 | 0 | 0 | 0 | 0 |
| P30085 | UMP-CMP kinase | CMPK1 | MKPLVVFVLG | + | + | + | + | + | + | + | + |
| O60906 | Sphingomyelin phosphodiesterase 2 | SMPD2 | MKPNFSLRLR | 0 | 0 | 0 | 0 | 0 | 0 | 0 | 0 |
| P52888 | Thimet oligopeptidase | THOP1 | MKPPAACAGD | + | + | 0 | 0 | + | 0 | + | 0 |
| Q15051-3 | Isoform 3 of IQ calmodulin-binding motif-containing protein 1 | IQCB1 | MKPTGTDPRI | 0 | 0 | + | 0 | 0 | 0 | 0 | 0 |
| Q8NFH4 | Nucleoporin Nup37 | NUP37 | MKQDASRNAA | + | + | 0 | 0 | 0 | + | 0 | + |
| O43242 | 26S proteasome non-ATPase regulatory subunit 3 | PSMD3 | MKQEGSARRR | + | + | 0 | 0 | + | + | + | + |
| P54277 | PMS1 protein homolog 1 | PMS1 | MKQLPAATVR | 0 | 0 | 0 | 0 | + | 0 | 0 | 0 |
| Q7Z6M1 | Rab9 effector protein with kelch motifs | RABEPK | MKQLPVLEPG | + | + | + | 0 | + | + | 0 | + |
| Q9P0K7 | Ankycorbin | RAI14 | MKSLKAKFRK | + | + | 0 | + | + | + | + | + |
| Q9BZF9 | Uveal autoantigen with coiled-coil domains and ankyrin repeats | UACA | MKSLKSRLRR | + | + | 0 | 0 | + | + | + | 0 |
| Q53GA4 | Pleckstrin homology-like domain family A member 2 | PHLDA2 | MKSPDEVLRE | + | + | 0 | 0 | 0 | 0 | 0 | 0 |
| P32969 | 60S ribosomal protein L9 | RPL9 | MKTILSNQTV | + | + | + | + | + | + | + | + |
| P83731 | 60S ribosomal protein L24 | RPL24 | MKVELCSFSG | 0 | + | 0 | 0 | 0 | 0 | 0 | 0 |
| Q9NV06 | DDB1- and CUL4-associated factor 13 | DCAF13 | MKVKMLSRNP | + | 0 | 0 | 0 | + | 0 | 0 | 0 |
| P10746 | Uroporphyrinogen-III synthase | UROS | MKVLLLKDAK | 0 | 0 | + | + | 0 | 0 | 0 | 0 |
| Q9C0C7 | Activating molecule in BECN1-regulated autophagy protein 1 | AMBRA1 | MKVVPEKNAV | + | + | 0 | 0 | 0 | 0 | 0 | 0 |
| Q9NVJ2 | ADP-ribosylation factor-like protein 8B | ARL8B | MLALISRLLD | + | + | + | + | 0 | + | + | + |
| O75970 | Multiple PDZ domain protein | MPDZ | MLEAIDKNRA | 0 | 0 | 0 | 0 | + | 0 | 0 | 0 |
| Q8NHU6 | Tudor domain-containing protein 7 | TDRD7 | MLEGDLVSKM | + | + | + | 0 | + | 0 | + | + |
| Q9NZJ0 | Denticleless protein homolog | DTL | MLFNSVLRQP | + | + | + | + | 0 | 0 | 0 | 0 |
| Q7Z4S6 | Kinesin-like protein KIF21A | KIF21A | MLGAPDESSV | + | + | + | 0 | 0 | 0 | 0 | 0 |
| Q13216 | DNA excision repair protein ERCC-8 | ERCC8 | MLGFLSARQT | 0 | 0 | + | + | 0 | 0 | 0 | 0 |
| P06576 | ATP synthase subunit beta, mitochondrial | ATP5B | MLGFVGRVAA | 0 | 0 | + | 0 | 0 | 0 | 0 | 0 |
| Q9Y3B8 | Oligoribonuclease, mitochondrial | REXO2 | MLGGSLGSRL | 0 | 0 | 0 | 0 | 0 | 0 | 0 | 0 |
| P20248 | Cyclin-A2 | CCNA2 | MLGNSAPGPA | + | 0 | + | + | 0 | 0 | 0 | 0 |
| P53990 | IST1 homolog | IST1 | MLGSGFKAER | + | + | + | + | + | + | + | 0 |
| P42345 | Serine/threonine-protein kinase mTOR | MTOR | MLGTGPAAAT | 0 | 0 | + | + | 0 | 0 | 0 | + |
| Q96IJ6 | Mannose-1-phosphate guanyltransferase alpha | GMPPA | MLKAVILIGG | + | 0 | 0 | 0 | 0 | 0 | + | + |
| Q9C0D5 | Protein TANC1 | TANC1 | MLKAVLKKSR | + | 0 | 0 | 0 | 0 | + | 0 | 0 |
| Q14160 | Protein scribble homolog | SCRIB | MLKCIPLWRC | + | 0 | 0 | 0 | 0 | 0 | 0 | 0 |
| O14578 | Citron Rho-interacting kinase | CIT | MLKFKYGARN | 0 | 0 | + | 0 | 0 | 0 | 0 | 0 |
| Q9Y678 | Coatomer subunit gamma-1 | COPG1 | MLKKFDKKDE | + | + | + | + | 0 | + | 0 | + |
| P40306 | Proteasome subunit beta type-10 | PSMB10 | MLKPALEPRG | 0 | 0 | 0 | 0 | 0 | 0 | 0 | 0 |
| Q8WWM7 | Ataxin-2-like protein | ATXN2L | MLKPQPLQQP | + | 0 | + | + | 0 | 0 | + | + |
| Q9Y2L1 | Exosome complex exonuclease RRP44 | DIS3 | MLKSKTFLKK | + | + | + | + | + | + | + | + |
| P52747 | Zinc finger protein 143 | ZNF143 | MLLAQINRDS | 0 | 0 | + | + | 0 | 0 | 0 | 0 |
| Q99543 | DnaJ homolog subfamily C member 2 | DNAJC2 | MLLLPSAADG | + | + | + | + | + | + | + | + |
| Q15434 | RNA-binding motif, single-stranded-interacting protein 2 | RBMS2 | MLLSVTSRPG | 0 | 0 | 0 | 0 | + | 0 | 0 | 0 |
| Q5TDH0 | Protein DDI1 homolog 2 | DDI2 | MLLTVYCVRR | 0 | 0 | + | 0 | + | 0 | 0 | 0 |
| Q9NQ39 | Putative 40S ribosomal protein S10-like | RPS10P5 | MLMPKKNRIA | + | + | + | + | 0 | + | + | + |
| Q14677 | Clathrin interactor 1 | CLINT1 | MLNMWKVREL | + | + | + | + | 0 | 0 | 0 | 0 |
| Q7L1T6 | Cytochrome b5 reductase 4 | CYB5R4 | MLNVPSQSFP | + | 0 | 0 | 0 | 0 | 0 | 0 | 0 |
| Q53HI1 | Protein unc-50 homolog | UNC50 | MLPSTSVNSL | + | + | + | 0 | 0 | + | + | + |
| P11831 | Serum response factor | SRF | MLPTQAGAAA | 0 | 0 | 0 | 0 | 0 | 0 | 0 | 0 |
| O15439 | Multidrug resistance-associated protein 4 | ABCC4 | MLPVYQEVKP | + | 0 | 0 | 0 | 0 | 0 | 0 | 0 |
| O76075 | DNA fragmentation factor subunit beta | DFFB | MLQKPKSVKL | + | + | 0 | 0 | 0 | 0 | 0 | 0 |
| P67812 | Signal peptidase complex catalytic subunit SEC11A | SEC11A | MLSLDFLDDV | + | + | + | + | + | + | + | + |
| P48147 | Prolyl endopeptidase | PREP | MLSLQYPDVY | + | + | + | 0 | 0 | + | 0 | 0 |
| O43704 | Sulfotransferase family cytosolic 1B member 1 | SULT1B1 | MLSPKDILRK | 0 | 0 | 0 | 0 | + | + | 0 | 0 |
| P53621 | Coatomer subunit alpha | COPA | MLTKFETKSA | + | + | + | + | + | + | + | + |
| Q969M7 | NEDD8-conjugating enzyme UBE2F | UBE2F | MLTLASKLKR | 0 | 0 | + | + | 0 | 0 | + | 0 |
| Q9UBQ0 | Vacuolar protein sorting-associated protein 29 | VPS29 | MLVLVLGDLH | 0 | 0 | 0 | 0 | 0 | 0 | 0 | 0 |
| Q9BSV6 | tRNA-splicing endonuclease subunit Sen34 | TSEN34 | MLVVEVANGR | 0 | 0 | 0 | 0 | 0 | 0 | 0 | 0 |
| Q92575 | UBX domain-containing protein 4 | UBXN4 | MLWFQGAIPA | + | 0 | + | + | 0 | + | + | + |
| P20936 | Ras GTPase-activating protein 1 | RASA1 | MMAAEAGSEE | 0 | 0 | 0 | 0 | 0 | 0 | + | + |
| P04080 | Cystatin-B | CSTB | MMCGAPSATQ | + | 0 | 0 | + | 0 | 0 | + | + |
| P02786 | Transferrin receptor protein 1 | TFRC | MMDQARSAFS | + | 0 | 0 | 0 | 0 | + | 0 | 0 |
| P06753 | Tropomyosin alpha-3 chain | TPM3 | MMEAIKKKMQ | - | 0 | 0 | 0 | 0 | 0 | 0 | 0 |
| Q01085 | Nucleolysin TIAR | TIAL1 | MMEDDGQPRT | + | 0 | 0 | 0 | 0 | + | 0 | 0 |
| Q9UNH7 | Sorting nexin-6 | SNX6 | MMEGLDDGPD | 0 | 0 | 0 | - | - | 0 | 0 | 0 |
| P82909 | 28S ribosomal protein S36, mitochondrial | MRPS36 | MMGSKMASAS | + | 0 | + | 0 | + | + | + | + |
| Q04637-4 | Isoform C of Eukaryotic translation initiation factor 4 gamma 1 | EIF4G1 | MMIPSQISYP | - | 0 | 0 | 0 | 0 | 0 | 0 | 0 |
| Q9NZJ9 | Diphosphoinositol polyphosphate phosphohydrolase 2 | NUDT4 | MMKFKPNQTR | - | + | - | - | +/- | - | 0 | 0 |
| Q53GL0 | Pleckstrin homology domain-containing family O member 1 | PLEKHO1 | MMKKNNSAKR | 0 | 0 | 0 | 0 | 0 | 0 | - | 0 |
| O95989 | Diphosphoinositol polyphosphate phosphohydrolase 1 | NUDT3 | MMKLKSNQTR | +/- | +/- | +/- | +/- | + | - | 0 | 0 |
| P52597 | Heterogeneous nuclear ribonucleoprotein F | HNRNPF | MMLGPEGGEG | +/- | - | +/- | - | +/- | +/- | - | - |
| P31943 | Heterogeneous nuclear ribonucleoprotein H | HNRNPH1 | MMLGTEGGEG | +/- | +/- | - | - | +/- | +/- | +/- | 0 |
| P55795 | Heterogeneous nuclear ribonucleoprotein H2 | HNRNPH2 | MMLSTEGREG | + | + | + | + | + | + | + | + |
| Q9Y6M1 | Insulin-like growth factor 2 mRNA-binding protein 2 | IGF2BP2 | MMNKLYIGNL | - | - | - | 0 | - | - | 0 | - |
| Q14157 | Ubiquitin-associated protein 2-like | UBAP2L | MMTSVGTNRA | 0 | 0 | + | + | + | + | 0 | 0 |
| P53004 | Biliverdin reductase A | BLVRA | MNAEPERKFG | + | 0 | 0 | 0 | 0 | + | 0 | 0 |
| Q9BWD1 | Acetyl-CoA acetyltransferase, cytosolic | ACAT2 | MNAGSDPVVI | + | + | + | + | +/- | + | + | + |
| Q6AI12 | Ankyrin repeat domain-containing protein 40 | ANKRD40 | MNALLEQKEQ | + | + | 0 | 0 | + | + | + | 0 |
| Q96S94-3 | Isoform 3 of Cyclin-L2 | CCNL2 | MNDSLRTDVF | 0 | 0 | 0 | 0 | 0 | + | 0 | 0 |
| P62847 | 40S ribosomal protein S24 | RPS24 | MNDTVTIRTR | + | + | + | + | + | +/- | + | + |
| P14324-2 | Isoform 2 of Farnesyl pyrophosphate synthase | FDPS | MNGDQNSDVY | 0 | 0 | + | 0 | 0 | 0 | 0 | 0 |
| P17480 | Nucleolar transcription factor 1 | UBTF | MNGEADCPTD | 0 | + | 0 | 0 | 0 | 0 | 0 | 0 |
| Q92734 | Protein TFG | TFG | MNGQLDLSGK | 0 | 0 | 0 | 0 | 0 | 0 | 0 | 0 |
| P55061 | Bax inhibitor 1 | TMBIM6 | MNIFDRKINF | 0 | 0 | + | + | 0 | 0 | 0 | 0 |
| Q9BZG1 | Ras-related protein Rab-34 | RAB34 | MNILAPVRRD | 0 | 0 | 0 | 0 | 0 | + | 0 | 0 |
| Q9Y371 | Endophilin-B1 | SH3GLB1 | MNIMDFNVKK | 0 | 0 | + | 0 | 0 | 0 | 0 | 0 |
| Q16222 | UDP-N-acetylhexosamine pyrophosphorylase | UAP1 | MNINDLKLTL | + | 0 | 0 | 0 | 0 | 0 | 0 | 0 |
| Q9NPA8-2 | Isoform 2 of Transcription and mRNA export factor ENY2 | ENY2 | MNKDAQMRAA | + | 0 | 0 | 0 | 0 | 0 | 0 | 0 |
| Q9Y6E2 | Basic leucine zipper and W2 domain-containing protein 2 | BZW2 | MNKHQKPVLT | 0 | 0 | 0 | 0 | 0 | 0 | + | 0 |
| Q96GG9 | DCN1-like protein 1 | DCUN1D1 | MNKLKSSQKD | + | + | + | + | + | + | + | + |
| P29353-2 | Isoform p52Shc of SHC-transforming protein 1 | SHC1 | MNKLSGGGGR | + | 0 | 0 | 0 | + | + | 0 | 0 |
| Q9NWZ3 | Interleukin-1 receptor-associated kinase 4 | IRAK4 | MNKPITPSTY | 0 | 0 | 0 | 0 | 0 | 0 | 0 | 0 |
| Q9BW62 | Katanin p60 ATPase-containing subunit A-like 1 | KATNAL1 | MNLAEICDNA | + | 0 | 0 | 0 | + | + | 0 | 0 |
| Q8TAC1 | Rieske domain-containing protein | RFESD | MNLDGSAQDP | + | 0 | + | + | 0 | 0 | 0 | 0 |
| Q9H4L7 | SWI/SNF-related matrix-associated actin-dependent regulator of chromatin subfamily A containing DEAD/H box 1 | SMARCAD1 | MNLFNLDRFR | + | 0 | + | + | + | + | 0 | 0 |
| Q8N6R0 | Methyltransferase-like protein 13 | METTL13 | MNLLPKSSRE | 0 | 0 | + | 0 | + | 0 | 0 | 0 |
| P17980 | 26S protease regulatory subunit 6A | PSMC3 | MNLLPNIESP | + | + | 0 | + | + | + | + | 0 |
| Q93062 | RNA-binding protein with multiple splicing | RBPMS | MNNGGKAEKE | 0 | 0 | 0 | 0 | 0 | 0 | + | 0 |
| Q7L1Q6 | Basic leucine zipper and W2 domain-containing protein 1 | BZW1 | MNNQKQQKPT | + | + | + | + | + | + | + | + |
| O75486-2 | Isoform 2 of Transcription initiation protein SPT3 homolog | SUPT3H | MNNTAASPMS | + | 0 | 0 | 0 | 0 | 0 | 0 | 0 |
| Q15067 | Peroxisomal acyl-coenzyme A oxidase 1 | ACOX1 | MNPDLRRERD | 0 | 0 | 0 | 0 | + | + | 0 | 0 |
| Q14847 | LIM and SH3 domain protein 1 | LASP1 | MNPNCARCGK | + | 0 | 0 | 0 | 0 | + | 0 | 0 |
| Q9NRW3 | dU-editing enzyme APOBEC-3C | APOBEC3C | MNPQIRNPMK | 0 | 0 | 0 | 0 | 0 | 0 | 0 | 0 |
| Q96K17 | Transcription factor BTF3 homolog 4 | BTF3L4 | MNQEKLAKLQ | + | + | + | + | + | + | + | + |
| Q5T7W0 | Zinc finger protein 618 | ZNF618 | MNQPGGAAAP | + | 0 | 0 | 0 | 0 | + | 0 | 0 |
| O75146 | Huntingtin-interacting protein 1-related protein | HIP1R | MNSIKNVPAR | + | + | 0 | + | + | + | 0 | 0 |
| Q6SJ93 | Protein FAM111B | FAM111B | MNSMKTEENK | 0 | 0 | 0 | 0 | + | 0 | 0 | 0 |
| Q8TEU7 | Rap guanine nucleotide exchange factor 6 | RAPGEF6 | MNSPVDPGAR | 0 | 0 | + | + | 0 | + | 0 | 0 |
| Q9NVT9 | Armadillo repeat-containing protein 1 | ARMC1 | MNSSTSTMSE | + | + | + | 0 | + | + | 0 | 0 |
| O43715 | TP53-regulated inhibitor of apoptosis 1 | TRIAP1 | MNSVGEACTD | + | 0 | 0 | 0 | + | 0 | + | 0 |
| Q9UKN8 | General transcription factor 3C polypeptide 4 | GTF3C4 | MNTADQARVG | + | 0 | 0 | 0 | 0 | + | 0 | 0 |
| Q9HCD5 | Nuclear receptor coactivator 5 | NCOA5 | MNTAPSRPSP | + | 0 | 0 | 0 | 0 | + | 0 | + |
| Q9Y6K8 | Adenylate kinase isoenzyme 5 | AK5 | MNTNDAKEYL | 0 | 0 | 0 | 0 | + | 0 | 0 | 0 |
| P61009 | Signal peptidase complex subunit 3 | SPCS3 | MNTVLSRANS | + | 0 | + | + | + | + | 0 | 0 |
| Q5T3I0 | G patch domain-containing protein 4 | GPATCH4 | MNVTPEVKSR | + | + | 0 | 0 | + | 0 | 0 | 0 |
| Q86XP3 | ATP-dependent RNA helicase DDX42 | DDX42 | MNWNKGGPGT | + | + | + | + | 0 | + | 0 | 0 |
| Q9UPQ3 | Arf-GAP with GTPase, ANK repeat and PH domain-containing protein 1 | AGAP1 | MNYQQQLANS | + | + | 0 | 0 | 0 | 0 | 0 | 0 |
| Q9H1I8 | Activating signal cointegrator 1 complex subunit 2 | ASCC2 | MPALPLDQLQ | 0 | 0 | - | - | 0 | - | 0 | 0 |
| O95059 | Ribonuclease P protein subunit p14 | RPP14 | MPAPAATYER | - | - | - | - | - | - | 0 | 0 |
| Q9P0J1 | [Pyruvate dehydrogenase [acetyl-transferring]]-phosphatase 1, mitochondrial | PDP1 | MPAPTQLFFP | - | 0 | 0 | 0 | 0 | 0 | 0 | 0 |
| Q9NWS8 | Required for meiotic nuclear division protein 1 homolog | RMND1 | MPATLLRAVA | 0 | + | 0 | 0 | 0 | 0 | 0 | 0 |
| Q8NEU8 | DCC-interacting protein 13-beta | APPL2 | MPAVDKLLLE | 0 | 0 | 0 | - | - | - | - | - |
| Q9NPF4 | Probable tRNA N6-adenosine threonylcarbamoyltransferase | OSGEP | MPAVLGFEGS | 0 | 0 | - | - | 0 | - | 0 | 0 |
| O94973 | AP-2 complex subunit alpha-2 | AP2A2 | MPAVSKGDGM | - | - | - | - | - | - | - | - |
| Q9BXJ9 | N-alpha-acetyltransferase 15, NatA auxiliary subunit | NAA15 | MPAVSLPPKE | - | - | - | - | - | - | - | - |
| P33316-2 | Deoxyuridine 5'-triphosphate nucleotidohydrolase | DUT | MPCSEETPAI | - | - | - | - | - | - | - | - |
| Q9NX47 | E3 ubiquitin-protein ligase MARCH5 | MARCH5 | MPDQALQQML | - | - | - | - | - | - | - | - |
| Q9UJ83 | 2-hydroxyacyl-CoA lyase 1 | HACL1 | MPDSNFAERS | 0 | 0 | 0 | 0 | 0 | - | 0 | 0 |
| P35998 | 26S protease regulatory subunit 7 | PSMC2 | MPDYLGADQR | - | - | - | - | - | - | - | - |
| Q658P3 | Metalloreductase STEAP3 | STEAP3 | MPEEMDKPLI | 0 | 0 | 0 | 0 | 0 | 0 | 0 | 0 |
| P42166 | Lamina-associated polypeptide 2, isoform alpha | TMPO | MPEFLEDPSV | - | 0 | - | - | 0 | 0 | 0 | - |
| P09960 | Leukotriene A-4 hydrolase | LTA4H | MPEIVDTCSL | - | - | 0 | 0 | 0 | - | - | 0 |
| P51665 | 26S proteasome non-ATPase regulatory subunit 7 | PSMD7 | MPELAVQKVV | 0 | 0 | 0 | 0 | 0 | 0 | 0 | 0 |
| P50991 | T-complex protein 1 subunit delta | CCT4 | MPENVAPRSG | - | - | 0 | - | - | - | - | - |
| Q7Z434 | Mitochondrial antiviral-signaling protein | MAVS | MPFAEDKTYK | - | 0 | 0 | 0 | - | 0 | - | 0 |
| Q15678 | Tyrosine-protein phosphatase non-receptor type 14 | PTPN14 | MPFGLKLRRT | 0 | 0 | 0 | 0 | - | - | - | 0 |
| O43920 | NADH dehydrogenase [ubiquinone] iron-sulfur protein 5 | NDUFS5 | MPFLDIQKRF | 0 | - | - | - | - | 0 | - | 0 |
| A6NHG4 | D-dopachrome decarboxylase-like protein | DDTL | MPFLELDTNL | - | - | - | - | - | - | - | - |
| Q7L8A9 | Vasohibin-1 | VASH1 | MPGGKKVAGG | 0 | 0 | 0 | 0 | - | - | 0 | 0 |
| P30041 | Peroxiredoxin-6 | PRDX6 | MPGGLLLGDV | - | - | - | - | - | - | - | - |
| Q9UKG1 | DCC-interacting protein 13-alpha | APPL1 | MPGIDKLPIE | - | - | 0 | 0 | - | - | - | - |
| Q86YS7 | C2 domain-containing protein 5 | C2CD5 | MPGKLKVKIV | - | 0 | 0 | 0 | 0 | 0 | 0 | 0 |
| Q86SK9 | Stearoyl-CoA desaturase 5 | SCD5 | MPGPATDAGK | - | - | 0 | 0 | - | - | 0 | - |
| P60468 | Protein transport protein Sec61 subunit beta | SEC61B | MPGPTPSGTN | - | - | - | - | - | - | - | - |
| P49770 | Translation initiation factor eIF-2B subunit beta | EIF2B2 | MPGSAAKGSE | - | - | - | - | - | - | - | - |
| Q9ULS5 | Transmembrane and coiled-coil domains protein 3 | TMCC3 | MPGSDTALTV | 0 | 0 | 0 | 0 | - | - | 0 | 0 |
| P39019 | 40S ribosomal protein S19 | RPS19 | MPGVTVKDVN | - | - | - | - | - | - | - | - |
| P09972 | Fructose-bisphosphate aldolase C | ALDOC | MPHSYPALSA | - | - | 0 | 0 | 0 | 0 | - | 0 |
| Q5JSJ4 | Protein DDX26B | DDX26B | MPILLFLIDT | 0 | 0 | - | - | 0 | 0 | 0 | 0 |
| Q9Y496 | Kinesin-like protein KIF3A | KIF3A | MPINKSEKPE | 0 | - | 0 | 0 | 0 | - | - | 0 |
| Q9Y3C1 | Nucleolar protein 16 | NOP16 | MPKAKGKTRR | - | 0 | 0 | 0 | - | - | 0 | 0 |
| Q96EY4 | Translation machinery-associated protein 16 | TMA16 | MPKAPKGKSA | - | - | - | - | - | - | - | - |
| P50238 | Cysteine-rich protein 1 | CRIP1 | MPKCPKCNKE | - | 0 | 0 | 0 | 0 | 0 | 0 | 0 |
| Q9ULM6 | CCR4-NOT transcription complex subunit 6 | CNOT6 | MPKEKYEPPD | - | 0 | - | 0 | 0 | 0 | 0 | 0 |
| Q13895 | Bystin | BYSL | MPKFKAARGV | - | - | - | - | - | - | - | 0 |
| P62424 | 60S ribosomal protein L7a | RPL7A | MPKGKKAKGK | - | - | 0 | 0 | - | 0 | 0 | 0 |
| Q96CT7 | Coiled-coil domain-containing protein 124 | CCDC124 | MPKKFQGENT | - | - | - | - | - | - | - | - |
| Q9Y3S2 | Zinc finger protein 330 | ZNF330 | MPKKKTGARK | - | - | 0 | 0 | - | - | - | - |
| Q6P1R4 | tRNA-dihydrouridine(16/17) synthase [NAD(P)(+)]-like | DUS1L | MPKLQGFEFW | - | - | 0 | 0 | 0 | 0 | 0 | 0 |
| O14602 | Eukaryotic translation initiation factor 1A, Y-chromosomal | EIF1AY | MPKNKGKGGK | - | - | - | - | - | - | - | - |
| O00458 | Interferon-related developmental regulator 1 | IFRD1 | MPKNKKRNTP | - | 0 | 0 | 0 | 0 | - | 0 | 0 |
| Q9H1V8 | Sodium-dependent neutral amino acid transporter SLC6A17 | SLC6A17 | MPKNSKVTQR | - | 0 | 0 | 0 | 0 | 0 | 0 | 0 |
| Q9H2J7 | Sodium-dependent neutral amino acid transporter B(0)AT2 | SLC6A15 | MPKNSKVVKR | 0 | 0 | 0 | 0 | 0 | 0 | - | - |
| P15311 | Ezrin | EZR | MPKPINVRVT | 0 | 0 | - | - | 0 | 0 | - | 0 |
| Q15651 | High mobility group nucleosome-binding domain-containing protein 3 | HMGN3 | MPKRKSPENT | 0 | 0 | - | 0 | 0 | 0 | 0 | 0 |
| P53999 | Activated RNA polymerase II transcriptional coactivator p15 | SUB1 | MPKSKELVSS | - | - | - | - | 0 | 0 | - | - |
| P26038 | Moesin | MSN | MPKTISVRVT | - | - | - | - | - | - | - | - |
| Q9UNQ2 | Probable dimethyladenosine transferase | DIMT1 | MPKVKSGAIG | - | - | - | - | - | - | 0 | 0 |
| P42677 | 40S ribosomal protein S27 | RPS27 | MPLAKDLLHP | - | - | 0 | 0 | - | - | - | - |
| Q9NR45 | Sialic acid synthase | NANS | MPLELELCPG | - | - | - | - | - | - | - | - |
| Q15008 | 26S proteasome non-ATPase regulatory subunit 6 | PSMD6 | MPLENLEEEG | - | - | - | - | - | - | - | - |
| O14908 | PDZ domain-containing protein GIPC1 | GIPC1 | MPLGLGRRKK | - | 0 | 0 | 0 | 0 | - | - | 0 |
| Q9UKV5 | E3 ubiquitin-protein ligase AMFR | AMFR | MPLLFLERFP | - | - | - | - | - | 0 | - | - |
| O60671 | Cell cycle checkpoint protein RAD1 | RAD1 | MPLLTQQIQD | 0 | 0 | - | - | 0 | 0 | 0 | 0 |
| Q96CP2 | FLYWCH family member 2 | FLYWCH2 | MPLPEPSEQE | 0 | 0 | 0 | 0 | 0 | 0 | - | 0 |
| O60828 | Polyglutamine-binding protein 1 | PQBP1 | MPLPVALQTR | - | - | - | - | - | - | - | 0 |
| Q96EK9 | Protein KTI12 homolog | KTI12 | MPLVVFCGLP | 0 | 0 | 0 | 0 | 0 | 0 | - | 0 |
| P14174 | Macrophage migration inhibitory factor | MIF | MPMFIVNTNV | - | - | - | - | - | - | - | - |
| P29373 | Cellular retinoic acid-binding protein 2 | CRABP2 | MPNFSGNWKI | - | - | 0 | 0 | 0 | 0 | 0 | - |
| P23469-3 | Isoform 3 of Receptor-type tyrosine-protein phosphatase epsilon | PTPRE | MPNGILEEQE | 0 | 0 | 0 | 0 | - | - | 0 | 0 |
| P21108 | Ribose-phosphate pyrophosphokinase 3 | PRPS1L1 | MPNIKIFSGS | - | - | 0 | 0 | - | 0 | 0 | 0 |
| P11908 | Ribose-phosphate pyrophosphokinase 2 | PRPS2 | MPNIVLFSGS | 0 | - | 0 | 0 | 0 | 0 | - | 0 |
| Q13362-4 | Isoform 4 of Serine/threonine-protein phosphatase 2A 56 kDa regulatory subunit gamma isoform | PPP2R5C | MPNKNKKEKE | 0 | 0 | - | 0 | 0 | 0 | 0 | 0 |
| P23381 | Tryptophan--tRNA ligase, cytoplasmic | WARS | MPNSEPASLL | - | 0 | - | - | 0 | - | 0 | 0 |
| Q9NQW7 | Xaa-Pro aminopeptidase 1 | XPNPEP1 | MPPKVTSELL | - | - | - | - | - | - | - | - |
| P82980 | Retinol-binding protein 5 | RBP5 | MPPNLTGYYR | 0 | 0 | - | - | 0 | 0 | 0 | 0 |
| P09211 | Glutathione S-transferase P | GSTP1 | MPPYTVVYFP | +/- | +/- | +/- | +/- | +/- | +/- | +/- | +/- |
| P49458 | Signal recognition particle 9 kDa protein | SRP9 | MPQYQTWEEF | - | - | - | - | - | - | - | 0 |
| P14868 | Aspartate--tRNA ligase, cytoplasmic | DARS | MPSASASRKS | - | - | 0 | 0 | 0 | 0 | 0 | 0 |
| Q5VV42-3 | Isoform 3 of Threonylcarbamoyladenosine tRNA methylthiotransferase | CDKAL1 | MPSASCDTLL | 0 | 0 | - | - | 0 | 0 | 0 | 0 |
| Q9UG63 | ATP-binding cassette sub-family F member 2 | ABCF2 | MPSDLAKKKA | - | - | - | - | - | - | - | - |
| A6NCE7 | Microtubule-associated proteins 1A/1B light chain 3 beta 2 | MAP1LC3B2 | MPSEKTFKQR | - | - | - | - | - | - | - | - |
| P62249 | 40S ribosomal protein S16 | RPS16 | MPSKGPLQSV | - | - | - | - | - | - | - | - |
| Q14919 | Dr1-associated corepressor | DRAP1 | MPSKKKKYNA | - | - | - | - | - | - | - | - |
| Q6ZN04 | RNA-binding protein MEX3B | MEX3B | MPSSLFADLE | 0 | 0 | - | - | 0 | 0 | 0 | 0 |
| O75694 | Nuclear pore complex protein Nup155 | NUP155 | MPSSLLGAAM | - | - | - | - | - | - | - | - |
| Q9Y2I8 | WD repeat-containing protein 37 | WDR37 | MPTESASCST | - | 0 | 0 | 0 | 0 | - | 0 | 0 |
| Q96QK1 | Vacuolar protein sorting-associated protein 35 | VPS35 | MPTTQQSPQD | - | 0 | - | - | 0 | - | - | - |
| Q9BZZ5 | Apoptosis inhibitor 5 | API5 | MPTVEELYRN | - | - | - | - | - | - | - | - |
| Q9NSD9 | Phenylalanine--tRNA ligase beta subunit | FARSB | MPTVSVKRDL | - | - | - | - | - | - | 0 | 0 |
| P04818 | Thymidylate synthase | TYMS | MPVAGSELPR | - | - | - | - | - | - | - | - |
| P60602 | Reactive oxygen species modulator 1 | ROMO1 | MPVAVGPYGQ | 0 | 0 | - | - | 0 | - | - | - |
| P33908 | Mannosyl-oligosaccharide 1,2-alpha-mannosidase IA | MAN1A1 | MPVGGLLPLF | - | 0 | - | - | 0 | - | - | - |
| O75369 | Filamin-B | FLNB | MPVTEKDLAE | 0 | 0 | - | - | 0 | - | - | - |
| P19387 | DNA-directed RNA polymerase II subunit RPB3 | POLR2C | MPYANQPTVR | 0 | 0 | - | - | - | - | 0 | 0 |
| O75083 | WD repeat-containing protein 1 | WDR1 | MPYEIKKVFA | - | 0 | - | - | - | - | - | - |
| P30047 | GTP cyclohydrolase 1 feedback regulatory protein | GCHFR | MPYLLISTQI | 0 | 0 | - | 0 | 0 | 0 | 0 | 0 |
| P04075 | Fructose-bisphosphate aldolase A | ALDOA | MPYQYPALTP | - | - | - | - | - | - | - | - |
| P16157 | Ankyrin-1 | ANK1 | MPYSVGFREA | 0 | 0 | - | - | 0 | 0 | 0 | 0 |
| Q9BSY4 | Coiled-coil-helix-coiled-coil-helix domain-containing protein 5 | CHCHD5 | MQAALEVTAR | 0 | 0 | 0 | 0 | + | 0 | 0 | 0 |
| P35249 | Replication factor C subunit 4 | RFC4 | MQAFLKGTSI | + | + | + | + | + | 0 | + | + |
| O43708 | Maleylacetoacetate isomerase | GSTZ1 | MQAGKPILYS | 0 | + | 0 | 0 | 0 | 0 | 0 | 0 |
| Q8NC01 | C-type lectin domain family 1 member A | CLEC1A | MQAKYSSTRD | 0 | 0 | 0 | 0 | + | + | 0 | 0 |
| P55884 | Eukaryotic translation initiation factor 3 subunit B | EIF3B | MQDAENVAVP | +/- | + | + | + | + | + | + | + |
| Q9H2J4 | Phosducin-like protein 3 | PDCL3 | MQDPNADTEW | 0 | 0 | + | + | 0 | 0 | 0 | 0 |
| Q8NHV4 | Protein NEDD1 | NEDD1 | MQENLRFASS | 0 | 0 | 0 | 0 | 0 | 0 | 0 | 0 |
| Q9Y6D5 | Brefeldin A-inhibited guanine nucleotide-exchange protein 2 | ARFGEF2 | MQESQTKSMF | 0 | 0 | 0 | 0 | + | 0 | 0 | 0 |
| Q96JG6 | Coiled-coil domain-containing protein 132 | CCDC132 | MQKIKSLMTR | 0 | + | + | 0 | + | 0 | 0 | 0 |
| Q53QZ3 | Rho GTPase-activating protein 15 | ARHGAP15 | MQKSTNSDTS | 0 | 0 | 0 | + | 0 | 0 | 0 | 0 |
| Q00535 | Cyclin-dependent kinase 5 | CDK5 | MQKYEKLEKI | 0 | 0 | + | + | 0 | 0 | 0 | 0 |
| P09936 | Ubiquitin carboxyl-terminal hydrolase isozyme L1 | UCHL1 | MQLKPMEINP | 0 | 0 | 0 | 0 | + | + | + | + |
| P11441 | Ubiquitin-like protein 4A | UBL4A | MQLTVKALQG | + | 0 | 0 | 0 | + | 0 | + | 0 |
| P63220 | 40S ribosomal protein S21 | RPS21 | MQNDAGEFVD | + | + | + | + | + | + | + | + |
| Q15185 | Prostaglandin E synthase 3 | PTGES3 | MQPASAKWYD | + | + | + | + | + | + | + | + |
| O43315 | Aquaporin-9 | AQP9 | MQPEGAEKGK | 0 | 0 | 0 | 0 | 0 | 0 | + | + |
| Q9BXJ8 | Transmembrane protein 120A | TMEM120A | MQPPPPGPLG | 0 | 0 | 0 | 0 | + | + | 0 | 0 |
| Q8NEM7 | Transcription factor SPT20 homolog | SUPT20H | MQQALELALD | + | 0 | 0 | 0 | 0 | 0 | 0 | 0 |
| Q15233 | Non-POU domain-containing octamer-binding protein | NONO | MQSNKTFNLE | + | + | 0 | 0 | 0 | 0 | + | 0 |
| Q53T59 | HCLS1-binding protein 3 | HS1BP3 | MQSPAVLVTS | + | + | + | + | + | + | + | + |
| Q13572 | Inositol-tetrakisphosphate 1-kinase | ITPK1 | MQTFLKGKRV | 0 | + | 0 | 0 | 0 | 0 | 0 | 0 |
| Q9H840 | Gem-associated protein 7 | GEMIN7 | MQTPVNIPVP | + | + | + | + | + | + | 0 | 0 |
| Q9ULA0 | Aspartyl aminopeptidase | DNPEP | MQVAMNGKAR | + | 0 | + | + | + | + | + | 0 |
| Q7Z5G4 | Golgin subfamily A member 7 | GOLGA7 | MRPQQAPVSG | + | + | 0 | 0 | 0 | 0 | 0 | 0 |
| P46940 | Ras GTPase-activating-like protein IQGAP1 | IQGAP1 | MSAADEVDGL | - | 0 | 0 | - | - | - | - | 0 |
| Q9BRF8 | Calcineurin-like phosphoesterase domain-containing protein 1 | CPPED1 | MSAAEAGGVF | 0 | 0 | 0 | - | 0 | 0 | 0 | 0 |
| Q6WCQ1 | Myosin phosphatase Rho-interacting protein | MPRIP | MSAAKENPCR | - | 0 | 0 | 0 | - | - | 0 | - |
| Q9H1B7 | Interferon regulatory factor 2-binding protein-like | IRF2BPL | MSAAQVSSSR | - | 0 | 0 | 0 | 0 | 0 | 0 | - |
| P53350 | Serine/threonine-protein kinase PLK1 | PLK1 | MSAAVTAGKL | - | - | - | - | - | - | - | - |
| O14745 | Na(+)/H(+) exchange regulatory cofactor NHE-RF1 | SLC9A3R1 | MSADAAAGAP | - | - | - | - | - | - | - | - |
| O60664 | Perilipin-3 | PLIN3 | MSADGAEADG | 0 | - | - | - | 0 | 0 | - | - |
| P27986 | Phosphatidylinositol 3-kinase regulatory subunit alpha | PIK3R1 | MSAEGYQYRA | 0 | 0 | 0 | 0 | 0 | 0 | 0 | 0 |
| P30519 | Heme oxygenase 2 | HMOX2 | MSAEVETSEG | 0 | 0 | - | - | - | - | - | - |
| P56211 | cAMP-regulated phosphoprotein 19 | ARPP19 | MSAEVPEAAS | 0 | 0 | 0 | 0 | 0 | 0 | - | 0 |
| Q9H0N0 | Ras-related protein Rab-6C | RAB6C | MSAGGDFGNP | - | - | - | - | - | - | 0 | 0 |
| Q9HD26 | Golgi-associated PDZ and coiled-coil motif-containing protein | GOPC | MSAGGPCPAA | - | 0 | 0 | 0 | - | - | 0 | 0 |
| P53396 | ATP-citrate synthase | ACLY | MSAKAISEQT | - | 0 | - | - | 0 | - | - | 0 |
| Q9BQT8 | Mitochondrial 2-oxodicarboxylate carrier | SLC25A21 | MSAKPEVSLV | 0 | 0 | - | - | 0 | 0 | 0 | 0 |
| Q8NFX7 | Syntaxin-binding protein 6 | STXBP6 | MSAKSAISKE | 0 | 0 | - | 0 | 0 | 0 | - | - |
| Q6NW29 | RWD domain-containing protein 4 | RWDD4 | MSANEDQEME | 0 | 0 | 0 | 0 | - | - | - | 0 |
| Q01105-2 | Isoform 2 of Protein SET | SET | MSAPAAKVSK | - | 0 | 0 | 0 | - | 0 | 0 | - |
| Q8WVJ2 | NudC domain-containing protein 2 | NUDCD2 | MSAPFEERSG | - | 0 | 0 | - | 0 | - | 0 | 0 |
| P80217 | Interferon-induced 35 kDa protein | IFI35 | MSAPLDAALH | 0 | 0 | 0 | 0 | 0 | 0 | - | - |
| Q14353 | Guanidinoacetate N-methyltransferase | GAMT | MSAPSATPIF | 0 | 0 | - | 0 | 0 | 0 | 0 | 0 |
| Q8TD16 | Protein bicaudal D homolog 2 | BICD2 | MSAPSEEEEY | 0 | 0 | 0 | 0 | 0 | 0 | 0 | - |
| Q9NQ29 | Putative RNA-binding protein Luc7-like 1 | LUC7L | MSAQAQMRAL | 0 | 0 | 0 | 0 | 0 | - | 0 | 0 |
| Q9NVR2 | Integrator complex subunit 10 | INTS10 | MSAQGDCEFL | 0 | - | 0 | - | 0 | 0 | 0 | 0 |
| P35659 | Protein DEK | DEK | MSASAPAAEG | 0 | 0 | - | - | 0 | 0 | - | - |
| Q9Y5A9 | YTH domain-containing family protein 2 | YTHDF2 | MSASSLLEQR | - | 0 | - | - | - | - | 0 | 0 |
| P61803 | Dolichyl-diphosphooligosaccharide--protein glycosyltransferase subunit DAD1 | DAD1 | MSASVVSVIS | - | - | - | - | - | - | - | - |
| Q9BYJ9 | YTH domain-containing family protein 1 | YTHDF1 | MSATSVDTQR | - | 0 | 0 | 0 | - | - | 0 | - |
| P04183 | Thymidine kinase, cytosolic | TK1 | MSCINLPTVL | - | - | - | - | 0 | 0 | - | 0 |
| P06454 | Prothymosin alpha | PTMA | MSDAAVDTSS | - | - | - | - | - | - | - | - |
| P26368 | Splicing factor U2AF 65 kDa subunit | U2AF2 | MSDFDEFERQ | - | - | - | - | - | - | - | - |
| O15126 | Secretory carrier-associated membrane protein 1 | SCAMP1 | MSDFDSNPFA | 0 | 0 | - | - | 0 | 0 | - | - |
| Q9BUN8 | Derlin-1 | DERL1 | MSDIGDWFRS | - | - | - | - | 0 | - | - | - |
| Q9UPM8 | AP-4 complex subunit epsilon-1 | AP4E1 | MSDIVEKTLT | 0 | 0 | - | 0 | 0 | 0 | 0 | 0 |
| P23526 | Adenosylhomocysteinase | AHCY | MSDKLPYKVA | - | - | - | - | - | - | - | - |
| P0CG34 | Thymosin beta-15A | TMSB15A | MSDKPDLSEV | 0 | 0 | 0 | 0 | - | 0 | - | 0 |
| P62328 | Thymosin beta-4 | TMSB4X | MSDKPDMAEI | 0 | 0 | 0 | 0 | - | 0 | 0 | 0 |
| Q13409 | Cytoplasmic dynein 1 intermediate chain 2 | DYNC1I2 | MSDKSELKAE | - | - | - | - | - | - | - | - |
| Q13177 | Serine/threonine-protein kinase PAK 2 | PAK2 | MSDNGELEDK | - | - | - | - | - | - | - | - |
| Q9Y5P4 | Collagen type IV alpha-3-binding protein | COL4A3BP | MSDNQSWNSS | 0 | 0 | 0 | 0 | 0 | 0 | 0 | - |
| Q6ZW49 | PAX-interacting protein 1 | PAXIP1 | MSDQAPKVPE | 0 | 0 | 0 | 0 | 0 | 0 | 0 | 0 |
| P47756 | F-actin-capping protein subunit beta | CAPZB | MSDQQLDCAL | - | - | - | - | - | - | - | - |
| O00267 | Transcription elongation factor SPT5 | SUPT5H | MSDSEDSNFS | 0 | 0 | - | - | 0 | 0 | 0 | 0 |
| P62136 | Serine/threonine-protein phosphatase PP1-alpha catalytic subunit | PPP1CA | MSDSEKLNLD | - | - | - | - | - | - | - | - |
| P62995 | Transformer-2 protein homolog beta | TRA2B | MSDSGEQNYG | 0 | 0 | 0 | 0 | 0 | - | 0 | 0 |
| O60234 | Glia maturation factor gamma | GMFG | MSDSLVVCEV | 0 | 0 | - | - | - | 0 | 0 | 0 |
| Q9UMY4 | Sorting nexin-12 | SNX12 | MSDTAVADTR | - | 0 | 0 | 0 | - | - | 0 | - |
| Q15813 | Tubulin-specific chaperone E | TBCE | MSDTLTADVI | - | - | - | - | - | - | - | - |
| Q9UNF1 | Melanoma-associated antigen D2 | MAGED2 | MSDTSESGAG | - | - | 0 | 0 | - | - | - | - |
| Q92945 | Far upstream element-binding protein 2 | KHSRP | MSDYSTGGPP | - | - | - | - | 0 | - | - | - |
| Q9HDC9 | Adipocyte plasma membrane-associated protein | APMAP | MSEADGLRQR | - | 0 | 0 | 0 | 0 | 0 | 0 | 0 |
| P56962 | Syntaxin-17 | STX17 | MSEDEEKVKL | - | 0 | - | 0 | 0 | 0 | 0 | 0 |
| P07384 | Calpain-1 catalytic subunit | CAPN1 | MSEEIITPVY | - | - | - | - | 0 | - | - | - |
| P35612 | Beta-adducin | ADD2 | MSEETVPEAA | 0 | 0 | - | 0 | 0 | 0 | 0 | 0 |
| O15258 | Protein RER1 | RER1 | MSEGDSVGES | - | - | 0 | 0 | - | 0 | - | - |
| P05423 | DNA-directed RNA polymerase III subunit RPC4 | POLR3D | MSEGNAAGEP | - | 0 | 0 | 0 | 0 | 0 | 0 | 0 |
| Q4ZIN3 | Membralin | TMEM259 | MSEHVEPAAP | - | 0 | 0 | 0 | 0 | 0 | 0 | - |
| Q14191 | Werner syndrome ATP-dependent helicase | WRN | MSEKKLETTA | - | - | - | - | - | - | - | 0 |
| P20962 | Parathymosin | PTMS | MSEKSVEAAA | - | - | - | - | - | - | - | - |
| P62873 | Guanine nucleotide-binding protein G(I)/G(S)/G(T) subunit beta-1 | GNB1 | MSELDQLRQE | - | - | - | - | - | - | - | - |
| Q9HAV0 | Guanine nucleotide-binding protein subunit beta-4 | GNB4 | MSELEQLRQE | 0 | - | - | - | - | 0 | - | - |
| Q15527 | Surfeit locus protein 2 | SURF2 | MSELPGDVRA | 0 | 0 | - | 0 | 0 | 0 | 0 | 0 |
| Q14204 | Cytoplasmic dynein 1 heavy chain 1 | DYNC1H1 | MSEPGGGGGE | 0 | 0 | - | 0 | 0 | 0 | - | 0 |
| Q08209 | Serine/threonine-protein phosphatase 2B catalytic subunit alpha isoform | PPP3CA | MSEPKAIDPK | - | - | - | - | - | - | - | - |
| Q8ND71 | GTPase IMAP family member 8 | GIMAP8 | MSEQSCQMSE | 0 | 0 | 0 | 0 | - | - | 0 | 0 |
| Q8N8S7 | Protein enabled homolog | ENAH | MSEQSICQAR | - | - | 0 | 0 | - | - | - | - |
| O94913 | Pre-mRNA cleavage complex 2 protein Pcf11 | PCF11 | MSEQTPAEAG | - | 0 | 0 | 0 | 0 | - | 0 | 0 |
| Q5TGZ0 | MICOS complex subunit MIC10 | MINOS1 | MSESELGRKW | 0 | 0 | 0 | 0 | 0 | - | 0 | 0 |
| P60983 | Glia maturation factor beta | GMFB | MSESLVVCDV | 0 | 0 | - | - | - | - | - | 0 |
| P17096 | High mobility group protein HMG-I/HMG-Y | HMGA1 | MSESSSKSSQ | - | - | - | - | - | - | - | - |
| P10412 | Histone H1.4 | HIST1H1E | MSETAPAAPA | - | 0 | - | - | - | - | - | - |
| P16403 | Histone H1.2 | HIST1H1C | MSETAPAAPA | 0 | 0 | 0 | 0 | 0 | 0 | - | - |
| P16401 | Histone H1.5 | HIST1H1B | MSETAPAETA | 0 | 0 | 0 | 0 | - | 0 | - | - |
| P16402 | Histone H1.3 | HIST1H1D | MSETAPLAPT | 0 | 0 | - | 0 | 0 | 0 | 0 | - |
| P41212 | Transcription factor ETV6 | ETV6 | MSETPAQCSI | 0 | 0 | - | 0 | 0 | - | 0 | 0 |
| P50552 | Vasodilator-stimulated phosphoprotein | VASP | MSETVICSSR | - | - | 0 | 0 | - | - | - | - |
| P32321 | Deoxycytidylate deaminase | DCTD | MSEVSCKKRD | - | - | 0 | 0 | - | - | - | - |
| Q8N6S5 | ADP-ribosylation factor-like protein 6-interacting protein 6 | ARL6IP6 | MSFAESGWRS | 0 | 0 | - | 0 | 0 | - | 0 | 0 |
| Q9H2G2 | STE20-like serine/threonine-protein kinase | SLK | MSFFNFRKIF | 0 | 0 | 0 | - | 0 | 0 | 0 | 0 |
| P16930 | Fumarylacetoacetase | FAH | MSFIPVAEDS | 0 | 0 | 0 | 0 | 0 | 0 | - | 0 |
| Q7L9L4 | MOB kinase activator 1B | MOB1B | MSFLFGSRSS | 0 | 0 | - | - | - | - | 0 | 0 |
| Q9H8S9 | MOB kinase activator 1A | MOB1A | MSFLFSSRSS | - | 0 | - | - | - | - | - | 0 |
| O75436 | Vacuolar protein sorting-associated protein 26A | VPS26A | MSFLGGFFGP | 0 | 0 | 0 | 0 | 0 | 0 | 0 | 0 |
| P54105 | Methylosome subunit pICln | CLNS1A | MSFLKSFPPP | - | - | - | - | 0 | - | - | - |
| Q7RTN6 | STE20-related kinase adapter protein alpha | STRADA | MSFLVSKPER | 0 | 0 | 0 | 0 | - | 0 | 0 | 0 |
| O75330 | Hyaluronan mediated motility receptor | HMMR | MSFPKAPLKR | 0 | 0 | - | - | 0 | 0 | 0 | 0 |
| O95168 | NADH dehydrogenase [ubiquinone] 1 beta subcomplex subunit 4 | NDUFB4 | MSFPKYKPSS | - | 0 | - | - | - | - | 0 | 0 |
| P40692 | DNA mismatch repair protein Mlh1 | MLH1 | MSFVAGVIRR | 0 | 0 | - | - | 0 | - | - | - |
| O60825 | 6-phosphofructo-2-kinase/fructose-2,6-bisphosphatase 2 | PFKFB2 | MSGASSSEQN | - | - | - | - | 0 | 0 | 0 | 0 |
| O60333 | Kinesin-like protein KIF1B | KIF1B | MSGASVKVAV | 0 | - | 0 | 0 | 0 | - | 0 | 0 |
| Q9UQ80 | Proliferation-associated protein 2G4 | PA2G4 | MSGEDEQQEQ | - | 0 | - | - | - | 0 | - | - |
| Q9BZL4 | Protein phosphatase 1 regulatory subunit 12C | PPP1R12C | MSGEDGPAAG | - | 0 | 0 | 0 | - | - | 0 | 0 |
| Q15102 | Platelet-activating factor acetylhydrolase IB subunit gamma | PAFAH1B3 | MSGEENPASK | - | - | - | - | - | - | - | - |
| Q9H9B4 | Sideroflexin-1 | SFXN1 | MSGELPPNIN | - | - | - | - | - | - | - | - |
| Q9BRT3 | Migration and invasion enhancer 1 | MIEN1 | MSGEPGQTSV | - | 0 | - | - | 0 | 0 | - | - |
| P33992 | DNA replication licensing factor MCM5 | MCM5 | MSGFDDPGIF | - | - | - | - | - | - | - | - |
| Q6DKJ4 | Nucleoredoxin | NXN | MSGFLEELLG | 0 | - | 0 | 0 | 0 | 0 | 0 | 0 |
| O95807 | Transmembrane protein 50A | TMEM50A | MSGFLEGLRC | - | - | - | - | - | - | - | - |
| Q15181 | Inorganic pyrophosphatase | PPA1 | MSGFSTEERA | - | - | 0 | 0 | - | - | - | - |
| Q8TDH9 | Biogenesis of lysosome-related organelles complex 1 subunit 5 | BLOC1S5 | MSGGGTETPV | 0 | 0 | - | - | - | 0 | 0 | 0 |
| Q07955 | Serine/arginine-rich splicing factor 1 | SRSF1 | MSGGGVIRGP | 0 | 0 | 0 | - | 0 | 0 | 0 | 0 |
| Q03135 | Caveolin-1 | CAV1 | MSGGKYVDSE | - | - | 0 | 0 | - | - | - | - |
| P52298 | Nuclear cap-binding protein subunit 2 | NCBP2 | MSGGLLKALR | - | - | - | - | - | - | - | - |
| P63279 | SUMO-conjugating enzyme UBC9 | UBE2I | MSGIALSRLA | - | - | 0 | - | - | - | - | 0 |
| Q5VTR2 | E3 ubiquitin-protein ligase BRE1A | RNF20 | MSGIGNKRAA | - | 0 | 0 | 0 | 0 | - | 0 | 0 |
| Q6ZS86-2 | Isoform 2 of Putative glycerol kinase 5 | GK5 | MSGLLTDPEQ | 0 | 0 | 0 | 0 | - | - | 0 | 0 |
| Q9NSK0 | Kinesin light chain 4 | KLC4 | MSGLVLGQRD | 0 | 0 | - | - | 0 | - | 0 | 0 |
| P08397 | Porphobilinogen deaminase | HMBS | MSGNGNAAAT | - | - | - | - | - | 0 | - | 0 |
| O00154-2 | Isoform 2 of Cytosolic acyl coenzyme A thioester hydrolase | ACOT7 | MSGPDVETPS | - | - | - | - | - | - | - | - |
| Q16774 | Guanylate kinase | GUK1 | MSGPRPVVLS | 0 | 0 | 0 | 0 | - | 0 | 0 | 0 |
| Q15654 | Thyroid receptor-interacting protein 6 | TRIP6 | MSGPTWLPPK | - | 0 | - | - | 0 | - | - | 0 |
| Q13492 | Phosphatidylinositol-binding clathrin assembly protein | PICALM | MSGQSLTDRI | - | 0 | 0 | 0 | - | - | 0 | 0 |
| A6NIH7 | Protein unc-119 homolog B | UNC119B | MSGSNPKAAA | - | 0 | - | - | 0 | 0 | - | - |
| P63218 | Guanine nucleotide-binding protein G(I)/G(S)/G(O) subunit gamma-5 | GNG5 | MSGSSSVAAM | - | 0 | - | 0 | 0 | 0 | 0 | 0 |
| O43719 | HIV Tat-specific factor 1 | HTATSF1 | MSGTNLDGND | - | - | - | - | - | - | - | - |
| Q7Z3U7 | Protein MON2 homolog | MON2 | MSGTSSPEAV | - | 0 | - | 0 | - | - | - | - |
| Q14289 | Protein-tyrosine kinase 2-beta | PTK2B | MSGVSEPLSR | 0 | 0 | 0 | 0 | 0 | 0 | 0 | 0 |
| P12956 | X-ray repair cross-complementing protein 6 | XRCC6 | MSGWESYYKT | 0 | 0 | - | 0 | 0 | 0 | 0 | 0 |
| P13861 | cAMP-dependent protein kinase type II-alpha regulatory subunit | PRKAR2A | MSHIQIPPGL | 0 | - | 0 | 0 | 0 | 0 | 0 | 0 |
| Q08752 | Peptidyl-prolyl cis-trans isomerase D | PPID | MSHPSPQAKP | - | 0 | 0 | 0 | 0 | 0 | - | 0 |
| Q12792 | Twinfilin-1 | TWF1 | MSHQTGIQAS | 0 | - | 0 | 0 | - | 0 | - | 0 |
| O00571 | ATP-dependent RNA helicase DDX3X | DDX3X | MSHVAVENAL | 0 | - | - | - | 0 | 0 | 0 | 0 |
| Q9UJM3 | ERBB receptor feedback inhibitor 1 | ERRFI1 | MSIAGVAAQE | - | 0 | 0 | 0 | 0 | 0 | 0 | 0 |
| Q2TAY7 | WD40 repeat-containing protein SMU1 | SMU1 | MSIEIESSDV | 0 | 0 | - | 0 | 0 | - | 0 | 0 |
| P09104 | Gamma-enolase | ENO2 | MSIEKIWARE | - | - | 0 | 0 | 0 | 0 | - | - |
| Q99583 | Max-binding protein MNT | MNT | MSIETLLEAA | 0 | 0 | - | 0 | 0 | 0 | 0 | 0 |
| Q9Y3A5 | Ribosome maturation protein SBDS | SBDS | MSIFTPTNQI | - | - | - | - | - | - | - | - |
| P06733 | Alpha-enolase | ENO1 | MSILKIHARE | 0 | 0 | - | - | 0 | 0 | 0 | 0 |
| P30613 | Pyruvate kinase PKLR | PKLR | MSIQENISSL | 0 | 0 | - | - | 0 | 0 | 0 | 0 |
| Q9BZK7 | F-box-like/WD repeat-containing protein TBL1XR1 | TBL1XR1 | MSISSDEVNF | - | - | 0 | 0 | 0 | - | 0 | 0 |
| P62308 | Small nuclear ribonucleoprotein G | SNRPG | MSKAHPPELK | - | - | - | - | 0 | 0 | - | 0 |
| P11142 | Heat shock cognate 71 kDa protein | HSPA8 | MSKGPAVGID | 0 | - | - | 0 | 0 | - | 0 | 0 |
| O75874 | Isocitrate dehydrogenase [NADP] cytoplasmic | IDH1 | MSKKISGGSV | - | - | - | - | - | - | - | - |
| Q9BWT6 | Meiotic nuclear division protein 1 homolog | MND1 | MSKKKGLSAE | - | - | - | - | - | 0 | - | 0 |
| O15066 | Kinesin-like protein KIF3B | KIF3B | MSKLKSSESV | - | - | 0 | 0 | 0 | - | - | - |
| O15511 | Actin-related protein 2/3 complex subunit 5 | ARPC5 | MSKNTVSSAR | - | 0 | 0 | 0 | - | - | - | - |
| Q92882 | Osteoclast-stimulating factor 1 | OSTF1 | MSKPPPKPVK | 0 | 0 | 0 | 0 | - | 0 | 0 | 0 |
| Q15019 | Septin-2 | sept-02 | MSKQQPTQFI | - | - | 0 | 0 | - | 0 | - | - |
| Q32P51 | Heterogeneous nuclear ribonucleoprotein A1-like 2 | HNRNPA1L2 | MSKSASPKEP | - | 0 | 0 | 0 | 0 | 0 | - | - |
| P09651 | Heterogeneous nuclear ribonucleoprotein A1 | HNRNPA1 | MSKSESPKEP | +/- | +/- | - | - | - | +/- | - | +/- |
| P43243 | Matrin-3 | MATR3 | MSKSFQQSSL | - | - | - | - | - | - | - | - |
| Q15404 | Ras suppressor protein 1 | RSU1 | MSKSLKKLVE | - | - | - | - | - | - | - | - |
| Q9Y2W1 | Thyroid hormone receptor-associated protein 3 | THRAP3 | MSKTNKSKSG | - | - | 0 | 0 | - | - | - | - |
| P13489 | Ribonuclease inhibitor | RNH1 | MSLDIQSLDI | 0 | 0 | - | 0 | - | 0 | - | - |
| O60499 | Syntaxin-10 | STX10 | MSLEDPFFVV | - | - | - | - | - | - | - | - |
| Q6P4A7 | Sideroflexin-4 | SFXN4 | MSLEQEEETQ | - | 0 | - | 0 | 0 | 0 | 0 | - |
| O00165 | HCLS1-associated protein X-1 | HAX1 | MSLFDLFRGF | - | - | - | - | 0 | - | - | - |
| Q6P444 | Mitochondrial fission regulator 2 | MTFR2 | MSLILNILRE | 0 | 0 | - | - | 0 | 0 | 0 | 0 |
| Q6YP21-3 | Isoform 3 of Kynurenine--oxoglutarate transaminase 3 | CCBL2 | MSLKFTNAKR | - | - | - | - | - | - | 0 | 0 |
| Q9UKM9 | RNA-binding protein Raly | RALY | MSLKLQASNV | - | - | - | - | - | - | - | - |
| O95140 | Mitofusin-2 | MFN2 | MSLLFSRCNS | 0 | 0 | - | - | 0 | 0 | 0 | 0 |
| Q9UDY8 | Mucosa-associated lymphoid tissue lymphoma translocation protein 1 | MALT1 | MSLLGDPLQA | 0 | 0 | 0 | 0 | 0 | 0 | 0 | - |
| P62316 | Small nuclear ribonucleoprotein Sm D2 | SNRPD2 | MSLLNKPKSE | - | - | - | - | - | - | - | - |
| O14976 | Cyclin-G-associated kinase | GAK | MSLLQSALDF | - | 0 | - | - | 0 | - | 0 | 0 |
| Q6WKZ4 | Rab11 family-interacting protein 1 | RAB11FIP1 | MSLMVSAGRG | 0 | 0 | 0 | 0 | 0 | 0 | 0 | 0 |
| Q8N5C7 | DTW domain-containing protein 1 | DTWD1 | MSLNPPIFLK | - | - | - | - | 0 | - | - | 0 |
| Q9NZC9 | SWI/SNF-related matrix-associated actin-dependent regulator of chromatin subfamily A-like protein 1 | SMARCAL1 | MSLPLTEEQR | 0 | 0 | 0 | 0 | 0 | 0 | 0 | 0 |
| Q92540 | Protein SMG7 | SMG7 | MSLQSAQYLR | 0 | 0 | - | 0 | 0 | - | 0 | 0 |
| Q9Y6A5 | Transforming acidic coiled-coil-containing protein 3 | TACC3 | MSLQVLNDKN | 0 | 0 | - | - | 0 | 0 | 0 | 0 |
| P00558 | Phosphoglycerate kinase 1 | PGK1 | MSLSNKLTLD | - | - | - | - | - | - | - | - |
| P02008 | Hemoglobin subunit zeta | HBZ | MSLTKTERTI | 0 | 0 | - | - | 0 | 0 | 0 | 0 |
| Q9NZ45 | CDGSH iron-sulfur domain-containing protein 1 | CISD1 | MSLTSSSSVR | - | 0 | 0 | 0 | 0 | - | 0 | - |
| Q06547 | GA-binding protein subunit beta-1 | GABPB1 | MSLVDLGKKL | - | 0 | - | - | - | - | - | - |
| P62269 | 40S ribosomal protein S18 | RPS18 | MSLVIPEKFQ | - | - | 0 | 0 | - | 0 | - | 0 |
| Q13315 | Serine-protein kinase ATM | ATM | MSLVLNDLLI | - | 0 | 0 | 0 | 0 | - | - | 0 |
| Q96IW7 | Vesicle-trafficking protein SEC22a | SEC22A | MSMILSASVI | - | 0 | 0 | 0 | - | - | 0 | - |
| Q9H1A4 | Anaphase-promoting complex subunit 1 | ANAPC1 | MSNFYEERTT | 0 | 0 | 0 | - | 0 | 0 | 0 | 0 |
| Q99598 | Translin-associated protein X | TSNAX | MSNKEGSGGF | - | - | 0 | 0 | - | - | - | - |
| Q13618 | Cullin-3 | CUL3 | MSNLSKGTGS | - | - | 0 | 0 | - | - | - | - |
| Q13153 | Serine/threonine-protein kinase PAK 1 | PAK1 | MSNNGLDIQD | - | - | 0 | 0 | 0 | 0 | 0 | 0 |
| P21399 | Cytoplasmic aconitate hydratase | ACO1 | MSNPFAHLAE | 0 | 0 | - | - | 0 | 0 | 0 | 0 |
| Q96BX8 | MOB kinase activator 3A | MOB3A | MSNPFLKQVF | 0 | 0 | 0 | 0 | - | 0 | - | 0 |
| Q969X1 | Protein lifeguard 3 | TMBIM1 | MSNPSAPPPY | - | 0 | 0 | 0 | 0 | 0 | 0 | - |
| Q05519 | Serine/arginine-rich splicing factor 11 | SRSF11 | MSNTTVVPST | 0 | 0 | 0 | - | 0 | 0 | 0 | 0 |
| Q96HC4 | PDZ and LIM domain protein 5 | PDLIM5 | MSNYSVSLVG | - | 0 | 0 | 0 | 0 | - | 0 | 0 |
| A8CG34 | Nuclear envelope pore membrane protein POM 121C | POM121C | MSPAAAAAGA | - | 0 | 0 | 0 | - | - | 0 | 0 |
| Q5NUL3 | Free fatty acid receptor 4 | FFAR4 | MSPECARAAG | 0 | 0 | - | 0 | 0 | 0 | 0 | 0 |
| Q9NXR7 | BRCA1-A complex subunit BRE | BRE | MSPEVALNRI | + | + | + | + | + | + | + | + |
| Q96FV9 | THO complex subunit 1 | THOC1 | MSPTPPLFSL | 0 | 0 | - | - | - | - | 0 | 0 |
| P07108 | Acyl-CoA-binding protein | DBI | MSQAEFEKAA | - | - | - | - | - | - | - | - |
| Q16851-2 | Isoform 2 of UTP--glucose-1-phosphate uridylyltransferase | UGP2 | MSQDGASQFQ | - | - | - | - | - | - | - | - |
| Q9NUL5 | UPF0515 protein C19orf66 | C19orf66 | MSQEGVELEK | 0 | 0 | 0 | 0 | - | - | 0 | 0 |
| P68402 | Platelet-activating factor acetylhydrolase IB subunit beta | PAFAH1B2 | MSQGDSNPAA | - | - | 0 | 0 | - | 0 | - | - |
| Q9BW04-2 | Isoform 2 of Specifically androgen-regulated gene protein | SARG | MSQKAKETVS | - | 0 | 0 | 0 | 0 | 0 | 0 | 0 |
| O43768 | Alpha-endosulfine | ENSA | MSQKQEEENP | 0 | 0 | - | - | 0 | 0 | - | 0 |
| P20810-5 | Isoform 5 of Calpastatin | CAST | MSQPGQKPAA | - | - | 0 | 0 | - | - | - | - |
| Q9BZ29 | Dedicator of cytokinesis protein 9 | DOCK9 | MSQPPLLPAS | - | 0 | 0 | 0 | 0 | 0 | 0 | 0 |
| P52564 | Dual specificity mitogen-activated protein kinase kinase 6 | MAP2K6 | MSQSKGKKRN | 0 | 0 | 0 | 0 | - | - | 0 | 0 |
| B4DJY2 | Transmembrane protein 233 | TMEM233 | MSQYAPSPDF | 0 | 0 | 0 | 0 | 0 | 0 | - | - |
| O75116 | Rho-associated protein kinase 2 | ROCK2 | MSRPPPTGKM | - | 0 | 0 | 0 | 0 | 0 | 0 | 0 |
| P04264 | Keratin, type II cytoskeletal 1 | KRT1 | MSRQFSSRSG | 0 | 0 | 0 | 0 | - | 0 | 0 | 0 |
| P13647 | Keratin, type II cytoskeletal 5 | KRT5 | MSRQSSVSFR | 0 | 0 | 0 | 0 | - | 0 | 0 | 0 |
| Q16537 | Serine/threonine-protein phosphatase 2A 56 kDa regulatory subunit epsilon isoform | PPP2R5E | MSSAPTTPPS | 0 | 0 | - | - | - | - | - | - |
| Q96RR4 | Calcium/calmodulin-dependent protein kinase kinase 2 | CAMKK2 | MSSCVSSQPS | - | 0 | 0 | 0 | 0 | - | 0 | 0 |
| Q8TBZ6 | tRNA methyltransferase 10 homolog A | TRMT10A | MSSEMLPAFI | - | 0 | 0 | 0 | 0 | 0 | 0 | 0 |
| Q9Y2V2 | Calcium-regulated heat stable protein 1 | CARHSP1 | MSSEPPPPPQ | - | 0 | 0 | 0 | 0 | 0 | - | - |
| Q5JPI9 | Methyltransferase-like protein 10 | METTL10 | MSSGADGGGG | - | - | 0 | - | - | - | - | - |
| P24723 | Protein kinase C eta type | PRKCH | MSSGTMKFNG | 0 | 0 | 0 | 0 | - | 0 | 0 | 0 |
| P25788 | Proteasome subunit alpha type-3 | PSMA3 | MSSIGTGYDL | 0 | - | - | - | - | - | - | - |
| P25705-2 | Isoform 2 of ATP synthase subunit alpha, mitochondrial | ATP5A1 | MSSILEERIL | +/- | 0 | - | 0 | 0 | +/- | 0 | 0 |
| P84022 | Mothers against decapentaplegic homolog 3 | SMAD3 | MSSILPFTPP | - | - | - | - | - | - | - | - |
| Q15796 | Mothers against decapentaplegic homolog 2 | SMAD2 | MSSILPFTPP | - | - | - | 0 | - | - | 0 | 0 |
| Q6PGP7 | Tetratricopeptide repeat protein 37 | TTC37 | MSSKEVKTAL | - | - | 0 | 0 | - | - | 0 | - |
| O14950 | Myosin regulatory light chain 12B | MYL12B | MSSKKAKTKT | - | - | 0 | 0 | - | - | - | - |
| Q9H501 | ESF1 homolog | ESF1 | MSSKQEIMSD | - | - | 0 | 0 | - | - | 0 | 0 |
| Q9UBI6 | Guanine nucleotide-binding protein G(I)/G(S)/G(O) subunit gamma-12 | GNG12 | MSSKTASTNN | - | - | - | - | - | - | - | - |
| Q9Y291 | 28S ribosomal protein S33, mitochondrial | MRPS33 | MSSLSEYAFR | 0 | 0 | 0 | 0 | - | - | 0 | 0 |
| Q96BW5 | Phosphotriesterase-related protein | PTER | MSSLSGKVQT | - | 0 | - | - | 0 | 0 | 0 | 0 |
| P62633 | Cellular nucleic acid-binding protein | CNBP | MSSNECFKCG | - | - | - | 0 | - | - | - | 0 |
| P33991 | DNA replication licensing factor MCM4 | MCM4 | MSSPASTPSR | - | 0 | 0 | 0 | 0 | - | 0 | - |
| Q8N461 | F-box/LRR-repeat protein 16 | FBXL16 | MSSPGIDGDP | - | 0 | 0 | 0 | 0 | 0 | 0 | 0 |
| P51397 | Death-associated protein 1 | DAP | MSSPPEGKLE | - | - | - | - | 0 | - | - | 0 |
| P62256 | Ubiquitin-conjugating enzyme E2 H | UBE2H | MSSPSPGKRR | 0 | 0 | 0 | 0 | 0 | 0 | 0 | 0 |
| Q96MD7 | Uncharacterized protein C9orf85 | C9orf85 | MSSQKGNVAR | - | 0 | 0 | 0 | 0 | - | 0 | 0 |
| Q13867 | Bleomycin hydrolase | BLMH | MSSSGLNSEK | 0 | 0 | - | 0 | 0 | 0 | 0 | 0 |
| Q9UQ13 | Leucine-rich repeat protein SHOC-2 | SHOC2 | MSSSLGKEKD | 0 | 0 | - | - | 0 | 0 | - | - |
| A8MXV4 | Nucleoside diphosphate-linked moiety X motif 19, mitochondrial | NUDT19 | MSSSLRPGPS | - | 0 | 0 | 0 | 0 | 0 | 0 | 0 |
| P22314 | Ubiquitin-like modifier-activating enzyme 1 | UBA1 | MSSSPLSKKR | - | - | - | - | - | - | - | - |
| Q00839 | Heterogeneous nuclear ribonucleoprotein U | HNRNPU | MSSSPVNVKK | - | 0 | - | - | 0 | - | - | - |
| P22307 | Non-specific lipid-transfer protein | SCP2 | MSSSPWEPAT | 0 | - | 0 | 0 | 0 | 0 | 0 | 0 |
| Q8IWS0 | PHD finger protein 6 | PHF6 | MSSSVEQKKG | - | - | - | - | - | - | - | - |
| Q9Y295 | Developmentally-regulated GTP-binding protein 1 | DRG1 | MSSTLAKIAE | - | - | - | - | - | - | - | - |
| Q99439 | Calponin-2 | CNN2 | MSSTQFNKGP | - | - | - | - | - | - | - | - |
| O00401 | Neural Wiskott-Aldrich syndrome protein | WASL | MSSVQQQPPP | - | 0 | 0 | 0 | - | - | - | - |
| Q9BRJ7 | Protein syndesmos | NUDT16L1 | MSTAAVPELK | - | 0 | - | 0 | - | 0 | 0 | 0 |
| P14621 | Acylphosphatase-2 | ACYP2 | MSTAQSLKSV | 0 | 0 | - | 0 | 0 | 0 | 0 | 0 |
| Q15059 | Bromodomain-containing protein 3 | BRD3 | MSTATTVAPA | 0 | 0 | - | 0 | 0 | 0 | 0 | 0 |
| Q9Y241 | HIG1 domain family member 1A, mitochondrial | HIGD1A | MSTDTGVSLP | - | - | - | - | - | - | - | - |
| P20340 | Ras-related protein Rab-6A | RAB6A | MSTGGDFGNP | - | - | - | - | - | - | - | - |
| Q15836 | Vesicle-associated membrane protein 3 | VAMP3 | MSTGPTAATG | - | - | 0 | 0 | - | - | 0 | - |
| P49189 | 4-trimethylaminobutyraldehyde dehydrogenase | ALDH9A1 | MSTGTFVVSQ | +/- | +/- | +/- | - | +/- | +/- | +/- | - |
| O60739 | Eukaryotic translation initiation factor 1b | EIF1B | MSTIQNLQSF | 0 | 0 | - | - | 0 | 0 | - | 0 |
| Q9H000 | Probable E3 ubiquitin-protein ligase makorin-2 | MKRN2 | MSTKQITCRY | 0 | 0 | 0 | 0 | 0 | - | 0 | 0 |
| P60006 | Anaphase-promoting complex subunit 15 | ANAPC15 | MSTLFPSLFP | - | - | - | - | 0 | - | - | 0 |
| P49419-2 | Isoform 2 of Alpha-aminoadipic semialdehyde dehydrogenase | ALDH7A1 | MSTLLINQPQ | +/- | 0 | 0 | 0 | 0 | +/- | 0 | 0 |
| P26640 | Valine--tRNA ligase | VARS | MSTLYVSPHP | - | - | 0 | 0 | - | 0 | 0 | 0 |
| P52292 | Importin subunit alpha-1 | KPNA2 | MSTNENANTP | - | - | 0 | 0 | - | - | 0 | - |
| Q9Y284 | Protein Asterix | WDR83OS | MSTNNMSDPR | - | 0 | 0 | 0 | 0 | - | 0 | 0 |
| P51532 | Transcription activator BRG1 | SMARCA4 | MSTPDPPLGG | - | - | - | - | 0 | - | 0 | 0 |
| P16885 | 1-phosphatidylinositol 4,5-bisphosphate phosphodiesterase gamma-2 | PLCG2 | MSTTVNVDSL | - | 0 | 0 | 0 | 0 | 0 | 0 | 0 |
| Q8TBF2 | Prostamide/prostaglandin F synthase | FAM213B | MSTVDLARVG | 0 | 0 | 0 | 0 | 0 | - | 0 | 0 |
| Q15532 | Protein SSXT | SS18 | MSVAFAAPRQ | 0 | 0 | 0 | 0 | 0 | - | - | 0 |
| O75177 | Calcium-responsive transactivator | SS18L1 | MSVAFASARP | 0 | 0 | - | - | 0 | 0 | 0 | 0 |
| Q9H773 | dCTP pyrophosphatase 1 | DCTPP1 | MSVAGGEIRG | - | 0 | 0 | 0 | - | - | - | 0 |
| Q8TAE6 | Protein phosphatase 1 regulatory subunit 14C | PPP1R14C | MSVATGSSET | - | 0 | 0 | 0 | 0 | 0 | 0 | 0 |
| Q92522 | Histone H1x | H1FX | MSVELEEALP | - | 0 | 0 | 0 | 0 | 0 | 0 | 0 |
| Q9H444 | Charged multivesicular body protein 4b | CHMP4B | MSVFGKLFGA | 0 | 0 | - | - | 0 | - | - | - |
| Q96C36 | Pyrroline-5-carboxylate reductase 2 | PYCR2 | MSVGFIGAGQ | - | 0 | - | - | 0 | - | 0 | 0 |
| Q27J81 | Inverted formin-2 | INF2 | MSVKEGAQRK | - | 0 | 0 | 0 | - | - | - | - |
| Q8TD19 | Serine/threonine-protein kinase Nek9 | NEK9 | MSVLGEYERH | - | 0 | - | - | - | - | 0 | 0 |
| Q7L2H7 | Eukaryotic translation initiation factor 3 subunit M | EIF3M | MSVPAFIDIS | 0 | 0 | - | - | - | - | - | - |
| P56945-8 | Isoform 8 of Breast cancer anti-estrogen resistance protein 1 | BCAR1 | MSVPNVLAKA | 0 | 0 | 0 | 0 | 0 | - | - | 0 |
| P55081 | Microfibrillar-associated protein 1 | MFAP1 | MSVPSALMKQ | 0 | 0 | - | 0 | 0 | 0 | 0 | 0 |
| Q6P597 | Kinesin light chain 3 | KLC3 | MSVQVAAPGS | - | - | 0 | 0 | 0 | 0 | 0 | 0 |
| Q15631 | Translin | TSN | MSVSEIFVEL | 0 | 0 | 0 | 0 | 0 | 0 | 0 | 0 |
| Q92598 | Heat shock protein 105 kDa | HSPH1 | MSVVGLDVGS | 0 | 0 | 0 | 0 | 0 | - | - | 0 |
| P49454 | Centromere protein F | CENPF | MSWALEEWKE | 0 | 0 | - | 0 | 0 | 0 | 0 | 0 |
| Q8TBA6 | Golgin subfamily A member 5 | GOLGA5 | MSWFVDLAGK | 0 | 0 | - | 0 | 0 | 0 | 0 | 0 |
| Q9NVI7 | ATPase family AAA domain-containing protein 3A | ATAD3A | MSWLFGINKG | 0 | - | - | - | 0 | 0 | 0 | - |
| Q5T9A4 | ATPase family AAA domain-containing protein 3B | ATAD3B | MSWLFGVNKG | 0 | 0 | - | - | 0 | 0 | 0 | 0 |
| Q8NEZ2 | Vacuolar protein sorting-associated protein 37A | VPS37A | MSWLFPLTKS | 0 | 0 | 0 | 0 | 0 | 0 | 0 | - |
| Q86Y82 | Syntaxin-12 | STX12 | MSYGPLDMYR | 0 | 0 | 0 | 0 | - | - | 0 | 0 |
| Q01130 | Serine/arginine-rich splicing factor 2 | SRSF2 | MSYGRPPPDV | - | - | 0 | 0 | - | 0 | 0 | 0 |
| O14907 | Tax1-binding protein 3 | TAX1BP3 | MSYIPGQPVT | - | - | 0 | 0 | - | - | - | - |
| Q9Y262 | Eukaryotic translation initiation factor 3 subunit L | EIF3L | MSYPADDYES | 0 | 0 | 0 | 0 | 0 | 0 | 0 | 0 |
| Q14195 | Dihydropyrimidinase-related protein 3 | DPYSL3 | MSYQGKKNIP | - | - | - | 0 | - | 0 | - | - |
| O15400 | Syntaxin-7 | STX7 | MSYTPGVGGD | - | - | - | - | - | - | - | - |
| Q9Y5J5 | Pleckstrin homology-like domain family A member 3 | PHLDA3 | MTAAATATVL | 0 | 0 | 0 | 0 | 0 | 0 | 0 | - |
| O00212 | Rho-related GTP-binding protein RhoD | RHOD | MTAAQAAGEE | - | 0 | 0 | 0 | 0 | 0 | 0 | 0 |
| Q02790 | Peptidyl-prolyl cis-trans isomerase FKBP4 | FKBP4 | MTAEEMKATE | 0 | 0 | +/- | 0 | 0 | 0 | +/- | +/- |
| P60484 | Phosphatidylinositol 3,4,5-trisphosphate 3-phosphatase and dual-specificity protein phosphatase PTEN | PTEN | MTAIIKEIVS | - | - | - | - | 0 | - | - | 0 |
| Q9BYB4 | Guanine nucleotide-binding protein subunit beta-like protein 1 | GNB1L | MTAPCPPPPP | - | 0 | - | - | 0 | - | 0 | - |
| Q5J8M3 | ER membrane protein complex subunit 4 | EMC4 | MTAQGGLVAN | - | 0 | 0 | 0 | - | - | - | - |
| Q9H3U1-2 | Isoform 2 of Protein unc-45 homolog A | UNC45A | MTASSVEQLR | - | - | - | - | - | - | - | - |
| P35221 | Catenin alpha-1 | CTNNA1 | MTAVHAGNIN | - | - | 0 | 0 | 0 | 0 | 0 | 0 |
| P05141 | ADP/ATP translocase 2 | SLC25A5 | MTDAAVSFAK | - | 0 | +/- | - | - | +/- | 0 | 0 |
| Q9H1Y0 | Autophagy protein 5 | ATG5 | MTDDKDVLRD | 0 | 0 | 0 | 0 | - | 0 | + | 0 |
| Q14155-1 | Isoform 1 of Rho guanine nucleotide exchange factor 7 | ARHGEF7 | MTDNSNNQLV | - | 0 | 0 | 0 | 0 | - | + | 0 |
| P46976 | Glycogenin-1 | GYG1 | MTDQAFVTLT | 0 | 0 | - | 0 | 0 | 0 | 0 | - |
| P63010 | AP-2 complex subunit beta | AP2B1 | MTDSKYFTTN | - | 0 | - | - | 0 | 0 | 0 | 0 |
| Q9H446 | RWD domain-containing protein 1 | RWDD1 | MTDYGEEQRN | 0 | 0 | 0 | 0 | 0 | + | 0 | 0 |
| P48449 | Lanosterol synthase | LSS | MTEGTCLRRR | 0 | 0 | 0 | 0 | 0 | +/- | 0 | 0 |
| P62253 | Ubiquitin-conjugating enzyme E2 G1 | UBE2G1 | MTELQSALLL | - | - | - | - | - | 0 | +/- | 0 |
| P07305 | Histone H1.0 | H1F0 | MTENSTSAPA | +/- | - | 0 | 0 | - | +/- | 0 | + |
| P12236 | ADP/ATP translocase 3 | SLC25A6 | MTEQAISFAK | 0 | 0 | - | - | 0 | - | 0 | 0 |
| P63244 | Guanine nucleotide-binding protein subunit beta-2-like 1 | GNB2L1 | MTEQMTLRGT | +/- | +/- | +/- | +/- | - | +/- | + | +/- |
| Q8WU79 | Stromal membrane-associated protein 2 | SMAP2 | MTGKSVKDVD | 0 | 0 | 0 | 0 | 0 | 0 | 0 | 0 |
| Q7Z4I7 | LIM and senescent cell antigen-like-containing domain protein 2 | LIMS2 | MTGSNMSDAL | 0 | 0 | 0 | 0 | - | 0 | 0 | 0 |
| O95139 | NADH dehydrogenase [ubiquinone] 1 beta subcomplex subunit 6 | NDUFB6 | MTGYTPDEKL | - | - | - | - | - | - | 0 | - |
| Q96AG4 | Leucine-rich repeat-containing protein 59 | LRRC59 | MTKAGSKGGN | +/- | +/- | +/- | +/- | - | +/- | +/- | +/- |
| P46977 | Dolichyl-diphosphooligosaccharide--protein glycosyltransferase subunit STT3A | STT3A | MTKFGFLRLS | - | +/- | +/- | +/- | - | +/- | 0 | 0 |
| P28838-2 | Isoform 2 of Cytosol aminopeptidase | LAP3 | MTKGLVLGIY | 0 | 0 | - | 0 | 0 | 0 | 0 | 0 |
| P61927 | 60S ribosomal protein L37 | RPL37 | MTKGTSSFGK | - | +/- | +/- | +/- | - | +/- | +/- | - |
| Q9NX24 | H/ACA ribonucleoprotein complex subunit 2 | NHP2 | MTKIKADPDG | 0 | 0 | - | 0 | - | - | - | 0 |
| Q9P0B6 | Coiled-coil domain-containing protein 167 | CCDC167 | MTKKKRENLG | + | 0 | 0 | + | 0 | + | 0 | 0 |
| Q9UI12 | V-type proton ATPase subunit H | ATP6V1H | MTKMDIRGAV | 0 | 0 | 0 | 0 | 0 | 0 | 0 | 0 |
| P43003 | Excitatory amino acid transporter 1 | SLC1A3 | MTKSNGEEPK | - | 0 | 0 | 0 | 0 | 0 | - | +/- |
| Q9BV20 | Methylthioribose-1-phosphate isomerase | MRI1 | MTLEAIRYSR | 0 | 0 | +/- | 0 | 0 | 0 | 0 | 0 |
| P29992 | Guanine nucleotide-binding protein subunit alpha-11 | GNA11 | MTLESMMACC | 0 | 0 | 0 | 0 | - | 0 | 0 | 0 |
| P55042 | GTP-binding protein RAD | RRAD | MTLNGGGSGA | 0 | 0 | 0 | 0 | 0 | 0 | 0 | 0 |
| P55040 | GTP-binding protein GEM | GEM | MTLNNVTMRQ | 0 | 0 | 0 | 0 | 0 | 0 | 0 | 0 |
| P31946 | 14-3-3 protein beta/alpha | YWHAB | MTMDKSELVQ | +/- | +/- | +/- | +/- | +/- | +/- | +/- | +/- |
| Q15007 | Pre-mRNA-splicing regulator WTAP | WTAP | MTNEEPLPKK | - | 0 | 0 | 0 | - | - | 0 | 0 |
| P46778 | 60S ribosomal protein L21 | RPL21 | MTNTKGKRRG | +/- | +/- | 0 | + | +/- | +/- | + | +/- |
| Q9H981 | Actin-related protein 8 | ACTR8 | MTQAEKGDTE | 0 | 0 | 0 | 0 | 0 | 0 | 0 | + |
| P08621 | U1 small nuclear ribonucleoprotein 70 kDa | SNRNP70 | MTQFLPPNLL | - | 0 | - | - | 0 | - | 0 | 0 |
| Q96MC6 | Hippocampus abundant transcript 1 protein | HIAT1 | MTQGKKKKRA | 0 | 0 | + | + | - | + | 0 | 0 |
| Q5JTD0 | Tight junction-associated protein 1 | TJAP1 | MTSAAPAKKP | - | 0 | 0 | 0 | - | - | 0 | 0 |
| O95777 | U6 snRNA-associated Sm-like protein LSm8 | LSM8 | MTSALENYIN | - | - | - | - | - | - | - | - |
| Q0VGL1 | Ragulator complex protein LAMTOR4 | LAMTOR4 | MTSALTQGLE | - | - | - | - | - | - | - | - |
| Q9H6T3 | RNA polymerase II-associated protein 3 | RPAP3 | MTSANKAIEL | 0 | 0 | - | - | 0 | 0 | 0 | 0 |
| P11277 | Spectrin beta chain, erythrocytic | SPTB | MTSATEFENV | 0 | 0 | - | 0 | 0 | 0 | 0 | 0 |
| Q92615 | La-related protein 4B | LARP4B | MTSDQDAKVV | + | 0 | - | 0 | 0 | +/- | - | 0 |
| Q03154 | Aminoacylase-1 | ACY1 | MTSKGPEEEH | 0 | 0 | 0 | 0 | 0 | 0 | 0 | 0 |
| Q3LXA3 | Bifunctional ATP-dependent dihydroxyacetone kinase/FAD-AMP lyase (cyclizing) | DAK | MTSKKLVNSV | - | - | - | - | 0 | 0 | 0 | 0 |
| Q8NB91 | Fanconi anemia group B protein | FANCB | MTSKQAMSSN | - | 0 | 0 | 0 | 0 | - | - | 0 |
| Q9H583 | HEAT repeat-containing protein 1 | HEATR1 | MTSLAQQLQR | - | 0 | - | - | - | - | 0 | 0 |
| Q99717 | Mothers against decapentaplegic homolog 5 | SMAD5 | MTSMASLFSF | - | 0 | 0 | 0 | 0 | 0 | 0 | 0 |
| Q9NPJ3 | Acyl-coenzyme A thioesterase 13 | ACOT13 | MTSMTQSLRE | +/- | + | +/- | +/- | - | +/- | 0 | 0 |
| Q32MZ4 | Leucine-rich repeat flightless-interacting protein 1 | LRRFIP1 | MTSPAAAQSR | - | 0 | 0 | 0 | - | +/- | 0 | + |
| Q9Y5S1 | Transient receptor potential cation channel subfamily V member 2 | TRPV2 | MTSPSSSPVF | 0 | 0 | - | - | 0 | 0 | 0 | 0 |
| P02794 | Ferritin heavy chain | FTH1 | MTTASTSQVR | - | 0 | 0 | 0 | - | - | 0 | - |
| Q13451 | Peptidyl-prolyl cis-trans isomerase FKBP5 | FKBP5 | MTTDEGAKNN | +/- | 0 | +/- | +/- | - | 0 | 0 | 0 |
| O60476 | Mannosyl-oligosaccharide 1,2-alpha-mannosidase IB | MAN1A2 | MTTPALLPLS | - | 0 | 0 | 0 | - | - | 0 | 0 |
| P52294 | Importin subunit alpha-5 | KPNA1 | MTTPGKENFR | 0 | 0 | - | - | - | 0 | 0 | 0 |
| Q9UM13 | Anaphase-promoting complex subunit 10 | ANAPC10 | MTTPNKTPPG | - | - | - | - | - | - | - | - |
| O00151 | PDZ and LIM domain protein 1 | PDLIM1 | MTTQQIDLQG | - | - | - | - | 0 | - | - | - |
| Q16186 | Proteasomal ubiquitin receptor ADRM1 | ADRM1 | MTTSGALFPS | - | - | - | - | - | - | - | - |
| Q9UN37 | Vacuolar protein sorting-associated protein 4A | VPS4A | MTTSTLQKAI | - | 0 | - | - | 0 | 0 | 0 | 0 |
| Q9UK76 | Hematological and neurological expressed 1 protein | HN1 | MTTTTTFKGV | - | - | - | - | - | - | - | - |
| Q01082 | Spectrin beta chain, non-erythrocytic 1 | SPTBN1 | MTTTVATDYD | 0 | 0 | 0 | 0 | - | 0 | - | - |
| Q15436 | Protein transport protein Sec23A | SEC23A | MTTYLEFIQQ | - | - | - | - | - | - | - | - |
| Q53H12 | Acylglycerol kinase, mitochondrial | AGK | MTVFFKTLRN | - | - | - | - | - | - | - | - |
| P14678 |  | SNRPB | MTVGKSSKML | - | - | 0 | 0 | - | 0 | - | 0 |
| Q9H3U1 | Protein unc-45 homolog A | UNC45A | MTVSGPGTPE | - | - | 0 | 0 | - | - | 0 | - |
| P62888 | 60S ribosomal protein L30 | RPL30 | MVAAKKTKKS | - | +/- | - | +/- | - | +/- | +/- | +/- |
| Q15758 | Neutral amino acid transporter B(0) | SLC1A5 | MVADPPRDSK | + | + | 0 | + | 0 | + | + | + |
| Q15149 | Plectin | PLEC | MVAGMLMPRD | 0 | 0 | 0 | 0 | - | 0 | 0 | 0 |
| Q8N6R1 | Stress-associated endoplasmic reticulum protein 2 | SERP2 | MVAKQRIRMA | 0 | 0 | 0 | 0 | 0 | + | 0 | + |
| A2VDF0 | Fucose mutarotase | FUOM | MVALKGVPAL | 0 | 0 | - | - | 0 | 0 | 0 | 0 |
| P35244 | Replication protein A 14 kDa subunit | RPA3 | MVDMMDLPRS | - | - | - | - | - | - | - | 0 |
| P61981 | 14-3-3 protein gamma | YWHAG | MVDREQLVQK | 0 | 0 | 0 | 0 | 0 | 0 | + | + |
| O75190 | DnaJ homolog subfamily B member 6 | DNAJB6 | MVDYYEVLGV | - | - | - | - | - | - | - | - |
| Q9P1Y5 | Calmodulin-regulated spectrin-associated protein 3 | CAMSAP3 | MVEAAPPGPG | - | 0 | 0 | 0 | 0 | 0 | 0 | 0 |
| P38159 | RNA-binding motif protein, X chromosome | RBMX | MVEADRPGKL | 0 | 0 | - | - | 0 | - | 0 | 0 |
| Q8IWA0 | WD repeat-containing protein 75 | WDR75 | MVEEENIRVV | 0 | 0 | 0 | 0 | 0 | 0 | 0 | 0 |
| Q9UBE0 | SUMO-activating enzyme subunit 1 | SAE1 | MVEKEEAGGG | +/- | +/- | +/- | +/- | - | +/- | +/- | +/- |
| Q9UKL0 | REST corepressor 1 | RCOR1 | MVEKGPEVSG | - | - | - | - | - | - | 0 | 0 |
| O43257 | Zinc finger HIT domain-containing protein 1 | ZNHIT1 | MVEKKTSVRS | 0 | 0 | 0 | 0 | - | 0 | 0 | 0 |
| A4D1U4 | Protein LCHN | LCHN | MVEQGDAAPL | - | 0 | 0 | 0 | 0 | 0 | 0 | 0 |
| Q96RL7 | Vacuolar protein sorting-associated protein 13A | VPS13A | MVFESVVVDV | 0 | 0 | 0 | 0 | 0 | 0 | - | 0 |
| P35610 | Sterol O-acyltransferase 1 | SOAT1 | MVGEEKMSLR | 0 | 0 | +/- | 0 | +/- | +/- | 0 | 0 |
| Q96HQ2 | CDKN2AIP N-terminal-like protein | CDKN2AIPNL | MVGGEAAAAV | +/- | 0 | 0 | 0 | + | +/- | + | 0 |
| P27694 | Replication protein A 70 kDa DNA-binding subunit | RPA1 | MVGQLSEGAI | 0 | 0 | - | - | 0 | 0 | 0 | 0 |
| P00374 | Dihydrofolate reductase | DHFR | MVGSLNCIVA | 0 | 0 | - | 0 | 0 | 0 | 0 | 0 |
| O43396 | Thioredoxin-like protein 1 | TXNL1 | MVGVKPVGSD | +/- | +/- | +/- | +/- | - | +/- | +/- | +/- |
| Q9HC21 | Mitochondrial thiamine pyrophosphate carrier | SLC25A19 | MVGYDPKPDG | 0 | 0 | - | 0 | 0 | - | 0 | 0 |
| P02100 | Hemoglobin subunit epsilon | HBE1 | MVHFTAEEKA | 0 | 0 | - | - | 0 | 0 | 0 | 0 |
| Q9NUQ7 | Ufm1-specific protease 2 | UFSP2 | MVISESMDIL | - | 0 | - | 0 | - | 0 | 0 | 0 |
| O14802 | DNA-directed RNA polymerase III subunit RPC1 | POLR3A | MVKEQFRETD | 0 | 0 | 0 | + | 0 | 0 | 0 | 0 |
| P31689 | DnaJ homolog subfamily A member 1 | DNAJA1 | MVKETTYYDV | - | 0 | - | - | - | 0 | - | - |
| P36871 | Phosphoglucomutase-1 | PGM1 | MVKIVTVKTQ | - | +/- | 0 | 0 | - | +/- | +/- | +/- |
| Q9BW83 | Intraflagellar transport protein 27 homolog | IFT27 | MVKLAAKCIL | 0 | 0 | - | 0 | 0 | 0 | 0 | 0 |
| P19338 | Nucleolin | NCL | MVKLAKAGKN | - | 0 | 0 | 0 | 0 | 0 | 0 | 0 |
| Q9BQ04 | RNA-binding protein 4B | RBM4B | MVKLFIGNLP | - | - | +/- | +/- | - | - | +/- | - |
| Q9P0U1 | Mitochondrial import receptor subunit TOM7 homolog | TOMM7 | MVKLSKEAKQ | 0 | 0 | - | + | - | +/- | 0 | 0 |
| P09661 |  | SNRPA1 | MVKLTAELIE | - | 0 | - | - | - | - | - | 0 |
| Q13823 | Nucleolar GTP-binding protein 2 | GNL2 | MVKPKYKGRS | - | 0 | - | + | - | + | 0 | 0 |
| Q9Y255 | PRELI domain-containing protein 1, mitochondrial | PRELID1 | MVKYFLGQSV | 0 | 0 | 0 | - | 0 | 0 | - | 0 |
| P61011 | Signal recognition particle 54 kDa protein | SRP54 | MVLADLGRKI | - | +/- | - | +/- | - | - | - | - |
| O43776 | Asparagine--tRNA ligase, cytoplasmic | NARS | MVLAELYVSD | - | - | - | - | - | - | - | - |
| Q86VV8 | Rotatin | RTTN | MVLAGLIRKL | - | 0 | - | 0 | 0 | 0 | - | - |
| O95671 | N-acetylserotonin O-methyltransferase-like protein | ASMTL | MVLCPVIGKL | - | - | 0 | 0 | 0 | 0 | - | 0 |
| P49591 | Serine--tRNA ligase, cytoplasmic | SARS | MVLDLDLFRV | - | +/- | +/- | +/- | - | - | +/- | - |
| P55036 | 26S proteasome non-ATPase regulatory subunit 4 | PSMD4 | MVLESTMVCV | 0 | 0 | - | 0 | - | 0 | - | - |
| Q8N5K1 | CDGSH iron-sulfur domain-containing protein 2 | CISD2 | MVLESVARIV | 0 | 0 | - | +/- | - | - | 0 | 0 |
| Q709C8 | Vacuolar protein sorting-associated protein 13C | VPS13C | MVLESVVADL | - | 0 | +/- | +/- | 0 | - | +/- | 0 |
| P48739 | Phosphatidylinositol transfer protein beta isoform | PITPNB | MVLIKEFRVV | +/- | +/- | +/- | +/- | - | +/- | +/- | +/- |
| P48444 | Coatomer subunit delta | ARCN1 | MVLLAAAVCT | - | 0 | - | - | 0 | - | - | - |
| P37108 | Signal recognition particle 14 kDa protein | SRP14 | MVLLESEQFL | - | 0 | +/- | +/- | - | - | +/- | - |
| Q00169 | Phosphatidylinositol transfer protein alpha isoform | PITPNA | MVLLKEYRVI | - | +/- | - | +/- | - | +/- | +/- | - |
| O75396 | Vesicle-trafficking protein SEC22b | SEC22B | MVLLTMIARV | - | +/- | - | +/- | - | +/- | +/- | +/- |
| Q7Z2Z2 | Elongation factor Tu GTP-binding domain-containing protein 1 | EFTUD1 | MVLNSLDKMI | - | 0 | - | 0 | 0 | - | 0 | - |
| Q8TB03 | Uncharacterized protein CXorf38 | CXorf38 | MVLSELAARL | - | 0 | - | 0 | - | - | - | 0 |
| P69905 | Hemoglobin subunit alpha | HBA1 | MVLSPADKTN | 0 | 0 | - | - | 0 | 0 | 0 | 0 |
| Q8TF71 | Monocarboxylate transporter 10 | SLC16A10 | MVLSQEEPDS | - | 0 | 0 | 0 | 0 | 0 | 0 | 0 |
| Q9Y4C1 | Lysine-specific demethylase 3A | KDM3A | MVLTLGESWP | - | 0 | - | 0 | 0 | 0 | 0 | 0 |
| Q9Y3A3 | MOB-like protein phocein | MOB4 | MVMAEGTAVL | 0 | - | - | 0 | - | 0 | 0 | 0 |
| Q9UN86 | Ras GTPase-activating protein-binding protein 2 | G3BP2 | MVMEKPSPLL | - | +/- | + | + | - | +/- | +/- | 0 |
| Q96G97 | Seipin | BSCL2 | MVNDPPVPAL | 0 | 0 | 0 | 0 | 0 | 0 | 0 | 0 |
| P13639 | Elongation factor 2 | EEF2 | MVNFTVDQIR | +/- | +/- | +/- | +/- | - | +/- | +/- | +/- |
| Q96I45 | Transmembrane protein 141 | TMEM141 | MVNLGLSRVD | 0 | 0 | - | 0 | 0 | 0 | 0 | 0 |
| O75438 | NADH dehydrogenase [ubiquinone] 1 beta subcomplex subunit 1 | NDUFB1 | MVNLLQIVRD | 0 | 0 | +/- | +/- | 0 | 0 | 0 | 0 |
| P62937 | Peptidyl-prolyl cis-trans isomerase A | PPIA | MVNPTVFFDI | 0 | 0 | +/- | +/- | 0 | +/- | +/- | + |
| Q969Q0 | 60S ribosomal protein L36a-like | RPL36AL | MVNVPKTRRT | - | +/- | - | +/- | - | +/- | +/- | +/- |
| Q71SY5 | Mediator of RNA polymerase II transcription subunit 25 | MED25 | MVPGSEGPAR | + | 0 | 0 | + | 0 | 0 | 0 | 0 |
| P56385 | ATP synthase subunit e, mitochondrial | ATP5I | MVPPVQVSPL | + | + | + | + | +/- | + | + | 0 |
| P41440 | Folate transporter 1 | SLC19A1 | MVPSSPAVEK | 0 | 0 | + | 0 | 0 | 0 | 0 | 0 |
| Q9BV57 | 1,2-dihydroxy-3-keto-5-methylthiopentene dioxygenase | ADI1 | MVQAWYMDDA | 0 | 0 | - | 0 | 0 | 0 | 0 | 0 |
| P0CG13 | Chromosome transmission fidelity protein 8 homolog | CHTF8 | MVQIVISSAR | - | - | 0 | 0 | 0 | 0 | 0 | 0 |
| O60502 | Protein O-GlcNAcase | MGEA5 | MVQKESQATL | - | - | - | - | - | - | - | - |
| Q8WXG6 | MAP kinase-activating death domain protein | MADD | MVQKKKFCPR | - | 0 | - | 0 | 0 | - | 0 | 0 |
| Q92503 | SEC14-like protein 1 | SEC14L1 | MVQKYQSPVR | 0 | 0 | 0 | 0 | - | - | 0 | 0 |
| Q15012 | Lysosomal-associated transmembrane protein 4A | LAPTM4A | MVSMSFKRNR | 0 | 0 | 0 | 0 | 0 | 0 | 0 | 0 |
| P55735 | Protein SEC13 homolog | SEC13 | MVSVINTVDT | 0 | 0 | 0 | 0 | 0 | 0 | 0 | 0 |
| Q9BU89 | Deoxyhypusine hydroxylase | DOHH | MVTEQEVDAI | + | + | + | + | +/- | + | +/- | 0 |
| P06396-2 | Isoform 2 of Gelsolin | GSN | MVVEHPEFLK | - | - | - | - | 0 | 0 | - | +/- |
| Q8IUX1 | Complex I assembly factor TMEM126B, mitochondrial | TMEM126B | MVVFGYEAGT | - | 0 | - | 0 | - | - | 0 | 0 |
| Q9H6K4 | Optic atrophy 3 protein | OPA3 | MVVGAFPMAK | 0 | 0 | - | 0 | 0 | 0 | 0 | 0 |
| Q9NPA8 | Transcription and mRNA export factor ENY2 | ENY2 | MVVSKMNKDA | 0 | 0 | 0 | 0 | - | 0 | 0 | 0 |
| Q9Y6D0 | Selenoprotein K | SELK | MVYISNGQVL | - | 0 | 0 | 0 | - | - | 0 | 0 |
| Q14247 | Src substrate cortactin | CTTN | MWKASAGHAV | 0 | 0 | 0 | 0 | 0 | 0 | 0 | 0 |
| O60934 | Nibrin | NBN | MWKLLPAAGP | 0 | 0 | + | 0 | 0 | 0 | 0 | 0 |
| P15927 | Replication protein A 32 kDa subunit | RPA2 | MWNSGFESYG | 0 | 0 | + | 0 | 0 | 0 | 0 | 0 |
| Q96EX1 | Small integral membrane protein 12 | SMIM12 | MWPVFWTVVR | 0 | 0 | + | 0 | 0 | 0 | 0 | 0 |
| Q6P3X3 | Tetratricopeptide repeat protein 27 | TTC27 | MWTPELAILR | + | + | + | + | 0 | + | 0 | + |
| O75204 | Transmembrane protein 127 | TMEM127 | MYAPGGAGLP | + | + | 0 | 0 | + | 0 | 0 | 0 |
| Q9UQE7 | Structural maintenance of chromosomes protein 3 | SMC3 | MYIKQVIIQG | + | + | + | + | + | + | + | + |
| Q9BQA9 | Uncharacterized protein C17orf62 | C17orf62 | MYLQVETRTS | 0 | + | 0 | 0 | + | + | 0 | 0 |
| Q9UQ35-2 | Isoform 2 of Serine/arginine repetitive matrix protein 2 | SRRM2 | MYNGIGLPTP | 0 | 0 | 0 | 0 | 0 | 0 | 0 | 0 |
| O43581 | Synaptotagmin-7 | SYT7 | MYRDPEAASP | - | 0 | 0 | 0 | 0 | 0 | 0 | 0 |
| P40121 | Macrophage-capping protein | CAPG | MYTAIPQSGS | + | 0 | 0 | 0 | 0 | 0 | 0 | + |
| Q8NG68 | Tubulin--tyrosine ligase | TTL | MYTFVVRDEN | 0 | 0 | 0 | 0 | 0 | 0 | 0 | 0 |
| Q9BUT9 | Protein FAM195A | FAM195A | MYTITKGPSK | 0 | 0 | + | 0 | 0 | 0 | 0 | 0 |
| P62861 | 40S ribosomal protein S30 | FAU | KVHGSLARAG | 0 | 0 | 0 | 0 | 0 | 0 | 0 | 0 |
