# Supplementary material for: MetAP1 and MetAP2 drive cell selectivity for a potent anti-cancer agent in synergy, by controlling glutathione redox state
Source: Oncotarget. 2016 Aug 11;7(39):63306–23. doi: 10.18632/oncotarget.11216 (PMC5325365; doi:10.18632/oncotarget.11216)
Supplement: Supplementary file 3 [file oncotarget-07-63306-s003.docx]

**Supplemental Table 2. The complete list of transitions measured for MetAP1 and MetAP2.**

| **Transitions for MetAP1 and MetAP2: Relative quantification experiment** | | | | | | | | | |
| --- | --- | --- | --- | --- | --- | --- | --- | --- | --- |
| **Protein Name** | **Accesion number** | **Peptide Sequence** | **Light** | | | **Heavy** | | | **Retention time (Minutes)** |
|  |  |  | **Precursor m/z  (charge)** | **Product m/z (Ion type, charge)** | **Optimized CE  (Volts)** | **Precursor m/z  (charge)** | **Product m/z (Ion type, charge)** | **Optimized CE  (Volts)** |  |
| **Methionine aminopeptidase 2** | **P50579** | ALDQASEEIWNDFR | 847.394458 (2+) | 1195.537828 (1+, y9) | 26.3 | 852.398593 (2+) | 1205.546097 (1+, y9) | 26.3 | 37 |
|  |  |  |  | 979.463206 (1+, y7) | 26.3 |  | 989.471475 (1+, y7) | 26.3 |  |
|  |  |  |  | 850.420613 (1+, y6) | 26.3 |  | 860.428882 (1+, y6) | 26.3 |  |
|  |  | IDFGTHISGR | 551.78563 (2+) | 569.31542 (1+, y5) | 21.5 | 556.789764 (2+) | 579.323689 (1+, y5) | 21.5 | 21.9 |
|  |  |  |  | 432.256508 (1+, y4) | 21.5 |  | 442.264777 (1+, y4) | 21.5 |  |
|  |  |  |  | 319.172444 (1+, y3) | 19.5 |  | 329.180713 (1+, y3) | 19.5 |  |
|  |  | NLNGHSIGQYR | 629.817992 (2+) | 1031.501717 (1+, y9) | 21.8 | 634.822127 (2+) | 1041.509986 (1+, y9) | 21.8 | 17.2 |
|  |  |  |  | 723.378414 (1+, y6) | 25.8 |  | 733.386683 (1+, y6) | 25.8 |  |
|  |  |  |  | 636.346386 (1+, y5) | 21.8 |  | 646.354655 (1+, y5) | 21.8 |  |
| **Methionine aminopeptidase 1** | **P53582** | HAQANGFSVVR | 593.309803 (2+) | 1048.553418 (1+, y10) | 24.7 | 598.313938 (2+) | 1058.561687 (1+, y10) | 24.7 | 18.8 |
|  |  |  |  | 977.516305 (1+, y9) | 22.7 |  | 987.524574 (1+, y9) | 22.7 |  |
|  |  |  |  | 849.457727 (1+, y8) | 22.7 |  | 859.465996 (1+, y8) | 22.7 |  |
|  |  | LQC[+57.0]PTC[+57.0]IK | 510.254262 (2+) | 778.358607 (1+, y6) | 16.2 | 514.261362 (2+) | 786.372806 (1+, y6) | 16.2 | 17.7 |
|  |  |  |  | 618.327959 (1+, y5) | 20.2 |  | 626.342158 (1+, y5) | 20.2 |  |
|  |  |  |  | 521.275195 (1+, y4) | 24.2 |  | 529.289394 (1+, y4) | 24.2 |  |
|  |  | LGIQGSYFC[+57.0]SQEC[+57.0]FK | 912.408197 (2+) | 1412.560949 (1+, y11) | 30.3 | 916.415296 (2+) | 1420.575148 (1+, y11) | 30.3 | 30.9 |
|  |  |  |  | 1268.507456 (1+, y9) | 28.3 |  | 1276.521655 (1+, y9) | 28.3 |  |
|  |  |  |  | 1105.444128 (1+, y8) | 28.3 |  | 1113.458327 (1+, y8) | 28.3 |  |
|  |  |  |  |  |  |  |  |  |  |
|  |  |  |  |  |  |  |  |  |  |
| **Transitions for MetAP2: Absolute quantification experiment** | | | | | | | | | |
| **Protein Name** | **Accesion number** | **Peptide Sequence** | **Light** | | | **Heavy** | | | **Retention time (Minutes)** |
|  |  |  | **Precursor m/z  (charge)** | **Product m/z (Ion type, charge)** | **Optimized CE  (Volts)** | **Precursor m/z  (charge)** | **Product m/z (Ion type, charge)** | **Optimized CE  (Volts)** |  |
| **Methionine aminopeptidase 2** | **P50579** | IDFGTHISGR | 551.78563 (2+) | 569.31542 (1+, y5) | 21.5 | 556.789764 (2+) | 579.323689 (1+, y5) | 21.5 | 22.8 |
|  |  |  |  | 432.256508 (1+, y4) | 21.5 |  | 442.264777 (1+, y4) | 21.5 |  |
|  |  |  |  | 319.172444 (1+, y3) | 19.5 |  | 329.180713 (1+, y3) | 19.5 |  |
|  |  |  | 371,528935 (3+) | 329.180713 (1+, y3) | 24.8 | 368,192845 (3+) | 319.172444 (1+, y3) | 24.8 | 22.8 |
|  |  |  |  | 579.323689 (1+, y5) | 22.8 |  | 569.31542 (1+, y5) | 22.8 |  |
|  |  |  |  | 737.392831 (1+, y7) | 22.8 |  | 727.384562 (1+, y7) | 22.8 |  |

**Relative quantification**

| **Retention time** | | | | | | | | | | | | | | | | | |
| --- | --- | --- | --- | --- | --- | --- | --- | --- | --- | --- | --- | --- | --- | --- | --- | --- | --- |
|  |  |  |  |  |  |  |  |  |  |  |  |  |  |  |  |  |  |
| **Raw DATA** | | | | | | | | | | | | | | | | | |
|  |  |  |  |  |  |  | H1 | H2 | H3 | H4 | U2 | U3 | U4 | K1 | K2 | K3 | K4 |
| **Peptide Sequence** | **Protein Name** | **Precursor Mz** | **Precursor Charge** | **Product Mz** | **Product Charge** | **Fragment Ion** | **H1-N10570 Retention Time** | **H2-N10576 Retention Time** | **H3-N10582 Retention Time** | **H4-N10588 Retention Time** | **U2-N10574 Retention Time** | **U3-N10580 Retention Time** | **U4-N10586 Retention Time** | **K1-N10572 Retention Time** | **K2-N10578 Retention Time** | **K3-N10584 Retention Time** | **K4-N10590 Retention Time** |
| ALDQASEEIWNDFR | sp\|P50579\|AMPM2_HUMAN | 847.394458 | 2 | 1195.53783 | 1 | y9 | 37.21 | 37.21 | 36.95 | 36.95 | 37.05 | 36.9 | 36.85 | 37.1 | 37 | 37 | 36.9 |
| ALDQASEEIWNDFR | sp\|P50579\|AMPM2_HUMAN | 847.394458 | 2 | 979.463206 | 1 | y7 | 37.21 | 37.21 | 36.95 | 36.7 | 36.9 | 36.9 | 36.85 | 37.16 | 37 | 36.85 | 36.9 |
| ALDQASEEIWNDFR | sp\|P50579\|AMPM2_HUMAN | 847.394458 | 2 | 850.420613 | 1 | y6 | 37.05 | 37.21 | 37 | 36.9 | 36.95 | 36.8 | 36.9 | 37.05 | 37 | 36.9 | 36.85 |
| ALDQASEEIWNDFR | sp\|P50579\|AMPM2_HUMAN | 852.398593 | 2 | 1205.5461 | 1 | y9 | 37.21 | 37.21 | 37.05 | 36.95 | 37 | 36.95 | 36.85 | 37.1 | 37 | 36.95 | 36.9 |
| ALDQASEEIWNDFR | sp\|P50579\|AMPM2_HUMAN | 852.398593 | 2 | 989.471475 | 1 | y7 | 37.21 | 37.21 | 37 | 36.95 | 37 | 36.95 | 36.85 | 37.1 | 37 | 36.95 | 36.9 |
| ALDQASEEIWNDFR | sp\|P50579\|AMPM2_HUMAN | 852.398593 | 2 | 860.428882 | 1 | y6 | 37.21 | 37.21 | 37.05 | 36.95 | 37 | 36.95 | 36.85 | 37.1 | 37 | 37 | 36.9 |
| IDFGTHISGR | sp\|P50579\|AMPM2_HUMAN | 551.78563 | 2 | 569.31542 | 1 | y5 | 22.25 | 22.14 | 22.25 | 21.84 | 22.04 | 21.94 | 21.94 | 22.04 | 21.94 | 21.89 | 21.84 |
| IDFGTHISGR | sp\|P50579\|AMPM2_HUMAN | 551.78563 | 2 | 432.256508 | 1 | y4 | 21.99 | 22.14 | 21.99 | 21.79 | 21.94 | 22.04 | 21.79 | 22.19 | 21.94 | 21.94 | 21.84 |
| IDFGTHISGR | sp\|P50579\|AMPM2_HUMAN | 551.78563 | 2 | 319.172444 | 1 | y3 | 22.04 | 22.09 | 21.94 | 21.89 | 22.04 | 22.09 | 21.84 | 22.04 | 21.99 | 21.89 | 21.84 |
| IDFGTHISGR | sp\|P50579\|AMPM2_HUMAN | 556.789764 | 2 | 579.323689 | 1 | y5 | 22.04 | 22.09 | 21.99 | 21.84 | 21.94 | 21.94 | 21.79 | 22.09 | 21.94 | 21.89 | 21.79 |
| IDFGTHISGR | sp\|P50579\|AMPM2_HUMAN | 556.789764 | 2 | 442.264777 | 1 | y4 | 22.04 | 22.14 | 21.99 | 21.84 | 21.94 | 21.94 | 21.79 | 22.09 | 21.94 | 21.89 | 21.84 |
| IDFGTHISGR | sp\|P50579\|AMPM2_HUMAN | 556.789764 | 2 | 329.180713 | 1 | y3 | 22.04 | 22.14 | 21.99 | 21.84 | 21.94 | 21.94 | 21.79 | 22.09 | 21.94 | 21.89 | 21.84 |
| NLNGHSIGQYR | sp\|P50579\|AMPM2_HUMAN | 629.817992 | 2 | 1031.50172 | 1 | y9 | 17.31 | 17.52 | 17.41 | 17.11 | 17.31 | 17.36 | 17.16 | 17.46 | 17.21 | 17.16 | 17.16 |
| NLNGHSIGQYR | sp\|P50579\|AMPM2_HUMAN | 629.817992 | 2 | 723.378414 | 1 | y6 | 17.36 | 17.46 | 17.36 | 17.16 | 17.36 | 17.31 | 17.16 | 17.46 | 17.26 | 17.16 | 17.16 |
| NLNGHSIGQYR | sp\|P50579\|AMPM2_HUMAN | 629.817992 | 2 | 636.346386 | 1 | y5 | 17.36 | 17.46 | 17.36 | 17.16 | 17.36 | 17.26 | 17.31 | 17.41 | 17.26 | 17.21 | 17.06 |
| NLNGHSIGQYR | sp\|P50579\|AMPM2_HUMAN | 634.822127 | 2 | 1041.50999 | 1 | y9 | 17.36 | 17.46 | 17.36 | 17.16 | 17.31 | 17.36 | 17.21 | 17.41 | 17.26 | 17.21 | 17.11 |
| NLNGHSIGQYR | sp\|P50579\|AMPM2_HUMAN | 634.822127 | 2 | 733.386683 | 1 | y6 | 17.36 | 17.52 | 17.36 | 17.11 | 17.31 | 17.36 | 17.16 | 17.41 | 17.26 | 17.21 | 17.16 |
| NLNGHSIGQYR | sp\|P50579\|AMPM2_HUMAN | 634.822127 | 2 | 646.354655 | 1 | y5 | 17.31 | 17.52 | 17.36 | 17.16 | 17.31 | 17.36 | 17.16 | 17.41 | 17.26 | 17.21 | 17.16 |
| LQCPTCIK | sp\|P53582\|AMPM1_HUMAN | 510.254262 | 2 | 778.358607 | 1 | y6 | 17.91 | 18.11 | 17.91 | 17.65 | 17.91 | 17.91 | 17.75 | 18.06 | 17.91 | 17.8 | 17.75 |
| LQCPTCIK | sp\|P53582\|AMPM1_HUMAN | 510.254262 | 2 | 618.327959 | 1 | y5 | 17.96 | 18.06 | 17.91 | 17.75 | 17.91 | 17.91 | 17.75 | 18.06 | 17.86 | 17.8 | 17.75 |
| LQCPTCIK | sp\|P53582\|AMPM1_HUMAN | 510.254262 | 2 | 521.275195 | 1 | y4 | 17.91 | 18.11 | 17.96 | 17.7 | 17.86 | 17.91 | 17.75 | 18.06 | 17.91 | 17.8 | 17.8 |
| LQCPTCIK | sp\|P53582\|AMPM1_HUMAN | 514.261362 | 2 | 786.372806 | 1 | y6 | 17.91 | 18.11 | 17.91 | 17.7 | 17.91 | 17.91 | 17.75 | 18.06 | 17.86 | 17.8 | 17.75 |
| LQCPTCIK | sp\|P53582\|AMPM1_HUMAN | 514.261362 | 2 | 626.342158 | 1 | y5 | 17.91 | 18.06 | 17.91 | 17.7 | 17.91 | 17.96 | 17.75 | 17.96 | 17.86 | 17.86 | 17.75 |
| LQCPTCIK | sp\|P53582\|AMPM1_HUMAN | 514.261362 | 2 | 529.289394 | 1 | y4 | 17.91 | 18.06 | 17.91 | 17.7 | 17.91 | 17.91 | 17.75 | 18.06 | 17.91 | 17.86 | 17.75 |
| LGIQGSYFCSQECFK | sp\|P53582\|AMPM1_HUMAN | 912.408197 | 2 | 1412.56095 | 1 | y11 | 30.99 | 31.09 | 30.99 | 30.89 | 30.94 | 30.89 | 30.78 | 31.04 | 30.83 | 30.89 | 30.83 |
| LGIQGSYFCSQECFK | sp\|P53582\|AMPM1_HUMAN | 912.408197 | 2 | 1268.50746 | 1 | y9 | 30.99 | 31.19 | 31.4 | 30.73 | 30.83 | 30.83 | 30.78 | 30.89 | 30.89 | 30.83 | 30.78 |
| LGIQGSYFCSQECFK | sp\|P53582\|AMPM1_HUMAN | 912.408197 | 2 | 1105.44413 | 1 | y8 | 30.99 | 31.14 | 31.04 | 30.78 | 30.89 | 30.94 | 30.83 | 30.99 | 30.94 | 30.94 | 30.78 |
| LGIQGSYFCSQECFK | sp\|P53582\|AMPM1_HUMAN | 916.415296 | 2 | 1420.57515 | 1 | y11 | 30.99 | 31.09 | 30.89 | 30.83 | 30.94 | 30.89 | 30.78 | 31.04 | 30.89 | 30.83 | 30.78 |
| LGIQGSYFCSQECFK | sp\|P53582\|AMPM1_HUMAN | 916.415296 | 2 | 1276.52166 | 1 | y9 | 30.99 | 31.09 | 30.89 | 30.78 | 30.94 | 30.89 | 30.78 | 31.04 | 30.94 | 30.83 | 30.78 |
| LGIQGSYFCSQECFK | sp\|P53582\|AMPM1_HUMAN | 916.415296 | 2 | 1113.45833 | 1 | y8 | 31.04 | 31.09 | 30.89 | 30.83 | 30.94 | 30.89 | 30.78 | 31.04 | 30.94 | 30.83 | 30.78 |
| HAQANGFSVVR | sp\|P53582\|AMPM1_HUMAN | 593.309803 | 2 | 1048.55342 | 1 | y10 | 19.04 | 19.25 | 18.89 | 18.89 | 18.89 | 18.89 | 18.79 | 18.99 | 18.94 | 18.89 | 18.79 |
| HAQANGFSVVR | sp\|P53582\|AMPM1_HUMAN | 593.309803 | 2 | 977.516305 | 1 | y9 | 19.04 | 19.14 | 19.04 | 18.84 | 18.89 | 18.84 | 18.79 | 19.04 | 18.89 | 18.84 | 18.79 |
| HAQANGFSVVR | sp\|P53582\|AMPM1_HUMAN | 593.309803 | 2 | 849.457727 | 1 | y8 | 19.25 | 19.2 | 18.99 | 18.89 | 18.89 | 18.99 | 18.74 | 19.04 | 18.94 | 18.94 | 18.84 |
| HAQANGFSVVR | sp\|P53582\|AMPM1_HUMAN | 598.313938 | 2 | 1058.56169 | 1 | y10 | 18.99 | 19.14 | 19.04 | 18.79 | 18.89 | 18.94 | 18.74 | 19.09 | 18.94 | 18.84 | 18.84 |
| HAQANGFSVVR | sp\|P53582\|AMPM1_HUMAN | 598.313938 | 2 | 987.524574 | 1 | y9 | 18.99 | 19.14 | 19.04 | 18.84 | 18.89 | 18.94 | 18.74 | 19.09 | 18.94 | 18.84 | 18.79 |
| HAQANGFSVVR | sp\|P53582\|AMPM1_HUMAN | 598.313938 | 2 | 859.465996 | 1 | y8 | 18.99 | 19.14 | 19.04 | 18.84 | 18.89 | 18.94 | 18.74 | 19.09 | 18.89 | 18.84 | 18.79 |

| **Statistical description** | | | | |
| --- | --- | --- | --- | --- |
| **Protein Name** | **Peptide Sequence** | **Retention time Mean** | **Standard deviation** | **CV (%)** |
| sp\|P50579\|AMPM2_HUMAN | ALDQASEEIWNDFR | 37.0 | 0.12 | 0.3 |
| sp\|P50579\|AMPM2_HUMAN | IDFGTHISGR | 22.0 | 0.12 | 0.5 |
| sp\|P50579\|AMPM2_HUMAN | NLNGHSIGQYR | 17.3 | 0.12 | 0.7 |
| sp\|P53582\|AMPM1_HUMAN | LQCPTCIK | 17.9 | 0.12 | 0.7 |
| sp\|P53582\|AMPM1_HUMAN | LGIQGSYFCSQECFK | 30.9 | 0.12 | 0.4 |
| sp\|P53582\|AMPM1_HUMAN | HAQANGFSVVR | 18.9 | 0.13 | 0.7 |

| **Total Area Ratio** | | | | | | | | | | | | | | | | | |
| --- | --- | --- | --- | --- | --- | --- | --- | --- | --- | --- | --- | --- | --- | --- | --- | --- | --- |
|  |  |  |  |  |  |  |  |  |  |  |  |  |  |  |  |  |  |
| **Raw Data** | | | | | | | | | | | | | | |  |  |  |
|  |  |  |  |  |  |  |  |  |  |  |  |  |  |  |  |  |  |
|  |  |  |  | Total Area Ratio | Total Area Ratio | Total Area Ratio | Total Area Ratio | Total Area Ratio | Total Area Ratio | Total Area Ratio | Total Area Ratio | Total Area Ratio | Total Area Ratio | Total Area Ratio |  |  |  |
|  |  |  |  | H1 | H2 | H3 | H4 | U2 | U3 | U4 | K1 | K2 | K3 | K4 |  |  |  |
| Peptide Sequence | Protein Name | Precursor Mz | Precursor Charge | H1-N10570 Total Area Ratio | H2-N10576 Total Area Ratio | H3-N10582 Total Area Ratio | H4-N10588 Total Area Ratio | U2-N10574 Total Area Ratio | U3-N10580 Total Area Ratio | U4-N10586 Total Area Ratio | K1-N10572 Total Area Ratio | K2-N10578 Total Area Ratio | K3-N10584 Total Area Ratio | K4-N10590 Total Area Ratio |  |  |  |
| NLNGHSIGQYR | sp\|P50579\|AMPM2_HUMAN | 629.817992 | 2 | 0.0144 | 0.0128 | 0.011 | 0.0166 | 0.0143 | 0.0159 | 0.0191 | 0.0327 | 0.0315 | 0.0282 | 0.0342 |  |  |  |
| ALDQASEEIWNDFR | sp\|P50579\|AMPM2_HUMAN | 847.394458 | 2 | 0.0214 | 0.026 | 0.0328 | 0.02 | 0.0165 | 0.0252 | 0.0226 | 0.062 | 0.0469 | 0.0532 | 0.0625 |  |  |  |
| IDFGTHISGR | sp\|P50579\|AMPM2_HUMAN | 551.78563 | 2 | 0.0221 | 0.0201 | 0.0224 | 0.0159 | 0.0143 | 0.0149 | 0.0133 | 0.0426 | 0.0433 | 0.0475 | 0.0539 |  |  |  |
| LGIQGSYFCSQECFK | sp\|P53582\|AMPM1_HUMAN | 912.408197 | 2 | 0.0112 | 0.0132 | 0.0132 | 0.011 | 0.0114 | 0.0155 | 0.013 | 0.0192 | 0.0187 | 0.0169 | 0.0165 |  |  |  |
| HAQANGFSVVR | sp\|P53582\|AMPM1_HUMAN | 593.309803 | 2 | 0.0156 | 0.0168 | 0.0202 | 0.0217 | 0.0265 | 0.0259 | 0.0259 | 0.0288 | 0.0258 | 0.0289 | 0.0263 |  |  |  |
| LQCPTCIK | sp\|P53582\|AMPM1_HUMAN | 510.254262 | 2 | 0.2427 | 0.2181 | 0.218 | 0.1959 | 0.263 | 0.2425 | 0.2227 | 0.3181 | 0.3196 | 0.3484 | 0.3205 |  |  |  |
|  |  |  |  |  |  |  |  |  |  |  |  |  |  |  |  |  |  |
|  |  |  |  |  |  |  |  |  |  |  |  |  |  |  |  |  |  |
| **Statistical description** | | | | | | | |  |  |  |  |  |  |  |  |  |  |
|  |  | **ALDQASEEIWNDFR** | **IDFGTHISGR** | **NLNGHSIGQYR** | **LQCPTCIK** | **LGIQGSYFCSQECFK** | **HAQANGFSVVR** |  |  |  |  |  |  |  |  |  |  |
| **Mean** | **HUVEC** | <LOD | 0.020125 | 0.0137 | 0.218675 | 0.01215 | 0.018575 |  |  |  |  |  |  |  |  |  |  |
|  | **U87** | <LOD | 0.01416667 | 0.01643333 | 0.24273333 | 0.0133 | 0.0261 |  |  |  |  |  |  |  |  |  |  |
|  | **K562** | 0.05615 | 0.046825 | 0.03165 | 0.32665 | 0.017825 | 0.02745 |  |  |  |  |  |  |  |  |  |  |
| **Standard deviation** | **HUVEC** | <LOD | 0.00299597 | 0.00238048 | 0.01911969 | 0.00121518 | 0.00285234 |  |  |  |  |  |  |  |  |  |  |
|  | **U87** | <LOD | 0.00080829 | 0.00244404 | 0.02015101 | 0.0020664 | 0.00034641 |  |  |  |  |  |  |  |  |  |  |
|  | **K562** | 0.00750133 | 0.00518933 | 0.00255147 | 0.01453375 | 0.00132508 | 0.00162993 |  |  |  |  |  |  |  |  |  |  |
| **CV (%)** | **HUVEC** | <LOD | 14.886805 | 17.3757383 | 8.74342691 | 10.0014958 | 15.3557912 |  |  |  |  |  |  |  |  |  |  |
|  | **U87** | <LOD | 5.70557913 | 14.8724566 | 8.30170827 | 15.5368258 | 1.327242 |  |  |  |  |  |  |  |  |  |  |
|  | **K562** | 13.3594536 | 11.0823962 | 8.06151711 | 4.44933532 | 7.43382112 | 5.93780847 |  |  |  |  |  |  |  |  |  |  |

| **MsStats Results** | | | | |
| --- | --- | --- | --- | --- |
|  |  |  |  |  |
| **Comparison K562-HUVEC** | | | | |
|  | **ajusted p-value** | **Fold change** | **-LOG(p-value)** | **LOG2(Fold change)** |
| **NLNGHSIGQYR** | 2.56E-07 | 2.2775858 | 6.592100107 | 1.187505406 |
| **ALDQASEEIWNDFR** | <LOD | <LOD |  |  |
| **IDFGTHISGR** | 2.18E-09 | 2.2891481 | 8.660644127 | 1.194810805 |
| **Protein MetAP2** | 1.00E-15 | 2.29222792 | 15 | 1.196750502 |
| **LGIQGSYFCSQECFK** | 0.000451152 | 1.45642856 | 3.345677162 | 0.542434939 |
| **HAQANGFSVVR** | 1.31E-06 | 1.4880394 | 5.882719278 | 0.573412729 |
| **LQCPTCIK** | 0.000252745 | 1.37320314 | 3.597316688 | 0.457545057 |
| **Protein MetAP1** | 2.75779E-12 | 1.43399424 | 11.55943818 | 0.520039229 |
|  |  |  |  |  |
|  |  |  |  |  |
| **Comparison K562-U87** | | | | |
|  | **ajusted p-value** | **Fold change** | **-LOG(p-value)** | **LOG2(Fold change)** |
| **NLNGHSIGQYR** | 1.64E-05 | 1.91098416 | 4.784510452 | 0.934315821 |
| **ALDQASEEIWNDFR** | <LOD | <LOD |  |  |
| **IDFGTHISGR** | 4.02E-11 | 3.23781735 | 10.3953872 | 1.695021603 |
| **Protein MetAP2** | 1.00E-15 | 2.59341884 | 15 | 1.374855221 |
| **LGIQGSYFCSQECFK** | 0.002489479 | 1.42108757 | 2.603891548 | 0.506995463 |
| **HAQANGFSVVR** | 3.81E-01 | 1.05222733 | 0.418880901 | 0.073446425 |
| **LQCPTCIK** | 0.017461646 | 1.22169442 | 1.757914818 | 0.288883476 |
| **Protein MetAP1** | 1.44648E-05 | 1.2400822 | 4.839686599 | 0.310435753 |
|  |  |  |  |  |
|  |  |  |  |  |
| **Comparison U87-HUVEC** | | | | |
|  | **ajusted p-value** | **Fold change** | **-LOG(p-value)** | **LOG2(Fold change)** |
| **NLNGHSIGQYR** | 1.50E-01 | 1.19183918 | 0.822774871 | 0.253189584 |
| **ALDQASEEIWNDFR** | <LOD | <LOD |  |  |
| **IDFGTHISGR** | 2.64E-04 | 0.70700347 | 3.579060493 | -0.500210799 |
| **Protein MetAP2** | 2.59E-02 | 0.88386337 | 1.586404445 | -0.178104718 |
| **LGIQGSYFCSQECFK** | 0.79526993 | 1.02486897 | 0.099485438 | 0.035439476 |
| **HAQANGFSVVR** | 8.70E-05 | 1.41418053 | 4.060700268 | 0.499966304 |
| **LQCPTCIK** | 0.155175577 | 1.12401523 | 0.809176631 | 0.168661581 |
| **Protein MetAP1** | 0.004666406 | 1.15637031 | 2.331017473 | 0.209603475 |

| **Raw Data** | | | | | | | | | |
| --- | --- | --- | --- | --- | --- | --- | --- | --- | --- |
| ProteinName | PeptideSequence | PrecursorCharge | FragmentIon | ProductCharge | IsotopeLabelType | Condition | BioReplicate | Run | Area |
| sp\|P50579\|AMPM2_HUMAN | ALDQASEEIWNDFR | 2 | y9 | 1 | light | H | H1 | 1 | 2679 |
| sp\|P50579\|AMPM2_HUMAN | ALDQASEEIWNDFR | 2 | y7 | 1 | light | H | H1 | 1 | 3056 |
| sp\|P50579\|AMPM2_HUMAN | ALDQASEEIWNDFR | 2 | y6 | 1 | light | H | H1 | 1 | 2205 |
| sp\|P50579\|AMPM2_HUMAN | ALDQASEEIWNDFR | 2 | y9 | 1 | heavy | H | H1 | 1 | 167713 |
| sp\|P50579\|AMPM2_HUMAN | ALDQASEEIWNDFR | 2 | y7 | 1 | heavy | H | H1 | 1 | 97767 |
| sp\|P50579\|AMPM2_HUMAN | ALDQASEEIWNDFR | 2 | y6 | 1 | heavy | H | H1 | 1 | 106210 |
| sp\|P50579\|AMPM2_HUMAN | IDFGTHISGR | 2 | y5 | 1 | light | H | H1 | 1 | 13238 |
| sp\|P50579\|AMPM2_HUMAN | IDFGTHISGR | 2 | y4 | 1 | light | H | H1 | 1 | 11431 |
| sp\|P50579\|AMPM2_HUMAN | IDFGTHISGR | 2 | y3 | 1 | light | H | H1 | 1 | 6338 |
| sp\|P50579\|AMPM2_HUMAN | IDFGTHISGR | 2 | y5 | 1 | heavy | H | H1 | 1 | 306310 |
| sp\|P50579\|AMPM2_HUMAN | IDFGTHISGR | 2 | y4 | 1 | heavy | H | H1 | 1 | 813298 |
| sp\|P50579\|AMPM2_HUMAN | IDFGTHISGR | 2 | y3 | 1 | heavy | H | H1 | 1 | 280355 |
| sp\|P50579\|AMPM2_HUMAN | NLNGHSIGQYR | 2 | y9 | 1 | light | H | H1 | 1 | 3241 |
| sp\|P50579\|AMPM2_HUMAN | NLNGHSIGQYR | 2 | y6 | 1 | light | H | H1 | 1 | 4267 |
| sp\|P50579\|AMPM2_HUMAN | NLNGHSIGQYR | 2 | y5 | 1 | light | H | H1 | 1 | 2519 |
| sp\|P50579\|AMPM2_HUMAN | NLNGHSIGQYR | 2 | y9 | 1 | heavy | H | H1 | 1 | 160351 |
| sp\|P50579\|AMPM2_HUMAN | NLNGHSIGQYR | 2 | y6 | 1 | heavy | H | H1 | 1 | 411799 |
| sp\|P50579\|AMPM2_HUMAN | NLNGHSIGQYR | 2 | y5 | 1 | heavy | H | H1 | 1 | 125851 |
| sp\|P50579\|AMPM2_HUMAN | ALDQASEEIWNDFR | 2 | y9 | 1 | light | K | K1 | 2 | 11370 |
| sp\|P50579\|AMPM2_HUMAN | ALDQASEEIWNDFR | 2 | y7 | 1 | light | K | K1 | 2 | 5939 |
| sp\|P50579\|AMPM2_HUMAN | ALDQASEEIWNDFR | 2 | y6 | 1 | light | K | K1 | 2 | 7425 |
| sp\|P50579\|AMPM2_HUMAN | ALDQASEEIWNDFR | 2 | y9 | 1 | heavy | K | K1 | 2 | 161493 |
| sp\|P50579\|AMPM2_HUMAN | ALDQASEEIWNDFR | 2 | y7 | 1 | heavy | K | K1 | 2 | 112395 |
| sp\|P50579\|AMPM2_HUMAN | ALDQASEEIWNDFR | 2 | y6 | 1 | heavy | K | K1 | 2 | 124794 |
| sp\|P50579\|AMPM2_HUMAN | IDFGTHISGR | 2 | y5 | 1 | light | K | K1 | 2 | 16712 |
| sp\|P50579\|AMPM2_HUMAN | IDFGTHISGR | 2 | y4 | 1 | light | K | K1 | 2 | 23132 |
| sp\|P50579\|AMPM2_HUMAN | IDFGTHISGR | 2 | y3 | 1 | light | K | K1 | 2 | 15059 |
| sp\|P50579\|AMPM2_HUMAN | IDFGTHISGR | 2 | y5 | 1 | heavy | K | K1 | 2 | 288626 |
| sp\|P50579\|AMPM2_HUMAN | IDFGTHISGR | 2 | y4 | 1 | heavy | K | K1 | 2 | 751329 |
| sp\|P50579\|AMPM2_HUMAN | IDFGTHISGR | 2 | y3 | 1 | heavy | K | K1 | 2 | 248205 |
| sp\|P50579\|AMPM2_HUMAN | NLNGHSIGQYR | 2 | y9 | 1 | light | K | K1 | 2 | 6813 |
| sp\|P50579\|AMPM2_HUMAN | NLNGHSIGQYR | 2 | y6 | 1 | light | K | K1 | 2 | 10323 |
| sp\|P50579\|AMPM2_HUMAN | NLNGHSIGQYR | 2 | y5 | 1 | light | K | K1 | 2 | 2394 |
| sp\|P50579\|AMPM2_HUMAN | NLNGHSIGQYR | 2 | y9 | 1 | heavy | K | K1 | 2 | 146629 |
| sp\|P50579\|AMPM2_HUMAN | NLNGHSIGQYR | 2 | y6 | 1 | heavy | K | K1 | 2 | 346811 |
| sp\|P50579\|AMPM2_HUMAN | NLNGHSIGQYR | 2 | y5 | 1 | heavy | K | K1 | 2 | 104704 |
| sp\|P50579\|AMPM2_HUMAN | ALDQASEEIWNDFR | 2 | y9 | 1 | light | U | U2 | 3 | 1379 |
| sp\|P50579\|AMPM2_HUMAN | ALDQASEEIWNDFR | 2 | y7 | 1 | light | U | U2 | 3 | 2852 |
| sp\|P50579\|AMPM2_HUMAN | ALDQASEEIWNDFR | 2 | y6 | 1 | light | U | U2 | 3 | 2267 |
| sp\|P50579\|AMPM2_HUMAN | ALDQASEEIWNDFR | 2 | y9 | 1 | heavy | U | U2 | 3 | 167402 |
| sp\|P50579\|AMPM2_HUMAN | ALDQASEEIWNDFR | 2 | y7 | 1 | heavy | U | U2 | 3 | 115184 |
| sp\|P50579\|AMPM2_HUMAN | ALDQASEEIWNDFR | 2 | y6 | 1 | heavy | U | U2 | 3 | 112337 |
| sp\|P50579\|AMPM2_HUMAN | IDFGTHISGR | 2 | y5 | 1 | light | U | U2 | 3 | 7085 |
| sp\|P50579\|AMPM2_HUMAN | IDFGTHISGR | 2 | y4 | 1 | light | U | U2 | 3 | 6134 |
| sp\|P50579\|AMPM2_HUMAN | IDFGTHISGR | 2 | y3 | 1 | light | U | U2 | 3 | 5001 |
| sp\|P50579\|AMPM2_HUMAN | IDFGTHISGR | 2 | y5 | 1 | heavy | U | U2 | 3 | 285034 |
| sp\|P50579\|AMPM2_HUMAN | IDFGTHISGR | 2 | y4 | 1 | heavy | U | U2 | 3 | 747554 |
| sp\|P50579\|AMPM2_HUMAN | IDFGTHISGR | 2 | y3 | 1 | heavy | U | U2 | 3 | 243105 |
| sp\|P50579\|AMPM2_HUMAN | NLNGHSIGQYR | 2 | y9 | 1 | light | U | U2 | 3 | 3691 |
| sp\|P50579\|AMPM2_HUMAN | NLNGHSIGQYR | 2 | y6 | 1 | light | U | U2 | 3 | 4115 |
| sp\|P50579\|AMPM2_HUMAN | NLNGHSIGQYR | 2 | y5 | 1 | light | U | U2 | 3 | 1534 |
| sp\|P50579\|AMPM2_HUMAN | NLNGHSIGQYR | 2 | y9 | 1 | heavy | U | U2 | 3 | 153522 |
| sp\|P50579\|AMPM2_HUMAN | NLNGHSIGQYR | 2 | y6 | 1 | heavy | U | U2 | 3 | 388515 |
| sp\|P50579\|AMPM2_HUMAN | NLNGHSIGQYR | 2 | y5 | 1 | heavy | U | U2 | 3 | 110492 |
| sp\|P50579\|AMPM2_HUMAN | ALDQASEEIWNDFR | 2 | y9 | 1 | light | H | H2 | 4 | 3969 |
| sp\|P50579\|AMPM2_HUMAN | ALDQASEEIWNDFR | 2 | y7 | 1 | light | H | H2 | 4 | 4272 |
| sp\|P50579\|AMPM2_HUMAN | ALDQASEEIWNDFR | 2 | y6 | 1 | light | H | H2 | 4 | 2742 |
| sp\|P50579\|AMPM2_HUMAN | ALDQASEEIWNDFR | 2 | y9 | 1 | heavy | H | H2 | 4 | 177629 |
| sp\|P50579\|AMPM2_HUMAN | ALDQASEEIWNDFR | 2 | y7 | 1 | heavy | H | H2 | 4 | 122772 |
| sp\|P50579\|AMPM2_HUMAN | ALDQASEEIWNDFR | 2 | y6 | 1 | heavy | H | H2 | 4 | 122455 |
| sp\|P50579\|AMPM2_HUMAN | IDFGTHISGR | 2 | y5 | 1 | light | H | H2 | 4 | 10093 |
| sp\|P50579\|AMPM2_HUMAN | IDFGTHISGR | 2 | y4 | 1 | light | H | H2 | 4 | 11259 |
| sp\|P50579\|AMPM2_HUMAN | IDFGTHISGR | 2 | y3 | 1 | light | H | H2 | 4 | 10155 |
| sp\|P50579\|AMPM2_HUMAN | IDFGTHISGR | 2 | y5 | 1 | heavy | H | H2 | 4 | 366484 |
| sp\|P50579\|AMPM2_HUMAN | IDFGTHISGR | 2 | y4 | 1 | heavy | H | H2 | 4 | 885199 |
| sp\|P50579\|AMPM2_HUMAN | IDFGTHISGR | 2 | y3 | 1 | heavy | H | H2 | 4 | 319202 |
| sp\|P50579\|AMPM2_HUMAN | NLNGHSIGQYR | 2 | y9 | 1 | light | H | H2 | 4 | 3975 |
| sp\|P50579\|AMPM2_HUMAN | NLNGHSIGQYR | 2 | y6 | 1 | light | H | H2 | 4 | 3133 |
| sp\|P50579\|AMPM2_HUMAN | NLNGHSIGQYR | 2 | y5 | 1 | light | H | H2 | 4 | 2209 |
| sp\|P50579\|AMPM2_HUMAN | NLNGHSIGQYR | 2 | y9 | 1 | heavy | H | H2 | 4 | 165764 |
| sp\|P50579\|AMPM2_HUMAN | NLNGHSIGQYR | 2 | y6 | 1 | heavy | H | H2 | 4 | 433150 |
| sp\|P50579\|AMPM2_HUMAN | NLNGHSIGQYR | 2 | y5 | 1 | heavy | H | H2 | 4 | 130051 |
| sp\|P50579\|AMPM2_HUMAN | ALDQASEEIWNDFR | 2 | y9 | 1 | light | K | K2 | 5 | 8235 |
| sp\|P50579\|AMPM2_HUMAN | ALDQASEEIWNDFR | 2 | y7 | 1 | light | K | K2 | 5 | 5967 |
| sp\|P50579\|AMPM2_HUMAN | ALDQASEEIWNDFR | 2 | y6 | 1 | light | K | K2 | 5 | 6806 |
| sp\|P50579\|AMPM2_HUMAN | ALDQASEEIWNDFR | 2 | y9 | 1 | heavy | K | K2 | 5 | 192670 |
| sp\|P50579\|AMPM2_HUMAN | ALDQASEEIWNDFR | 2 | y7 | 1 | heavy | K | K2 | 5 | 126714 |
| sp\|P50579\|AMPM2_HUMAN | ALDQASEEIWNDFR | 2 | y6 | 1 | heavy | K | K2 | 5 | 128208 |
| sp\|P50579\|AMPM2_HUMAN | IDFGTHISGR | 2 | y5 | 1 | light | K | K2 | 5 | 19682 |
| sp\|P50579\|AMPM2_HUMAN | IDFGTHISGR | 2 | y4 | 1 | light | K | K2 | 5 | 23109 |
| sp\|P50579\|AMPM2_HUMAN | IDFGTHISGR | 2 | y3 | 1 | light | K | K2 | 5 | 12611 |
| sp\|P50579\|AMPM2_HUMAN | IDFGTHISGR | 2 | y5 | 1 | heavy | K | K2 | 5 | 300972 |
| sp\|P50579\|AMPM2_HUMAN | IDFGTHISGR | 2 | y4 | 1 | heavy | K | K2 | 5 | 712584 |
| sp\|P50579\|AMPM2_HUMAN | IDFGTHISGR | 2 | y3 | 1 | heavy | K | K2 | 5 | 266670 |
| sp\|P50579\|AMPM2_HUMAN | NLNGHSIGQYR | 2 | y9 | 1 | light | K | K2 | 5 | 6872 |
| sp\|P50579\|AMPM2_HUMAN | NLNGHSIGQYR | 2 | y6 | 1 | light | K | K2 | 5 | 10387 |
| sp\|P50579\|AMPM2_HUMAN | NLNGHSIGQYR | 2 | y5 | 1 | light | K | K2 | 5 | 4537 |
| sp\|P50579\|AMPM2_HUMAN | NLNGHSIGQYR | 2 | y9 | 1 | heavy | K | K2 | 5 | 172895 |
| sp\|P50579\|AMPM2_HUMAN | NLNGHSIGQYR | 2 | y6 | 1 | heavy | K | K2 | 5 | 402979 |
| sp\|P50579\|AMPM2_HUMAN | NLNGHSIGQYR | 2 | y5 | 1 | heavy | K | K2 | 5 | 115616 |
| sp\|P50579\|AMPM2_HUMAN | ALDQASEEIWNDFR | 2 | y9 | 1 | light | U | U3 | 6 | 3833 |
| sp\|P50579\|AMPM2_HUMAN | ALDQASEEIWNDFR | 2 | y7 | 1 | light | U | U3 | 6 | 3797 |
| sp\|P50579\|AMPM2_HUMAN | ALDQASEEIWNDFR | 2 | y6 | 1 | light | U | U3 | 6 | 2524 |
| sp\|P50579\|AMPM2_HUMAN | ALDQASEEIWNDFR | 2 | y9 | 1 | heavy | U | U3 | 6 | 177595 |
| sp\|P50579\|AMPM2_HUMAN | ALDQASEEIWNDFR | 2 | y7 | 1 | heavy | U | U3 | 6 | 111969 |
| sp\|P50579\|AMPM2_HUMAN | ALDQASEEIWNDFR | 2 | y6 | 1 | heavy | U | U3 | 6 | 113379 |
| sp\|P50579\|AMPM2_HUMAN | IDFGTHISGR | 2 | y5 | 1 | light | U | U3 | 6 | 7642 |
| sp\|P50579\|AMPM2_HUMAN | IDFGTHISGR | 2 | y4 | 1 | light | U | U3 | 6 | 7592 |
| sp\|P50579\|AMPM2_HUMAN | IDFGTHISGR | 2 | y3 | 1 | light | U | U3 | 6 | 6465 |
| sp\|P50579\|AMPM2_HUMAN | IDFGTHISGR | 2 | y5 | 1 | heavy | U | U3 | 6 | 341617 |
| sp\|P50579\|AMPM2_HUMAN | IDFGTHISGR | 2 | y4 | 1 | heavy | U | U3 | 6 | 827360 |
| sp\|P50579\|AMPM2_HUMAN | IDFGTHISGR | 2 | y3 | 1 | heavy | U | U3 | 6 | 287122 |
| sp\|P50579\|AMPM2_HUMAN | NLNGHSIGQYR | 2 | y9 | 1 | light | U | U3 | 6 | 3567 |
| sp\|P50579\|AMPM2_HUMAN | NLNGHSIGQYR | 2 | y6 | 1 | light | U | U3 | 6 | 5170 |
| sp\|P50579\|AMPM2_HUMAN | NLNGHSIGQYR | 2 | y5 | 1 | light | U | U3 | 6 | 2236 |
| sp\|P50579\|AMPM2_HUMAN | NLNGHSIGQYR | 2 | y9 | 1 | heavy | U | U3 | 6 | 156627 |
| sp\|P50579\|AMPM2_HUMAN | NLNGHSIGQYR | 2 | y6 | 1 | heavy | U | U3 | 6 | 421191 |
| sp\|P50579\|AMPM2_HUMAN | NLNGHSIGQYR | 2 | y5 | 1 | heavy | U | U3 | 6 | 114343 |
| sp\|P50579\|AMPM2_HUMAN | ALDQASEEIWNDFR | 2 | y9 | 1 | light | H | H3 | 7 | 7307 |
| sp\|P50579\|AMPM2_HUMAN | ALDQASEEIWNDFR | 2 | y7 | 1 | light | H | H3 | 7 | 4560 |
| sp\|P50579\|AMPM2_HUMAN | ALDQASEEIWNDFR | 2 | y6 | 1 | light | H | H3 | 7 | 3025 |
| sp\|P50579\|AMPM2_HUMAN | ALDQASEEIWNDFR | 2 | y9 | 1 | heavy | H | H3 | 7 | 200642 |
| sp\|P50579\|AMPM2_HUMAN | ALDQASEEIWNDFR | 2 | y7 | 1 | heavy | H | H3 | 7 | 131103 |
| sp\|P50579\|AMPM2_HUMAN | ALDQASEEIWNDFR | 2 | y6 | 1 | heavy | H | H3 | 7 | 122741 |
| sp\|P50579\|AMPM2_HUMAN | IDFGTHISGR | 2 | y5 | 1 | light | H | H3 | 7 | 14736 |
| sp\|P50579\|AMPM2_HUMAN | IDFGTHISGR | 2 | y4 | 1 | light | H | H3 | 7 | 14562 |
| sp\|P50579\|AMPM2_HUMAN | IDFGTHISGR | 2 | y3 | 1 | light | H | H3 | 7 | 8187 |
| sp\|P50579\|AMPM2_HUMAN | IDFGTHISGR | 2 | y5 | 1 | heavy | H | H3 | 7 | 373173 |
| sp\|P50579\|AMPM2_HUMAN | IDFGTHISGR | 2 | y4 | 1 | heavy | H | H3 | 7 | 970750 |
| sp\|P50579\|AMPM2_HUMAN | IDFGTHISGR | 2 | y3 | 1 | heavy | H | H3 | 7 | 332544 |
| sp\|P50579\|AMPM2_HUMAN | NLNGHSIGQYR | 2 | y9 | 1 | light | H | H3 | 7 | 3539 |
| sp\|P50579\|AMPM2_HUMAN | NLNGHSIGQYR | 2 | y6 | 1 | light | H | H3 | 7 | 3083 |
| sp\|P50579\|AMPM2_HUMAN | NLNGHSIGQYR | 2 | y5 | 1 | light | H | H3 | 7 | 691 |
| sp\|P50579\|AMPM2_HUMAN | NLNGHSIGQYR | 2 | y9 | 1 | heavy | H | H3 | 7 | 154329 |
| sp\|P50579\|AMPM2_HUMAN | NLNGHSIGQYR | 2 | y6 | 1 | heavy | H | H3 | 7 | 395218 |
| sp\|P50579\|AMPM2_HUMAN | NLNGHSIGQYR | 2 | y5 | 1 | heavy | H | H3 | 7 | 115280 |
| sp\|P50579\|AMPM2_HUMAN | ALDQASEEIWNDFR | 2 | y9 | 1 | light | K | K3 | 8 | 9199 |
| sp\|P50579\|AMPM2_HUMAN | ALDQASEEIWNDFR | 2 | y7 | 1 | light | K | K3 | 8 | 8811 |
| sp\|P50579\|AMPM2_HUMAN | ALDQASEEIWNDFR | 2 | y6 | 1 | light | K | K3 | 8 | 5949 |
| sp\|P50579\|AMPM2_HUMAN | ALDQASEEIWNDFR | 2 | y9 | 1 | heavy | K | K3 | 8 | 203593 |
| sp\|P50579\|AMPM2_HUMAN | ALDQASEEIWNDFR | 2 | y7 | 1 | heavy | K | K3 | 8 | 127579 |
| sp\|P50579\|AMPM2_HUMAN | ALDQASEEIWNDFR | 2 | y6 | 1 | heavy | K | K3 | 8 | 118893 |
| sp\|P50579\|AMPM2_HUMAN | IDFGTHISGR | 2 | y5 | 1 | light | K | K3 | 8 | 22048 |
| sp\|P50579\|AMPM2_HUMAN | IDFGTHISGR | 2 | y4 | 1 | light | K | K3 | 8 | 32369 |
| sp\|P50579\|AMPM2_HUMAN | IDFGTHISGR | 2 | y3 | 1 | light | K | K3 | 8 | 18501 |
| sp\|P50579\|AMPM2_HUMAN | IDFGTHISGR | 2 | y5 | 1 | heavy | K | K3 | 8 | 345925 |
| sp\|P50579\|AMPM2_HUMAN | IDFGTHISGR | 2 | y4 | 1 | heavy | K | K3 | 8 | 882210 |
| sp\|P50579\|AMPM2_HUMAN | IDFGTHISGR | 2 | y3 | 1 | heavy | K | K3 | 8 | 307829 |
| sp\|P50579\|AMPM2_HUMAN | NLNGHSIGQYR | 2 | y9 | 1 | light | K | K3 | 8 | 4744 |
| sp\|P50579\|AMPM2_HUMAN | NLNGHSIGQYR | 2 | y6 | 1 | light | K | K3 | 8 | 13188 |
| sp\|P50579\|AMPM2_HUMAN | NLNGHSIGQYR | 2 | y5 | 1 | light | K | K3 | 8 | 3498 |
| sp\|P50579\|AMPM2_HUMAN | NLNGHSIGQYR | 2 | y9 | 1 | heavy | K | K3 | 8 | 194805 |
| sp\|P50579\|AMPM2_HUMAN | NLNGHSIGQYR | 2 | y6 | 1 | heavy | K | K3 | 8 | 437533 |
| sp\|P50579\|AMPM2_HUMAN | NLNGHSIGQYR | 2 | y5 | 1 | heavy | K | K3 | 8 | 128930 |
| sp\|P50579\|AMPM2_HUMAN | ALDQASEEIWNDFR | 2 | y9 | 1 | light | U | U4 | 9 | 4053 |
| sp\|P50579\|AMPM2_HUMAN | ALDQASEEIWNDFR | 2 | y7 | 1 | light | U | U4 | 9 | 2377 |
| sp\|P50579\|AMPM2_HUMAN | ALDQASEEIWNDFR | 2 | y6 | 1 | light | U | U4 | 9 | 2826 |
| sp\|P50579\|AMPM2_HUMAN | ALDQASEEIWNDFR | 2 | y9 | 1 | heavy | U | U4 | 9 | 179672 |
| sp\|P50579\|AMPM2_HUMAN | ALDQASEEIWNDFR | 2 | y7 | 1 | heavy | U | U4 | 9 | 121977 |
| sp\|P50579\|AMPM2_HUMAN | ALDQASEEIWNDFR | 2 | y6 | 1 | heavy | U | U4 | 9 | 108396 |
| sp\|P50579\|AMPM2_HUMAN | IDFGTHISGR | 2 | y5 | 1 | light | U | U4 | 9 | 5640 |
| sp\|P50579\|AMPM2_HUMAN | IDFGTHISGR | 2 | y4 | 1 | light | U | U4 | 9 | 8545 |
| sp\|P50579\|AMPM2_HUMAN | IDFGTHISGR | 2 | y3 | 1 | light | U | U4 | 9 | 6327 |
| sp\|P50579\|AMPM2_HUMAN | IDFGTHISGR | 2 | y5 | 1 | heavy | U | U4 | 9 | 347519 |
| sp\|P50579\|AMPM2_HUMAN | IDFGTHISGR | 2 | y4 | 1 | heavy | U | U4 | 9 | 899558 |
| sp\|P50579\|AMPM2_HUMAN | IDFGTHISGR | 2 | y3 | 1 | heavy | U | U4 | 9 | 297311 |
| sp\|P50579\|AMPM2_HUMAN | NLNGHSIGQYR | 2 | y9 | 1 | light | U | U4 | 9 | 5351 |
| sp\|P50579\|AMPM2_HUMAN | NLNGHSIGQYR | 2 | y6 | 1 | light | U | U4 | 9 | 5078 |
| sp\|P50579\|AMPM2_HUMAN | NLNGHSIGQYR | 2 | y5 | 1 | light | U | U4 | 9 | 1836 |
| sp\|P50579\|AMPM2_HUMAN | NLNGHSIGQYR | 2 | y9 | 1 | heavy | U | U4 | 9 | 151839 |
| sp\|P50579\|AMPM2_HUMAN | NLNGHSIGQYR | 2 | y6 | 1 | heavy | U | U4 | 9 | 382052 |
| sp\|P50579\|AMPM2_HUMAN | NLNGHSIGQYR | 2 | y5 | 1 | heavy | U | U4 | 9 | 106854 |
| sp\|P50579\|AMPM2_HUMAN | ALDQASEEIWNDFR | 2 | y9 | 1 | light | H | H4 | 10 | 3149 |
| sp\|P50579\|AMPM2_HUMAN | ALDQASEEIWNDFR | 2 | y7 | 1 | light | H | H4 | 10 | 2283 |
| sp\|P50579\|AMPM2_HUMAN | ALDQASEEIWNDFR | 2 | y6 | 1 | light | H | H4 | 10 | 3925 |
| sp\|P50579\|AMPM2_HUMAN | ALDQASEEIWNDFR | 2 | y9 | 1 | heavy | H | H4 | 10 | 206035 |
| sp\|P50579\|AMPM2_HUMAN | ALDQASEEIWNDFR | 2 | y7 | 1 | heavy | H | H4 | 10 | 130399 |
| sp\|P50579\|AMPM2_HUMAN | ALDQASEEIWNDFR | 2 | y6 | 1 | heavy | H | H4 | 10 | 130795 |
| sp\|P50579\|AMPM2_HUMAN | IDFGTHISGR | 2 | y5 | 1 | light | H | H4 | 10 | 11800 |
| sp\|P50579\|AMPM2_HUMAN | IDFGTHISGR | 2 | y4 | 1 | light | H | H4 | 10 | 8717 |
| sp\|P50579\|AMPM2_HUMAN | IDFGTHISGR | 2 | y3 | 1 | light | H | H4 | 10 | 4435 |
| sp\|P50579\|AMPM2_HUMAN | IDFGTHISGR | 2 | y5 | 1 | heavy | H | H4 | 10 | 349842 |
| sp\|P50579\|AMPM2_HUMAN | IDFGTHISGR | 2 | y4 | 1 | heavy | H | H4 | 10 | 909716 |
| sp\|P50579\|AMPM2_HUMAN | IDFGTHISGR | 2 | y3 | 1 | heavy | H | H4 | 10 | 306042 |
| sp\|P50579\|AMPM2_HUMAN | NLNGHSIGQYR | 2 | y9 | 1 | light | H | H4 | 10 | 3082 |
| sp\|P50579\|AMPM2_HUMAN | NLNGHSIGQYR | 2 | y6 | 1 | light | H | H4 | 10 | 4934 |
| sp\|P50579\|AMPM2_HUMAN | NLNGHSIGQYR | 2 | y5 | 1 | light | H | H4 | 10 | 2783 |
| sp\|P50579\|AMPM2_HUMAN | NLNGHSIGQYR | 2 | y9 | 1 | heavy | H | H4 | 10 | 167509 |
| sp\|P50579\|AMPM2_HUMAN | NLNGHSIGQYR | 2 | y6 | 1 | heavy | H | H4 | 10 | 364034 |
| sp\|P50579\|AMPM2_HUMAN | NLNGHSIGQYR | 2 | y5 | 1 | heavy | H | H4 | 10 | 117744 |
| sp\|P50579\|AMPM2_HUMAN | ALDQASEEIWNDFR | 2 | y9 | 1 | light | K | K4 | 11 | 11876 |
| sp\|P50579\|AMPM2_HUMAN | ALDQASEEIWNDFR | 2 | y7 | 1 | light | K | K4 | 11 | 7301 |
| sp\|P50579\|AMPM2_HUMAN | ALDQASEEIWNDFR | 2 | y6 | 1 | light | K | K4 | 11 | 8871 |
| sp\|P50579\|AMPM2_HUMAN | ALDQASEEIWNDFR | 2 | y9 | 1 | heavy | K | K4 | 11 | 197311 |
| sp\|P50579\|AMPM2_HUMAN | ALDQASEEIWNDFR | 2 | y7 | 1 | heavy | K | K4 | 11 | 124395 |
| sp\|P50579\|AMPM2_HUMAN | ALDQASEEIWNDFR | 2 | y6 | 1 | heavy | K | K4 | 11 | 127360 |
| sp\|P50579\|AMPM2_HUMAN | IDFGTHISGR | 2 | y5 | 1 | light | K | K4 | 11 | 29432 |
| sp\|P50579\|AMPM2_HUMAN | IDFGTHISGR | 2 | y4 | 1 | light | K | K4 | 11 | 35646 |
| sp\|P50579\|AMPM2_HUMAN | IDFGTHISGR | 2 | y3 | 1 | light | K | K4 | 11 | 19586 |
| sp\|P50579\|AMPM2_HUMAN | IDFGTHISGR | 2 | y5 | 1 | heavy | K | K4 | 11 | 380395 |
| sp\|P50579\|AMPM2_HUMAN | IDFGTHISGR | 2 | y4 | 1 | heavy | K | K4 | 11 | 886222 |
| sp\|P50579\|AMPM2_HUMAN | IDFGTHISGR | 2 | y3 | 1 | heavy | K | K4 | 11 | 304420 |
| sp\|P50579\|AMPM2_HUMAN | NLNGHSIGQYR | 2 | y9 | 1 | light | K | K4 | 11 | 7081 |
| sp\|P50579\|AMPM2_HUMAN | NLNGHSIGQYR | 2 | y6 | 1 | light | K | K4 | 11 | 11650 |
| sp\|P50579\|AMPM2_HUMAN | NLNGHSIGQYR | 2 | y5 | 1 | light | K | K4 | 11 | 5097 |
| sp\|P50579\|AMPM2_HUMAN | NLNGHSIGQYR | 2 | y9 | 1 | heavy | K | K4 | 11 | 158571 |
| sp\|P50579\|AMPM2_HUMAN | NLNGHSIGQYR | 2 | y6 | 1 | heavy | K | K4 | 11 | 415260 |
| sp\|P50579\|AMPM2_HUMAN | NLNGHSIGQYR | 2 | y5 | 1 | heavy | K | K4 | 11 | 122902 |
| sp\|P53582\|AMPM1_HUMAN | LQCPTCIK | 2 | y6 | 1 | light | H | H1 | 1 | 52018 |
| sp\|P53582\|AMPM1_HUMAN | LQCPTCIK | 2 | y5 | 1 | light | H | H1 | 1 | 26028 |
| sp\|P53582\|AMPM1_HUMAN | LQCPTCIK | 2 | y4 | 1 | light | H | H1 | 1 | 5797 |
| sp\|P53582\|AMPM1_HUMAN | LQCPTCIK | 2 | y6 | 1 | heavy | H | H1 | 1 | 228362 |
| sp\|P53582\|AMPM1_HUMAN | LQCPTCIK | 2 | y5 | 1 | heavy | H | H1 | 1 | 95725 |
| sp\|P53582\|AMPM1_HUMAN | LQCPTCIK | 2 | y4 | 1 | heavy | H | H1 | 1 | 21309 |
| sp\|P53582\|AMPM1_HUMAN | LGIQGSYFCSQECFK | 2 | y11 | 1 | light | H | H1 | 1 | 2607 |
| sp\|P53582\|AMPM1_HUMAN | LGIQGSYFCSQECFK | 2 | y9 | 1 | light | H | H1 | 1 | 1742 |
| sp\|P53582\|AMPM1_HUMAN | LGIQGSYFCSQECFK | 2 | y8 | 1 | light | H | H1 | 1 | 3570 |
| sp\|P53582\|AMPM1_HUMAN | LGIQGSYFCSQECFK | 2 | y11 | 1 | heavy | H | H1 | 1 | 265524 |
| sp\|P53582\|AMPM1_HUMAN | LGIQGSYFCSQECFK | 2 | y9 | 1 | heavy | H | H1 | 1 | 144535 |
| sp\|P53582\|AMPM1_HUMAN | LGIQGSYFCSQECFK | 2 | y8 | 1 | heavy | H | H1 | 1 | 294782 |
| sp\|P53582\|AMPM1_HUMAN | HAQANGFSVVR | 2 | y10 | 1 | light | H | H1 | 1 | 4013 |
| sp\|P53582\|AMPM1_HUMAN | HAQANGFSVVR | 2 | y9 | 1 | light | H | H1 | 1 | 5287 |
| sp\|P53582\|AMPM1_HUMAN | HAQANGFSVVR | 2 | y8 | 1 | light | H | H1 | 1 | 3131 |
| sp\|P53582\|AMPM1_HUMAN | HAQANGFSVVR | 2 | y10 | 1 | heavy | H | H1 | 1 | 220019 |
| sp\|P53582\|AMPM1_HUMAN | HAQANGFSVVR | 2 | y9 | 1 | heavy | H | H1 | 1 | 385126 |
| sp\|P53582\|AMPM1_HUMAN | HAQANGFSVVR | 2 | y8 | 1 | heavy | H | H1 | 1 | 189334 |
| sp\|P53582\|AMPM1_HUMAN | LQCPTCIK | 2 | y6 | 1 | light | K | K1 | 2 | 67655 |
| sp\|P53582\|AMPM1_HUMAN | LQCPTCIK | 2 | y5 | 1 | light | K | K1 | 2 | 37751 |
| sp\|P53582\|AMPM1_HUMAN | LQCPTCIK | 2 | y4 | 1 | light | K | K1 | 2 | 4337 |
| sp\|P53582\|AMPM1_HUMAN | LQCPTCIK | 2 | y6 | 1 | heavy | K | K1 | 2 | 224506 |
| sp\|P53582\|AMPM1_HUMAN | LQCPTCIK | 2 | y5 | 1 | heavy | K | K1 | 2 | 99620 |
| sp\|P53582\|AMPM1_HUMAN | LQCPTCIK | 2 | y4 | 1 | heavy | K | K1 | 2 | 20817 |
| sp\|P53582\|AMPM1_HUMAN | LGIQGSYFCSQECFK | 2 | y11 | 1 | light | K | K1 | 2 | 3884 |
| sp\|P53582\|AMPM1_HUMAN | LGIQGSYFCSQECFK | 2 | y9 | 1 | light | K | K1 | 2 | 3350 |
| sp\|P53582\|AMPM1_HUMAN | LGIQGSYFCSQECFK | 2 | y8 | 1 | light | K | K1 | 2 | 5937 |
| sp\|P53582\|AMPM1_HUMAN | LGIQGSYFCSQECFK | 2 | y11 | 1 | heavy | K | K1 | 2 | 226726 |
| sp\|P53582\|AMPM1_HUMAN | LGIQGSYFCSQECFK | 2 | y9 | 1 | heavy | K | K1 | 2 | 144563 |
| sp\|P53582\|AMPM1_HUMAN | LGIQGSYFCSQECFK | 2 | y8 | 1 | heavy | K | K1 | 2 | 315829 |
| sp\|P53582\|AMPM1_HUMAN | HAQANGFSVVR | 2 | y10 | 1 | light | K | K1 | 2 | 5423 |
| sp\|P53582\|AMPM1_HUMAN | HAQANGFSVVR | 2 | y9 | 1 | light | K | K1 | 2 | 8782 |
| sp\|P53582\|AMPM1_HUMAN | HAQANGFSVVR | 2 | y8 | 1 | light | K | K1 | 2 | 5142 |
| sp\|P53582\|AMPM1_HUMAN | HAQANGFSVVR | 2 | y10 | 1 | heavy | K | K1 | 2 | 185893 |
| sp\|P53582\|AMPM1_HUMAN | HAQANGFSVVR | 2 | y9 | 1 | heavy | K | K1 | 2 | 320371 |
| sp\|P53582\|AMPM1_HUMAN | HAQANGFSVVR | 2 | y8 | 1 | heavy | K | K1 | 2 | 166369 |
| sp\|P53582\|AMPM1_HUMAN | LQCPTCIK | 2 | y6 | 1 | light | U | U2 | 3 | 64347 |
| sp\|P53582\|AMPM1_HUMAN | LQCPTCIK | 2 | y5 | 1 | light | U | U2 | 3 | 31785 |
| sp\|P53582\|AMPM1_HUMAN | LQCPTCIK | 2 | y4 | 1 | light | U | U2 | 3 | 9707 |
| sp\|P53582\|AMPM1_HUMAN | LQCPTCIK | 2 | y6 | 1 | heavy | U | U2 | 3 | 261764 |
| sp\|P53582\|AMPM1_HUMAN | LQCPTCIK | 2 | y5 | 1 | heavy | U | U2 | 3 | 121695 |
| sp\|P53582\|AMPM1_HUMAN | LQCPTCIK | 2 | y4 | 1 | heavy | U | U2 | 3 | 18969 |
| sp\|P53582\|AMPM1_HUMAN | LGIQGSYFCSQECFK | 2 | y11 | 1 | light | U | U2 | 3 | 1735 |
| sp\|P53582\|AMPM1_HUMAN | LGIQGSYFCSQECFK | 2 | y9 | 1 | light | U | U2 | 3 | 1419 |
| sp\|P53582\|AMPM1_HUMAN | LGIQGSYFCSQECFK | 2 | y8 | 1 | light | U | U2 | 3 | 3482 |
| sp\|P53582\|AMPM1_HUMAN | LGIQGSYFCSQECFK | 2 | y11 | 1 | heavy | U | U2 | 3 | 214098 |
| sp\|P53582\|AMPM1_HUMAN | LGIQGSYFCSQECFK | 2 | y9 | 1 | heavy | U | U2 | 3 | 127618 |
| sp\|P53582\|AMPM1_HUMAN | LGIQGSYFCSQECFK | 2 | y8 | 1 | heavy | U | U2 | 3 | 241861 |
| sp\|P53582\|AMPM1_HUMAN | HAQANGFSVVR | 2 | y10 | 1 | light | U | U2 | 3 | 6401 |
| sp\|P53582\|AMPM1_HUMAN | HAQANGFSVVR | 2 | y9 | 1 | light | U | U2 | 3 | 9441 |
| sp\|P53582\|AMPM1_HUMAN | HAQANGFSVVR | 2 | y8 | 1 | light | U | U2 | 3 | 4774 |
| sp\|P53582\|AMPM1_HUMAN | HAQANGFSVVR | 2 | y10 | 1 | heavy | U | U2 | 3 | 225851 |
| sp\|P53582\|AMPM1_HUMAN | HAQANGFSVVR | 2 | y9 | 1 | heavy | U | U2 | 3 | 357089 |
| sp\|P53582\|AMPM1_HUMAN | HAQANGFSVVR | 2 | y8 | 1 | heavy | U | U2 | 3 | 194062 |
| sp\|P53582\|AMPM1_HUMAN | LQCPTCIK | 2 | y6 | 1 | light | H | H2 | 4 | 59910 |
| sp\|P53582\|AMPM1_HUMAN | LQCPTCIK | 2 | y5 | 1 | light | H | H2 | 4 | 31685 |
| sp\|P53582\|AMPM1_HUMAN | LQCPTCIK | 2 | y4 | 1 | light | H | H2 | 4 | 8252 |
| sp\|P53582\|AMPM1_HUMAN | LQCPTCIK | 2 | y6 | 1 | heavy | H | H2 | 4 | 302468 |
| sp\|P53582\|AMPM1_HUMAN | LQCPTCIK | 2 | y5 | 1 | heavy | H | H2 | 4 | 135110 |
| sp\|P53582\|AMPM1_HUMAN | LQCPTCIK | 2 | y4 | 1 | heavy | H | H2 | 4 | 20228 |
| sp\|P53582\|AMPM1_HUMAN | LGIQGSYFCSQECFK | 2 | y11 | 1 | light | H | H2 | 4 | 3233 |
| sp\|P53582\|AMPM1_HUMAN | LGIQGSYFCSQECFK | 2 | y9 | 1 | light | H | H2 | 4 | 3652 |
| sp\|P53582\|AMPM1_HUMAN | LGIQGSYFCSQECFK | 2 | y8 | 1 | light | H | H2 | 4 | 2762 |
| sp\|P53582\|AMPM1_HUMAN | LGIQGSYFCSQECFK | 2 | y11 | 1 | heavy | H | H2 | 4 | 255716 |
| sp\|P53582\|AMPM1_HUMAN | LGIQGSYFCSQECFK | 2 | y9 | 1 | heavy | H | H2 | 4 | 156598 |
| sp\|P53582\|AMPM1_HUMAN | LGIQGSYFCSQECFK | 2 | y8 | 1 | heavy | H | H2 | 4 | 319495 |
| sp\|P53582\|AMPM1_HUMAN | HAQANGFSVVR | 2 | y10 | 1 | light | H | H2 | 4 | 4188 |
| sp\|P53582\|AMPM1_HUMAN | HAQANGFSVVR | 2 | y9 | 1 | light | H | H2 | 4 | 7586 |
| sp\|P53582\|AMPM1_HUMAN | HAQANGFSVVR | 2 | y8 | 1 | light | H | H2 | 4 | 2607 |
| sp\|P53582\|AMPM1_HUMAN | HAQANGFSVVR | 2 | y10 | 1 | heavy | H | H2 | 4 | 252466 |
| sp\|P53582\|AMPM1_HUMAN | HAQANGFSVVR | 2 | y9 | 1 | heavy | H | H2 | 4 | 405038 |
| sp\|P53582\|AMPM1_HUMAN | HAQANGFSVVR | 2 | y8 | 1 | heavy | H | H2 | 4 | 201062 |
| sp\|P53582\|AMPM1_HUMAN | LQCPTCIK | 2 | y6 | 1 | light | K | K2 | 5 | 76859 |
| sp\|P53582\|AMPM1_HUMAN | LQCPTCIK | 2 | y5 | 1 | light | K | K2 | 5 | 46519 |
| sp\|P53582\|AMPM1_HUMAN | LQCPTCIK | 2 | y4 | 1 | light | K | K2 | 5 | 12357 |
| sp\|P53582\|AMPM1_HUMAN | LQCPTCIK | 2 | y6 | 1 | heavy | K | K2 | 5 | 276193 |
| sp\|P53582\|AMPM1_HUMAN | LQCPTCIK | 2 | y5 | 1 | heavy | K | K2 | 5 | 128441 |
| sp\|P53582\|AMPM1_HUMAN | LQCPTCIK | 2 | y4 | 1 | heavy | K | K2 | 5 | 20080 |
| sp\|P53582\|AMPM1_HUMAN | LGIQGSYFCSQECFK | 2 | y11 | 1 | light | K | K2 | 5 | 4981 |
| sp\|P53582\|AMPM1_HUMAN | LGIQGSYFCSQECFK | 2 | y9 | 1 | light | K | K2 | 5 | 2402 |
| sp\|P53582\|AMPM1_HUMAN | LGIQGSYFCSQECFK | 2 | y8 | 1 | light | K | K2 | 5 | 5568 |
| sp\|P53582\|AMPM1_HUMAN | LGIQGSYFCSQECFK | 2 | y11 | 1 | heavy | K | K2 | 5 | 247308 |
| sp\|P53582\|AMPM1_HUMAN | LGIQGSYFCSQECFK | 2 | y9 | 1 | heavy | K | K2 | 5 | 146618 |
| sp\|P53582\|AMPM1_HUMAN | LGIQGSYFCSQECFK | 2 | y8 | 1 | heavy | K | K2 | 5 | 298130 |
| sp\|P53582\|AMPM1_HUMAN | HAQANGFSVVR | 2 | y10 | 1 | light | K | K2 | 5 | 7000 |
| sp\|P53582\|AMPM1_HUMAN | HAQANGFSVVR | 2 | y9 | 1 | light | K | K2 | 5 | 10728 |
| sp\|P53582\|AMPM1_HUMAN | HAQANGFSVVR | 2 | y8 | 1 | light | K | K2 | 5 | 5511 |
| sp\|P53582\|AMPM1_HUMAN | HAQANGFSVVR | 2 | y10 | 1 | heavy | K | K2 | 5 | 252050 |
| sp\|P53582\|AMPM1_HUMAN | HAQANGFSVVR | 2 | y9 | 1 | heavy | K | K2 | 5 | 437702 |
| sp\|P53582\|AMPM1_HUMAN | HAQANGFSVVR | 2 | y8 | 1 | heavy | K | K2 | 5 | 209810 |
| sp\|P53582\|AMPM1_HUMAN | LQCPTCIK | 2 | y6 | 1 | light | U | U3 | 6 | 63547 |
| sp\|P53582\|AMPM1_HUMAN | LQCPTCIK | 2 | y5 | 1 | light | U | U3 | 6 | 35228 |
| sp\|P53582\|AMPM1_HUMAN | LQCPTCIK | 2 | y4 | 1 | light | U | U3 | 6 | 7967 |
| sp\|P53582\|AMPM1_HUMAN | LQCPTCIK | 2 | y6 | 1 | heavy | U | U3 | 6 | 289831 |
| sp\|P53582\|AMPM1_HUMAN | LQCPTCIK | 2 | y5 | 1 | heavy | U | U3 | 6 | 131685 |
| sp\|P53582\|AMPM1_HUMAN | LQCPTCIK | 2 | y4 | 1 | heavy | U | U3 | 6 | 18710 |
| sp\|P53582\|AMPM1_HUMAN | LGIQGSYFCSQECFK | 2 | y11 | 1 | light | U | U3 | 6 | 3282 |
| sp\|P53582\|AMPM1_HUMAN | LGIQGSYFCSQECFK | 2 | y9 | 1 | light | U | U3 | 6 | 2628 |
| sp\|P53582\|AMPM1_HUMAN | LGIQGSYFCSQECFK | 2 | y8 | 1 | light | U | U3 | 6 | 4348 |
| sp\|P53582\|AMPM1_HUMAN | LGIQGSYFCSQECFK | 2 | y11 | 1 | heavy | U | U3 | 6 | 251549 |
| sp\|P53582\|AMPM1_HUMAN | LGIQGSYFCSQECFK | 2 | y9 | 1 | heavy | U | U3 | 6 | 133309 |
| sp\|P53582\|AMPM1_HUMAN | LGIQGSYFCSQECFK | 2 | y8 | 1 | heavy | U | U3 | 6 | 277971 |
| sp\|P53582\|AMPM1_HUMAN | HAQANGFSVVR | 2 | y10 | 1 | light | U | U3 | 6 | 7663 |
| sp\|P53582\|AMPM1_HUMAN | HAQANGFSVVR | 2 | y9 | 1 | light | U | U3 | 6 | 11836 |
| sp\|P53582\|AMPM1_HUMAN | HAQANGFSVVR | 2 | y8 | 1 | light | U | U3 | 6 | 3151 |
| sp\|P53582\|AMPM1_HUMAN | HAQANGFSVVR | 2 | y10 | 1 | heavy | U | U3 | 6 | 245857 |
| sp\|P53582\|AMPM1_HUMAN | HAQANGFSVVR | 2 | y9 | 1 | heavy | U | U3 | 6 | 423012 |
| sp\|P53582\|AMPM1_HUMAN | HAQANGFSVVR | 2 | y8 | 1 | heavy | U | U3 | 6 | 206743 |
| sp\|P53582\|AMPM1_HUMAN | LQCPTCIK | 2 | y6 | 1 | light | H | H3 | 7 | 59428 |
| sp\|P53582\|AMPM1_HUMAN | LQCPTCIK | 2 | y5 | 1 | light | H | H3 | 7 | 32538 |
| sp\|P53582\|AMPM1_HUMAN | LQCPTCIK | 2 | y4 | 1 | light | H | H3 | 7 | 9390 |
| sp\|P53582\|AMPM1_HUMAN | LQCPTCIK | 2 | y6 | 1 | heavy | H | H3 | 7 | 301255 |
| sp\|P53582\|AMPM1_HUMAN | LQCPTCIK | 2 | y5 | 1 | heavy | H | H3 | 7 | 148068 |
| sp\|P53582\|AMPM1_HUMAN | LQCPTCIK | 2 | y4 | 1 | heavy | H | H3 | 7 | 15673 |
| sp\|P53582\|AMPM1_HUMAN | LGIQGSYFCSQECFK | 2 | y11 | 1 | light | H | H3 | 7 | 2442 |
| sp\|P53582\|AMPM1_HUMAN | LGIQGSYFCSQECFK | 2 | y9 | 1 | light | H | H3 | 7 | 2102 |
| sp\|P53582\|AMPM1_HUMAN | LGIQGSYFCSQECFK | 2 | y8 | 1 | light | H | H3 | 7 | 5260 |
| sp\|P53582\|AMPM1_HUMAN | LGIQGSYFCSQECFK | 2 | y11 | 1 | heavy | H | H3 | 7 | 267208 |
| sp\|P53582\|AMPM1_HUMAN | LGIQGSYFCSQECFK | 2 | y9 | 1 | heavy | H | H3 | 7 | 153655 |
| sp\|P53582\|AMPM1_HUMAN | LGIQGSYFCSQECFK | 2 | y8 | 1 | heavy | H | H3 | 7 | 320775 |
| sp\|P53582\|AMPM1_HUMAN | HAQANGFSVVR | 2 | y10 | 1 | light | H | H3 | 7 | 6392 |
| sp\|P53582\|AMPM1_HUMAN | HAQANGFSVVR | 2 | y9 | 1 | light | H | H3 | 7 | 8986 |
| sp\|P53582\|AMPM1_HUMAN | HAQANGFSVVR | 2 | y8 | 1 | light | H | H3 | 7 | 4617 |
| sp\|P53582\|AMPM1_HUMAN | HAQANGFSVVR | 2 | y10 | 1 | heavy | H | H3 | 7 | 278842 |
| sp\|P53582\|AMPM1_HUMAN | HAQANGFSVVR | 2 | y9 | 1 | heavy | H | H3 | 7 | 483950 |
| sp\|P53582\|AMPM1_HUMAN | HAQANGFSVVR | 2 | y8 | 1 | heavy | H | H3 | 7 | 225456 |
| sp\|P53582\|AMPM1_HUMAN | LQCPTCIK | 2 | y6 | 1 | light | K | K3 | 8 | 84286 |
| sp\|P53582\|AMPM1_HUMAN | LQCPTCIK | 2 | y5 | 1 | light | K | K3 | 8 | 48450 |
| sp\|P53582\|AMPM1_HUMAN | LQCPTCIK | 2 | y4 | 1 | light | K | K3 | 8 | 12539 |
| sp\|P53582\|AMPM1_HUMAN | LQCPTCIK | 2 | y6 | 1 | heavy | K | K3 | 8 | 273128 |
| sp\|P53582\|AMPM1_HUMAN | LQCPTCIK | 2 | y5 | 1 | heavy | K | K3 | 8 | 123126 |
| sp\|P53582\|AMPM1_HUMAN | LQCPTCIK | 2 | y4 | 1 | heavy | K | K3 | 8 | 20694 |
| sp\|P53582\|AMPM1_HUMAN | LGIQGSYFCSQECFK | 2 | y11 | 1 | light | K | K3 | 8 | 2369 |
| sp\|P53582\|AMPM1_HUMAN | LGIQGSYFCSQECFK | 2 | y9 | 1 | light | K | K3 | 8 | 3633 |
| sp\|P53582\|AMPM1_HUMAN | LGIQGSYFCSQECFK | 2 | y8 | 1 | light | K | K3 | 8 | 5851 |
| sp\|P53582\|AMPM1_HUMAN | LGIQGSYFCSQECFK | 2 | y11 | 1 | heavy | K | K3 | 8 | 254488 |
| sp\|P53582\|AMPM1_HUMAN | LGIQGSYFCSQECFK | 2 | y9 | 1 | heavy | K | K3 | 8 | 146859 |
| sp\|P53582\|AMPM1_HUMAN | LGIQGSYFCSQECFK | 2 | y8 | 1 | heavy | K | K3 | 8 | 299634 |
| sp\|P53582\|AMPM1_HUMAN | HAQANGFSVVR | 2 | y10 | 1 | light | K | K3 | 8 | 4581 |
| sp\|P53582\|AMPM1_HUMAN | HAQANGFSVVR | 2 | y9 | 1 | light | K | K3 | 8 | 12940 |
| sp\|P53582\|AMPM1_HUMAN | HAQANGFSVVR | 2 | y8 | 1 | light | K | K3 | 8 | 8176 |
| sp\|P53582\|AMPM1_HUMAN | HAQANGFSVVR | 2 | y10 | 1 | heavy | K | K3 | 8 | 259299 |
| sp\|P53582\|AMPM1_HUMAN | HAQANGFSVVR | 2 | y9 | 1 | heavy | K | K3 | 8 | 424000 |
| sp\|P53582\|AMPM1_HUMAN | HAQANGFSVVR | 2 | y8 | 1 | heavy | K | K3 | 8 | 207115 |
| sp\|P53582\|AMPM1_HUMAN | LQCPTCIK | 2 | y6 | 1 | light | U | U4 | 9 | 68477 |
| sp\|P53582\|AMPM1_HUMAN | LQCPTCIK | 2 | y5 | 1 | light | U | U4 | 9 | 36218 |
| sp\|P53582\|AMPM1_HUMAN | LQCPTCIK | 2 | y4 | 1 | light | U | U4 | 9 | 5766 |
| sp\|P53582\|AMPM1_HUMAN | LQCPTCIK | 2 | y6 | 1 | heavy | U | U4 | 9 | 332984 |
| sp\|P53582\|AMPM1_HUMAN | LQCPTCIK | 2 | y5 | 1 | heavy | U | U4 | 9 | 141657 |
| sp\|P53582\|AMPM1_HUMAN | LQCPTCIK | 2 | y4 | 1 | heavy | U | U4 | 9 | 21307 |
| sp\|P53582\|AMPM1_HUMAN | LGIQGSYFCSQECFK | 2 | y11 | 1 | light | U | U4 | 9 | 2290 |
| sp\|P53582\|AMPM1_HUMAN | LGIQGSYFCSQECFK | 2 | y9 | 1 | light | U | U4 | 9 | 2318 |
| sp\|P53582\|AMPM1_HUMAN | LGIQGSYFCSQECFK | 2 | y8 | 1 | light | U | U4 | 9 | 4328 |
| sp\|P53582\|AMPM1_HUMAN | LGIQGSYFCSQECFK | 2 | y11 | 1 | heavy | U | U4 | 9 | 248194 |
| sp\|P53582\|AMPM1_HUMAN | LGIQGSYFCSQECFK | 2 | y9 | 1 | heavy | U | U4 | 9 | 137105 |
| sp\|P53582\|AMPM1_HUMAN | LGIQGSYFCSQECFK | 2 | y8 | 1 | heavy | U | U4 | 9 | 302576 |
| sp\|P53582\|AMPM1_HUMAN | HAQANGFSVVR | 2 | y10 | 1 | light | U | U4 | 9 | 7868 |
| sp\|P53582\|AMPM1_HUMAN | HAQANGFSVVR | 2 | y9 | 1 | light | U | U4 | 9 | 11758 |
| sp\|P53582\|AMPM1_HUMAN | HAQANGFSVVR | 2 | y8 | 1 | light | U | U4 | 9 | 5350 |
| sp\|P53582\|AMPM1_HUMAN | HAQANGFSVVR | 2 | y10 | 1 | heavy | U | U4 | 9 | 278703 |
| sp\|P53582\|AMPM1_HUMAN | HAQANGFSVVR | 2 | y9 | 1 | heavy | U | U4 | 9 | 461412 |
| sp\|P53582\|AMPM1_HUMAN | HAQANGFSVVR | 2 | y8 | 1 | heavy | U | U4 | 9 | 222549 |
| sp\|P53582\|AMPM1_HUMAN | LQCPTCIK | 2 | y6 | 1 | light | H | H4 | 10 | 63599 |
| sp\|P53582\|AMPM1_HUMAN | LQCPTCIK | 2 | y5 | 1 | light | H | H4 | 10 | 33725 |
| sp\|P53582\|AMPM1_HUMAN | LQCPTCIK | 2 | y4 | 1 | light | H | H4 | 10 | 5206 |
| sp\|P53582\|AMPM1_HUMAN | LQCPTCIK | 2 | y6 | 1 | heavy | H | H4 | 10 | 341830 |
| sp\|P53582\|AMPM1_HUMAN | LQCPTCIK | 2 | y5 | 1 | heavy | H | H4 | 10 | 161384 |
| sp\|P53582\|AMPM1_HUMAN | LQCPTCIK | 2 | y4 | 1 | heavy | H | H4 | 10 | 20204 |
| sp\|P53582\|AMPM1_HUMAN | LGIQGSYFCSQECFK | 2 | y11 | 1 | light | H | H4 | 10 | 2413 |
| sp\|P53582\|AMPM1_HUMAN | LGIQGSYFCSQECFK | 2 | y9 | 1 | light | H | H4 | 10 | 1503 |
| sp\|P53582\|AMPM1_HUMAN | LGIQGSYFCSQECFK | 2 | y8 | 1 | light | H | H4 | 10 | 4234 |
| sp\|P53582\|AMPM1_HUMAN | LGIQGSYFCSQECFK | 2 | y11 | 1 | heavy | H | H4 | 10 | 266165 |
| sp\|P53582\|AMPM1_HUMAN | LGIQGSYFCSQECFK | 2 | y9 | 1 | heavy | H | H4 | 10 | 146279 |
| sp\|P53582\|AMPM1_HUMAN | LGIQGSYFCSQECFK | 2 | y8 | 1 | heavy | H | H4 | 10 | 328004 |
| sp\|P53582\|AMPM1_HUMAN | HAQANGFSVVR | 2 | y10 | 1 | light | H | H4 | 10 | 5318 |
| sp\|P53582\|AMPM1_HUMAN | HAQANGFSVVR | 2 | y9 | 1 | light | H | H4 | 10 | 9182 |
| sp\|P53582\|AMPM1_HUMAN | HAQANGFSVVR | 2 | y8 | 1 | light | H | H4 | 10 | 3373 |
| sp\|P53582\|AMPM1_HUMAN | HAQANGFSVVR | 2 | y10 | 1 | heavy | H | H4 | 10 | 217255 |
| sp\|P53582\|AMPM1_HUMAN | HAQANGFSVVR | 2 | y9 | 1 | heavy | H | H4 | 10 | 403149 |
| sp\|P53582\|AMPM1_HUMAN | HAQANGFSVVR | 2 | y8 | 1 | heavy | H | H4 | 10 | 204870 |
| sp\|P53582\|AMPM1_HUMAN | LQCPTCIK | 2 | y6 | 1 | light | K | K4 | 11 | 90672 |
| sp\|P53582\|AMPM1_HUMAN | LQCPTCIK | 2 | y5 | 1 | light | K | K4 | 11 | 53824 |
| sp\|P53582\|AMPM1_HUMAN | LQCPTCIK | 2 | y4 | 1 | light | K | K4 | 11 | 7210 |
| sp\|P53582\|AMPM1_HUMAN | LQCPTCIK | 2 | y6 | 1 | heavy | K | K4 | 11 | 300864 |
| sp\|P53582\|AMPM1_HUMAN | LQCPTCIK | 2 | y5 | 1 | heavy | K | K4 | 11 | 149055 |
| sp\|P53582\|AMPM1_HUMAN | LQCPTCIK | 2 | y4 | 1 | heavy | K | K4 | 11 | 23400 |
| sp\|P53582\|AMPM1_HUMAN | LGIQGSYFCSQECFK | 2 | y11 | 1 | light | K | K4 | 11 | 4422 |
| sp\|P53582\|AMPM1_HUMAN | LGIQGSYFCSQECFK | 2 | y9 | 1 | light | K | K4 | 11 | 2675 |
| sp\|P53582\|AMPM1_HUMAN | LGIQGSYFCSQECFK | 2 | y8 | 1 | light | K | K4 | 11 | 4262 |
| sp\|P53582\|AMPM1_HUMAN | LGIQGSYFCSQECFK | 2 | y11 | 1 | heavy | K | K4 | 11 | 253787 |
| sp\|P53582\|AMPM1_HUMAN | LGIQGSYFCSQECFK | 2 | y9 | 1 | heavy | K | K4 | 11 | 148851 |
| sp\|P53582\|AMPM1_HUMAN | LGIQGSYFCSQECFK | 2 | y8 | 1 | heavy | K | K4 | 11 | 286223 |
| sp\|P53582\|AMPM1_HUMAN | HAQANGFSVVR | 2 | y10 | 1 | light | K | K4 | 11 | 7515 |
| sp\|P53582\|AMPM1_HUMAN | HAQANGFSVVR | 2 | y9 | 1 | light | K | K4 | 11 | 12487 |
| sp\|P53582\|AMPM1_HUMAN | HAQANGFSVVR | 2 | y8 | 1 | light | K | K4 | 11 | 4835 |
| sp\|P53582\|AMPM1_HUMAN | HAQANGFSVVR | 2 | y10 | 1 | heavy | K | K4 | 11 | 264492 |
| sp\|P53582\|AMPM1_HUMAN | HAQANGFSVVR | 2 | y9 | 1 | heavy | K | K4 | 11 | 454904 |
| sp\|P53582\|AMPM1_HUMAN | HAQANGFSVVR | 2 | y8 | 1 | heavy | K | K4 | 11 | 226400 |

**AbsoluteQuant-Calibration Curve**

| **ProteinName** | **PeptideSequence** | **ReplicateName** | **Quantity injected in column (fmol)** | **Replicate** | **BestRetentionTime** | **TotalArea** |
| --- | --- | --- | --- | --- | --- | --- |
| sp\|P50579\|AMPM2_HUMAN | IDFGTHISGR | N12312 | 1.56 | 1 | 22.71 | 14021 |
| sp\|P50579\|AMPM2_HUMAN | IDFGTHISGR | N12320 | 1.56 | 2 | 22.81 | 15293 |
| sp\|P50579\|AMPM2_HUMAN | IDFGTHISGR | N12321 | 1.56 | 3 | 22.66 | 18564 |
| sp\|P50579\|AMPM2_HUMAN | IDFGTHISGR | N12313 | 3.125 | 1 | 22.66 | 31989 |
| sp\|P50579\|AMPM2_HUMAN | IDFGTHISGR | N12316_150723152113 | 3.125 | 2 | 22.81 | 36183 |
| sp\|P50579\|AMPM2_HUMAN | IDFGTHISGR | N12317 | 3.125 | 3 | 22.81 | 28014 |
| sp\|P50579\|AMPM2_HUMAN | IDFGTHISGR | N12282 | 6.25 | 1 | 22.95 | 53386 |
| sp\|P50579\|AMPM2_HUMAN | IDFGTHISGR | N12283 | 6.25 | 2 | 22.9 | 56037 |
| sp\|P50579\|AMPM2_HUMAN | IDFGTHISGR | N12284 | 6.25 | 3 | 22.9 | 49540 |
| sp\|P50579\|AMPM2_HUMAN | IDFGTHISGR | N12285 | 12.5 | 1 | 22.9 | 78919 |
| sp\|P50579\|AMPM2_HUMAN | IDFGTHISGR | N12286 | 12.5 | 2 | 22.76 | 74725 |
| sp\|P50579\|AMPM2_HUMAN | IDFGTHISGR | N12287 | 12.5 | 3 | 22.81 | 75969 |
| sp\|P50579\|AMPM2_HUMAN | IDFGTHISGR | N12288 | 25 | 1 | 22.76 | 112919 |
| sp\|P50579\|AMPM2_HUMAN | IDFGTHISGR | N12289 | 25 | 2 | 22.76 | 119678 |
| sp\|P50579\|AMPM2_HUMAN | IDFGTHISGR | N12290 | 25 | 3 | 22.76 | 125190 |
| sp\|P50579\|AMPM2_HUMAN | IDFGTHISGR | N12291 | 50 | 1 | 22.81 | 193267 |
| sp\|P50579\|AMPM2_HUMAN | IDFGTHISGR | N12292 | 50 | 2 | 22.76 | 205688 |
| sp\|P50579\|AMPM2_HUMAN | IDFGTHISGR | N12293 | 50 | 3 | 22.81 | 188214 |
|  |  |  |  |  |  |  |
|  |  |  |  |  |  |  |
|  |  |  |  |  |  |  |
|  |  |  |  |  |  |  |
|  |  |  |  |  |  |  |
| **Overall Retention time Mean** | **Overall Standard deviation** | **Overall Retention time CV (%)** |  |  |  |  |
| 22.8 | 0.08 | 0.35 |  |  |  |  |
|  |  |  |  |  |  |  |
|  |  |  |  |  |  |  |
|  |  |  |  |  |  |  |
|  |  |  |  |  |  |  |
| **Injected quantity on column (fmol)** | **Peak Area mean** | **Peak Area Standard Deviation** | **CV (%)** | **Accuracy (%)** |  |  |
| 1.56 | 1.60E+04 | 2.34E+03 | 15 | 66 |  |  |
| 3.125 | 3.21E+04 | 4.08E+03 | 13 | 98 |  |  |
| 6.25 | 5.30E+04 | 3.27E+03 | 6 | 108 |  |  |
| 12.5 | 7.65E+04 | 2.15E+03 | 3 | 96 |  |  |
| 25 | 1.19E+05 | 6.15E+03 | 5 | 96 |  |  |
| 50 | 1.96E+05 | 8.99E+03 | 5 | 104 |  |  |

**AbsoluteQuant-Samples**

| Raw DATA | | | | | | | | | | | |
| --- | --- | --- | --- | --- | --- | --- | --- | --- | --- | --- | --- |
| Protein Name | Peptide Sequence | Replicate Name | Condition | BioReplicate | File Name | light Precursor Mz | light Total Area | light Best Retention Time | heavy Precursor Mz | heavy Total Area | heavy Best Retention Time |
| sp\|P50579\|AMPM2_HUMAN | IDFGTHISGR | N12196 | HUVEC | 1 | N12196.raw | 368.192845 | 31540 | 21.99 | 371.528935 | 258893 | 21.99 |
| sp\|P50579\|AMPM2_HUMAN | IDFGTHISGR | N12197 | HUVEC | 2 | N12197.raw | 368.192845 | 29165 | 21.85 | 371.528935 | 254764 | 21.8 |
| sp\|P50579\|AMPM2_HUMAN | IDFGTHISGR | N12198 | HUVEC | 3 | N12198.raw | 368.192845 | 26842 | 22.09 | 371.528935 | 250767 | 22.09 |
| sp\|P50579\|AMPM2_HUMAN | IDFGTHISGR | N12201 | K562 | 1 | N12201.raw | 368.192845 | 36306 | 22.09 | 371.528935 | 303877 | 22.09 |
| sp\|P50579\|AMPM2_HUMAN | IDFGTHISGR | N12202 | K562 | 2 | N12202.raw | 368.192845 | 33827 | 22.23 | 371.528935 | 265678 | 22.28 |
| sp\|P50579\|AMPM2_HUMAN | IDFGTHISGR | N12203 | K562 | 3 | N12203.raw | 368.192845 | 34077 | 22.28 | 371.528935 | 252596 | 22.23 |
| sp\|P50579\|AMPM2_HUMAN | IDFGTHISGR | N12220 | U87 | 1 | N12220.raw | 368.192845 | 10192 | 22.52 | 371.528935 | 179102 | 22.43 |
| sp\|P50579\|AMPM2_HUMAN | IDFGTHISGR | N12221 | U87 | 2 | N12221.raw | 368.192845 | 14599 | 22.62 | 371.528935 | 181743 | 22.47 |
| sp\|P50579\|AMPM2_HUMAN | IDFGTHISGR | N12222 | U87 | 3 | N12222.raw | 368.192845 | 13010 | 22.52 | 371.528935 | 197864 | 22.52 |
|  |  |  |  |  |  |  |  |  |  |  |  |
|  |  |  |  |  |  |  |  |  |  |  |  |
| **Statistical description** | | | | |  |  |  |  |  |  |  |
|  | **Condition** | **IDFGTHISGR - Light 368,1928+++** | **IDFGTHISGR - heavy 371,5289+++** | **Peak Area Ratio To Heavy** |  |  |  |  |  |  |  |
| **Mean** | HUVEC | 29182.46 | 254807.91 | 1.14E-01 |  |  |  |  |  |  |  |
|  | K562 | 34736.69 | 274050.22 | 1.27E-01 |  |  |  |  |  |  |  |
|  | U87 | 12600.16 | 186236.46 | 6.77E-02 |  |  |  |  |  |  |  |
| **Standard deviation** | HUVEC | 2349.21 | 4062.96 | 7.39E-03 |  |  |  |  |  |  |  |
|  | K562 | 1364.44 | 26645.88 | 7.72E-03 |  |  |  |  |  |  |  |
|  | U87 | 2231.69 | 10156.05 | 1.18E-02 |  |  |  |  |  |  |  |
| **CV (%)** | HUVEC | 8.05 | 1.59 | 6.46 |  |  |  |  |  |  |  |
|  | K562 | 3.93 | 9.72 | 6.07 |  |  |  |  |  |  |  |
|  | U87 | 17.71 | 5.45 | 17.48 |  |  |  |  |  |  |  |
|  |  |  |  |  |  |  |  |  |  |  |  |
|  |  |  |  |  |  |  |  |  |  |  |  |
| **Overall Retention time Mean** | **Overall Standard deviation** | **Overall Retention time CV (%)** |  |  |  |  |  |  |  |  |  |
| 22.21 | 0.24 | 1.08 |  |  |  |  |  |  |  |  |  |
|  |  |  |  |  |  |  |  |  |  |  |  |
|  |  |  |  |  |  |  |  |  |  |  |  |
|  | **Calculated quantity injected in column (fmol)** | **Status** |  |  |  |  |  |  |  |  |  |
| **HUVEC** | 2.6 | below LOQ |  |  |  |  |  |  |  |  |  |
| **K562** | 3.5 | OK |  |  |  |  |  |  |  |  |  |
| **U87** | 0.7 | below LOQ |  |  |  |  |  |  |  |  |  |
